# The burden of hyperkalaemia in chronic kidney disease: a systematic literature review

**DOI:** 10.1093/ckj/sfaf127

**Published:** 2025-04-29

**Authors:** Jürgen Floege, Andrew H Frankel, Kevin F Erickson, Ketevan Rtveladze, Yogesh Punekar, Jahangir Nabi Mir, Jessica Walters, Alexandra Ehm, James Fotheringham

**Affiliations:** Division of Nephrology and Dept of Cardiology, RWTH Aachen University, Aachen, Germany; Imperial College Healthcare NHS Trust, London, UK; Baylor College of Medicine, TX, USA; IQVIA, London, UK; IQVIA, London, UK; IQVIA, Gurugram, Haryana, India; IQVIA, London, UK; Boehringer Ingelheim, Germany; School of Medicine and Population Health, University of Sheffield, Sheffield, UK

**Keywords:** CKD, epidemiology, health and economic outcomes, hyperkalaemia, RAASi, sub-optimal dosing

## Abstract

**Background:**

The global epidemiology and burden of hyperkalaemia in patients with chronic kidney disease (CKD) are unclear due to the inconsistent definitions of hyperkalaemia. The combination of adverse effects and interaction between comorbidity and pharmacotherapies, such as renin–angiotensin–aldosterone system inhibitors (RAASi), justify a systematic understanding of this common complication of CKD.

**Methods:**

This systematic literature review aimed to identify and descriptively summarize the evidence on hyperkalaemia risk factors and associated characteristics in adult CKD patients, including the effects of sub-optimal RAASi. Medline^®^ and Embase^®^ databases were searched from January 2000 to April 2024, with additional hand searching. Publications were screened by two independent reviewers. Data were extracted by one reviewer and verified by another reviewer; study quality assessment was also conducted.

**Results:**

A total of 138 studies described in 145 publications met the eligibility criteria. The published literature revealed varying prevalence of hyperkalaemia amongst inconsistent definitions and a significant increase in the prevalence and incidence of hyperkalaemia among patients with CKD, regardless of RAASi treatment. Hyperkalaemia was associated with adverse outcomes and increased hospital resource use. Additionally, studies pointed to negative health and economic outcomes due to sub-optimal RAASi dosing in CKD patients with hyperkalaemia, as well as in those with CKD and comorbid heart failure.

**Conclusions:**

This review expands on current research, offering a new perspective specifically focused on CKD patients and wider clinical and economic outcomes. Identification of wider clinical and economic consequences of hyperkalaemia in CKD patients, and the interplay between these risks and the risks of sub-optimal RAASi dosing, justify the need for future research. Clinicians should exercise caution when managing this condition in this complex patient group.

## Graphical Abstract

**Graphical Abstract:**
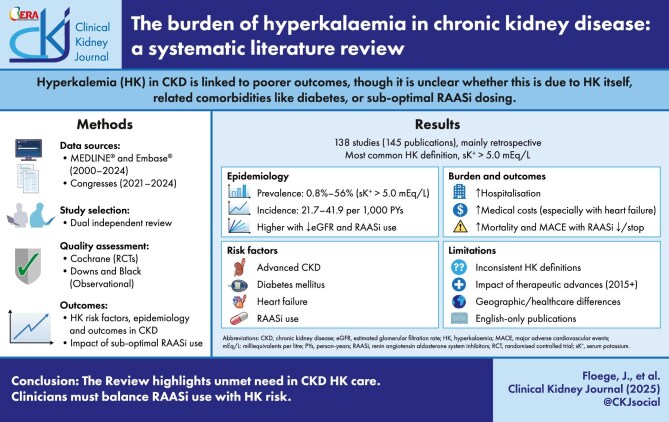

KEY LEARNING POINTS **What was known:**

- The global epidemiology of hyperkalaemia varies across different populations, definitions, healthcare settings, diseases and pharmacotherapies.
- The definition of hyperkalaemia is not well defined within clinical guidelines and current literature, with prevalence and incidence varying according to definition/threshold used.
- As new chronic kidney disease (CKD) pharmacotherapies emerge, and with increasing observation of multimorbid patients, this systematic literature review aimed to provide a contemporary assessment of epidemiology and burden specifically within adult CKD patients, due to their increased risk of hyperkalaemia and related events.

**This study adds:**

- There is an increased prevalence/incidence of hyperkalaemia in adult CKD patients, which is linked to adverse outcomes and increased hospital resource use.
- Modifying renin–angiotensin–aldosterone system inhibitors (RAASi) dose to lower serum potassium may have detrimental consequences on clinical outcomes of CKD patients, particularly in those with comorbid conditions like heart failure.
- There is substantial evidence of negative health and economic outcomes due to sub-optimal RAASi dosing in CKD patients with hyperkalaemia, particularly for those with CKD and comorbid conditions.

**Potential impact:**

- Treatments currently available for CKD patients with or at risk of chronic hyperkalaemia are still limited, presenting a specific unmet need, and careful consideration by clinicians is required when managing these multimorbid patients.

## INTRODUCTION

Although there is no clear definition for hyperkalaemia in guidelines and current literature, it is typically defined as serum potassium (sK^+^) levels exceeding the upper limit of the normal reference range, which is generally greater than 5.0 or 5.5 milliequivalents (mEq)/litre (L), though this may vary slightly between laboratories [1–4]. In clinical practice, hyperkalaemia is often classified by severity as mild (sK^+^ level 5.5–5.9 mEq/L), moderate (sK^+^ level 6.0–6.4 mEq/L) or severe (sK^+^ level ≥6.5 mEq/L) [5]. While these thresholds provide a framework for classification, the clinical relevance of elevated sK^+^ levels depends on the broader context, including weather the sample was obtained during routine monitoring or in response to clinical symptoms [6–8].

Hyperkalaemia often occurs in patients with acute kidney injury or chronic kidney disease (CKD) due to several factors. These include impaired renal excretion of K^+^ due to low estimated glomerular filtration rate (eGFR), shift of intracellular K^+^ to the extracellular space and in conjunction with a maintained dietary K^+^ intake [3]. Diabetes mellitus (DM) and cardiovascular disease (CVD) [including heart failure (HF)] are also known risk factors for hyperkalaemia [9]. Additionally, patients with CKD and CVD often receive treatments that affect K^+^ excretion, potentially leading to hyperkalaemia. These treatments include renin–angiotensin–aldosterone system inhibitors (RAASi) such as angiotensin-converting enzyme inhibitors (ACEi), angiotensin-receptor blockers (ARBs) and, increasingly, mineralocorticoid receptor antagonists (MRAs) [10–12].

Traditionally, long-term management of hyperkalaemia has focused on prevention through a low dietary K^+^ intake, adequate diuretic therapy and addressing metabolic acidosis [13, 14]. Discontinuation or dose-modification of medications that may precipitate hyperkalaemia, such as RAASi might be needed [15]. However, sub-optimal RAASi dosing or halting RAASi in patients with CKD can lead to increased risk of cardiovascular events [hazard ratio (HR) 1.45] and all-cause death (HR 2.26) and even worse outcomes in populations with HF and DM [16, 17]. Mortality rates of patients on sub-optimal RAASi doses are nearly double compared with those on maximum RAASi doses [18]. Recently, contemporary potassium-lowering agents have shown a favourable safety profile and can reduce the risk of hyperkalaemia, allowing patients to continue RAASi treatment [13, 14]. Guidelines for managing diabetes (2020) and blood pressure (2021) in CKD recommend treating hyperkalaemia with K^+^-lowering therapies before RAASi down-titration [19].

A recent (2022) published systematic literature review (SLR) and meta-analysis sought to provide a comprehensive overview of the epidemiology of hyperkalaemia across different populations, definitions, healthcare settings, diseases and pharmacotherapies, given this lack of extensive current literature [3]. As new CKD pharmacotherapies emerge, and with increasing observation of multimorbid patients, our SLR aimed to provide a contemporary assessment specifically within adult CKD patients, due to their increased risk of hyperkalaemia and related events. While hyperkalaemia has been linked to poorer outcomes, it remains unclear whether these outcomes are directly due to hyperkalaemia itself, related comorbid conditions such as DM, or consequent sub-optimal RAASi dosing. Findings from this SLR in CKD patients were therefore qualitatively summarized to (i) describe the risk factors and global epidemiology of hyperkalaemia; (ii) identify the clinical, humanistic and economic burden of hyperkalaemia; and (iii) describe how RAASi use is associated with hyperkalaemia, the consequent impact of hyperkalaemia on RAASi use, and examine the sub-optimal dosing effect of RAASi on clinical and economic outcomes. The findings of this SLR are intended to provide healthcare practitioners with evidence-based insights for managing pharmacotherapy in CKD patients with hyperkalaemia, thereby enhancing clinical decision-making.

## MATERIALS AND METHODS

The SLR was conducted according to the general recommendations of the Cochrane Handbook for Systematic Reviews of Interventions [20], the general principles of the Centre for Reviews and Dissemination (CRD, University of York) guidance [21] for undertaking reviews in health care, the Preferred Reporting Items for Systematic Reviews and Meta-Analyses (PRISMA) guidelines [22] and the methods for systematic reviews as specified by National Institute for Health and Care Excellence (NICE) [23]. The SLR was registered with the International Prospective Register of Systematic Reviews (PROSPERO; www.crd.york.ac.uk/prospero/display_record.php?ID=CRD42023440088). To gather evidence reflecting the current practice standards, searches in Medline^®^ and Embase^®^ were conducted from January 2000 to April 2024 and to identify relevant studies not available through electronic databases, abstracts from relevant conferences published from January 2021 through April 2024 were hand-searched. For further information see the supplementary materials (Supplementary data, Tables S1–S9).

The SLR eligibility criteria, determined using the PICOS (population, intervention, comparator, outcomes and study design) framework [20], is outlined in Table 1.

**Table 1:** SLR eligibility criteria.

PICOS	Inclusion criteria	Exclusion criteria
Population	• Adults with CKD + hyperkalaemia versus CKD + normokalaemia/hypokalaemia^a^	• Patients with indications other than CKD
	• Adults with CKD + chronic HF + hyperkalaemia versus CKD normokalaemia/hypokalaemia^a^	• Patients with kidney damage due to trauma
	• Adults with CKD + HT + hyperkalaemia versus CKD + normokalaemia/hypokalaemia^a^	• Children and paediatric populations
	• Adults with CKD + DM + hyperkalaemia versus CKD + normokalaemia/hypokalaemia^a^	• Animal studies
	• Aged 18 years of age	
Intervention/comparator	Not applicable	No applicable
Outcomes	Epidemiology:	Any outcomes not listed in the inclusion criteria
	• Prevalence, incidence, epidemiology trends like risk factors (kidney failure, DM, adrenal disease), patient population size	
	Clinical burden:	
	• Time to ESRD, time to acute kidney injury, time to CV death, time to renal death, time to all-cause mortality, time to non-fatal MI, time to non-fatal stroke	
	• Hospitalization rate, length of stay, requirement of dialysis	
	Financial burden:	
	• Direct costs (cost of dialysis, hospitalization cost, treatment cost, medical costs, etc.)	
	• Indirect costs (cost of caregiver cost, productivity loss etc.)	
	Humanistic burden:	
	• HRQoL (SF-36, EQ-5D, KDQOL, FACIT-F), patient-reported outcomes and caregiver outcomes	
	Sub-optimal dosing:	
	• Prevalence and incidence of RAASi-associated hyperkalaemia	
	• Proportion of patients with RAASi discontinuation	
	• Proportion of patients with RAASi sub-optimal dosing/dose modification	
	• Impact of RAASi sub-optimal dosing/dose modification on clinical/economic or humanistic outcome	
Study design(s)	• RCTs, nRCTs and single arm trials	• Case reports
	• Prospective and retrospective observational studies	• Case studies
	• Cross-sectional and case–control study	• Editorials, notes, comments
	• Cost of illness studies	
Limits	• Timeframe: full-texts 2000 to current; conference abstracts 2021, 2022, 2023 and 2024	• Exclude publications prior to 2000 and conference abstracts prior to 2021
	• Geography: no restrictions: the regional differences will be reported where data is available	• Non-English literature
	• Language: English only^b^	

a The control group was relevant only for epidemiology and burden of illness review.

b The majority of abstracts/full text were in English.

CV, cardiovascular; EQ-5D, EuroQol 5 dimensions; FACIT-F, Functional assessment of chronic illness therapy—fatigue; HRQoL, health-related quality of life; HT, hypertension; KDQOL, Kidney disease and quality of life; MI, myocardial infarction; nRCTs, non-randomized controlled trials; SF-36, Short form-36.

### Study selection

Titles and abstracts were independently reviewed by two researchers against the pre-defined eligibility criteria using the PICOS framework. Publications selected as potentially relevant from abstract review were retained and full text publications were independently assessed by two reviewers, with discrepancies resolved by consulting a third reviewer and on reaching consensus.

### Data extraction and study quality assessment

Data extraction from the full text studies identified by the searches was conducted by one researcher and quality checked by another independent researcher. The methodological quality of the randomized controlled trials (RCTs) was assessed using the Cochrane Collaboration tool for assessing the risk of bias [24]. The Downs and Black checklist [25] was used to assess the risk of bias in non-randomized/observational studies. The complete list of extracted data (Supplementary data, Table S10) and the quality assessment checklist, along with the results (Supplementary data, Table S11, Figs S9 and S10), are detailed in the supplementary materials.

## RESULTS

A total of 138 studies, described in 144 publications [16, 26–146, 147–168] met the PICOS criteria. The PRISMA flow diagrams, per research question are presented in Supplementary data, Figs S1 and S2 of the supplementary materials; a full list of the included studies is presented in Supplementary data, Tables S13 and S14 of the supplementary materials.

Most of the studies (*n* = 87) were retrospective observational, followed by 33 RCTs. Most of these studies were based in the USA (*n* = 35) or were multinational (*n* = 17). Across the studies, 38 different definitions or thresholds of hyperkalaemia were applied, with some studies using more than one. The most frequently employed criteria were sK^+^ >5.0 mEq/L in 27 studies, followed by sK^+^ >5.5 mEq/L in 23 studies and sK^+^ ≥5.5 mEq/L in 22 studies. Among the studies that provided information on CKD stages, there was significant variation in the distribution of disease severity (Supplementary data, Table S14). All extracted results are tabulated in the supplementary materials (Supplementary data, Tables S15–S25).

### Risk factors for hyperkalaemia

The studies included in this review identified CKD stage and eGFR category as the most significant risk factors for hyperkalaemia in CKD patients, along with comorbidities such as DM and HF. Additionally, this SLR highlighted that one of the most critical risk factors of hyperkalaemia in CKD patients was the prescription of RAASi [29], including MRAs. For example, the HR by multivariate analysis was 7.00 for ACEi vs calcium channel blocker use [95% confidence interval (CI) 2.29–21.39; *P* < .001], 2.85 for ACEi vs beta-blocker (β-blocker) use (95% CI 1.5–5.42; *P =* .001) [63] and 7.71 for aliskiren use (95% CI 1.14–52.3; *P* = .04) [79].

### Epidemiology of hyperkalaemia

A total of 33 studies [26–29, 31–35, 38, 41, 42, 44–56, 139–146] were reviewed to determine the prevalence of hyperkalaemia and hypokalaemia/normokalaemia among CKD patients. Prevalence differed depending on the hyperkalaemia definition used. The prevalence rate of the most commonly used threshold of hyperkalaemia (sK^+^ >5.0 mEq/L) varied significantly between studies due to the differing stages of disease and multimorbid patients, ranging from 0.8% in a subgroup of patients with stage 1 CKD not on renal replacement therapy (*N* = 118 patients) in Taiwan between November 2002 and July 2010 [46], to 56.0% in a subgroup of patients with stage 5 CKD (*N* = 421 patients) in Mexico between February 2019 and August 2022 [146]. In addition, the overall prevalence of hyperkalaemia varied considerably by region, with studies from the USA reporting rates ranging from 1.1% [44] to 20.1% [48], China from 3.8% [29] to 44.4% [26], and Japan showing higher rates ranging from 10.6% [56] to 57.6% [31]. Studies form the European region, including the UK, Italy, Spain and France, reported CKD prevalence estimates ranging from 0.7% [41] to 33.6% [141]. Additional data from Australia [53], Taiwan [46, 52], Thailand [140], Turkey [142], Mexico [146] and Pakistan [144] further highlighted the geographic variation, likely reflecting differences in CKD stage distribution, comorbidity profiles, and hyperkalaemia definitions across studies (Supplementary data, Table S16).

Further, in the nine studies [31, 34, 46, 50, 53, 56, 140, 141, 146] reporting prevalence of hyperkalaemia among different stages of CKD, the overall prevalence of hyperkalaemia was observed to increase with declining kidney function [31].

Seven additional studies [16, 61, 66, 105, 128, 131, 141] have reported the increasing prevalence of RAASi associated hyperkalaemia in CKD patients. For example, a Spanish study of patients with stage 3–5 CKD (non-dialyzed), DM and/or HF between 1971 and 2017, reported the prevalence rate of hyperkalaemia for patients taking RAASi and for patients not taking RAASi. In the analysis of patients with different CKD stages and comorbidities such as DM and HF, the prevalence rate was higher among CKD patients taking RAASi vs patients not taking RAASi [128].

The prevalence rates of hyperkalaemia in CKD patients were found to vary for those on ARBs vs ACEi, with one US study of stage 1–5 CKD patients reporting higher hyperkalaemia prevalence among patients taking ARBs (31% vs 20.4%, for ARBs vs ACEi) [61] and a Danish study reporting higher prevalence in CKD patients taking ACEi (17.2% vs 34.3%, for ARBs vs ACEi) [66]; both studies defined hyperkalaemia at sK^+^ >5.0 mEq/L.

Two studies [47, 55] have reported the incidence of hyperkalaemia and hypokalaemia/normokalaemia among CKD patients. In a US study with moderate and advanced non-dialysis-dependent CKD (*N* = 1227 patients, 86% stage 3 or 4), the incidence rate of hyperkalaemia (sK^+^ >5.3 mEq/L) was 21.7 cases per 1000 person-years [vs 9.7 cases of hypokalaemia (defined as sK^+^ <3.6 mEq/L) per 1000 person-years] [47]. A higher incidence was reported in a Spanish study of patients with CKD (*N* = 1129 patients): 41.9 cases per 1000 person-years of hyperkalaemia (defined as sK^+^ >5.0 mEq/L) [vs 18.9 cases of hypokalaemia (sK^+^ <3.5 mEq/L) per 1000 person-years] [55].

An additional 69 studies [57, 59, 60, 62–65, 67–70, 72–74, 77–86, 88, 90, 91, 93, 94, 101, 102, 104–116, 118–121, 123, 125–127, 129, 130, 132, 134, 135, 150–153, 155, 158, 160–163, 165, 168] reported the incidence of RAASi-associated hyperkalaemia in CKD patients. Supplementary data, Figs S3 and S4 show the trend of increased hyperkalaemia incidence and incidence rate with declining kidney function, as well as the increased hyperkalaemia incidence with RAASi use. Notably, a trend of increased incidence of hyperkalaemia with MRA use was reported in 15 studies (from 19 publications [70, 80, 83, 85, 88, 93–95, 97–100, 107, 114, 118, 119, 129, 132, 153], all of which compared patients on an MRA with those on placebo/non-MRA).

### Burden of hyperkalaemia

Twelve studies [26, 28, 29, 31, 34, 38, 40, 42, 44, 55, 71, 147] reported the health outcomes of hyperkalaemia, focusing on hospitalization numbers, rates and length of stay. Compared with CKD patients with normokalaemia, those with hyperkalaemia (including stage 1–5 CKD and CKD concomitant with end-stage renal disease (ESRD) or HF] had an average number of 0.8–1.9 vs 0.3–1.4 hospitalizations during 1 year of follow-up period [29, 34, 42, 147]. Four studies [26, 28, 29, 34] also noted that CKD patients with hyperkalaemia had longer hospital stays compared with those without hyperkalaemia.

Nine studies [29, 30, 34, 36, 37, 42, 43, 71, 147] estimated the economic burden of hyperkalaemia, all focusing on direct healthcare costs. The total healthcare costs (pharmacy and medical) per patient per year in a US study were estimated 1-year post-index date, revealing that hyperkalaemic patients (including at least two sK^+^ readings >5.0 mEq/L on different dates) used more healthcare resources and therefore were considerably more costly (ranging from $10 687 to $67 758) than normokalaemic patients (sK^+^ 3.8–5.0 mEq/L) (ranging from $6417 to $58 969), regardless of the CKD stage (stage 1 to 5; stage 5 with and without dialysis) [30]. Similar results of substantial costs associated with hyperkalaemia were reported consistently in four other studies in an overall CKD population analysis [37, 43, 71, 147].

Hyperkalaemia can become a medical emergency requiring hospitalization, increasing the economic burden [169]. Addressing this specifically, the total all-cause healthcare costs (medical and pharmacy) during 1 year post-discharge in a US study were almost twice as high in CKD patients with hospitalizations related to hyperkalaemia (sK^+^ >5.0 mEq/L) compared with CKD patients with hospitalizations but without evidence of hyperkalaemia (sK^+^ <5.0 mEq/L) [36]. These findings were further corroborated by two detailed analysis in China and Japan [34, 46].

Patients with CKD who develop hyperkalaemia due to RAASi treatment may need to reduce their dosage (sub-optimal dosing) or stop the treatment altogether. An additional 16 studies [58, 69, 76, 77, 84, 95, 103, 114, 121, 125, 127, 133, 142, 149, 154, 157] on CKD patients with sub-optimal RAASi dosing due to hyperkalaemia and 44 studies [16, 57, 58, 60, 70, 75–80, 84–88, 92–94, 103, 107, 111, 113, 114, 116–120, 122, 124, 125, 127, 129, 130, 133, 142, 149, 155, 157, 159, 165, 166, 167] on those who discontinued RAASi for the same reason were identified. A trend of down-titration was observed across these studies, with a UK study showing that the highest proportions of sub-optimal dosing and discontinuation were among patients on higher RAASi doses compared with lower doses, across three hyperkalaemia thresholds (sK^+^ 5 mEq/L, sK^+^ 5.5 mEq/L, sK^+^ 6 mEq/L) [103].

Despite the association between RAASi and hyperkalaemia, this does not always result in more frequent dose reductions in patients with CKD. An Australian study of patients with stage 3–5 CKD (*N* = 20 184 patients) after 3.9 years of follow-up reported the proportion of patients with sub-optimal RAASi dosing decreasing from stage 3a (10.7%) to stage 5 (5.4%), despite an increased incidence of hyperkalaemia in the later stages of the disease [127].

RAASi discontinuation compared with RAASi continuation was associated with a greater absolute 5-year risk of mortality (54.5% vs 40.9%) and major adverse cardiovascular events (59.5% vs 47.6%) in patients with advanced CKD (eGFR <30 mL/min per 1.73^2^) [137], and lower mean eGFR (12.6 vs 13.3 mL/min/1.73 m^2^) [136]. These risks were observed greatest amongst the most advanced CKD patients investigated [137]. The mean number of inpatient visits was generally higher amongst patients with sub-optimal dosing as compared with optimal dosing [71]. Finally, the median medical costs for patients with CKD and a diagnosis of hyperkalaemia in the USA were significantly higher for patients with optimal RAASi dosing as compared with sub-optimal dosing ($12 671 vs $9065, respectively) [71]. However, for the subgroup of patients with CKD and HF and a diagnosis of hyperkalaemia who received RAASi at an optimal dose, the overall median medical cost were lower than the same patients who received sub-optimal dosing ($27 075 vs $28 293) [71]. See Supplementary data, Figs S5 to S8 for graphical visualizations of sub-optimal RAASi dosing findings.

## DISCUSSION

This SLR highlights the complex relationship between the presence of hyperkalaemia and outcomes. Findings, including the most contemporary literature, confirmed that the prevalence of hyperkalaemia in CKD depends on the definition of the condition, i.e. the sK^+^ threshold applied, as previously described [3, 170]. The data collected suggest that the prevalence of hyperkalaemia increases with declining kidney function, in agreement with hyperkalaemia being a well-established consequence of advancing CKD [170]. These findings closely reflect the prevalence findings described previously [171]. Similarly, as expected [45, 170, 171], other underlying conditions such as DM and HF contributed to higher sK^+^ concentration.

RAASi medications for CKD are among the key determinants of elevated sK^+^ concentration [170, 171], and hyperkalaemia has previously been proven prevalent in patients treated with these therapies [3]. Supporting this, the SLR identified evidence of increased prevalence and incidence of hyperkalaemia among patients with CKD treated with RAASi, including ACEi, ARBs and MRAs. Despite this observation, all these agents have previously been shown to reduce the incidence of endpoints important to stakeholders [172].

The results of this SLR show that CKD patients with hyperkalaemia, with or without comorbidities, experience higher absolute numbers and hospitalization rates, as well as longer hospitalization periods compared with CKD patients with normokalaemia. It should be noted that not all studies reported the cause of hospitalization or whether it was directly due to hyperkalaemia; when specified, it included kidney function/cardiac diagnosis or it was stated as related to hyperkalaemia. In the presence of other comorbidities, CKD patients with hyperkalaemia are likely to have worse outcomes as previously suggested [173], although in the present analysis few studies focused on hyperkalaemic patients with CKD and relevant comorbidities. Evidence describing multimorbid populations would be more relevant to the populations treated in clinical practice.

It has been reported in the literature that the annual mortality rate for patients with CKD and hyperkalaemia was more than double compared with patients with CKD without hyperkalaemia [174]. Other concordant studies strongly associated hyperkalaemia and increased mortality [170]. The present SLR observed similar findings, with generally worse health outcomes and an increased clinical burden among hyperkalaemic CKD patients. However, no published study could firmly resolve the question whether worse outcomes directly relate to hyperkalaemia or worse underlying or associated medical conditions, thus all studies remain at the level of an association with no evidence for causality.

From this analysis, it was also concluded that hyperkalaemia is associated with substantial additional healthcare expenditure, including medical and pharmacy expenses, both for the patient and healthcare systems globally. Inpatient care during hyperkalaemia-related hospitalization was a main driver of these costs among patients with hyperkalaemia and concomitant CKD or/and another comorbidity (e.g. HF). These results are consistent with previous reviews evaluating hyperkalaemia-related costs [175]. Regarding humanistic burden elements, only a single study was eligible for inclusion in this SLR and further research to evidence any quality-of-life burden here is needed.

Among the current strategies to improve sK^+^ concentration and its observed burden are dose reductions or halting of RAASi treatment. Sub-optimal dosing and treatment discontinuation was observed at higher rates in patients receiving high doses of RAASi [103, 113], however the percentage varied by CKD stage [77, 127]. The finding suggests the adverse outcomes from hyperkalaemia and the adverse outcomes from sub-optimal dosing both place CKD patients at higher risk of negative health outcomes. Furthermore, increasing hyperkalaemia incidence does not always lead to sub-optimal RAASi dosing [127, 142]. Whilst it is highly likely that the increasing incidence of hyperkalaemia is caused by the increasing severity of CKD, and thus why RAASi use increases, it might be assumed that the risk of adverse clinical outcomes from RAASi sub-optimal dosing in those with severe CKD outweighs the dangers of hyperkalaemia in these patients. Our SLR findings on the clinical impact of sub-optimal dosing support this observation. It is imperative that the impact and balance between the risks are explored in further research.

Consequences of sub-optimal RAASi dosing or discontinuation in CKD patients often leads to adverse clinical outcomes due to the increased risk of cardiorenal events [16, 58, 71, 73, 103, 121, 136–138, 149, 156, 157, 164]. However, in one study, sub-optimal RAASi dosing in patients with CKD and hyperkalaemia was associated with better clinical and economic outcomes compared with optimal dosing in the same patients [71]. The latter observation is likely due to the occurrence of hyperkalaemic events when RAASi therapies are prescribed at their maximum recommended doses, which have a high clinical and economic burden as already identified in this SLR and by previous research, as well as patients on optimal RAASi doses likely having comorbid conditions that are costly to manage. Moreover, the study also found that sub-optimal RAASi dosing in patients with CKD and hyperkalaemia and comorbid HF was associated with worse economic outcomes when compared with optimal dosing, confirming that there is a high cost of comorbid conditions like HF when RAASi is not used optimally. This highlights the importance of sK^+^ monitoring amongst CKD patients treated with RAASi, and the much-needed research into alternative approaches for hyperkalaemia management in CKD, particularly at advanced stages of disease.

## LIMITATIONS

The included studies varied greatly in terms of patient populations, settings, study design, sample size and hyperkalaemia definitions used; therefore, results are not always comparable across the studies. Moreover, most studies reporting the epidemiology of RAASi associated hyperkalaemia in CKD patients also reported rates for varying subgroups of CKD patients, differing by CKD stages and comorbidities. As these are also risk factors for hyperkalaemia in CKD patients, the differing epidemiology of hyperkalaemia attributed to RAASi between studies in this SLR should be interpreted with caution.

It is worth acknowledging that hyperkalaemia itself may be an indicator of poor health rather than the cause of adverse outcomes observed in this review; this should be investigated in future research. Moreover, therapeutic developments for hyperkalaemia since 2015 may have influenced the epidemiology of hyperkalaemia, although the impact of these advances remains unclear and warrants further research.

Finally, heterogeneity in reported hyperkalaemia prevalence may reflect geographic and healthcare system-related factors, such as differences in diet and cuisine, access to dietary counselling or dietetics services, availability, and affordability of medications associated with hyperkalaemia, and healthcare delivery models (e.g. public vs insurance-based systems). Finally, the restriction of the search to English-language publications may have further limited the ability to capture such regional differences in hyperkalaemia epidemiology.

Further studies are needed to explore the impact of these factors, assess the role of novel therapies and better understand the causal relationship between hyperkalaemia and clinical outcomes.

## CONCLUSIONS

This SLR including the most contemporary literature highlights evidence that hyperkalaemia, with increasing prevalence in advancing CKD, is associated with adverse health outcomes and the additional use of hospital resources. Therefore, measures that could maintain sK^+^ concentration within the normal range may decrease the prevalence/incidence and hence the total burden of hyperkalaemia. However, some measures that lower sK^+^ in response to hyperkalaemia by modifying medications dose will have potential detrimental consequences in CKD outcomes, particularly in patients with comorbid conditions like HF. This emphasizes that the treatments currently available for CKD patients with or at risk of chronic hyperkalaemia are still limited, presenting a specific unmet need, and careful consideration by clinicians is required when managing these multimorbid patients [171, 174].

## Supplementary Material

sfaf127_Supplemental_Files

## Data Availability

The data underlying this article are available in the article and in its online supplementary material.

## References

[1] Palmer BF, Carrero JJ, Clegg DJ et al. Clinical management of hyperkalemia. Mayo Clin Proc 2021;96:744–62. 10.1016/j.mayocp.2020.06.014 33160639

[2] Rosano GM, Tamargo J, Kjeldsen KP et al. Expert consensus document on the management of hyperkalaemia in patients with cardiovascular disease treated with renin angiotensin aldosterone system inhibitors: coordinated by the Working Group on Cardiovascular Pharmacotherapy of the European Society of Cardiology. Eur Heart J Cardiovasc Pharmacother 2018;4:180–8. 29726985 10.1093/ehjcvp/pvy015 PMC13588647

[3] Humphrey T, Davids MR, Chothia M-Y et al. How common is hyperkalaemia? A systematic review and meta-analysis of the prevalence and incidence of hyperkalaemia reported in observational studies. Clin Kidney J 2022;15:727–37. 10.1093/ckj/sfab243 35371465 PMC8967676

[4] Ferreira JP, Zannad F, Butler J et al. Empagliflozin and serum potassium in heart failure: an analysis from EMPEROR-Pooled. Eur Heart J 2022;43:2984–93. 10.1093/eurheartj/ehac306 35687107 PMC9375711

[5] Lott C, Truhlář A, Alfonzo A et al. European Resuscitation Council Guidelines: cardiac arrest in special circumstances. Resuscitation 2021;161:152–219. 33773826 10.1016/j.resuscitation.2021.02.011

[6] Lehnhardt A, Kemper MJ. Pathogenesis, diagnosis and management of hyperkalemia. Pediatr Nephrol 2011;26:377–84. 10.1007/s00467-010-1699-3 21181208 PMC3061004

[7] Elliott MJ, Ronksley PE, Clase CM et al. Management of patients with acute hyperkalemia. Can Med Assoc J 2010;182:1631–5. 10.1503/cmaj.100461 20855477 PMC2952010

[8] Clase CM, Carrero J-J, Ellison DH et al. Potassium homeostasis and management of dyskalemia in kidney diseases: conclusions from a Kidney Disease: Improving Global Outcomes (KDIGO) Controversies Conference. Kidney Int 2020;97:42–61. 10.1016/j.kint.2019.09.018 31706619

[9] Watanabe R. Hyperkalemia in chronic kidney disease. Rev Assoc Med Bras 2020;66:s31–6. 10.1590/1806-9282.66.s1.31 31939533

[10] Douglas M, Rizzolo D, Kruger D. Hyperkalemia in adults: review of a common electrolyte imbalance. Clin Rev 2017;27:40–9.

[11] Rowan C, Kovesdy C, Du Mond C et al. Comparison of potassium values before and after patiromer initiation among patients receiving chronic hemodialysis in the United States. Nephrol Dial Transplant 2017;32(Suppl 3):iii692–3.

[12] Sarafidis PA, Blacklock R, Wood E et al. Prevalence and factors associated with hyperkalemia in predialysis patients followed in a low-clearance clinic. Clin J Am Soc Nephrol 2012;7:1234–41. 10.2215/CJN.01150112 22595825 PMC3408123

[13] Bjune T, Bøe TB, Kjellevold SA et al. Hyperkalemia and the use of new potassium binders a single center experience from Vestfold Norway (the PotBind study). Int J Nephrol Renovasc Dis 2023;16:73–82. 36960344 10.2147/IJNRD.S401623 PMC10027611

[14] Bianchi S, Regolisti G. Pivotal clinical trials, meta-analyses and current guidelines in the treatment of hyperkalemia. Nephrol Dial Transplant 2019;34:iii51–61. 10.1093/ndt/gfz213 31800075

[15] Kovesdy CP. Management of hyperkalaemia in chronic kidney disease. Nat Rev Nephrol 2014;10:653–62. 10.1038/nrneph.2014.168 25223988

[16] Santoro A, Perrone V, Giacomini E et al. Association between hyperkalemia, RAASi non-adherence and outcomes in chronic kidney disease. J Nephrol 2022;35:463–72. 10.1007/s40620-021-01070-6 34115311 PMC8927011

[17] Evans M, Palaka E, Furuland H et al. The value of maintaining normokalaemia and enabling RAASi therapy in chronic kidney disease. BMC Nephrol 2019;20:1–11. 10.1186/s12882-019-1228-y 30704421 PMC6357372

[18] Kuijvenhoven MA, Haak EA, Gombert-Handoko KB et al. Evaluation of the concurrent use of potassium-influencing drugs as risk factors for the development of hyperkalemia. Int J Clin Pharm 2013;35:1099–104. 10.1007/s11096-013-9830-8 23974985

[19] Burton JO, Coats AJ, Kovesdy CP et al. An international Delphi consensus regarding best practice recommendations for hyperkalaemia across the cardiorenal spectrum. Eur J Heart Fail 2022;24:1467–77. 10.1002/ejhf.2612 35791065 PMC9804940

[20] Higgins JP, Thomas J, Chandler J et al. Cochrane Handbook for Systematic Reviews of Interventions. Chichester, England: John Wiley & Sons Ltd., 2019. 10.1002/9781119536604

[21] Centre for Reviews and Dissemination (CRD) . Guidance for Undertaking Reviews in Health Care. Systematic Reviews CRD. Layerthorpe, York: University of York, 2008. https://www.york.ac.uk/media/crd/Systematic_Reviews.pdf (27 April 2023, date last accessed).

[22] Page MJ, McKenzie JE, Bossuyt PM et al. The PRISMA 2020 statement: an updated guideline for reporting systematic reviews. Int J Surg 2021;88:105906. 10.1016/j.ijsu.2021.105906 33789826

[23] National Institute for Health and Care Excellence (NICE) . Single Technology Appraisal: User Guide for Company Evidence Submission Template. London, UK: The National Institute for Health and Care Excellence (NICE), 2015. https://www.nice.org.uk/process/pmg24/chapter/clinical-effectiveness#quality-assessment-of-the-relevant-clinical-effectiveness-evidence (27 April 2023, date last accessed).

[24] Higgins JPT, Altman DG, Gøtzsche PC et al. The Cochrane Collaboration's tool for assessing risk of bias in randomised trials. BMJ 2011;343:d5928. 10.1136/bmj.d5928 22008217 PMC3196245

[25] Downs SH, Black N. The feasibility of creating a checklist for the assessment of the methodological quality both of randomised and non-randomised studies of health care interventions. J Epidemiol Commun Health 1998;52:377–84. 10.1136/jech.52.6.377 PMC1756728 9764259

[26] Li X, Li B, Guo Y. Impact of hyperkalemia on hospitalization days in advanced chronic kidney disease patients with Type-2 diabetes mellitus: a prospective study. Pakistan J Med Sci 2023;39:885–90. 10.12669/pjms.39.3.6874 PMC10214826 37250542

[27] Wang J, Wang F. WCN23-1101 Instantaneous and persistent elevation of serum potassium and progression of chronic kidney disease, a single center-based cohort study. Kidney Int Rep 2023;8:S159. 10.1016/j.ekir.2023.02.358

[28] Calabrese V, Cernaro V, Battaglia V et al. Correlation between hyperkalemia and the duration of several hospitalizations in patients with chronic kidney disease. J Clin Med 2022;11:244. 10.3390/jcm11010244 35011985 PMC8746076

[29] Zhang J, He X, Wu J. the impact of hyperkalemia on mortality and healthcare resource utilization among patients with chronic kidney disease: a matched cohort study in China. Front Public Health 2022;10:855395. 10.3389/fpubh.2022.855395 35400057 PMC8987151

[30] Sharma A, Alvarez PJ, Woods SD et al. Healthcare resource utilization and costs associated with hyperkalemia in a large managed care population. J Pharm Health Serv Res 2021;12:35–41. 10.1093/jphsr/rmaa004

[31] Kohsaka S, Okami S, Kanda E et al. Cardiovascular and renal outcomes associated with hyperkalemia in chronic kidney disease: a hospital-based cohort study. Mayo Clinic Proc 2021;5:274–85. 10.1016/j.mayocpiqo.2020.10.001 PMC8105529 33997627

[32] Grandy S, Jackson J, Moon R et al. Health-related quality of life and lifestyle changes in patients with chronic kidney disease and hyperkalaemia: real-world data from the US, five European countries and China. Int J Clin Pract 2021;75:e14326. 10.1111/ijcp.14326 33960068

[33] Sharma A, Alvarez PJ, Woods SD et al. A model to predict risk of hyperkalemia in patients with chronic kidney disease using a large administrative claims database. Clinicoecon Outcomes Res 2020;12:657–67. 10.2147/CEOR.S267063 33204127 PMC7665575

[34] Kanda E, Kashihara N, Kohsaka S et al. Clinical and economic burden of hyperkalemia: a nationwide hospital-based cohort study in Japan. Kidney Medicine 2020;2:742–52.e1. 10.1016/j.xkme.2020.09.003 33319198 PMC7729225

[35] Jimenez-Marrero S, Cainzos-Achirica M, Monterde D et al. Real-world epidemiology of potassium derangements among chronic cardiovascular, metabolic and renal conditions: a population-based analysis. Clin Epidemiol 2020;12:941–52. 10.2147/CLEP.S253745 32982459 PMC7494006

[36] Betts KA, Woolley JM, Mu F et al. Postdischarge health care costs and readmission in patients with hyperkalemia-related hospitalizations. Kidney Int Rep 2020;5:1280–90. 10.1016/j.ekir.2020.06.004 32775827 PMC7403556

[37] Mu F, Betts KA, Woolley JM et al. Prevalence and economic burden of hyperkalemia in the United States Medicare population. Curr Med Res Opin 2020;36:1333–41. 10.1080/03007995.2020.1775072 32459116

[38] James G, Carrero JJ, Kumar S et al. Pos-328 The burden of hyperkalemia in patients with chronic kidney disease: a report from the DISCOVER CKD retrospective cohort. Kidney Int Rep 2021;6:S141–2. 10.1016/j.ekir.2021.03.344

[39] James G, Carrero JJ, Khezrian M et al. Pos-305 Hospitalizations and length of stay in patients with CKD with and without hyperkalemia: a report from the DISCOVER CKD retrospective cohort. Kidney Int Rep 2022;7:S136–7. 10.1016/j.ekir.2022.01.325

[40] Thomsen RW, Nicolaisen SK, Hasvold P et al. Elevated potassium levels in patients with chronic kidney disease: occurrence, risk factors and clinical outcomes-a Danish population-based cohort study. Nephrol Dial Transplant 2018;33:1610–20. 10.1093/ndt/gfx312 29177463

[41] Furuland H, McEwan P, Evans M et al. Serum potassium as a predictor of adverse clinical outcomes in patients with chronic kidney disease: New risk equations using the UK clinical practice research datalink. BMC Nephrol 2018;19:211. 10.1186/s12882-018-1007-1 30134846 PMC6106824

[42] Neuenschwander JF, Silverstein AR, Teigland CL et al. The increased clinical and economic burden of hyperkalemia in Medicare patients admitted to long-term care settings. Adv Ther 2023;40:1204–23. 10.1007/s12325-022-02420-x 36652174 PMC9988794

[43] Betts KA, Woolley JM, Mu F et al. The cost of hyperkalemia in the United States. Kidney Int Rep 2018;3:385–93. 10.1016/j.ekir.2017.11.003 29725642 PMC5932129

[44] Luo J, Brunelli SM, Jensen DE et al. Association between serum potassium and outcomes in patients with reduced kidney function. Clin J Am Soc Nephrol 2016;11:90–100. 10.2215/CJN.01730215 26500246 PMC4702219

[45] Nakhoul GN, Huang H, Arrigain S et al. Serum potassium, end-stage renal disease and mortality in chronic kidney disease. Am J Nephrol 2015;41:456–63. 10.1159/000437151 26228532 PMC4686260

[46] Wang HH, Hung CC, Hwang DY et al. Hypokalemia, its contributing factors and renal outcomes in patients with chronic kidney disease. PLoS One 2013;8:e67140. 10.1371/journal.pone.0067140 23843989 PMC3699540

[47] Hayes J, Kalantar-Zadeh K, Lu JL et al. Association of hypo- and hyperkalemia with disease progression and mortality in males with chronic kidney disease: the role of race. Nephron Clin Pract 2012;120:C8–16. 22156587 10.1159/000329511 PMC3267990

[48] Jain N, Kotla S, Little BB et al. Predictors of hyperkalemia and death in patients with cardiac and renal disease. Am J Cardiol 2012;109:1510–3. 10.1016/j.amjcard.2012.01.367 22342847

[49] Wagner S, Metzger M, Flamant M et al. Association of plasma potassium with mortality and end-stage kidney disease in patients with chronic kidney disease under nephrologist care—The NephroTest study. BMC Nephrol 2017;18:295. 10.1186/s12882-017-0710-7 28899351 PMC5596852

[50] Collins AJ, Pitt B, Reaven N et al. Association of serum potassium with all-cause mortality in patients with and without heart failure, chronic kidney disease, and/or diabetes. Am J Nephrol 2017;46:213–21. 10.1159/000479802 28866674 PMC5637309

[51] Korgaonkar S, Tilea A, Gillespie BW et al. Serum potassium and outcomes in CKD: insights from the RRI-CKD cohort study. Clin J Am Soc Nephrol 2010;5:762–9. 10.2215/CJN.05850809 20203167 PMC2863985

[52] Hwang J-C, Wang C-T, Chen C-A et al. Hypokalemia is associated with increased mortality rate in chronic hemodialysis patients. Blood Purif 2011;32:254–61. 10.1159/000325226 21849775

[53] Brookes EM, Snider J, Hart GK et al. Serum potassium abnormalities in chronic kidney disease: prevalence, patient characteristics and clinical outcomes. Intern Med J 2021;51:1906–18. 10.1111/imj.14970 33314585

[54] Shang G, Gao Y, Liu K et al. Serum potassium in elderly heart failure patients as a predictor of readmission within 1 year. Heart Vessels 2022;38:507–16. 36318301 10.1007/s00380-022-02192-y

[55] Jimenez-Marrero S, Cainzos-Achirica M, Monterde D et al. Impact on clinical outcomes and health costs of deranged potassium levels in patients with chronic cardiovascular, metabolic, and renal conditions. Rev Esp Cardiol 2021;74:312–20. 32694080 10.1016/j.rec.2020.06.013

[56] Tanaka K, Saito H, Iwasaki T et al. Association between serum potassium levels and adverse outcomes in chronic kidney disease: the Fukushima CKD cohort study. Clin Exp Nephrol 2021;25:410–7. 10.1007/s10157-020-02010-7 33411113

[57] Li JF, Qu X, Gao Z et al. Association between dosing of spironolactone and outcomes in heart failure with preserved ejection fraction patients combined with chronic kidney disease——balance of efficacy and risk. Front Pharmacol 2023;14:1084442. 10.3389/fphar.2023.1084442 36778020 PMC9911545

[58] Kanda E, Rastogi A, Murohara T et al. Clinical impact of suboptimal RAASi therapy following an episode of hyperkalemia. BMC Nephrol 2023;24:18. 10.1186/s12882-022-03054-5 36658531 PMC9854063

[59] Lin DSH, Lin FJ, Lin YS et al. The effects of mineralocorticoid receptor antagonists on cardiovascular outcomes in patients with end-stage renal disease and heart failure. Eur J Heart Fail 2023;25:98–107. 10.1002/ejhf.2740 36404402

[60] Tumlin JA, Kopyt NP, Wilson DJ. Safety and efficacy of maximally tolerated RAS therapy alone or in combination with spironolactone in diabetic kidney disease: effect on proteinuria and eGFR in the MRA-ACE trial. J Am Soc Nephrol 2022;33:677. 10.1681/ASN.20223311S1677c35110363

[61] Sadjadi SA, McMillan JI, Jaipaul N et al. A comparative study of the prevalence of hyperkalemia with the use of angiotensinconverting enzyme inhibitors versus angiotensin receptor blockers. Ther Clin Risk Manage 2009;5:547–52. 10.2147/tcrm.s5176 PMC2710386 19707264

[62] Khosla N, Kalaitzidis R, Bakris GL. Predictors of hyperkalemia risk following hypertension control with aldosterone blockade. Am J Nephrol 2009;30:418–24. 10.1159/000237742 19738369

[63] Weinberg JM, Appel LJ, Bakris G et al. Risk of hyperkalemia in nondiabetic patients with chronic kidney disease receiving antihypertensive therapy. Arch Intern Med 2009;169:1587–94. 10.1001/archinternmed.2009.284 19786678

[64] Knoll GA, Sahgal A, Nair RC et al. Renin-angiotensin system blockade and the risk of hyperkalemia in chronic hemodialysis patients. Am J Med 2002;112:110–4. 10.1016/S0002-9343(01)01068-3 11835948

[65] Hayashi K, Kumagai H, Saruta T. Effect of efonidipine and ACE inhibitors on proteinuria in human hypertension with renal impairment. Am J Hypertens 2003;16:116–22. 10.1016/S0895-7061(02)03147-3 12559677

[66] Adelborg K, Nicolaisen SK, Hasvold P et al. Predictors for repeated hyperkalemia and potassium trajectories in high-risk patients—A population-based cohort study. PLoS One 2019;14:e0218739. 10.1371/journal.pone.0218739 31226134 PMC6588240

[67] Sengul E, Sahin T, Sevin E et al. Effect of spironolactone on urinary protein excretion in patients with chronic kidney disease. Ren Fail 2009;31:928–32. 10.3109/08860220903216121 20030528

[68] Abolghasmi R, Taziki O. Efficacy of low dose spironolactone in chronic kidney disease with resistant hypertension. Saudi J Kidney Dis Transplant 2011;22:75–8. 21196617

[69] Frohlich H, Nelges C, Tager T et al. Long-term changes of renal function in relation to ace inhibitor/angiotensin receptor blocker dosing in patients with heart failure and chronic kidney disease. Am Heart J 2016;178:28–36. 10.1016/j.ahj.2016.03.024 27502849

[70] Eschalier R, McMurray JJV, Swedberg K et al. Safety and efficacy of eplerenone in patients at high risk for hyperkalemia and/or worsening renal function: analyses of the EMPHASIS-HF study subgroups (eplerenone in mild patients hospitalization and survival study in heart failure). J Am Coll Cardiol 2013;62:1585–93. 10.1016/j.jacc.2013.04.086 23810881

[71] Polson M, Lord TC, Kangethe A et al. Clinical and economic impact of hyperkalemia in patients with chronic kidney disease and heart failure. J Manag Care Spec Pharm 2017;23:S2–9. 10.18553/jmcp.2017.23.4-a.s2a PMC10408418 28485202

[72] Wetmore JB, Yan H, Horne L et al. Risk of hyperkalemia from renin-angiotensin-aldosterone system inhibitors and factors associated with treatment discontinuities in a real-world population. Nephrol Dial Transplant 2021;36:826–39. 10.1093/ndt/gfz263 31846025

[73] Yang A, Shi M, Lau ESH et al. Clinical outcomes following discontinuation of renin-angiotensin-system inhibitors in patients with type 2 diabetes and advanced chronic kidney disease: a prospective cohort study. eClinicalMedicine 2023;55:101751. 10.1016/j.eclinm.2022.101751 36457651 PMC9706514

[74] Buckallew AR, Tellor KB, Watson R et al. Evaluation of the safety and tolerability of spironolactone in patients with heart failure and chronic kidney disease. Eur J Clin Pharmacol 2021;77:955–60. 10.1007/s00228-020-03069-7 33449127

[75] De Rosa ML, Cardace P, Rossi M et al. Evaluation of long-term efficacy and tolerability of irbesartan in elderly hypertensive patients with renal impairment in an open-label study. Curr Ther Res 2002;63:201–15. 10.1016/S0011-393X(02)80027-3

[76] Ren H, Leon SJ, Whitlock R et al. Prescription patterns of sodium and calcium polystyrene sulfonate in patients with hyperkalemia and chronic kidney disease receiving RAAS inhibitors. Clin Kidney J 2022;15:1713–9. 10.1093/ckj/sfac077 36003673 PMC9394712

[77] Riccio E, Capuano I, Buonanno P et al. RAAS inhibitor prescription and hyperkalemia event in patients with chronic kidney disease: a single-center retrospective study. Front Cardiovasc Med 2022;9:824095. 10.3389/fcvm.2022.824095 35224054 PMC8874323

[78] Uchida HA, Nakajima H, Hashimoto M et al. Efficacy and safety of esaxerenone in hypertensive patients with diabetic kidney disease: a multicenter, open-label, prospective study. Adv Ther 2022;39:5158–75. 36070133 10.1007/s12325-022-02294-z PMC9449923

[79] Tang SCW, Chan KW, Ip DKM et al. Direct Renin Inhibition in Non-diabetic chronic Kidney disease (DRINK): a prospective randomized trial. Nephrol Dial Transplant 2021;36:1648–56. 10.1093/ndt/gfaa085 32617578

[80] Bakris G, Pergola PE, Delgado B et al. Effect of KBP-5074 on blood pressure in advanced chronic kidney disease: results of the BLOCK-CKD study. Hypertension 2021;78:74–81. 10.1161/HYPERTENSIONAHA.121.17073 33966452 PMC8189259

[81] Einhorn LM, Zhan M, Hsu VD et al. The frequency of hyperkalemia and its significance in chronic kidney disease. Arch Intern Med 2009;169:1156–62. 10.1001/archinternmed.2009.132 19546417 PMC3544306

[82] Johnson ES, Weinstein JR, Thorp ML et al. Predicting the risk of hyperkalemia in patients with chronic kidney disease starting lisinopril. Pharmacoepidemiol Drug Saf 2010;19:266–72. 10.1002/pds.1923 20112435 PMC3905673

[83] Raebel MA, Ross C, Xu S et al. Diabetes and drug-associated hyperkalemia: effect of potassium monitoring. J Gen Intern Med 2010;25:326–33. 10.1007/s11606-009-1228-x 20087674 PMC2842549

[84] Frimodt-Moller M, Hoj Nielsen A, Strandgaard S et al. Feasibility of combined treatment with enalapril and candesartan in advanced chronic kidney disease. Nephrol Dial Transplant 2010;25:842–7. 10.1093/ndt/gfp547 19903661

[85] Taheri S, Mortazavi M, Pourmoghadas A et al. A prospective double-blind randomized placebo-controlled clinical trial to evaluate the safety and efficacy of spironolactone in patients with advanced congestive heart failure on continuous ambulatory peritoneal dialysis. *Saudi J Kidney Dis Transpl* 2012;23:507–12. 22569436

[86] Espinel E, Joven J, Gil I et al. Risk of hyperkalemia in patients with moderate chronic kidney disease initiating angiotensin converting enzyme inhibitors or angiotensin receptor blockers: a randomized study. BMC Res Notes 2013;6:306. 10.1186/1756-0500-6-306 23915518 PMC3750227

[87] Boesby L, Elung-Jensen T, Klausen TW et al. Moderate antiproteinuric effect of add-on aldosterone blockade with eplerenone in non-diabetic chronic kidney disease. a randomized cross-over study. PLoS One 2011;6:e26904. 10.1371/journal.pone.0026904 22073219 PMC3208556

[88] Edwards NC, Steeds RP, Chue CD et al. The safety and tolerability of spironolactone in patients with mild to moderate chronic kidney disease. Brit J Clin Pharmacol 2012;73:447–54. 10.1111/j.1365-2125.2011.04102.x 21950312 PMC3370349

[89] Tseng WC, Liu JS, Hung SC et al. Effect of spironolactone on the risks of mortality and hospitalization for heart failure in pre-dialysis advanced chronic kidney disease: a nationwide population-based study. Int J Cardiol 2017;238:72–8. 10.1016/j.ijcard.2017.03.080 28363684

[90] Ando K, Ohtsu H, Uchida S et al. Anti-albuminuric effect of the aldosterone blocker eplerenone in non-diabetic hypertensive patients with albuminuria: a double-blind, randomised, placebo-controlled trial. Lancet Diabetes Endocrinol 2014;2:944–53. 10.1016/S2213-8587(14)70194-9 25466242

[91] Maddirala S, Khan A, Vincent A et al. Effect of angiotensin converting enzyme inhibitors and angiotensin receptor blockers on serum potassium levels and renal function in ambulatory outpatients: risk factors analysis. Am J Med Sci 2008;336:330–5. 10.1097/MAJ.0b013e3181836ac7 18854676

[92] Rysava R, Tesar V, Merta M. Effect of telmisartan on blood pressure control and kidney function in hypertensive, proteinuric patients with chronic kidney disease. Blood Press Monit 2005;10:207–13. 10.1097/01.mbp.0000172708.97534.15 16077267

[93] Pitt B, Filippatos G, Agarwal R et al. Cardiovascular events with finerenone in kidney disease and type 2 diabetes. N Engl J Med 2021;385:2252–63. 10.1056/NEJMoa2110956 34449181

[94] Bakris GL, Agarwal R, Anker SD et al. Effect of finerenone on chronic kidney disease outcomes in type 2 diabetes. N Engl J Med 2020;383:2219–29. 10.1056/NEJMoa2025845 33264825

[95] Agarwal R, Joseph A, Anker SD et al. Hyperkalemia risk with finerenone: results from the FIDELIO-DKD trial. J Am Soc Nephrol 2022;33:225–37. 10.1681/ASN.2021070942 34732509 PMC8763180

[96] Zhang H. Effect of finerenone on CKD outcomes in type 2 diabetes: a Chinese subgroup analysis of the FIDELIO-DKD study. Kidney week. J Am Soc Nephrol 2022;33:678.

[97] Filippatos G, Anker SD, Agarwal R et al. Finerenone and cardiovascular outcomes in patients with chronic kidney disease and type 2 diabetes. Circulation 2021;143:540–52. 10.1161/CIRCULATIONAHA.120.051898 33198491 PMC7864612

[98] Rossing P, Filippatos G, Agarwal R et al. Finerenone in predominantly advanced CKD and type 2 diabetes with or without sodium-glucose cotransporter-2 inhibitor therapy. Kidney Int Rep 2022;7:36–45. 10.1016/j.ekir.2021.10.008 35005312 PMC8720648

[99] Rossing P, Burgess E, Agarwal R et al. Finerenone in patients with chronic kidney disease and type 2 diabetes according to baseline hba1c and insulin use: an analysis from the FIDELIO-DKD study. Diabetes Care 2022;45:e888–97. 10.2337/dc21-1944 PMC9271031 35061867

[100] Zhang H, Xie J, Hao C et al. Finerenone in patients with chronic kidney disease and type 2 diabetes: the FIDELIO-DKD subgroup from China. Kidney Dis 2023;9:498–506. 10.1159/000531997 PMC10712967 38089437

[101] Fang G, Annis IE, Farley JF et al. Incidence of and risk factors for severe adverse events in elderly patients taking angiotensin-converting enzyme inhibitors or angiotensin II receptor blockers after an acute myocardial infarction. Pharmacotherapy 2018;38:29–41. 10.1002/phar.2051 29059475 PMC5766359

[102] Silvarino R, Rios P, Baldovinos G et al. Is chronic kidney disease progression influenced by the type of renin-angiotensin-system blocker used? Nephron 2019;143:100–7. 10.1159/000500925 31203280

[103] Linde C, Bakhai A, Furuland H et al. Real-world associations of renin-angiotensin-aldosterone system inhibitor dose, hyperkalemia, and adverse clinical outcomes in a cohort of patients with new-onset chronic kidney disease or heart failure in the United Kingdom. J Am Heart Assoc 2019;8:e012655. 10.1161/JAHA.119.012655 31711387 PMC6915283

[104] Iskandar H, Hidayat, WU, Hersunaryati YA et al. A retrospective study on the potential drug interaction between angiotensin converting enzyme inhibitor or angiotensin receptor antagonist and other drugs in end-stage chronic renal failure patients. Int Res J Pharmacy 2012;3:86–9.

[105] Kashihara N, Kohsaka S, Kanda E et al. Hyperkalemia in real-world patients under continuous medical care in Japan. Kidney Int Rep 2019;4:1248–60. 10.1016/j.ekir.2019.05.018 31517144 PMC6734103

[106] Saito Y, Yamamoto H, Nakajima H et al. Incidence of and risk factors for newly diagnosed hyperkalemia after hospital discharge in non-dialysis-dependent CKD patients treated with RAS inhibitors. PLoS One 2017;12:e0184402. 10.1371/journal.pone.0184402 28877239 PMC5587314

[107] Pitt B, Kober L, Ponikowski P et al. Safety and tolerability of the novel non-steroidal mineralocorticoid receptor antagonist BAY 94-8862 in patients with chronic heart failure and mild or moderate chronic kidney disease: a randomized, double-blind trial. Eur Heart J 2013;34:2453–63. 10.1093/eurheartj/eht187 23713082 PMC3743070

[108] Persson F, Lewis JB, Lewis EJ et al. Impact of baseline renal function on the efficacy and safety of Aliskiren added to losartan in patients with type 2 diabetes and nephropathy. Diabetes Care 2010;33:2304–9. 10.2337/dc10-0833 20693353 PMC2963484

[109] Gwoo S, Kim YN, Shin HS et al. Predictors of hyperkalemia risk after hypertension control with aldosterone blockade according to the presence or absence of chronic kidney disease. Nephron Clin Pract 2014;128:381–6. 10.1159/000369138 25572273

[110] Vukusich A, Kunstmann S, Varela C et al. A randomized, double-blind, placebo-controlled trial of spironolactone on carotid intima-media thickness in nondiabetic hemodialysis patients. Clin J Am Soc Nephrol 2010;5:1380–7. 10.2215/CJN.09421209 20522535 PMC2924413

[111] Walsh M, Manns B, Garg AX et al. The safety of eplerenone in hemodialysis patients: a noninferiority randomized controlled trial. Clin J Am Soc Nephrol 2015;10:1602–8. 10.2215/CJN.12371214 26138259 PMC4559523

[112] An J, Niu F, Sim JJ. Cardiovascular and kidney outcomes of spironolactone or eplerenone in combination with ACEI/ARBs in patients with diabetic kidney disease. Pharmacotherapy 2021;41:998–1008. 10.1002/phar.2633 34655484

[113] Qu X, Yao H, Chen C et al. Spironolactone improves the all-cause mortality and re-hospitalization rates in acute myocardial infarction with chronic kidney disease patients. Front Pharmacol 2021;12:632978. 10.3389/fphar.2021.632978 34135751 PMC8201517

[114] Edwards NC, Price AM, Mehta S et al. Effects of spironolactone and chlorthalidone on cardiovascular structure and function in chronic kidney disease: a randomized, open-label trial. Clin J Am Soc Nephrol 2021;16:1491–501. 10.2215/CJN.01930221 34462286 PMC8499017

[115] Straburzynska-Migaj E, Senni M, Wachter R et al. Initiation of sacubitril/valsartan in patients with renal impairment early after acute decompensated heart failure in the TRANSITION study. Eur J Heart Fail 2021;23:46.

[116] Ruilope LM, Aldigier JC, Ponticelli C et al. Safety of the combination of valsartan and benazepril in patients with chronic renal disease. J Hypertens 2000;18:89–95. 10.1097/00004872-200018010-00013 10678548

[117] De Rosa ML, De Cristofaro A, Rossi M et al. Irbesartan effects on renal function in patients with renal impairment and hypertension: a drug-withdrawal study. J Cardiovasc Pharmacol 2001;38:482–9. 10.1097/00005344-200109000-00017 11486253

[118] Hammer F, Malzahn U, Donhauser J et al. A randomized controlled trial of the effect of spironolactone on left ventricular mass in hemodialysis patients. Kidney Int 2019;95:983–91. 10.1016/j.kint.2018.11.025 30712923

[119] Charytan DM, Himmelfarb J, Ikizler TA et al. Safety and cardiovascular efficacy of spironolactone in dialysis-dependent ESRD (SPin-D): a randomized, placebo-controlled, multiple dosage trial. Kidney Int 2019;95:973–82. 10.1016/j.kint.2018.08.034 30473139 PMC6431563

[120] Haynes R, Judge PK, Staplin N et al. Effects of sacubitril/valsartan versus irbesartan in patients with chronic kidney disease: a randomized double-blind trial. Circulation 2018;138:1505–14. 10.1161/CIRCULATIONAHA.118.034818 30002098

[121] Lee J-H, Kwon YE, Park JT et al. The effect of renin-angiotensin system blockade on renal protection in chronic kidney disease patients with hyperkalemia. J Renin Angiotensin Aldosterone Syst 2014;15:491–7. 10.1177/1470320313507122 25143329

[122] Agrawal A, Kamila S, Reddy S et al. Effect of telmisartan on kidney function in patients with chronic kidney disease: an observational study. J Drug Assess 2016;5:24–28. 10.1080/21556660.2016.1252380 27994942 PMC5136975

[123] Woo K-T, Choong H-L, Wong K-S et al. A retrospective aliskiren and losartan study in non-diabetic chronic kidney disease. World J Nephrol 2013;2:129–35. 24255896 10.5527/wjn.v2.i4.129 PMC3832869

[124] Yildirim T, Arici M, Piskinpasa S et al. Major barriers against renin-angiotensin-aldosterone system blocker use in chronic kidney disease stages 3-5 in clinical practice: a safety concern? Ren Fail 2012;34:1095–9. 10.3109/0886022X.2012.717478 22950572

[125] Pisoni R, Acelajado MC, Cartmill FR et al. Long-term effects of aldosterone blockade in resistant hypertension associated with chronic kidney disease. J Hum Hypertens 2012;26:502–6. 10.1038/jhh.2011.60 21677673 PMC5636621

[126] Hirai T, Yamaga R, Fujita A et al. Low body mass index is a risk factor for hyperkalaemia associated with angiotensin converting enzyme inhibitors and angiotensin II receptor blockers treatments. J Clin Pharm Ther 2018;43:829–35. 10.1111/jcpt.12720 29908131

[127] Jun M, Jardine MJ, Perkovic V et al. Hyperkalemia and renin-angiotensin aldosterone system inhibitor therapy in chronic kidney disease: a general practice-based, observational study. PLoS One 2019;14:e0213192. 10.1371/journal.pone.0213192 30845156 PMC6405190

[128] Belmar Vega L, Galabia ER, Bada da Silva J et al. Epidemiology of hyperkalemia in chronic kidney disease. Nefrologia 2019;39:277–86. 10.1016/j.nefro.2018.11.011 30898450

[129] Beldhuis IE, Myhre PL, Claggett B et al. Efficacy and safety of spironolactone in patients with HFpEF and chronic kidney disease. JACC Heart Fail 2019;7:25–32. 30606484 10.1016/j.jchf.2018.10.017

[130] Heshka J, Ruzicka M, Hiremath S et al. Spironolactone for difficult to control hypertension in chronic kidney disease: an analysis of safety and efficacy. J Am Soc Hypertens 2010;4:295–301. 10.1016/j.jash.2010.09.006 21130976

[131] Johnson M, Morrison FJ, McMahon G et al. Outcomes in patients with cardiometabolic disease who develop hyperkalemia while treated with a renin-angiotensin-aldosterone system inhibitor. Am Heart J 2023;258:49–59. 10.1016/j.ahj.2023.01.002 36642227

[132] Provenzano M, Puchades MJ, Garofalo C et al. Albuminuria-lowering effect of dapagliflozin, eplerenone, and their combination in patients with chronic kidney disease: a randomized cross-over clinical trial. J Am Soc Nephrol 2022;33:1569–80. 10.1681/ASN.2022020207 35440501 PMC9342643

[133] Leon SJ, Whitlock R, Rigatto C et al. Hyperkalemia-related discontinuation of renin-angiotensin-aldosterone system inhibitors and clinical outcomes in CKD: a population-based cohort study. Am J Kidney Dis 2022;80:164–73.e1. 10.1053/j.ajkd.2022.01.002 35085685

[134] Salik JR, Golas SB, McCoy TH. A comparative study assessing the incidence and degree of hyperkalemia in patients on angiotensin-converting enzyme inhibitors versus angiotensin-receptor blockers. J Hum Hypertens 2022;36:485–7. 10.1038/s41371-021-00625-1 34650213

[135] Tokunaga M, Kabashima N, Serino R et al. Renoprotective effects of telmisartan in patients with advanced chronic kidney disease. Clin Nephrol 2010;73:139–46. 10.5414/CNP73139 20129021

[136] Bhandari S, Mehta S, Khwaja A et al. Renin–angiotensin system inhibition in advanced chronic kidney disease. N Engl J Med 2022;387:2021–32. 10.1056/NEJMoa2210639 36326117

[137] Fu EL, Evans M, Clase CM et al. Stopping renin-angiotensin system inhibitors in patients with advanced CKD and risk of adverse outcomes: a nationwide study. J Am Soc Nephrol 2021;32:424. 10.1681/ASN.2020050682 33372009 PMC8054897

[138] Ahmed AK, Kamath NS, El Kossi M et al. The impact of stopping inhibitors of the renin–angiotensin system in patients with advanced chronic kidney disease. Nephrol Dial Transplant 2010;25:3977–82. 10.1093/ndt/gfp511 19820248

[139] Parmar S, Ali M, Lopez T et al. WCN24-2118 Hyperkalaemia in patients with chronic kidney disease and heart failure. Kidney Int Rep 2024;9:S327–8. 10.1016/j.ekir.2024.02.633

[140] Sevamontree C, Jintajirapan S, Phakdeekitcharoen P et al. The prevalence and risk factors of hyperkalemia in the outpatient setting. Int J Nephrol 2024;2024:1–9. 10.1155/2024/5694131 PMC10824579 38292832

[141] Valdivielso JM, Carriazo S, Martin M et al. Gender-specific risk factors and outcomes of hyperkalemia in CKD patients: smoking as a driver of hyperkalemia in men. Clin Kidney J 2024;17:sfad212. 10.1093/ckj/sfad212 38186899 PMC10768768

[142] Gülçiçek S, Seyahi N. Hyperkalemia: a cause of non-adherence to renin-angiotensin-aldosterone system inhibitors in chronic kidney disease: a retrospective study. Istanbul Med J 2023;24:404–11. 10.4274/imj.galenos.2023.55889

[143] Zhou J, Jin X, Zhou J et al. Clinical outcomes by serum potassium levels for patients hospitalized for heart failure: Secondary analysis of data from the China National Heart Failure Registry. Clin Cardiol 2023;46:1345–52. 10.1002/clc.24114 37577821 PMC10642319

[144] Qadir A, Ullah Z, Khalil MD et al. Frequency of hyperkalaemia in non-dialysis dependent chronic kidney disease (CKD) patients. Medical Forum Monthly 2023;34.

[145] de Rooij EN, de Fijter JW, Le Cessie S et al. Serum potassium and risk of death or kidney replacement therapy in older people with CKD stages 4-5: eight-year follow-up. Am J Kidney Dis 2023;82:257–66.e1. 10.1053/j.ajkd.2023.03.008 37182596

[146] Perez-Navarro LM, Valdez-Ortiz R, Reyna-Blanco J. Prevalence and factors associated with hyperkalemia in outpatients with CKD. J Am Soc Nephrol 2023;34:397. 10.1681/ASN.20233411S1397a

[147] Bakris GL, Agiro A, Greatsinger A et al. REVOLUTIONIZE III: consequences of recurrent hyperkalemia on healthcare resource utilization and cost. J Am Soc Nephrol 2023;34:190–1. 10.1681/ASN.20233411S1190d

[148] Gaol DL, Nilasari D, Halim DS et al. WCN24-2258 Serum potassium profile and associated factors in hemodialysis patients: single center study. Kidney Int Rep 2024;9:S8. 10.1016/j.ekir.2024.02.028

[149] Rastogi A, Pollack C, Lesen E et al. Association between reduced RAASi therapy and progression to ESKD in hyperkalemic CKD patients. J Am Soc Nephrol 2023;34:379. 10.1681/ASN.20233411S1379b

[150] Marup FH, Peters C, Nielsen S et al. Potassium levels and eGFR do not predict severe hyperkalemia following spironolactone introduction in patients with CKD at high risk of hyperkalemia. Nephrol Dial Transplant 2023;38:i668–9. 10.1093/ndt/gfad063c_3068

[151] Zhou Q, Yu W, Shao X et al. Efficacy and safety of sacubitril/valsartan in patients with stage 3B-5 CKD and hypertension. Nephrol Dial Transplant 2023;38:i665–7. 10.1093/ndt/gfad063c_5705

[152] Rajak K, Halder A, Khanal R et al. Renal dysfunction associated with finerenone—a pharmacovigilance analysis. J Am Coll Cardiol 2023;81:583. 10.1016/S0735-1097(23)01027-6

[153] Bornstein SR, de Zeeuw D, Heerspink HJ et al. Aldosterone synthase inhibitor (BI 690517) therapy for people with diabetes and albuminuric chronic kidney disease: a multicentre, randomized, double-blind, placebo-controlled, phase I trial. Diabetes Obes Metab 2024;26:2128–38. 10.1111/dom.15518 38497241

[154] Jiménez-Marrero S, Cainzos-Achirica M, Monterde D et al. Serum potassium abnormalities, renin-angiotensin-aldosterone system inhibitor discontinuation, and clinical outcomes in patients with chronic cardiovascular, metabolic, and renal conditions: a population-based analysis. Eur J Inter Med 2024;125:89–97. 10.1016/j.ejim.2024.03.021 38548513

[155] García-Prieto A, Verdalles Ú, de José AP et al. Renin–angiotensin–aldosterone system blockers effect in chronic kidney disease progression in hypertensive elderly patients without proteinuria: PROERCAN trial. Hipertens Riesgo Vasc 2024;41:95–103. 10.1016/j.hipert.2023.11.005 38508877

[156] An J, Zhou H, Ni L et al. Discontinuation of renin-angiotensin-aldosterone system inhibitors secondary to hyperkalemia translates into higher cardiorenal outcomes. Am J Nephrol 2023;54:258–67. 10.1159/000531102 37231821 PMC10623389

[157] Agiro A, AN A, Cook EE et al. Real-world modifications of renin-angiotensin-aldosterone system inhibitors in patients with hyperkalemia initiating sodium zirconium cyclosilicate therapy: the OPTIMIZE I study. Adv Ther 2023;40:2886–901. 10.1007/s12325-023-02518-w 37140706 PMC10220114

[158] Gregg LP, Richardson P, Herrera MA et al. Documented adverse drug reactions and discontinuation of angiotensin converting enzyme inhibitors and angiotensin receptor blockers in chronic kidney disease. Am J Nephrol 2023;54:126–35. 10.1159/000530988 37231800 PMC10424561

[159] Chinnadurai R, Rengarajan S, Budden JJ et al. Maintaining renin-angiotensin-aldosterone system inhibitor treatment with patiromer in hyperkalaemic chronic kidney disease patients: comparison of a propensity-matched real-world population with AMETHYST-DN. Am J Nephrol 2023;54:408–15. 10.1159/000533753 37725919

[160] Nicholas SB, Correa-Rotter R, Desai N et al. Interim results from FINE-REAL: a prospective study providing insights into the use of finerenone in routine clinical settings. J Am Soc Nephrol 2023;34:857. 10.1681/ASN.20233411S1857a 39340711 PMC11649709

[161] McFarland KL, Sheridan EA. A retrospective analysis of sacubitril/valsartan in heart failure and chronic kidney disease. J Pharm Technol 2023;39:117–22. 10.1177/87551225231168543 37323769 PMC10268039

[162] Ding Y, Wan L, Z-c Z et al. Effects of sacubitril-valsartan in patients undergoing maintenance dialysis. Ren Fail 2023;45:2222841. 10.1080/0886022X.2023.2222841 37334931 PMC10281424

[163] Jariwala P, Pramod G. Effect of fineronone on heart failure outcomes in type 2 diabetes with chronic kidney disease: an early Indian experience. Indian Heart J 2023;75:S49. 10.1016/j.ihj.2023.11.105

[164] Svensson M, Kim K, Cars T et al. EE268 healthcare costs and all-cause mortality following a hyperkalemia event and reduction of RAASi therapy in Sweden. Value Health 2023;26:S103. 10.1016/j.jval.2023.09.535

[165] Tuttle KR, Hauske SJ, Canziani ME et al. Efficacy and safety of aldosterone synthase inhibition with and without empagliflozin for chronic kidney disease: a randomised, controlled, phase 2 trial. Lancet 2024;403:379–90. 10.1016/S0140-6736(23)02408-X 38109916

[166] Guney I, Selcuk NY, Altintepe L et al. Antifibrotic effects of aldosterone receptor blocker (spironolactone) in patients with chronic kidney disease. Ren Fail 2009;31:779–84. 10.3109/08860220903150312 19925284

[167] Edwards NC, Steeds RP, Stewart PM et al. Effect of spironolactone on left ventricular mass and aortic stiffness in early-stage chronic kidney disease: a randomized controlled trial. J Am Coll Cardiol 2009;54:505–12. 10.1016/j.jacc.2009.03.066 19643310

[168] Obertynska O. The risks and benefits of spironolactone use in heart failure with a reduced left ventricular ejection fraction and chronic kidney disease. Eur Heart J 2021;42:895. 10.1093/eurheartj/ehab724.0895

[169] An JN, Lee JP, Jeon HJ et al. Severe hyperkalemia requiring hospitalization: predictors of mortality. Crit Care 2012;16:1–14. 10.1186/cc11872 PMC3672605 23171442

[170] Gilligan S, Raphael KL. Hyperkalemia and hypokalemia in CKD: prevalence, risk factors, and clinical outcomes. Adv Chronic Kidney Dis 2017;24:315–8. 10.1053/j.ackd.2017.06.004 29031358

[171] Kovesdy CP. Updates in hyperkalemia: outcomes and therapeutic strategies. Rev Endocr Metab Disord 2017;18:41–7. 10.1007/s11154-016-9384-x 27600582 PMC5339065

[172] Matus Gonzalez A, Evangelidis N, Howell M et al. Outcomes for clinical trials involving adults with chronic kidney disease: a multinational Delphi survey involving patients, caregivers and health professionals. Nephrol Dial Transplant 2024;gfae010. 10.1093/ndt/gfae010 38236705

[173] Perazella MA. Drug-induced hyperkalemia: old culprits and new offenders. Am J Med 2000;109:307–14. 10.1016/S0002-9343(00)00496-4 10996582

[174] Fitch K, Woolley JM, Engel T et al. The clinical and economic burden of hyperkalemia on medicare and commercial payers. Am Health Drug Benefits 2017;10:202. 28794824 PMC5536196

[175] Evans M, Lewis RD, Morgan AR et al. A narrative review of chronic kidney disease in clinical practice: current challenges and future perspectives. Adv Ther 2022;39:33–43. 10.1007/s12325-021-01927-z 34739697 PMC8569052

