# Supplementary material for: The burden of hyperkalaemia in chronic kidney disease: a systematic literature review
Source: Clin Kidney J. 2025 Apr 29;18(5):sfaf127. doi: 10.1093/ckj/sfaf127 (PMC12082095; doi:10.1093/ckj/sfaf127)

Supplementary Data

Figure S1. PRISMA flowchart – Epidemiology and burden of illness search

Figure S2. PRISMA flowchart – Sub-optimal dosing search

Figure S1. PRISMA flowchart – Sub-optimal dosing search

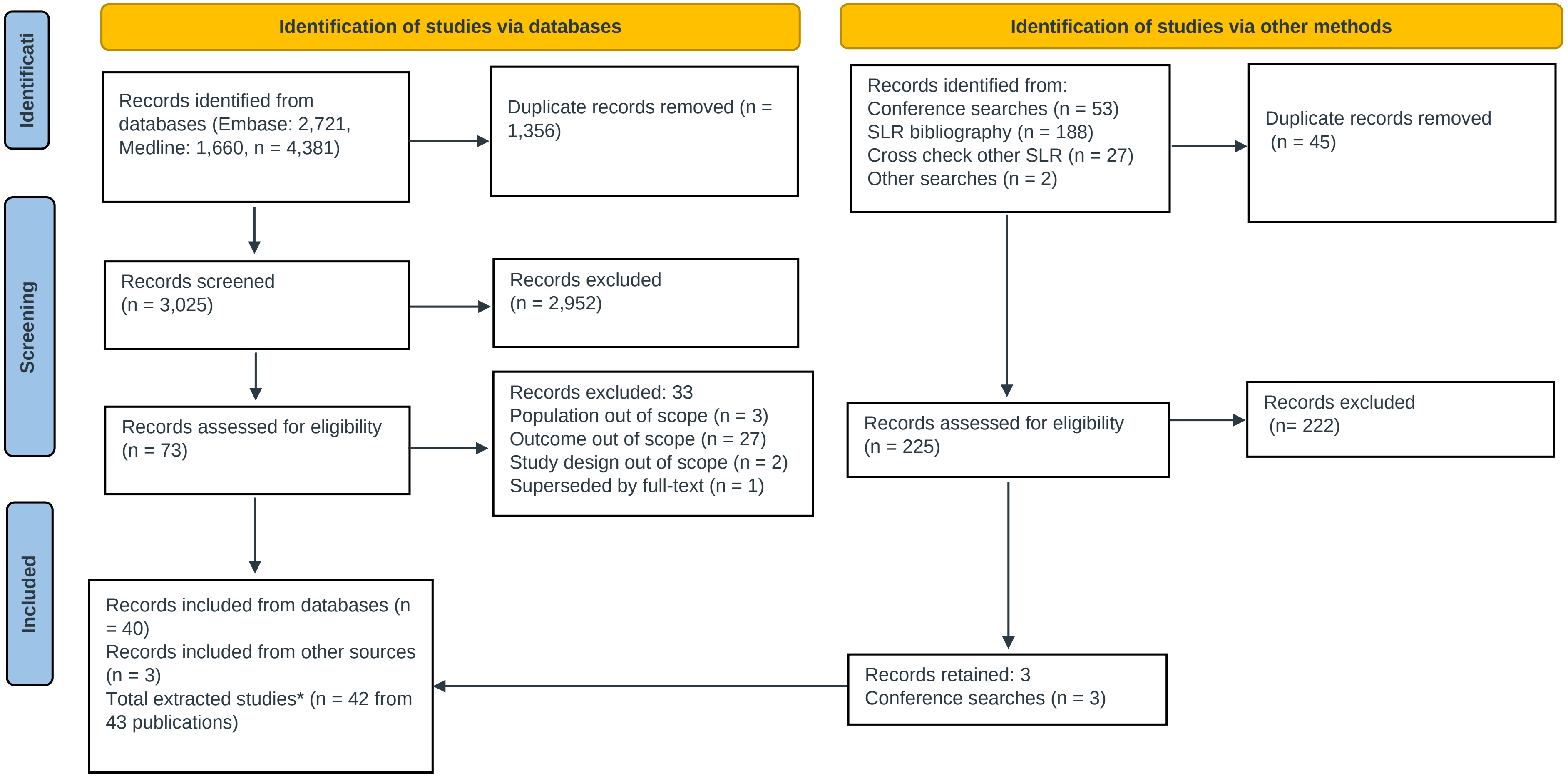

*All reporting data vs control group

Figure S2. PRISMA flowchart – Sub-optimal dosing search

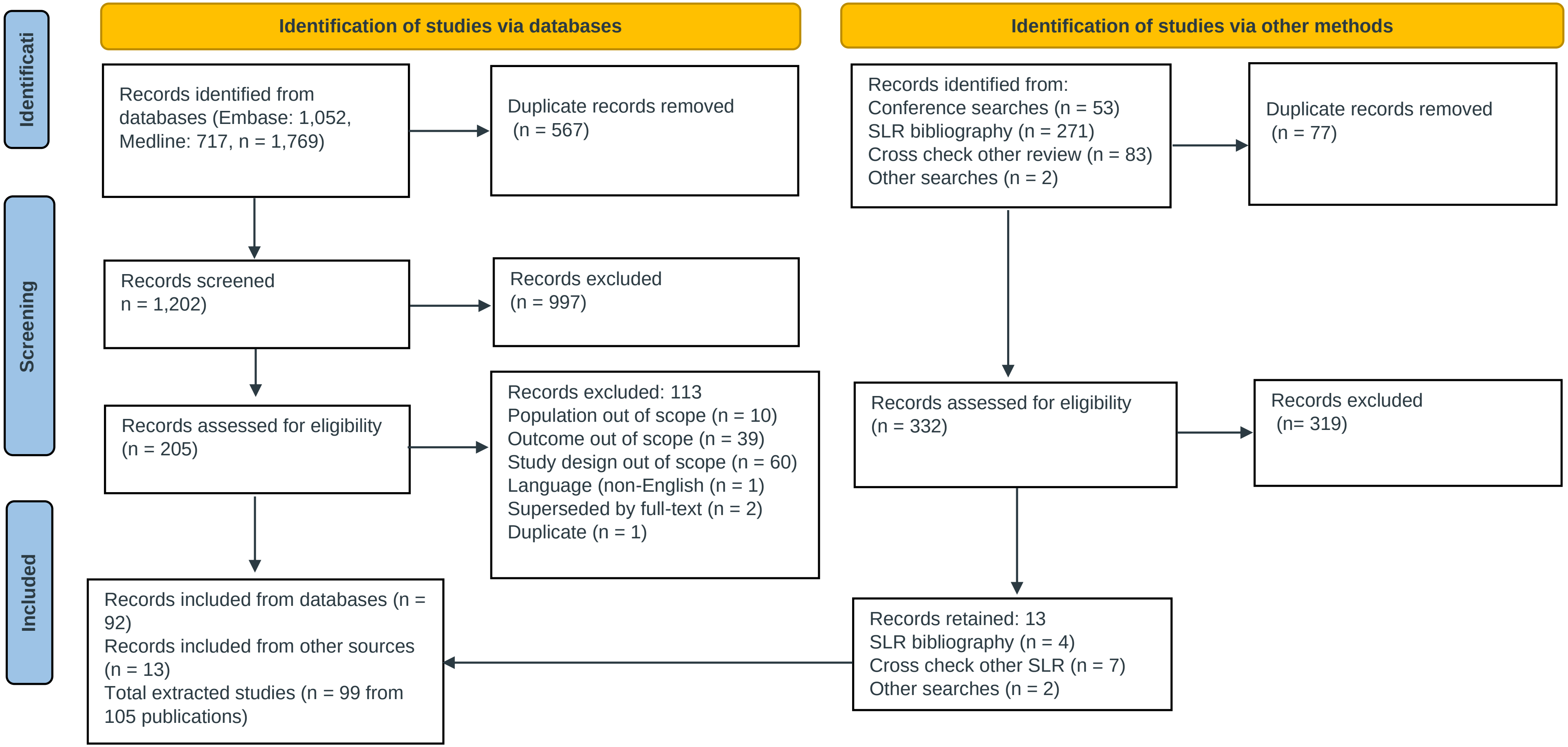

Supplement: sfaf127_Supplemental_Files [file sfaf127_supplemental_files.zip › Supp1_PRISMA_Figures_S1-S2.pdf]
