# Supplementary material for: The burden of hyperkalaemia in chronic kidney disease: a systematic literature review
Source: Clin Kidney J. 2025 Apr 29;18(5):sfaf127. doi: 10.1093/ckj/sfaf127 (PMC12082095; doi:10.1093/ckj/sfaf127)

Supplementary Data

Figure S3. Incidence rate (%) of RAASi-associated hyperkalaemia by CKD stages

Figure S4. Incidence rate (per 100 PYs) of RAASi-associated hyperkalaemia by CKD stages

Figure S5. Impact of sub-optimal RAASi dosing/ discontinuation on mortality

Figure S6. Impact of sub-optimal RAASi dosing/ discontinuation on cardiorenal outcomes

Figure S7. Impact of sub-optimal RAASi dosing on inpatient hospitalisations

Figure S8. Impact of sub-optimal RAASi dosing on healthcare costs

Figure S9. Downs and Black quality assessment summary across non-comparative non-randomised/ observational studies

Figure S10. Downs and Black quality assessment summary across comparative across non-randomised/ observational studies

Figure S3. Incidence rate (%) of RAASi-associated hyperkalaemia by CKD stages

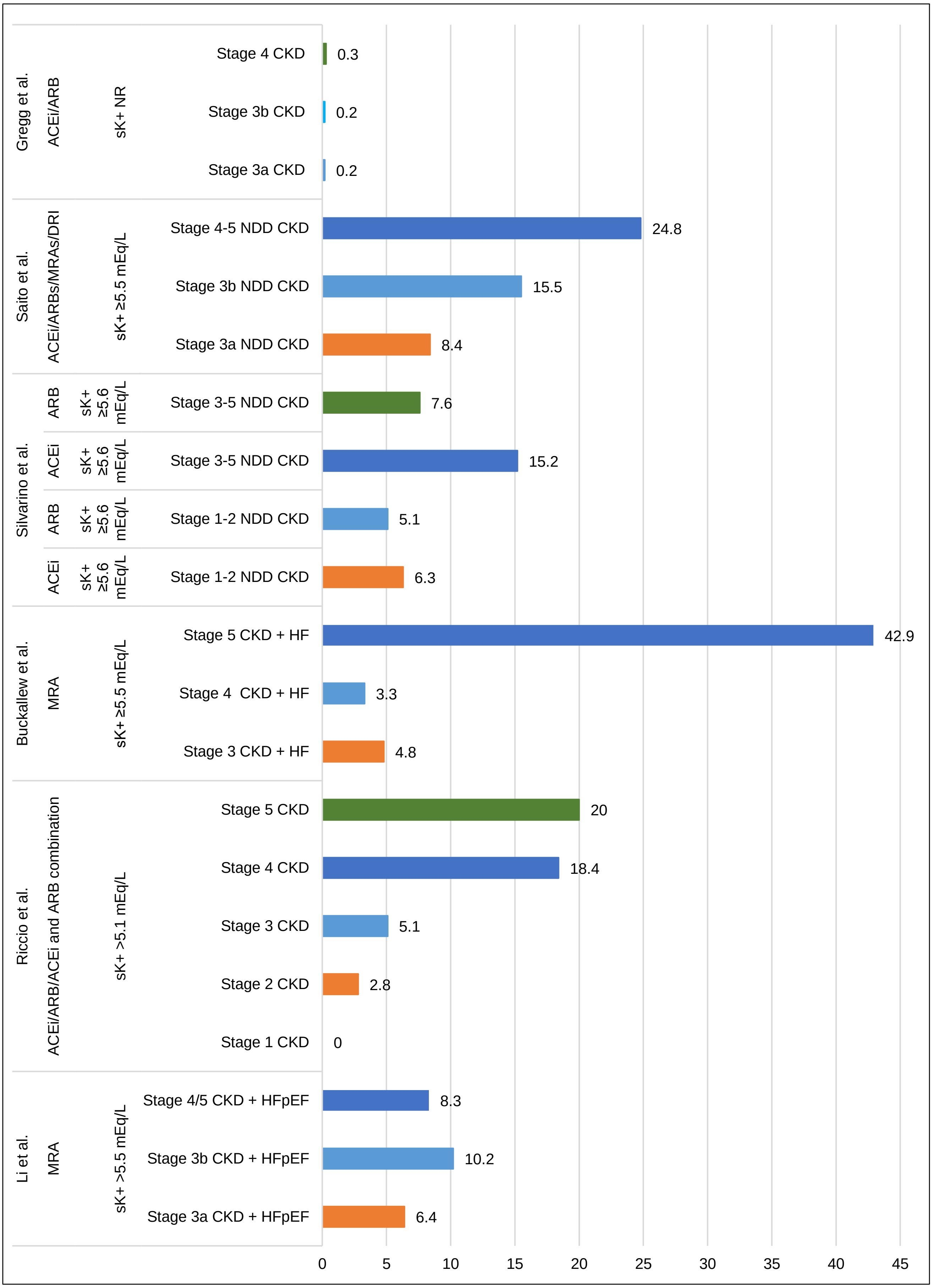

Abbreviations: ACEi: angiotensin converting enzyme inhibitors; ARB: angiotensin receptor blocker; CKD: chronic kidney disease; DRI: direct renin inhibitor; HFpEF: heart failure with preserved ejection fraction; mEq/L: milliequivalents per litre; MRA: mineralocorticoid receptor antagonist; NDD: non-dialysis dependent; RAASi: renin-angiotensin-aldosterone system inhibitors; sK+: serum potassium.

Figure S4. Incidence rate (per 100 PYs) of RAASi-associated hyperkalaemia by CKD stages

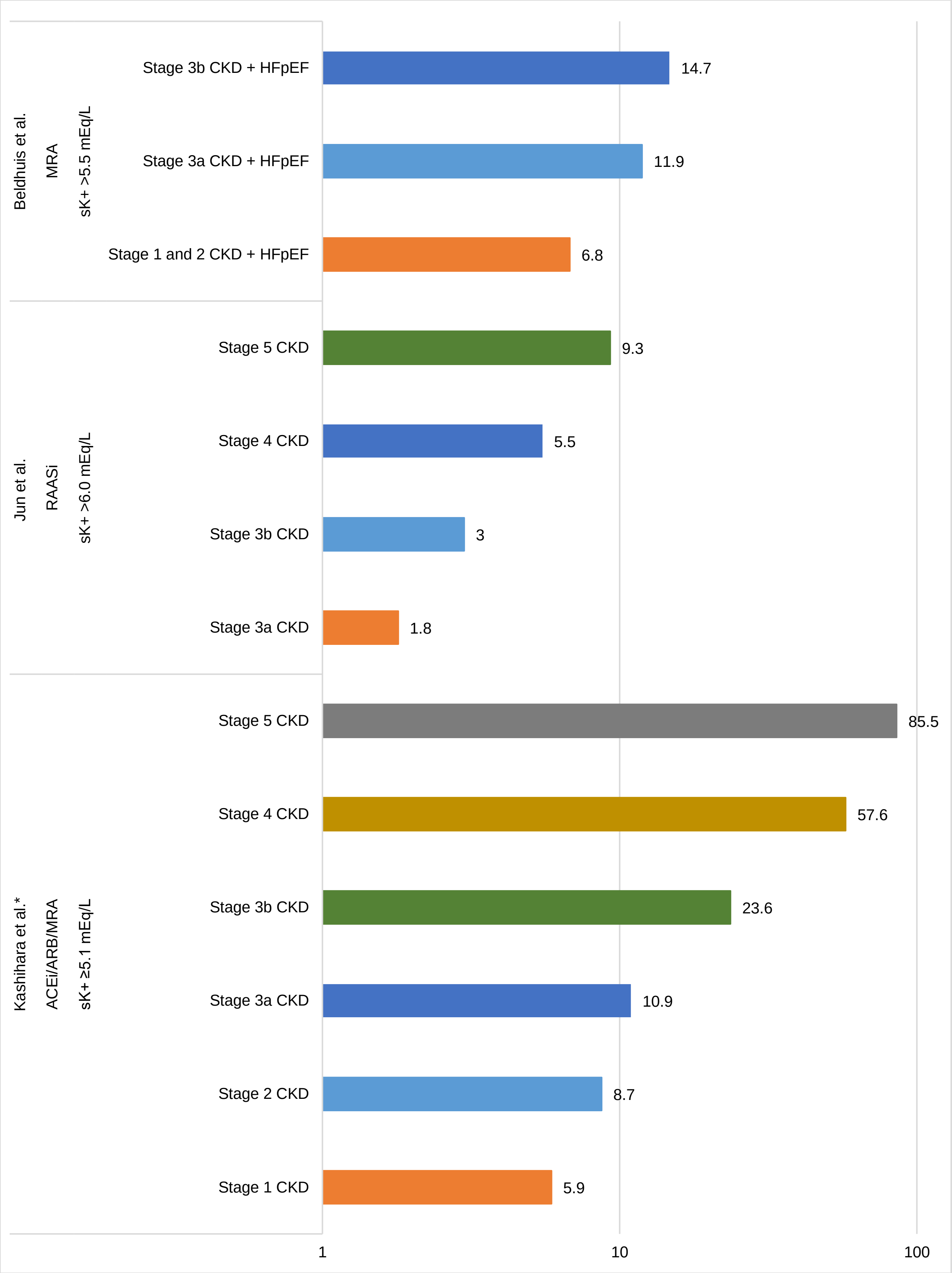

Notes: * In comparison to other studies, Kashihara et al. included around 50% of patients with stage 4-5 CKD and used a minimal threshold for hyperkalaemia (sK+ 5.1 mEq/L)

Abbreviations: ACEi: angiotensin converting enzyme inhibitors; ARB: angiotensin receptor blocker; CKD: chronic kidney disease; HFpEF: heart failure with preserved ejection fraction; mEq/L: milliequivalents per litre; MRA: mineralocorticoid receptor antagonist; RAASi: renin-angiotensin-aldosterone system inhibitors; PY: patient years; sK+: serum potassium.

Figure S5. Impact of sub-optimal RAASi dosing/ discontinuation on mortality

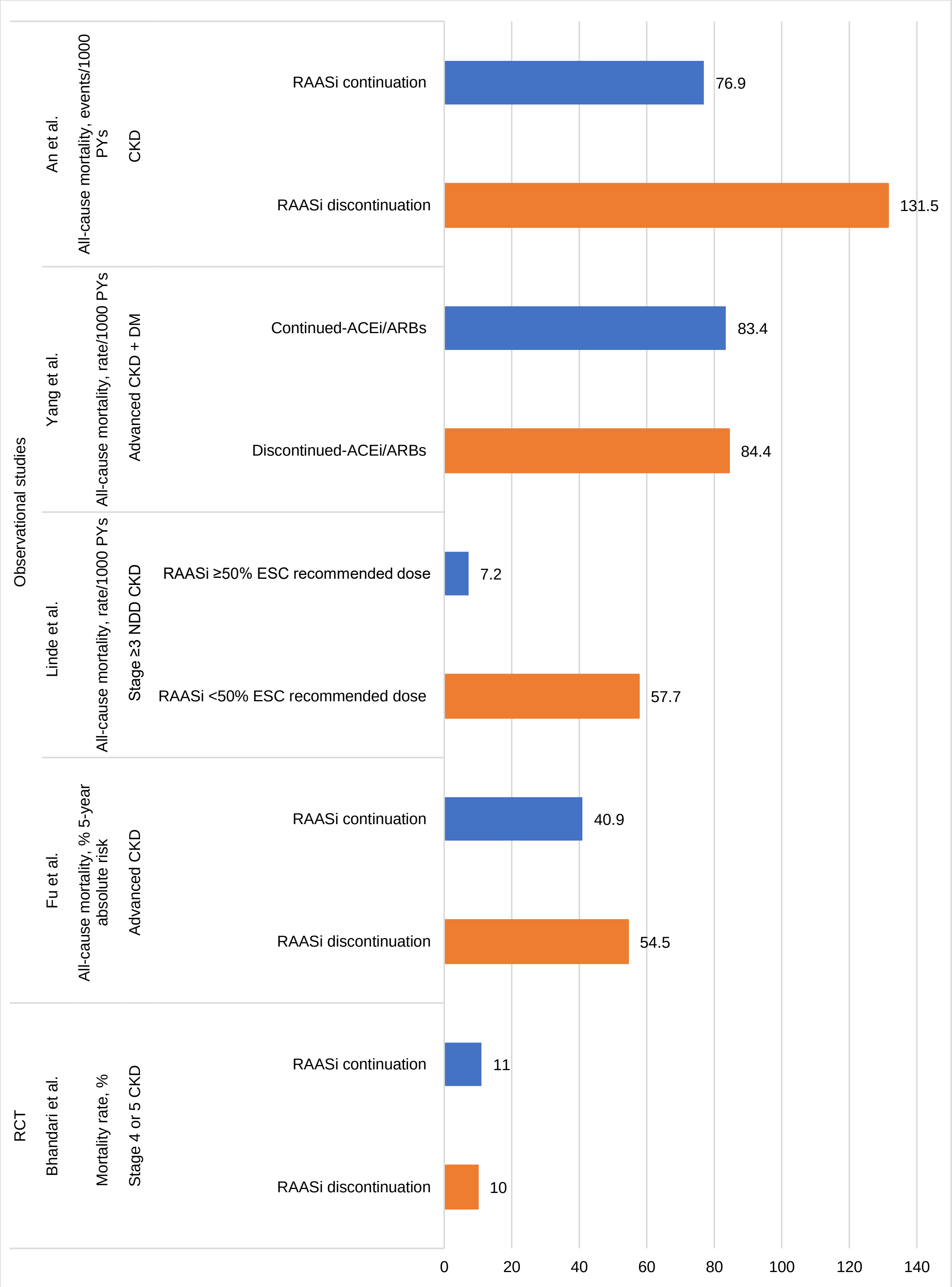

Abbreviations: ACEi: angiotensin converting enzyme inhibitors; ARB: angiotensin receptor blocker; CKD: chronic kidney disease; DM: diabetes mellitus; ESC: European Society of Cardiology; NDD: non-dialysis dependent; PYs: patient years; RCT: randomised controlled trial; RAASi: renin-angiotensin-aldosterone system inhibitors.

Figure S6. Impact of sub-optimal RAASi dosing/ discontinuation on cardiorenal outcomes

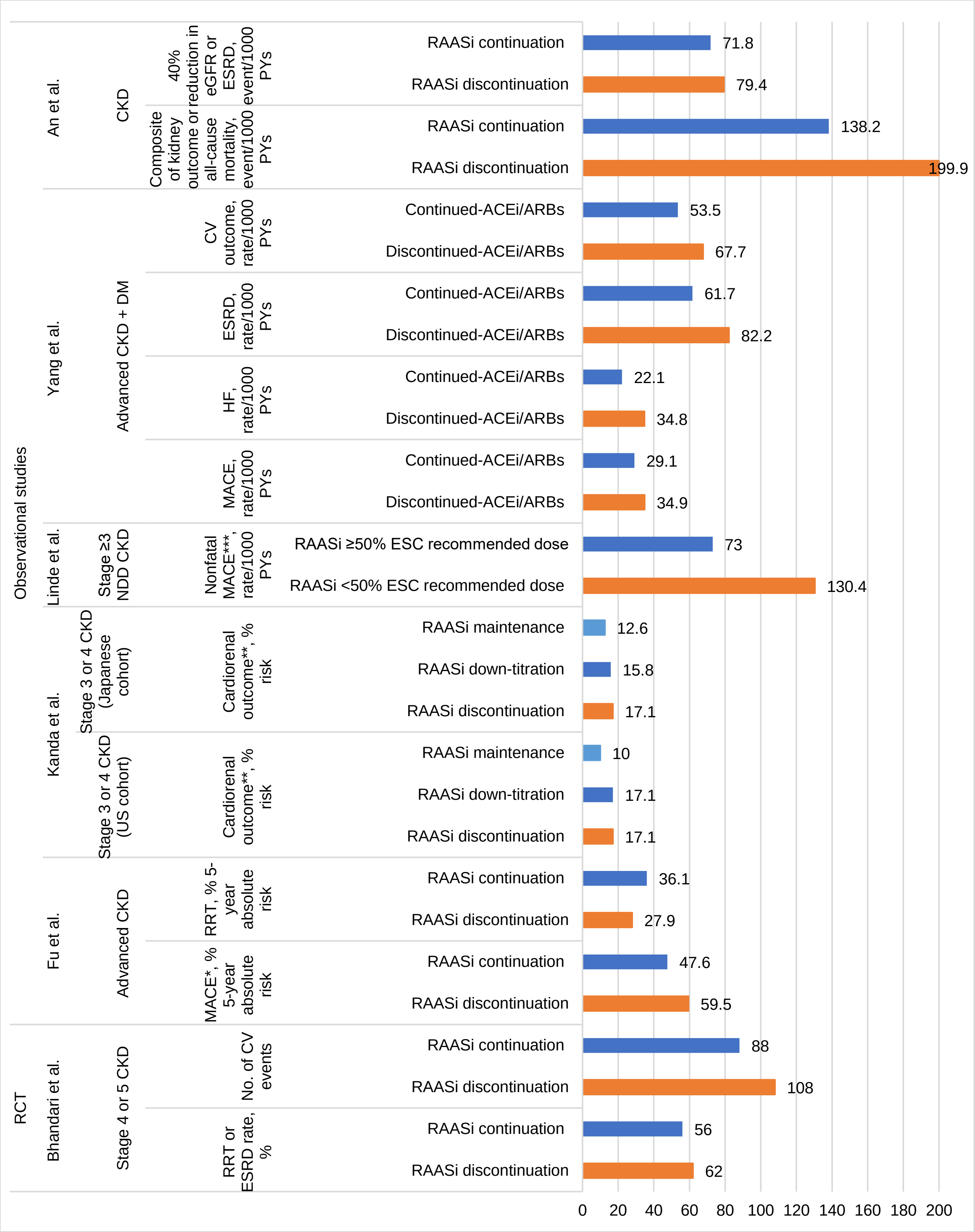

Notes: *Composite of mortality, MI, and cerebrovascular events; **composite of HF emergency visit, HF hospitalisation, or progression to ESRD at 180 days of follow up; ***Composite of arrhythmia, HF, MI, and stroke.

Abbreviations: ACEi: angiotensin converting enzyme inhibitors; ARB: angiotensin receptor blocker; CKD: chronic kidney disease; CV: cardiovascular; DM: diabetes mellitus; eGFR: estimated glomerular filtration rate; ESC: European Society of Cardiology; ESRD: end-stage renal disease; HF: heart failure; MACE: major adverse cardiovascular events; MI: myocardial infarction; NDD: non-dialysis dependent; PYs: patient years; RAASi: renin-angiotensin-aldosterone system inhibitors; RCT: randomised controlled trial; RRT: renal replacement therapy; US: United States

Figure S7. Impact of sub-optimal RAASi dosing on inpatient hospitalisations

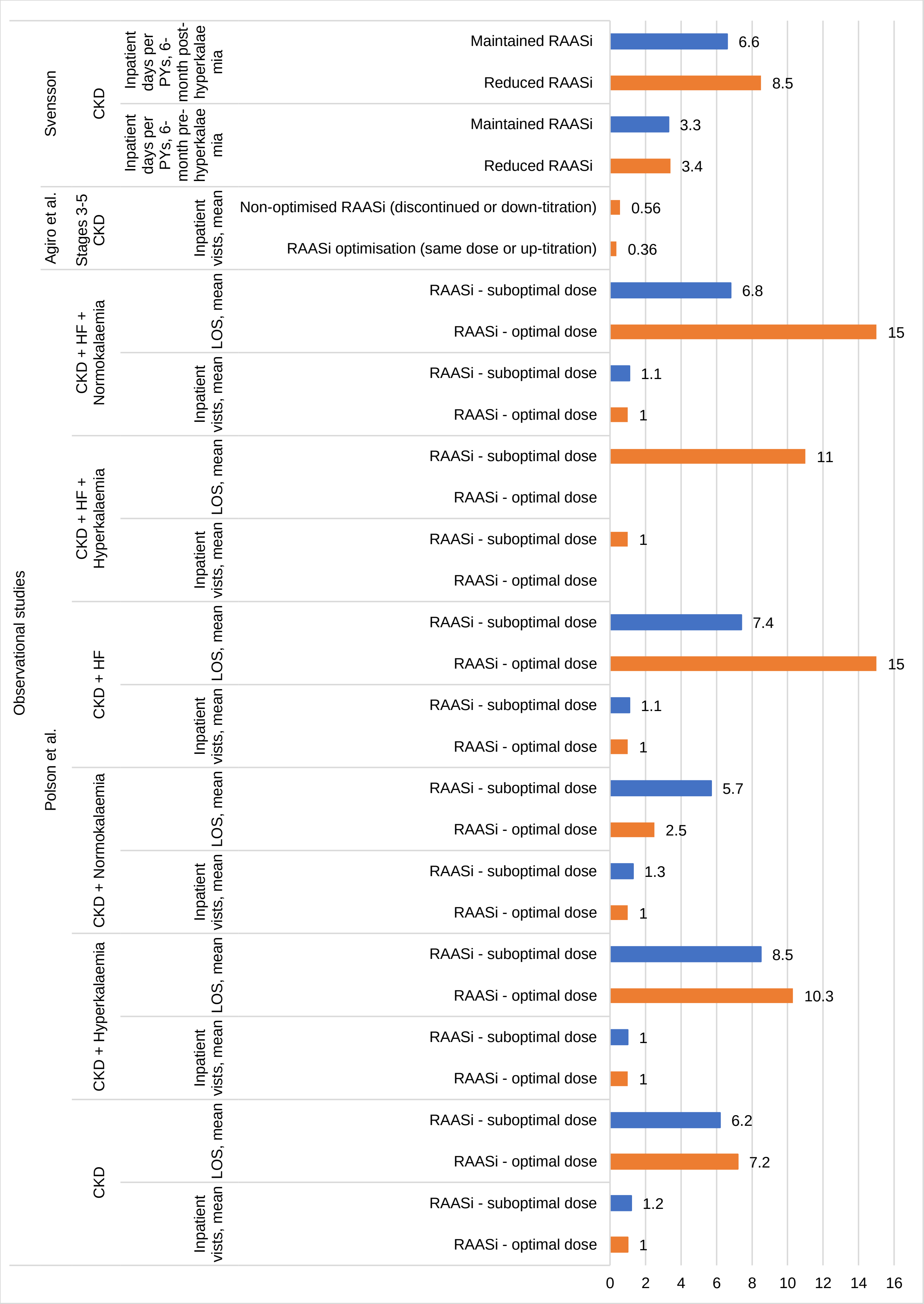

Abbreviations: CKD: chronic kidney disease; HF: heart failure; LOS: Length of stay; RAASi: renin-angiotensin-aldosterone system inhibitors

Figure S8. Impact of sub-optimal RAASi dosing on healthcare costs

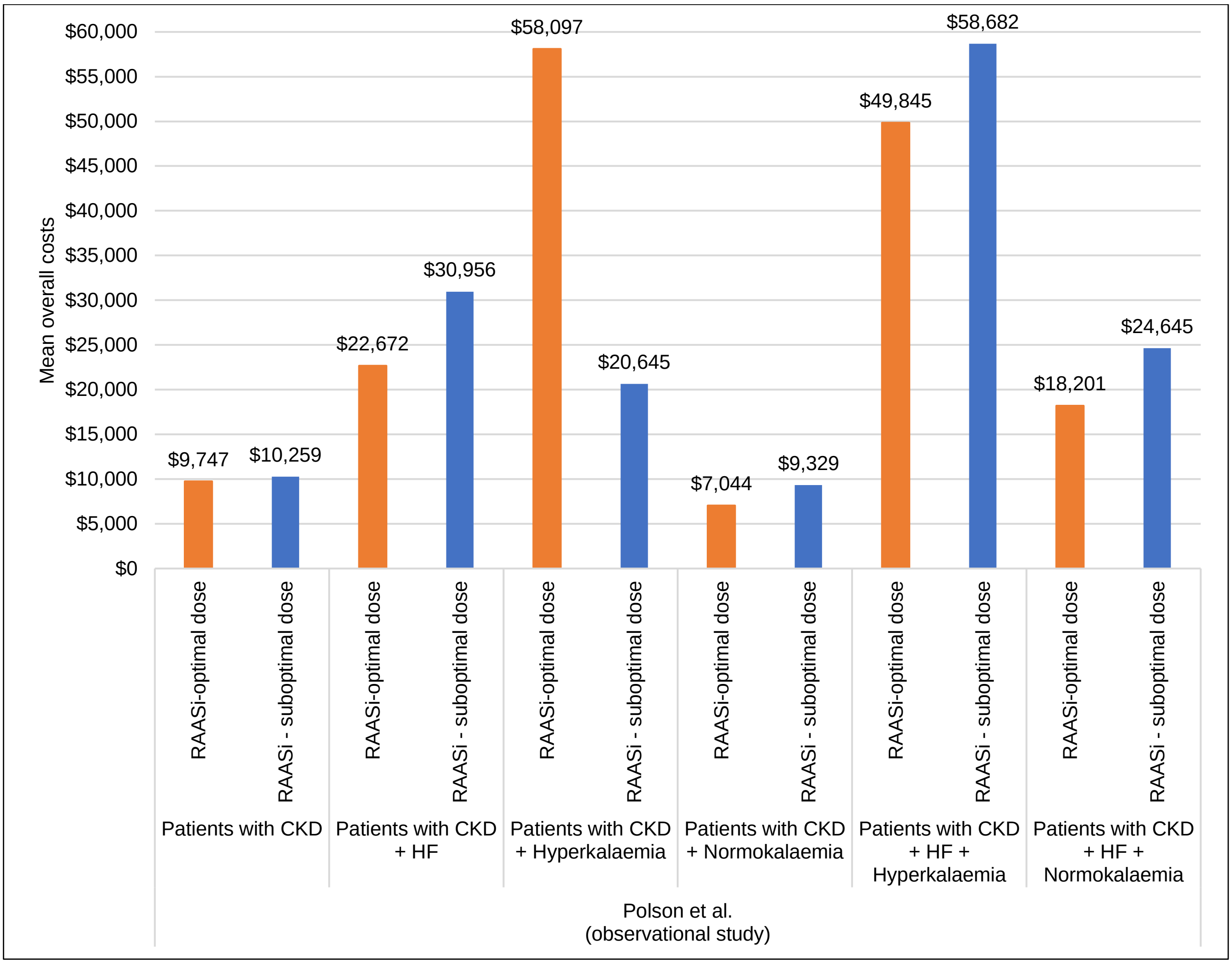

Abbreviations: CKD: chronic kidney disease; HF: heart failure; RAASi: renin-angiotensin-aldosterone system inhibitors.

Figure S9. Downs and Black quality assessment summary across non-comparative non-randomised/ observational studies

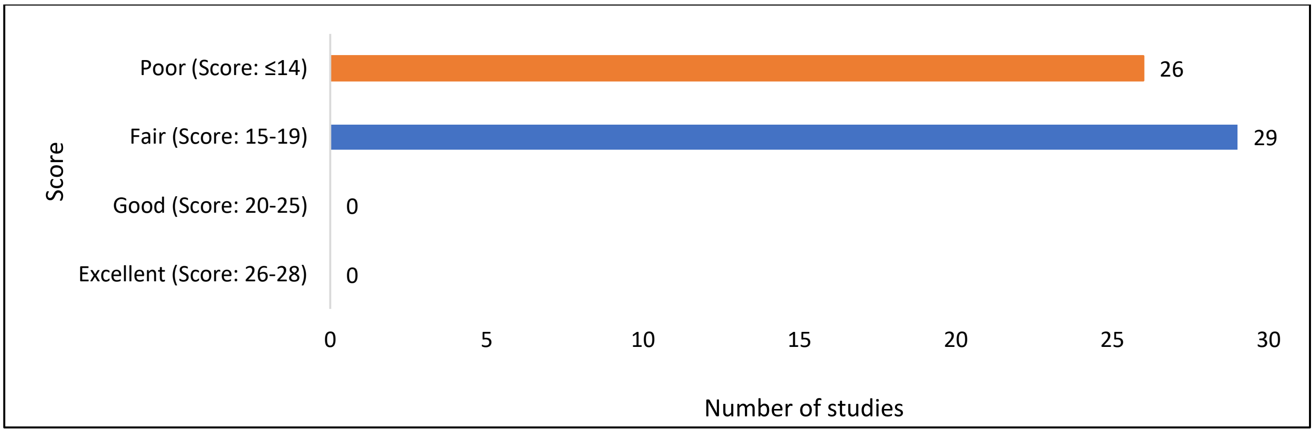

Figure S10. Downs and Black quality assessment summary across comparative across non-randomised/ observational studies

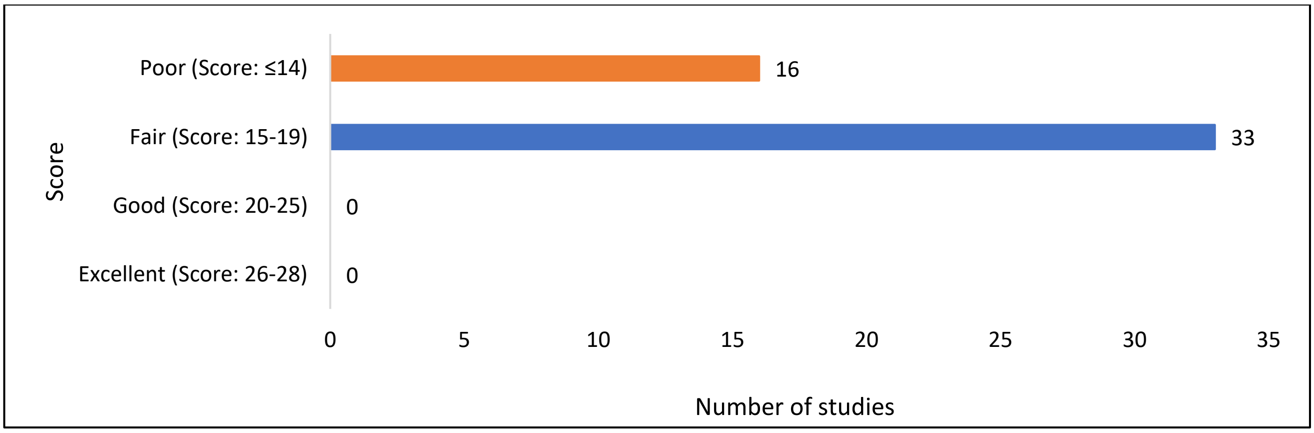

Supplement: sfaf127_Supplemental_Files [file sfaf127_supplemental_files.zip › Supp2_Results_Figures_S3-S10.pdf]
