# Supplementary material for: The burden of hyperkalaemia in chronic kidney disease: a systematic literature review
Source: Clin Kidney J. 2025 Apr 29;18(5):sfaf127. doi: 10.1093/ckj/sfaf127 (PMC12082095; doi:10.1093/ckj/sfaf127)
Supplement: sfaf127_Supplemental_Files [file sfaf127_supplemental_files.zip › Supp3_Search & DE Items_Tables_S1-S10.docx]

**Supplementary Data**

[Table S1. Epidemiology and burden of illness search strategy (Embase^®^) - original SLR](#TS1)

[Table S2. Epidemiology and burden of illness search strategy (Embase^®^) - SLR update](#TS2)

[Table S3. Epidemiology and burden of illness search strategy (Ovid Medline^®^) - original SLR](#TS3)

[Table S4. Epidemiology and burden of illness search strategy (Ovid Medline^®^) - SLR update](#TS4)

[Table S5. Suboptimal dosing search strategy (Embase^®^) - original SLR](#TS5)

[Table S6. Suboptimal dosing search strategy (Embase^®^) - SLR update](#TS6)

[Table S7. Suboptimal dosing search strategy (Ovid Medline^®^) – original SLR](#TS7)

[Table S8. Suboptimal dosing search strategy (Ovid Medline^®^) – SLR update](#TS8)

[Table S9. List of conference proceedings searched](#TS9)

[Table S10. Data extraction items](#S10)

Table S1. Epidemiology and burden of illness search strategy (Embase^®^) - original SLR

| **#** | **Searches** | **Results** |
| --- | --- | --- |
| **Database(s): Embase 1974 to 2023 April 19. Search date: 20 April 2023** | | |
| **Disease terms** | | |
| 1 | chronic kidney disease/ | 110756 |
| 2 | chronic kidney failure/ | 142624 |
| 3 | (kidney failure/ or kidney disease/) and chronic.ti,ab. | 48344 |
| 4 | ((chronic or progressive) adj2 (renal or kidney)).ti,ab. | 170909 |
| 5 | (chronic adj (kidney or renal) adj insufficienc*).ti,ab. | 6787 |
| 6 | CKD.ti,ab. | 79407 |
| 7 | diabetic nephropathy/ | 52750 |
| 8 | exp glomerulonephritis/ | 66588 |
| 9 | exp proteinuria/ | 124550 |
| 10 | kidney tubule acidosis/ | 4605 |
| 11 | renovascular hypertension/ | 15304 |
| 12 | (diabetic adj (kidney or renal) adj (disease* or failure)).ti,ab. | 7842 |
| 13 | ((renal or renovascular) adj2 hypertensi*).ti,ab. | 18192 |
| 14 | (glomerulosclerosis or glomerulonephritis or nephropath* or proteinuria* or albuminuria or microalbuminuria).ti,ab. | 198808 |
| 15 | (glomerular adj (sclerosis or nephritis)).ti,ab. | 3081 |
| 16 | ((renal or distal or proximal or tubul*) adj2 acidos*).ti,ab. | 4455 |
| 17 | hyperuricemia/ or hyperuric?emi*.ti,ab. | 24122 |
| 18 | secondary hyperparathyroidism/ or renal osteodystrophy/ | 13570 |
| 19 | (renal adj2 (osteo* or hyperparathyroidism)).ti,ab. | 4970 |
| 20 | or/1-19 | 543835 |
| 21 | obstructive uropathy/ | 3656 |
| 22 | exp urinary tract obstruction/ | 31332 |
| 23 | ((uropath* or ureter* or urethra*) adj obstruct*).ti,ab. | 11232 |
| 24 | (renal or chronic or kidney).ti,ab. | 3039607 |
| 25 | (21 or 22 or 23) and 24 | 18515 |
| 26 | 20 or 25 | 557573 |
| 27 | (transplant* or donor* or graft* or allograft*).ti. | 646237 |
| 28 | pregnan*.ti. | 303459 |
| 29 | *hemodialysis/ not (predialysis or pre dialysis or ("not" adj4 dialysis)).ti. | 60093 |
| 30 | 26 not (27 or 28 or 29) | 497608 |
| 31 | *hyperkalemia/ or *hyperkalaemia/ | 5942 |
| 32 | (hyperkalemi$ or hyperkalaemi$).tw. | 15079 |
| 33 | (hyperpotassemi$ or hyperpotassaemi$).tw. | 171 |
| 34 | *hypokalemia/ or *hypokalaemia/ | 6579 |
| 35 | (hypokalemi$ or hypokalaemi$).tw. | 19317 |
| 36 | (normokalaemi$ or normokalemi$).tw. | 1031 |
| 37 | ((maintenance or maintain$ or achiev$ or increas$ or elevat$ or reduc$ or lower$ or decreas$ or abnormal$ or normal$ or high$) and potassium).tw. | 137159 |
| 38 | or/31-37 | 162224 |
| 39 | 30 and 38 | 10806 |
| **Epidemiology terms** | | |
| 40 | prevalence/ | 933573 |
| 41 | exp incidence/ | 671296 |
| 42 | exp *epidemiology/ | 581542 |
| 43 | (incidence$ or prevalence$ or epidemiolog$ or mortalit$ or die or fatal or death$ or morbid$ or co-morbid$ or comorbid$ or (time adj3 (mortality or death* or die*))).ti,ab. or (ESRD or end stage renal disease or MI or myocardial infarction or non-fatal MI or non-fatal myocardial infarction or stroke or non-fatal stroke).ti,ab,kw. | 5946672 |
| 44 | *risk factor/ or (risk factor or risk factors).ti,ab. | 1139821 |
| 45 | or/40-44 | 6838319 |
| **Humanistic burden terms** | | |
| 46 | quality of life.ti,ab,kw. | 594899 |
| 47 | ((instrument or instruments) adj3 quality of life).ti,ab,kw. | 6148 |
| 48 | Quality-Adjusted Life Year/ | 34979 |
| 49 | quality adjusted life.ti,ab,kw. | 26343 |
| 50 | (qaly$ or qald$ or qale$ or qtime$ or life year or life years).ti,ab,kw. | 44232 |
| 51 | disability adjusted life.ti,ab,kw. | 6247 |
| 52 | daly$.ti,ab,kw. | 6093 |
| 53 | (sf36 or sf 36 or short form 36 or shortform 36 or short form36 or shortform36 or sf thirtysix or sfthirtysix or sfthirty six or sf thirty six or shortform thirtysix or shortform thirty six or short form thirtysix or short form thirty six).ti,ab,kw. | 49846 |
| 54 | (hql or hqol or h qol or hrqol or hr qol).ti,ab,kw. | 38788 |
| 55 | (health$ adj2 year$ adj2 equivalent$).ti,ab,kw. | 53 |
| 56 | (quality of wellbeing or quality of well being or index of wellbeing or index of well being or qwb).ti,ab,kw. | 879 |
| 57 | (euro qual or euro qual5d or euro qol5d or eq-5d or eq5-d or eq5d or euroqual or euroqol or euroqual5d or euroqol5d or eq-5d).ti,ab,kw. | 29741 |
| 58 | (health adj3 (utilit$ or status)).ti,ab,kw. | 117092 |
| 59 | (utilit$ adj3 (valu$ or measur$ or health or life or estimat$ or elicit$ or disease or score$ or weight)).ti,ab,kw. | 25295 |
| 60 | (preference$ adj3 (valu$ or measur$ or health or life or estimat$ or elicit$ or disease or score$ or instrument or instruments)).ti,ab,kw. | 18904 |
| 61 | disutilit$.ti,ab,kw. | 1229 |
| 62 | (time trade-off or time tradeoff or tto or standard gamble).ti,ab. | 4095 |
| 63 | exp "functional assessment of chronic illness therapy fatigue scale"/ | 1255 |
| 64 | (FACIT fatigue or FACIT* or Functional assessment of chronic illness therapy).ti,ab,kw. | 3953 |
| 65 | (KDQOL or Kidney Disease Quality of Life-36 or KDQOL-36 or Kidney Disease Quality of Life Short Form or KDQOL-SF).ti,ab,kw. | 1063 |
| 66 | or/46-65 | 755442 |
| **Economic burden terms** | | |
| 67 | exp pharmacoeconomics/ | 228916 |
| 68 | exp socioeconomics/ | 1292576 |
| 69 | exp health economics/ | 1017788 |
| 70 | cost/ | 62301 |
| 71 | exp economic aspect/ | 2419056 |
| 72 | (economic$ or cost or costs or costly or costing or price or prices or pricing or pharmacoeconomic$ or pharmaco-economic$ or expense or expenses or financial or finance or financed).ti,ab,kw. | 1489177 |
| 73 | exp economics/ | 248985 |
| 74 | exp economic evaluation/ | 351568 |
| 75 | cost utility analysis/ | 12162 |
| 76 | (cost$ adj2 (effective$ or utilit$ or analys$ or benefit$ or minimi$ or outcome or outcomes)).ti,ab,kw. | 299495 |
| 77 | cost of illness.ab,ti,kw. | 4658 |
| 78 | "cost of illness"/ | 21139 |
| 79 | cost minimization analysis/ | 3971 |
| 80 | cost effectiveness analysis/ | 179298 |
| 81 | cost benefit analysis/ | 93660 |
| 82 | (cba or cea or cua or cma or cca).ti,ab,kw. | 79874 |
| 83 | cost control/ | 75822 |
| 84 | exp budget/ | 33567 |
| 85 | budget$.ti,ab,kw. | 46917 |
| 86 | markov$.ti,ab,kw. | 40626 |
| 87 | monte carlo.ti,ab,kw. | 61183 |
| 88 | (decision$ adj2 (tree$ or analys$ or model$)).ti,ab,kw. | 48046 |
| 89 | exp Monte Carlo method/ | 51511 |
| 90 | exp "decision tree"/ | 21098 |
| 91 | microsimulation.ti,ab,kw. | 2675 |
| 92 | discrete event simulation.ti,ab,kw. | 1440 |
| 93 | patient level simulation.ti,ab,kw. | 244 |
| 94 | simulation/ | 248708 |
| 95 | (expenditure$ not energy).ti,ab,kw. | 50128 |
| 96 | (value adj1 money).ti,ab,kw. | 45 |
| 97 | exp "health care cost"/ | 335285 |
| 98 | hospitalization cost/ | 9826 |
| 99 | nursing cost/ | 205 |
| 100 | "drug cost"/ | 85204 |
| 101 | exp resource allocation/ | 24672 |
| 102 | exp resource management/ | 45607 |
| 103 | ((health care or resourc$ or service$ or hospital$) adj2 (utili$ or us$)).ti,ab,kw. | 223240 |
| 104 | "length of stay"/ | 263296 |
| 105 | ((length or duration or extended or prolonged) adj stay).ti,ab,kw. | 2188 |
| 106 | health care utilization/ | 92559 |
| 107 | exp Absenteeism/ | 19776 |
| 108 | absenteeism.ti,ab,kw. | 10632 |
| 109 | sick leave.ti,ab,kw. | 8265 |
| 110 | exp Unemployment/ | 27052 |
| 111 | exp Employment/ | 123750 |
| 112 | exp work capacity/ | 13602 |
| 113 | exp employment status/ | 46250 |
| 114 | exp work disability/ | 5740 |
| 115 | (employment or unemployment or unemployed or employability or employable).ti,ab,kw. | 119300 |
| 116 | (work capacit$ or work status or work activit$).ti,ab,kw. | 11814 |
| 117 | or/67-116 | 4067582 |
| 118 | 45 or 66 or 117 | 10137802 |
| 119 | 39 and 118 | 4390 |
| 120 | limit 119 to english language | 4143 |
| 121 | (((animal$ not human$).mp. or animal/) not (animal/ and human/)) or animal/ or animal experiment/ or animal model/ or animal tissue/ or nonhuman/ | 9553461 |
| 122 | (news or comment or editorial or note or case reports or letter).pt. | 3006183 |
| 123 | 121 or 122 | 12269116 |
| 124 | 120 not 123 | 3646 |
| **Limits** | | |
| 125 | limit 124 to yr="2000 -Current" | 3430 |
| 126 | conference.so. | 649334 |
| 127 | conference abstract.pt. | 4735417 |
| 128 | 126 or 127 | 4749093 |
| 129 | limit 128 to yr="2000 - 2020" | 4133174 |
| 130 | 125 not 129 | 2205 |

Table S2. Epidemiology and burden of illness search strategy (Embase®) - SLR update

| **#** | **Searches** | **Results** |
| --- | --- | --- |
| **Database(s): Embase 1974 to 2024 April 05. Search date: 08 April 2024** | | |
| **Disease terms** | | |
| 1 | chronic kidney disease/ | 112517 |
| 2 | chronic kidney failure/ | 158745 |
| 3 | (kidney failure/ or kidney disease/) and chronic.ti,ab. | 51948 |
| 4 | ((chronic or progressive) adj2 (renal or kidney)).ti,ab. | 183298 |
| 5 | (chronic adj (kidney or renal) adj insufficienc*).ti,ab. | 6943 |
| 6 | CKD.ti,ab. | 88421 |
| 7 | diabetic nephropathy/ | 56386 |
| 8 | exp glomerulonephritis/ | 70899 |
| 9 | exp proteinuria/ | 133471 |
| 10 | kidney tubule acidosis/ | 4800 |
| 11 | renovascular hypertension/ | 15408 |
| 12 | (diabetic adj (kidney or renal) adj (disease* or failure)).ti,ab. | 8939 |
| 13 | ((renal or renovascular) adj2 hypertensi*).ti,ab. | 18594 |
| 14 | (glomerulosclerosis or glomerulonephritis or nephropath* or proteinuria* or albuminuria or microalbuminuria).ti,ab. | 209778 |
| 15 | (glomerular adj (sclerosis or nephritis)).ti,ab. | 3230 |
| 16 | ((renal or distal or proximal or tubul*) adj2 acidos*).ti,ab. | 4669 |
| 17 | hyperuricemia/ or hyperuric?emi*.ti,ab. | 25806 |
| 18 | secondary hyperparathyroidism/ or renal osteodystrophy/ | 13984 |
| 19 | (renal adj2 (osteo* or hyperparathyroidism)).ti,ab. | 5099 |
| 20 | or/1-19 | 579959 |
| 21 | obstructive uropathy/ | 3920 |
| 22 | exp urinary tract obstruction/ | 32725 |
| 23 | ((uropath* or ureter* or urethra*) adj obstruct*).ti,ab. | 11685 |
| 24 | (renal or chronic or kidney).ti,ab. | 3169839 |
| 25 | (21 or 22 or 23) and 24 | 19507 |
| 26 | 20 or 25 | 594278 |
| 27 | (transplant* or donor* or graft* or allograft*).ti. | 666830 |
| 28 | pregnan*.ti. | 314837 |
| 29 | *hemodialysis/ not (predialysis or pre dialysis or ("not" adj4 dialysis)).ti. | 65004 |
| 30 | 26 not (27 or 28 or 29) | 530172 |
| 31 | *hyperkalemia/ or *hyperkalaemia/ | 6178 |
| 32 | (hyperkalemi$ or hyperkalaemi$).tw. | 16206 |
| 33 | (hyperpotassemi$ or hyperpotassaemi$).tw. | 181 |
| 34 | *hypokalemia/ or *hypokalaemia/ | 6729 |
| 35 | (hypokalemi$ or hypokalaemi$).tw. | 20605 |
| 36 | (normokalaemi$ or normokalemi$).tw. | 1090 |
| 37 | ((maintenance or maintain$ or achiev$ or increas$ or elevat$ or reduc$ or lower$ or decreas$ or abnormal$ or normal$ or high$) and potassium).tw. | 142623 |
| 38 | or/31-37 | 169099 |
| 39 | 30 and 38 | 11702 |
| **Epidemiology terms** | | |
| 40 | prevalence/ | 994227 |
| 41 | exp incidence/ | 713950 |
| 42 | exp *epidemiology/ | 603338 |
| 43 | (incidence$ or prevalence$ or epidemiolog$ or mortalit$ or die or fatal or death$ or morbid$ or co-morbid$ or comorbid$ or (time adj3 (mortality or death* or die*))).ti,ab. or (ESRD or end stage renal disease or MI or myocardial infarction or non-fatal MI or non-fatal myocardial infarction or stroke or non-fatal stroke).ti,ab,kw. | 6225963 |
| 44 | *risk factor/ or (risk factor or risk factors).ti,ab. | 1201822 |
| 45 | or/40-44 | 7169381 |
| **Humanistic burden terms** | | |
| 46 | quality of life.ti,ab,kw. | 634034 |
| 47 | ((instrument or instruments) adj3 quality of life).ti,ab,kw. | 6339 |
| 48 | Quality-Adjusted Life Year/ | 37072 |
| 49 | quality adjusted life.ti,ab,kw. | 27669 |
| 50 | (qaly$ or qald$ or qale$ or qtime$ or life year or life years).ti,ab,kw. | 46919 |
| 51 | disability adjusted life.ti,ab,kw. | 7081 |
| 52 | daly$.ti,ab,kw. | 6936 |
| 53 | (sf36 or sf 36 or short form 36 or shortform 36 or short form36 or shortform36 or sf thirtysix or sfthirtysix or sfthirty six or sf thirty six or shortform thirtysix or shortform thirty six or short form thirtysix or short form thirty six).ti,ab,kw. | 51547 |
| 54 | (hql or hqol or h qol or hrqol or hr qol).ti,ab,kw. | 41224 |
| 55 | (health$ adj2 year$ adj2 equivalent$).ti,ab,kw. | 53 |
| 56 | (quality of wellbeing or quality of well being or index of wellbeing or index of well being or qwb).ti,ab,kw. | 915 |
| 57 | (euro qual or euro qual5d or euro qol5d or eq-5d or eq5-d or eq5d or euroqual or euroqol or euroqual5d or euroqol5d or eq-5d).ti,ab,kw. | 32049 |
| 58 | (health adj3 (utilit$ or status)).ti,ab,kw. | 123674 |
| 59 | (utilit$ adj3 (valu$ or measur$ or health or life or estimat$ or elicit$ or disease or score$ or weight)).ti,ab,kw. | 26705 |
| 60 | (preference$ adj3 (valu$ or measur$ or health or life or estimat$ or elicit$ or disease or score$ or instrument or instruments)).ti,ab,kw. | 20039 |
| 61 | disutilit$.ti,ab,kw. | 1340 |
| 62 | (time trade-off or time tradeoff or tto or standard gamble).ti,ab. | 4281 |
| 63 | exp "functional assessment of chronic illness therapy fatigue scale"/ | 1525 |
| 64 | (FACIT fatigue or FACIT* or Functional assessment of chronic illness therapy).ti,ab,kw. | 4319 |
| 65 | (KDQOL or Kidney Disease Quality of Life-36 or KDQOL-36 or Kidney Disease Quality of Life Short Form or KDQOL-SF).ti,ab,kw. | 1179 |
| 66 | or/46-65 | 804267 |
| **Economic burden terms** | | |
| 67 | exp pharmacoeconomics/ | 240315 |
| 68 | exp socioeconomics/ | 1409728 |
| 69 | exp health economics/ | 1066134 |
| 70 | cost/ | 64271 |
| 71 | exp economic aspect/ | 2584360 |
| 72 | (economic$ or cost or costs or costly or costing or price or prices or pricing or pharmacoeconomic$ or pharmaco-economic$ or expense or expenses or financial or finance or financed).ti,ab,kw. | 1580702 |
| 73 | exp economics/ | 250336 |
| 74 | exp economic evaluation/ | 366043 |
| 75 | cost utility analysis/ | 12843 |
| 76 | (cost$ adj2 (effective$ or utilit$ or analys$ or benefit$ or minimi$ or outcome or outcomes)).ti,ab,kw. | 316548 |
| 77 | cost of illness.ab,ti,kw. | 4853 |
| 78 | "cost of illness"/ | 21609 |
| 79 | cost minimization analysis/ | 4111 |
| 80 | cost effectiveness analysis/ | 188954 |
| 81 | cost benefit analysis/ | 96581 |
| 82 | (cba or cea or cua or cma or cca).ti,ab,kw. | 83287 |
| 83 | cost control/ | 77927 |
| 84 | exp budget/ | 34481 |
| 85 | budget$.ti,ab,kw. | 49202 |
| 86 | markov$.ti,ab,kw. | 42752 |
| 87 | monte carlo.ti,ab,kw. | 64236 |
| 88 | (decision$ adj2 (tree$ or analys$ or model$)).ti,ab,kw. | 53365 |
| 89 | exp Monte Carlo method/ | 54809 |
| 90 | exp "decision tree"/ | 23965 |
| 91 | microsimulation.ti,ab,kw. | 2886 |
| 92 | discrete event simulation.ti,ab,kw. | 1523 |
| 93 | patient level simulation.ti,ab,kw. | 266 |
| 94 | simulation/ | 266335 |
| 95 | (expenditure$ not energy).ti,ab,kw. | 52300 |
| 96 | (value adj1 money).ti,ab,kw. | 45 |
| 97 | exp "health care cost"/ | 350959 |
| 98 | hospitalization cost/ | 10725 |
| 99 | nursing cost/ | 214 |
| 100 | "drug cost"/ | 88147 |
| 101 | exp resource allocation/ | 25659 |
| 102 | exp resource management/ | 47495 |
| 103 | ((health care or resourc$ or service$ or hospital$) adj2 (utili$ or us$)).ti,ab,kw. | 235979 |
| 104 | "length of stay"/ | 285660 |
| 105 | ((length or duration or extended or prolonged) adj stay).ti,ab,kw. | 2339 |
| 106 | health care utilization/ | 99523 |
| 107 | exp Absenteeism/ | 20345 |
| 108 | absenteeism.ti,ab,kw. | 11150 |
| 109 | sick leave.ti,ab,kw. | 8554 |
| 110 | exp Unemployment/ | 28678 |
| 111 | exp Employment/ | 132299 |
| 112 | exp work capacity/ | 14073 |
| 113 | exp employment status/ | 50195 |
| 114 | exp work disability/ | 5853 |
| 115 | (employment or unemployment or unemployed or employability or employable).ti,ab,kw. | 128862 |
| 116 | (work capacit$ or work status or work activit$).ti,ab,kw. | 12242 |
| 117 | or/67-116 | 4341263 |
| 118 | 45 or 66 or 117 | 10669946 |
| 119 | 39 and 118 | 4870 |
| **Limits** | | |
| 120 | limit 119 to english language | 4607 |
| 121 | (((animal$ not human$).mp. or animal/) not (animal/ and human/)) or animal/ or animal experiment/ or animal model/ or animal tissue/ or nonhuman/ | 9844249 |
| 122 | (news or comment or editorial or note or case reports or letter).pt. | 3096097 |
| 123 | 121 or 122 | 12640168 |
| 124 | 120 not 123 | 4064 |
| 125 | limit 124 to yr="2023 -Current" | 516 |
| 126 | conference.so. | 698451 |
| 127 | conference abstract.pt. | 5101169 |
| 128 | 126 or 127 | 5115068 |
| 129 | limit 128 to yr="2000-2022" | 4772370 |
| 130 | 125 not 129 | 516 |

Table S3. Epidemiology and burden of illness search strategy (Ovid Medline^®^) - original SLR

| **#** | **Searches** | **Results** |
| --- | --- | --- |
| **Database(s): Ovid MEDLINE(R) and Epub Ahead of Print, In-Process, In-Data-Review & Other Non-Indexed Citations and Daily 1946 to April 19, 2023. Search date: 20 April 2023** | | |
| **Disease terms** | | |
| 1 | renal insufficiency, chronic/ | 35079 |
| 2 | exp kidney failure, chronic/ | 100606 |
| 3 | kidney diseases/ and chronic.ti,ab. | 13143 |
| 4 | ((chronic or progressive) adj2 (renal or kidney)).ti,ab. | 108665 |
| 5 | (chronic adj (kidney or renal) adj insufficienc*).ti,ab. | 5311 |
| 6 | CKD.ti,ab. | 41230 |
| 7 | diabetic nephropathies/ | 29238 |
| 8 | exp glomerulonephritis/ | 51754 |
| 9 | exp proteinuria/ | 42267 |
| 10 | acidosis, renal tubular/ | 2912 |
| 11 | exp hypertension, renal/ | 19712 |
| 12 | (diabetic adj (kidney or renal) adj (disease* or failure)).ti,ab. | 5152 |
| 13 | ((renal or renovascular) adj2 hypertensi*).ti,ab. | 14702 |
| 14 | (glomerulosclerosis or glomerulonephritis or nephropath* or proteinuria* or albuminuria or microalbuminuria).ti,ab. | 136624 |
| 15 | (glomerular adj (sclerosis or nephritis)).ti,ab. | 2257 |
| 16 | ((renal or distal or proximal or tubul*) adj2 acidos*).ti,ab. | 3536 |
| 17 | hyperuricemia/ or hyperuric?emi*.ti,ab. | 11539 |
| 18 | exp hyperparathyroidism, secondary/ | 8998 |
| 19 | (renal adj2 (osteo* or hyperparathyroidism)).ti,ab. | 3822 |
| 20 | or/1-19 | 375761 |
| 21 | ureteral obstruction/ | 14580 |
| 22 | exp urethral obstruction/ | 12016 |
| 23 | ((uropath* or ureter* or urethra*) adj obstruct*).ti,ab. | 8305 |
| 24 | (renal of kidney or chronic).ti,ab. | 1362024 |
| 25 | (21 or 22 or 23) and 24 | 2119 |
| 26 | 20 or 25 | 376638 |
| 27 | (transplant* or donor* or graft* or allograft*).ti. | 471778 |
| 28 | pregnan*.ti. | 262161 |
| 29 | *renal dialysis/ not (predialysis or pre dialysis or ("not" adj4 dialysis)).ti. | 66131 |
| 30 | 26 not (27 or 28 or 29) | 314997 |
| 31 | *hyperkalemia/ or *hyperkalaemia/ | 4434 |
| 32 | (hyperkalemi$ or hyperkalaemi$).tw. | 9812 |
| 33 | (hyperpotassemi$ or hyperpotassaemi$).tw. | 176 |
| 34 | *hypokalemia/ or *hypokalaemia/ | 5466 |
| 35 | (hypokalemi$ or hypokalaemi$).tw. | 12997 |
| 36 | (normokalaemi$ or normokalemi$).tw. | 704 |
| 37 | ((maintenance or maintain$ or achiev$ or increas$ or elevat$ or reduc$ or lower$ or decreas$ or abnormal$ or normal$ or high$) and potassium).tw. | 109304 |
| 38 | or/31-37 | 127114 |
| 39 | 30 and 38 | 6326 |
| **Epidemiology terms** | | |
| 40 | prevalence/ | 341159 |
| 41 | exp incidence/ | 299525 |
| 42 | exp *epidemiology/ | 13121 |
| 43 | (incidence$ or prevalence$ or epidemiolog$ or mortalit$ or die or fatal or death$ or morbid$ or co-morbid$ or comorbid$ or (time adj3 (mortality or death* or die*))).ti,ab. or (ESRD or end stage renal disease or MI or myocardial infarction or non-fatal MI or non-fatal myocardial infarction or stroke or non-fatal stroke).ti,ab,kw. | 4132858 |
| 44 | *risk factor/ or (risk factor or risk factors).ti,ab. | 740864 |
| 45 | or/40-44 | 4603243 |
| **Humanistic burden terms** | | |
| 46 | quality of life.ti,ab,kw. | 369989 |
| 47 | ((instrument or instruments) adj3 quality of life).ti,ab,kw. | 4394 |
| 48 | Quality-Adjusted Life Year/ | 15553 |
| 49 | quality adjusted life.ti,ab,kw. | 16856 |
| 50 | (qaly$ or qald$ or qale$ or qtime$ or life year or life years).ti,ab,kw. | 27314 |
| 51 | disability adjusted life.ti,ab,kw. | 5052 |
| 52 | daly$.ti,ab,kw. | 4630 |
| 53 | (sf36 or sf 36 or short form 36 or shortform 36 or short form36 or shortform36 or sf thirtysix or sfthirtysix or sfthirty six or sf thirty six or shortform thirtysix or shortform thirty six or short form thirtysix or short form thirty six).ti,ab,kw. | 30230 |
| 54 | (hql or hqol or h qol or hrqol or hr qol).ti,ab,kw. | 23389 |
| 55 | (health$ adj2 year$ adj2 equivalent$).ti,ab,kw. | 48 |
| 56 | (quality of wellbeing or quality of well being or index of wellbeing or index of well being or qwb).ti,ab,kw. | 702 |
| 57 | (euro qual or euro qual5d or euro qol5d or eq-5d or eq5-d or eq5d or euroqual or euroqol or euroqual5d or euroqol5d or eq-5d).ti,ab,kw. | 16076 |
| 58 | (health adj3 (utilit$ or status)).ti,ab,kw. | 89024 |
| 59 | (utilit$ adj3 (valu$ or measur$ or health or life or estimat$ or elicit$ or disease or score$ or weight)).ti,ab,kw. | 15582 |
| 60 | (preference$ adj3 (valu$ or measur$ or health or life or estimat$ or elicit$ or disease or score$ or instrument or instruments)).ti,ab,kw. | 14073 |
| 61 | disutilit$.ti,ab,kw. | 608 |
| 62 | (time trade-off or time tradeoff or tto or standard gamble).ti,ab. | 2787 |
| 63 | (FACIT fatigue or FACIT* or Functional assessment of chronic illness therapy).ti,ab,kw. | 1543 |
| 64 | (KDQOL or Kidney Disease Quality of Life-36 or KDQOL-36 or Kidney Disease Quality of Life Short Form or KDQOL-SF).ti,ab,kw. | 570 |
| 65 | or/46-64 | 486694 |
| **Economic burden terms** | | |
| 66 | economics/ | 27498 |
| 67 | economics, pharmaceutical/ | 3098 |
| 68 | exp economics, medical/ | 14387 |
| 69 | exp economics, hospital/ | 25697 |
| 70 | economics, nursing/ | 4013 |
| 71 | Economics, Dental/ | 1920 |
| 72 | (economic$ or cost or costs or costly or costing or price or prices or pricing or pharmacoeconomic$ or pharmaco-economic$ or expense or expenses or financial or finance or financed).ti,ab,kf. | 1152885 |
| 73 | Cost allocation/ | 2018 |
| 74 | Cost control/ | 21661 |
| 75 | Cost savings/ | 12697 |
| 76 | "Cost of Illness"/ | 31409 |
| 77 | cost of illness.ab,ti,kf. | 2734 |
| 78 | (cost$ adj2 (effective$ or utilit$ or analys$ or benefit$ or minimi$ or outcome or outcomes)).ti,ab,kf. | 218248 |
| 79 | cost-benefit analysis/ | 92157 |
| 80 | exp "Costs and Cost Analysis"/ | 263826 |
| 81 | exp "fees and charges"/ | 31339 |
| 82 | exp budgets/ | 14098 |
| 83 | Direct service costs/ | 1217 |
| 84 | Drug costs/ | 17356 |
| 85 | Health expenditures/ | 23849 |
| 86 | budget$.ti,ab,kf. | 35558 |
| 87 | exp "Fees and Charges"/ | 31339 |
| 88 | (value adj2 (money or monetary)).ti,ab,kf. | 2995 |
| 89 | models, economic/ | 11062 |
| 90 | economic model$.ti,ab,kf. | 4519 |
| 91 | markov chains/ | 15931 |
| 92 | monte carlo method/ | 32067 |
| 93 | decision tree/ | 12072 |
| 94 | exp Decision Theory/ | 13193 |
| 95 | (decision adj2 (tree$ or analys$ or model$)).ti,ab,kf. | 34176 |
| 96 | markov$.ti,ab,kf. | 32024 |
| 97 | monte carlo.ti,ab,kf. | 59363 |
| 98 | (cba or cea or cua or cma or cca).ti,ab,kf. | 54851 |
| 99 | microsimulation.ti,ab,kf. | 1766 |
| 100 | patient level simulation.ti,ab,kf. | 102 |
| 101 | discrete event simulation.ti,ab,kf. | 921 |
| 102 | simulation.ti,ab,kf. | 265883 |
| 103 | (expenditure$ not energy).ti,ab,kf. | 37002 |
| 104 | exp Health Care Costs/ | 71667 |
| 105 | exp Hospital Costs/ | 11933 |
| 106 | exp Health Resources/ | 28942 |
| 107 | exp Resource Allocation/ | 18898 |
| 108 | Health Services/ | 27497 |
| 109 | ((health care or resourc$ or service$ or hospital$) adj2 (utili$ or us$)).ti,ab,kf. | 156596 |
| 110 | ((length or duration or extended or prolonged) adj stay).ti,ab,kf. | 1240 |
| 111 | exp Drug Utilization/ | 28063 |
| 112 | "Length of Stay"/ | 101943 |
| 113 | exp Absenteeism/ | 9749 |
| 114 | absenteeism.ti,ab,kf. | 7313 |
| 115 | exp Sick Leave/ | 6718 |
| 116 | sick leave.ti,ab,kf. | 6293 |
| 117 | exp Unemployment/ | 7762 |
| 118 | unemploy$.ti,ab,kf. | 23106 |
| 119 | exp Employment/ | 99228 |
| 120 | (employment or unemployment or unemployed or employability or employable).ti,ab,kf. | 90172 |
| 121 | (work capacity or work status or work activity).ti,ab,kf. | 8145 |
| 122 | or/66-121 | 2090489 |
| 123 | 45 or 65 or 122 | 6517080 |
| 124 | 39 and 123 | 2179 |
| **Limits** | | |
| 125 | limit 124 to english language | 2012 |
| 126 | (((animal$ not human$).mp. or animal/) not (animal/ and human/)) or animal/ or animal experiment/ or animal model/ or animal tissue/ or nonhuman/ | 7376121 |
| 127 | (news or comment or editorial or note or case reports or letter).pt. | 4453385 |
| 128 | 126 or 127 | 11573174 |
| 129 | 125 not 128 | 1596 |
| 130 | limit 129 to yr="2000 -Current" | 1428 |

Table S4. Epidemiology and burden of illness search strategy (Ovid Medline^®^) - SLR update

| **#** | **Searches** | **Results** |
| --- | --- | --- |
| **Database(s): Ovid MEDLINE(R) and Epub Ahead of Print, In-Process, In-Data-Review & Other Non-Indexed Citations and Daily 1946 to April 05, 2024. Search date: 08 April 2024** | | |
| **Disease terms** | | |
| 1 | renal insufficiency, chronic/ | 38588 |
| 2 | exp kidney failure, chronic/ | 102261 |
| 3 | kidney diseases/ and chronic.ti,ab. | 13330 |
| 4 | ((chronic or progressive) adj2 (renal or kidney)).ti,ab. | 116558 |
| 5 | (chronic adj (kidney or renal) adj insufficienc*).ti,ab. | 5395 |
| 6 | CKD.ti,ab. | 45692 |
| 7 | diabetic nephropathies/ | 30347 |
| 8 | exp glomerulonephritis/ | 53076 |
| 9 | exp proteinuria/ | 43144 |
| 10 | acidosis, renal tubular/ | 2949 |
| 11 | exp hypertension, renal/ | 19798 |
| 12 | (diabetic adj (kidney or renal) adj (disease* or failure)).ti,ab. | 6021 |
| 13 | ((renal or renovascular) adj2 hypertensi*).ti,ab. | 14936 |
| 14 | (glomerulosclerosis or glomerulonephritis or nephropath* or proteinuria* or albuminuria or microalbuminuria).ti,ab. | 142114 |
| 15 | (glomerular adj (sclerosis or nephritis)).ti,ab. | 2297 |
| 16 | ((renal or distal or proximal or tubul*) adj2 acidos*).ti,ab. | 3646 |
| 17 | hyperuricemia/ or hyperuric?emi*.ti,ab. | 12355 |
| 18 | exp hyperparathyroidism, secondary/ | 9136 |
| 19 | (renal adj2 (osteo* or hyperparathyroidism)).ti,ab. | 3888 |
| 20 | or/1-19 | 391356 |
| 21 | ureteral obstruction/ | 14893 |
| 22 | exp urethral obstruction/ | 12242 |
| 23 | ((uropath* or ureter* or urethra*) adj obstruct*).ti,ab. | 8613 |
| 24 | (renal of kidney or chronic).ti,ab. | 1434188 |
| 25 | (21 or 22 or 23) and 24 | 2263 |
| 26 | 20 or 25 | 392253 |
| 27 | (transplant* or donor* or graft* or allograft*).ti. | 489331 |
| 28 | pregnan*.ti. | 273573 |
| 29 | *renal dialysis/ not (predialysis or pre dialysis or ("not" adj4 dialysis)).ti. | 66554 |
| 30 | 26 not (27 or 28 or 29) | 329642 |
| 31 | *hyperkalemia/ or *hyperkalaemia/ | 4610 |
| 32 | (hyperkalemi$ or hyperkalaemi$).tw. | 10355 |
| 33 | (hyperpotassemi$ or hyperpotassaemi$).tw. | 177 |
| 34 | *hypokalemia/ or *hypokalaemia/ | 5605 |
| 35 | (hypokalemi$ or hypokalaemi$).tw. | 13555 |
| 36 | (normokalaemi$ or normokalemi$).tw. | 728 |
| 37 | ((maintenance or maintain$ or achiev$ or increas$ or elevat$ or reduc$ or lower$ or decreas$ or abnormal$ or normal$ or high$) and potassium).tw. | 113831 |
| 38 | or/31-37 | 132342 |
| 39 | 30 and 38 | 6702 |
| **Epidemiology terms** | | |
| 40 | prevalence/ | 350801 |
| 41 | exp incidence/ | 305592 |
| 42 | exp *epidemiology/ | 13156 |
| 43 | (incidence$ or prevalence$ or epidemiolog$ or mortalit$ or die or fatal or death$ or morbid$ or co-morbid$ or comorbid$ or (time adj3 (mortality or death* or die*))).ti,ab. or (ESRD or end stage renal disease or MI or myocardial infarction or non-fatal MI or non-fatal myocardial infarction or stroke or non-fatal stroke).ti,ab,kw. | 4390195 |
| 44 | *risk factor/ or (risk factor or risk factors).ti,ab. | 795887 |
| 45 | or/40-44 | 4886535 |
| **Humanistic burden terms** | | |
| 46 | quality of life.ti,ab,kw. | 404539 |
| 47 | ((instrument or instruments) adj3 quality of life).ti,ab,kw. | 4579 |
| 48 | Quality-Adjusted Life Year/ | 16257 |
| 49 | quality adjusted life.ti,ab,kw. | 18217 |
| 50 | (qaly$ or qald$ or qale$ or qtime$ or life year or life years).ti,ab,kw. | 29855 |
| 51 | disability adjusted life.ti,ab,kw. | 5925 |
| 52 | daly$.ti,ab,kw. | 5427 |
| 53 | (sf36 or sf 36 or short form 36 or shortform 36 or short form36 or shortform36 or sf thirtysix or sfthirtysix or sfthirty six or sf thirty six or shortform thirtysix or shortform thirty six or short form thirtysix or short form thirty six).ti,ab,kw. | 31689 |
| 54 | (hql or hqol or h qol or hrqol or hr qol).ti,ab,kw. | 25555 |
| 55 | (health$ adj2 year$ adj2 equivalent$).ti,ab,kw. | 48 |
| 56 | (quality of wellbeing or quality of well being or index of wellbeing or index of well being or qwb).ti,ab,kw. | 749 |
| 57 | (euro qual or euro qual5d or euro qol5d or eq-5d or eq5-d or eq5d or euroqual or euroqol or euroqual5d or euroqol5d or eq-5d).ti,ab,kw. | 17861 |
| 58 | (health adj3 (utilit$ or status)).ti,ab,kw. | 95125 |
| 59 | (utilit$ adj3 (valu$ or measur$ or health or life or estimat$ or elicit$ or disease or score$ or weight)).ti,ab,kw. | 16790 |
| 60 | (preference$ adj3 (valu$ or measur$ or health or life or estimat$ or elicit$ or disease or score$ or instrument or instruments)).ti,ab,kw. | 15159 |
| 61 | disutilit$.ti,ab,kw. | 672 |
| 62 | (time trade-off or time tradeoff or tto or standard gamble).ti,ab. | 2930 |
| 63 | (FACIT fatigue or FACIT* or Functional assessment of chronic illness therapy).ti,ab,kw. | 1733 |
| 64 | (KDQOL or Kidney Disease Quality of Life-36 or KDQOL-36 or Kidney Disease Quality of Life Short Form or KDQOL-SF).ti,ab,kw. | 617 |
| 65 | or/46-64 | 529719 |
| **Economic burden terms** | | |
| 66 | economics/ | 27529 |
| 67 | economics, pharmaceutical/ | 3130 |
| 68 | exp economics, medical/ | 14428 |
| 69 | exp economics, hospital/ | 25809 |
| 70 | economics, nursing/ | 4013 |
| 71 | Economics, Dental/ | 1922 |
| 72 | (economic$ or cost or costs or costly or costing or price or prices or pricing or pharmacoeconomic$ or pharmaco-economic$ or expense or expenses or financial or finance or financed).ti,ab,kf. | 1244287 |
| 73 | Cost allocation/ | 2019 |
| 74 | Cost control/ | 21682 |
| 75 | Cost savings/ | 12796 |
| 76 | "Cost of Illness"/ | 32136 |
| 77 | cost of illness.ab,ti,kf. | 2965 |
| 78 | (cost$ adj2 (effective$ or utilit$ or analys$ or benefit$ or minimi$ or outcome or outcomes)).ti,ab,kf. | 236068 |
| 79 | cost-benefit analysis/ | 94347 |
| 80 | exp "Costs and Cost Analysis"/ | 269646 |
| 81 | exp "fees and charges"/ | 31427 |
| 82 | exp budgets/ | 14198 |
| 83 | Direct service costs/ | 1217 |
| 84 | Drug costs/ | 17557 |
| 85 | Health expenditures/ | 24639 |
| 86 | budget$.ti,ab,kf. | 37693 |
| 87 | exp "Fees and Charges"/ | 31427 |
| 88 | (value adj2 (money or monetary)).ti,ab,kf. | 3184 |
| 89 | models, economic/ | 11119 |
| 90 | economic model$.ti,ab,kf. | 4818 |
| 91 | markov chains/ | 16090 |
| 92 | monte carlo method/ | 32750 |
| 93 | decision tree/ | 12180 |
| 94 | exp Decision Theory/ | 13599 |
| 95 | (decision adj2 (tree$ or analys$ or model$)).ti,ab,kf. | 39436 |
| 96 | markov$.ti,ab,kf. | 34207 |
| 97 | monte carlo.ti,ab,kf. | 62720 |
| 98 | (cba or cea or cua or cma or cca).ti,ab,kf. | 57827 |
| 99 | microsimulation.ti,ab,kf. | 1970 |
| 100 | patient level simulation.ti,ab,kf. | 111 |
| 101 | discrete event simulation.ti,ab,kf. | 989 |
| 102 | simulation.ti,ab,kf. | 290727 |
| 103 | (expenditure$ not energy).ti,ab,kf. | 39223 |
| 104 | exp Health Care Costs/ | 72771 |
| 105 | exp Hospital Costs/ | 12023 |
| 106 | exp Health Resources/ | 29368 |
| 107 | exp Resource Allocation/ | 19026 |
| 108 | Health Services/ | 28164 |
| 109 | ((health care or resourc$ or service$ or hospital$) adj2 (utili$ or us$)).ti,ab,kf. | 168542 |
| 110 | ((length or duration or extended or prolonged) adj stay).ti,ab,kf. | 1322 |
| 111 | exp Drug Utilization/ | 28401 |
| 112 | "Length of Stay"/ | 104126 |
| 113 | exp Absenteeism/ | 9843 |
| 114 | absenteeism.ti,ab,kf. | 7788 |
| 115 | exp Sick Leave/ | 6898 |
| 116 | sick leave.ti,ab,kf. | 6615 |
| 117 | exp Unemployment/ | 7885 |
| 118 | unemploy$.ti,ab,kf. | 24615 |
| 119 | exp Employment/ | 102025 |
| 120 | (employment or unemployment or unemployed or employability or employable).ti,ab,kf. | 96301 |
| 121 | (work capacity or work status or work activity).ti,ab,kf. | 8434 |
| 122 | or/66-121 | 2234795 |
| 123 | 45 or 65 or 122 | 6938233 |
| 124 | 39 and 123 | 2367 |
| **Limits** | | |
| 125 | limit 124 to english language | 2196 |
| 126 | (((animal$ not human$).mp. or animal/) not (animal/ and human/)) or animal/ or animal experiment/ or animal model/ or animal tissue/ or nonhuman/ | 7532922 |
| 127 | (news or comment or editorial or note or case reports or letter).pt. | 4607171 |
| 128 | 126 or 127 | 11879920 |
| 129 | 125 not 128 | 1749 |
| 130 | limit 129 to yr="2023-Current" | 232 |

Table S5. Suboptimal dosing search strategy (Embase^®^) - original SLR

| **#** | **Searches** | **Results** |
| --- | --- | --- |
| **Database(s): Embase 1974 to 2023 April 26. Search date: 28 April 2023** | | |
| **Disease terms** | | |
| 1 | chronic kidney disease/ | 110521 |
| 2 | chronic kidney failure/ | 142882 |
| 3 | (kidney failure/ or kidney disease/) and chronic.ti,ab. | 48370 |
| 4 | ((chronic or progressive) adj2 (renal or kidney)).ti,ab. | 171048 |
| 5 | (chronic adj (kidney or renal) adj insufficienc*).ti,ab. | 6788 |
| 6 | CKD.ti,ab. | 79490 |
| 7 | diabetic nephropathy/ | 52798 |
| 8 | exp glomerulonephritis/ | 66615 |
| 9 | exp proteinuria/ | 124656 |
| 10 | kidney tubule acidosis/ | 4607 |
| 11 | renovascular hypertension/ | 15306 |
| 12 | (diabetic adj (kidney or renal) adj (disease* or failure)).ti,ab. | 7856 |
| 13 | ((renal or renovascular) adj2 hypertensi*).ti,ab. | 18201 |
| 14 | (glomerulosclerosis or glomerulonephritis or nephropath* or proteinuria* or albuminuria or microalbuminuria).ti,ab. | 198911 |
| 15 | (glomerular adj (sclerosis or nephritis)).ti,ab. | 3081 |
| 16 | ((renal or distal or proximal or tubul*) adj2 acidos*).ti,ab. | 4456 |
| 17 | hyperuricemia/ or hyperuric?emi*.ti,ab. | 24155 |
| 18 | secondary hyperparathyroidism/ or renal osteodystrophy/ | 13564 |
| 19 | (renal adj2 (osteo* or hyperparathyroidism)).ti,ab. | 4971 |
| 20 | or/1-19 | 544304 |
| 21 | obstructive uropathy/ | 3656 |
| 22 | exp urinary tract obstruction/ | 31358 |
| 23 | ((uropath* or ureter* or urethra*) adj obstruct*).ti,ab. | 11244 |
| 24 | (renal or chronic or kidney).ti,ab. | 3042165 |
| 25 | (21 or 22 or 23) and 24 | 18528 |
| 26 | 20 or 25 | 558054 |
| 27 | (transplant* or donor* or graft* or allograft*).ti. | 646632 |
| 28 | pregnan*.ti. | 303749 |
| 29 | *hemodialysis/ not (predialysis or pre dialysis or ("not" adj4 dialysis)).ti. | 60113 |
| 30 | 26 not (27 or 28 or 29) | 498066 |
| 31 | *hyperkalemia/ or *hyperkalaemia/ | 5948 |
| 32 | (hyperkalemi$ or hyperkalaemi$).tw. | 15092 |
| 33 | (hyperpotassemi$ or hyperpotassaemi$).tw. | 171 |
| 34 | *hypokalemia/ or *hypokalaemia/ | 6584 |
| 35 | (hypokalemi$ or hypokalaemi$).tw. | 19329 |
| 36 | (normokalaemi$ or normokalemi$).tw. | 1030 |
| 37 | ((maintenance or maintain$ or achiev$ or increas$ or elevat$ or reduc$ or lower$ or decreas$ or abnormal$ or normal$ or high$) and potassium).tw. | 137297 |
| 38 | or/31-37 | 162379 |
| 39 | 30 and 38 | 10815 |
| **Intervention terms** | | |
| 40 | exp dipeptidyl carboxypeptidase inhibitor/ | 200807 |
| 41 | dipeptidyl carboxypeptidase inhibit*.mp. | 133687 |
| 42 | (ACE or ACE1 or ACEI or ACE-I or ACEs).mp. | 76871 |
| 43 | captopril/ or cilazapril/ or enalapril/ or enalaprilat/ or fosinopril/ or lisinopril/ or perindopril/ or ramipril/ or teprotide/ | 86733 |
| 44 | (alacepril or benazepril or benazeprila or benazeprilat or captopril or ceranapril or ceronapril or cilazapril or cilazaprilat or deacetylalacepril or delapril or enalapril or enalaprilat or fosinopril or fosinoprilic acid or imidapril or libenzapril or lisinopril or moexipril or perindopril or quinapril or quinaprilat or ramipril or ramiprilat or rentiapril or spirapril or temocapril or teprotide or trandolapril or zofenopril).mp. | 99460 |
| 45 | (innovace* or innozide* or zestril* or carace* or zestoretic* or coversyl* or accupro* or accuretic* or tritace* or triapin* or vascace* or capoten* or capozide* or cozidocapt* or zidocapt* or gopten* or tarka* or tanatril* or perdix*).mp. | 2920 |
| 46 | angiotensin 1 receptor antagonist/ | 6141 |
| 47 | angiotensin 2 receptor antagonist/ | 7541 |
| 48 | (angiotensin receptor antagonist or angiotensin 1 receptor antagonist or angiotensin I antagonist or AT 1 receptor blocker or AT 1 receptor antagonist or angiotensin 2 receptor antagonist or angiotensin II antagonist or AT 2 receptor blocker or AT 2 receptor antagonist or ARB or ARBs).mp. | 79927 |
| 49 | losartan/ or saralasin/ or valsartan/ | 39262 |
| 50 | (azilsartan or candesartan or elisartan or embusartan or eprosartan or forasartan or irbesartan or losartan or olmesartan or saprisartan or saralasin or tasosartan or telmisartan or valsartan or vasvalsartan or zolasartan).mp. | 68377 |
| 51 | (amias* or aprovel* or atacand* or avalide* or avapro* or benicar* or coaprovel* or codiovan* or cozaar* or diovan* or edarbi* or miardis* or micardis* or olmetec* or sevikar* or teveten*).mp. | 2385 |
| 52 | aldosterone antagonist/ | 9083 |
| 53 | spironolactone/ | 35340 |
| 54 | (eplerenone* or spironolactone*).mp. | 38946 |
| 55 | (alaton* or aldactone* or crl635 or crl635 or coflumactone* or flumactone* or inspra* or lasilactone* or osiren* or osyrol* or prilactone* or sas 1060 or sas1060 or sc 9420 or sc9420 or spiractin* or spiridon* or spiro ct or spiroctan* or spirohexal* or spirolang* or uractone* or verospiron* or xenalon*).mp. | 2704 |
| 56 | ("cgp 30 083" or cgp 30083 or cgp30083 or sc 66110 or sc66110).mp. | 19 |
| 57 | (aliskiren* or rasilez*).mp. | 3709 |
| 58 | ((angiotensin* or renin or aldosterone or ACE) adj5 (antagonist* or blocker* or inhibitor*)).mp. | 136581 |
| 59 | (RAAS or RAS or RASI or RAASi or RAAS inhibit* or renin angiotensin aldosterone system inhibit*).mp. | 145543 |
| 60 | (valsartan and sucubitril).mp. | 4 |
| 61 | (entresto or lcz 696 or lcz696 or neparvis or "valsartan plus sucubitril").mp. | 1236 |
| 62 | or/40-61 | 462158 |
| **Dosing terms** | | |
| 63 | ((suboptim* or sub-optim*) adj2 (medicine? or medicat* or prescrib* or prescription* or drug*)).mp. | 1485 |
| 64 | ((under adj prescrib*) or underprescrib* or under-prescrib*).mp. | 984 |
| 65 | ((reduc* or modif* or discontinu*) adj (medicine? or medicat* or prescrib* or prescription* or drug*)).mp. | 20105 |
| 66 | (down-titration or downtitration).mp. | 530 |
| 67 | or/63-66 | 23049 |
| **Study design terms** | | |
| 68 | Randomized Controlled Trial/ | 781096 |
| 69 | exp Randomized Controlled Trials as Topic/ | 258571 |
| 70 | Clinical Trial/ | 1073779 |
| 71 | controlled clinical trial/ | 469077 |
| 72 | multicenter study/ | 374644 |
| 73 | Phase 1 clinical trial/ | 70993 |
| 74 | Phase 2 clinical trial/ | 106396 |
| 75 | Phase 3 clinical trial/ | 69139 |
| 76 | Phase 4 clinical trial/ | 5410 |
| 77 | exp randomization/ | 99208 |
| 78 | Single Blind Procedure/ | 51535 |
| 79 | Double Blind Procedure/ | 209600 |
| 80 | Crossover Procedure/ | 74919 |
| 81 | Placebo/ | 401844 |
| 82 | (randomi?ed controlled trial* or rct).tw. | 347938 |
| 83 | (random* adj2 allocat*).tw. | 54439 |
| 84 | ((singl* or doubl* or treb* or tripl*) adj (blind$3 or dumm$3 or mask$3)).tw. | 281298 |
| 85 | placebo*.tw. | 366735 |
| 86 | Prospective Study/ | 870565 |
| 87 | or/68-86 | 3110438 |
| 88 | case control study/ | 205040 |
| 89 | longitudinal study/ | 192319 |
| 90 | retrospective study/ | 1455023 |
| 91 | prospective study/ | 870565 |
| 92 | exp observational study/ | 324922 |
| 93 | exp register/ | 191274 |
| 94 | cohort analysis/ | 1024497 |
| 95 | (Case control adj2 stud*).mp. | 271575 |
| 96 | (observational adj2 stud*).mp. | 426279 |
| 97 | (cross sectional adj2 stud*).mp. | 635282 |
| 98 | (rwe or 'real world').mp. | 131516 |
| 99 | ((chart or regist*) adj2 (review* or stud*)).mp. | 168001 |
| 100 | ((case control or prospective or retrospective or longitudinal) adj2 stud*).mp. | 2945480 |
| 101 | (cohort adj2 stud*).mp. | 521774 |
| 102 | (follow up adj2 stud*).mp. | 98852 |
| 103 | or/88-102 | 4415923 |
| 104 | 62 or 67 | 484154 |
| 105 | 87 or 103 | 6309224 |
| 106 | 39 and 104 and 105 | 1478 |
| **Limits** | | |
| 107 | limit 106 to english language | 1425 |
| 108 | (((animal$ not human$).mp. or animal/) not (animal/ and human/)) or animal/ or animal experiment/ or animal model/ or animal tissue/ or nonhuman/ | 9562629 |
| 109 | (news or comment or editorial or note or case reports or letter).pt. | 3008897 |
| 110 | 108 or 109 | 12280680 |
| 111 | 107 not 110 | 1302 |
| 112 | limit 111 to yr="2000 -Current" | 1234 |
| 113 | conference.so. | 649337 |
| 114 | conference abstract.pt. | 4736772 |
| 115 | 113 or 114 | 4750448 |
| 116 | limit 115 to yr="2000 - 2020" | 4133174 |
| 117 | 112 not 116 | 876 |

Table S6. Suboptimal dosing search strategy (Embase^®^) - SLR update

| **#** | **Searches** | **Results** |
| --- | --- | --- |
| **Database(s): Embase 1974 to 2024 April 05. Search date: 08 April 2024** | | |
| **Disease terms** | | |
| 1 | chronic kidney disease/ | 112517 |
| 2 | chronic kidney failure/ | 158745 |
| 3 | (kidney failure/ or kidney disease/) and chronic.ti,ab. | 51948 |
| 4 | ((chronic or progressive) adj2 (renal or kidney)).ti,ab. | 183298 |
| 5 | (chronic adj (kidney or renal) adj insufficienc*).ti,ab. | 6943 |
| 6 | CKD.ti,ab. | 88421 |
| 7 | diabetic nephropathy/ | 56386 |
| 8 | exp glomerulonephritis/ | 70899 |
| 9 | exp proteinuria/ | 133471 |
| 10 | kidney tubule acidosis/ | 4800 |
| 11 | renovascular hypertension/ | 15408 |
| 12 | (diabetic adj (kidney or renal) adj (disease* or failure)).ti,ab. | 8939 |
| 13 | ((renal or renovascular) adj2 hypertensi*).ti,ab. | 18594 |
| 14 | (glomerulosclerosis or glomerulonephritis or nephropath* or proteinuria* or albuminuria or microalbuminuria).ti,ab. | 209778 |
| 15 | (glomerular adj (sclerosis or nephritis)).ti,ab. | 3230 |
| 16 | ((renal or distal or proximal or tubul*) adj2 acidos*).ti,ab. | 4669 |
| 17 | hyperuricemia/ or hyperuric?emi*.ti,ab. | 25806 |
| 18 | secondary hyperparathyroidism/ or renal osteodystrophy/ | 13984 |
| 19 | (renal adj2 (osteo* or hyperparathyroidism)).ti,ab. | 5099 |
| 20 | or/1-19 | 579959 |
| 21 | obstructive uropathy/ | 3920 |
| 22 | exp urinary tract obstruction/ | 32725 |
| 23 | ((uropath* or ureter* or urethra*) adj obstruct*).ti,ab. | 11685 |
| 24 | (renal or chronic or kidney).ti,ab. | 3169839 |
| 25 | (21 or 22 or 23) and 24 | 19507 |
| 26 | 20 or 25 | 594278 |
| 27 | (transplant* or donor* or graft* or allograft*).ti. | 666830 |
| 28 | pregnan*.ti. | 314837 |
| 29 | *hemodialysis/ not (predialysis or pre dialysis or ("not" adj4 dialysis)).ti. | 65004 |
| 30 | 26 not (27 or 28 or 29) | 530172 |
| 31 | *hyperkalemia/ or *hyperkalaemia/ | 6178 |
| 32 | (hyperkalemi$ or hyperkalaemi$).tw. | 16206 |
| 33 | (hyperpotassemi$ or hyperpotassaemi$).tw. | 181 |
| 34 | *hypokalemia/ or *hypokalaemia/ | 6729 |
| 35 | (hypokalemi$ or hypokalaemi$).tw. | 20605 |
| 36 | (normokalaemi$ or normokalemi$).tw. | 1090 |
| 37 | ((maintenance or maintain$ or achiev$ or increas$ or elevat$ or reduc$ or lower$ or decreas$ or abnormal$ or normal$ or high$) and potassium).tw. | 142623 |
| 38 | or/31-37 | 169099 |
| 39 | 30 and 38 | 11702 |
| **Intervention terms** | | |
| 40 | exp dipeptidyl carboxypeptidase inhibitor/ | 209815 |
| 41 | dipeptidyl carboxypeptidase inhibit*.mp. | 140197 |
| 42 | (ACE or ACE1 or ACEI or ACE-I or ACEs).mp. | 80035 |
| 43 | captopril/ or cilazapril/ or enalapril/ or enalaprilat/ or fosinopril/ or lisinopril/ or perindopril/ or ramipril/ or teprotide/ | 89461 |
| 44 | (alacepril or benazepril or benazeprila or benazeprilat or captopril or ceranapril or ceronapril or cilazapril or cilazaprilat or deacetylalacepril or delapril or enalapril or enalaprilat or fosinopril or fosinoprilic acid or imidapril or libenzapril or lisinopril or moexipril or perindopril or quinapril or quinaprilat or ramipril or ramiprilat or rentiapril or spirapril or temocapril or teprotide or trandolapril or zofenopril).mp. | 102562 |
| 45 | (innovace* or innozide* or zestril* or carace* or zestoretic* or coversyl* or accupro* or accuretic* or tritace* or triapin* or vascace* or capoten* or capozide* or cozidocapt* or zidocapt* or gopten* or tarka* or tanatril* or perdix*).mp. | 2956 |
| 46 | angiotensin 1 receptor antagonist/ | 6201 |
| 47 | angiotensin 2 receptor antagonist/ | 7764 |
| 48 | (angiotensin receptor antagonist or angiotensin 1 receptor antagonist or angiotensin I antagonist or AT 1 receptor blocker or AT 1 receptor antagonist or angiotensin 2 receptor antagonist or angiotensin II antagonist or AT 2 receptor blocker or AT 2 receptor antagonist or ARB or ARBs).mp. | 85800 |
| 49 | losartan/ or saralasin/ or valsartan/ | 40998 |
| 50 | (azilsartan or candesartan or elisartan or embusartan or eprosartan or forasartan or irbesartan or losartan or olmesartan or saprisartan or saralasin or tasosartan or telmisartan or valsartan or vasvalsartan or zolasartan).mp. | 72107 |
| 51 | (amias* or aprovel* or atacand* or avalide* or avapro* or benicar* or coaprovel* or codiovan* or cozaar* or diovan* or edarbi* or miardis* or micardis* or olmetec* or sevikar* or teveten*).mp. | 2421 |
| 52 | aldosterone antagonist/ | 9619 |
| 53 | spironolactone/ | 37392 |
| 54 | (eplerenone* or spironolactone*).mp. | 41193 |
| 55 | (alaton* or aldactone* or crl635 or crl635 or coflumactone* or flumactone* or inspra* or lasilactone* or osiren* or osyrol* or prilactone* or sas 1060 or sas1060 or sc 9420 or sc9420 or spiractin* or spiridon* or spiro ct or spiroctan* or spirohexal* or spirolang* or uractone* or verospiron* or xenalon*).mp. | 2741 |
| 56 | ("cgp 30 083" or cgp 30083 or cgp30083 or sc 66110 or sc66110).mp. | 19 |
| 57 | (aliskiren* or rasilez*).mp. | 3768 |
| 58 | ((angiotensin* or renin or aldosterone or ACE) adj5 (antagonist* or blocker* or inhibitor*)).mp. | 143511 |
| 59 | (RAAS or RAS or RASI or RAASi or RAAS inhibit* or renin angiotensin aldosterone system inhibit*).mp. | 151855 |
| 60 | (valsartan and sucubitril).mp. | 4 |
| 61 | (entresto or lcz 696 or lcz696 or neparvis or "valsartan plus sucubitril").mp. | 1317 |
| 62 | or/40-61 | 484776 |
| **Dosing terms** | | |
| 63 | ((suboptim* or sub-optim*) adj2 (medicine? or medicat* or prescrib* or prescription* or drug*)).mp. | 1571 |
| 64 | ((under adj prescrib*) or underprescrib* or under-prescrib*).mp. | 1084 |
| 65 | ((reduc* or modif* or discontinu*) adj (medicine? or medicat* or prescrib* or prescription* or drug*)).mp. | 21329 |
| 66 | (down-titration or downtitration).mp. | 557 |
| 67 | or/63-66 | 24475 |
| **Study design terms** | | |
| 68 | Randomized Controlled Trial/ | 815260 |
| 69 | exp Randomized Controlled Trials as Topic/ | 271958 |
| 70 | Clinical Trial/ | 1080586 |
| 71 | controlled clinical trial/ | 472804 |
| 72 | multicenter study/ | 388904 |
| 73 | Phase 1 clinical trial/ | 75678 |
| 74 | Phase 2 clinical trial/ | 113034 |
| 75 | Phase 3 clinical trial/ | 74783 |
| 76 | Phase 4 clinical trial/ | 7068 |
| 77 | exp randomization/ | 99486 |
| 78 | Single Blind Procedure/ | 54196 |
| 79 | Double Blind Procedure/ | 217604 |
| 80 | Crossover Procedure/ | 77520 |
| 81 | Placebo/ | 411093 |
| 82 | (randomi?ed controlled trial* or rct).tw. | 368366 |
| 83 | (random* adj2 allocat*).tw. | 56975 |
| 84 | ((singl* or doubl* or treb* or tripl*) adj (blind$3 or dumm$3 or mask$3)).tw. | 289305 |
| 85 | placebo*.tw. | 377299 |
| 86 | Prospective Study/ | 912538 |
| 87 | or/68-86 | 3226211 |
| 88 | case control study/ | 215465 |
| 89 | longitudinal study/ | 210100 |
| 90 | retrospective study/ | 1596412 |
| 91 | prospective study/ | 912538 |
| 92 | exp observational study/ | 367080 |
| 93 | exp register/ | 199377 |
| 94 | cohort analysis/ | 1142835 |
| 95 | (Case control adj2 stud*).mp. | 283477 |
| 96 | (observational adj2 stud*).mp. | 474884 |
| 97 | (cross sectional adj2 stud*).mp. | 704491 |
| 98 | (rwe or 'real world').mp. | 151384 |
| 99 | ((chart or regist*) adj2 (review* or stud*)).mp. | 180027 |
| 100 | ((case control or prospective or retrospective or longitudinal) adj2 stud*).mp. | 3158213 |
| 101 | (cohort adj2 stud*).mp. | 559570 |
| 102 | (follow up adj2 stud*).mp. | 102273 |
| 103 | or/88-102 | 4755691 |
| 104 | 62 or 67 | 508107 |
| 105 | 87 or 103 | 6701055 |
| 106 | 39 and 104 and 105 | 1615 |
| **Limits** | | |
| 107 | limit 106 to english language | 1561 |
| 108 | (((animal$ not human$).mp. or animal/) not (animal/ and human/)) or animal/ or animal experiment/ or animal model/ or animal tissue/ or nonhuman/ | 9844249 |
| 109 | (news or comment or editorial or note or case reports or letter).pt. | 3096097 |
| 110 | 108 or 109 | 12640168 |
| 111 | 107 not 110 | 1436 |
| 112 | limit 111 to yr="2023 -Current" | 176 |
| 113 | conference.so. | 698451 |
| 114 | conference abstract.pt. | 5101169 |
| 115 | 113 or 114 | 5115068 |
| 116 | limit 115 to yr="2000-2022" | 4772370 |
| 117 | 112 not 116 | 176 |

Table S7. Suboptimal dosing search strategy (Ovid Medline^®^) – original SLR

| **#** | **Searches** | **Results** |
| --- | --- | --- |
| **Database(s): Ovid MEDLINE(R) and Epub Ahead of Print, In-Process, In-Data-Review & Other Non-Indexed Citations and Daily 1946 to April 27, 2023. Search date: 28 April 2023** | | |
| **Disease terms** | | |
| 1 | renal insufficiency, chronic/ | 35135 |
| 2 | exp kidney failure, chronic/ | 100620 |
| 3 | kidney diseases/ and chronic.ti,ab. | 13144 |
| 4 | ((chronic or progressive) adj2 (renal or kidney)).ti,ab. | 108776 |
| 5 | (chronic adj (kidney or renal) adj insufficienc*).ti,ab. | 5318 |
| 6 | CKD.ti,ab. | 41306 |
| 7 | diabetic nephropathies/ | 29258 |
| 8 | exp glomerulonephritis/ | 51784 |
| 9 | exp proteinuria/ | 42276 |
| 10 | acidosis, renal tubular/ | 2911 |
| 11 | exp hypertension, renal/ | 19712 |
| 12 | (diabetic adj (kidney or renal) adj (disease* or failure)).ti,ab. | 5171 |
| 13 | ((renal or renovascular) adj2 hypertensi*).ti,ab. | 14709 |
| 14 | (glomerulosclerosis or glomerulonephritis or nephropath* or proteinuria* or albuminuria or microalbuminuria).ti,ab. | 136732 |
| 15 | (glomerular adj (sclerosis or nephritis)).ti,ab. | 2259 |
| 16 | ((renal or distal or proximal or tubul*) adj2 acidos*).ti,ab. | 3536 |
| 17 | hyperuricemia/ or hyperuric?emi*.ti,ab. | 11554 |
| 18 | exp hyperparathyroidism, secondary/ | 8998 |
| 19 | (renal adj2 (osteo* or hyperparathyroidism)).ti,ab. | 3823 |
| 20 | or/1-19 | 376046 |
| 21 | ureteral obstruction/ | 14583 |
| 22 | exp urethral obstruction/ | 12015 |
| 23 | ((uropath* or ureter* or urethra*) adj obstruct*).ti,ab. | 8304 |
| 24 | (renal of kidney or chronic).ti,ab. | 1363235 |
| 25 | (21 or 22 or 23) and 24 | 2119 |
| 26 | 20 or 25 | 376924 |
| 27 | (transplant* or donor* or graft* or allograft*).ti. | 472277 |
| 28 | pregnan*.ti. | 262363 |
| 29 | *renal dialysis/ not (predialysis or pre dialysis or ("not" adj4 dialysis)).ti. | 66139 |
| 30 | 26 not (27 or 28 or 29) | 315260 |
| 31 | *hyperkalemia/ or *hyperkalaemia/ | 4432 |
| 32 | (hyperkalemi$ or hyperkalaemi$).tw. | 9816 |
| 33 | (hyperpotassemi$ or hyperpotassaemi$).tw. | 176 |
| 34 | *hypokalemia/ or *hypokalaemia/ | 5468 |
| 35 | (hypokalemi$ or hypokalaemi$).tw. | 13007 |
| 36 | (normokalaemi$ or normokalemi$).tw. | 706 |
| 37 | ((maintenance or maintain$ or achiev$ or increas$ or elevat$ or reduc$ or lower$ or decreas$ or abnormal$ or normal$ or high$) and potassium).tw. | 109373 |
| 38 | or/31-37 | 127194 |
| 39 | 30 and 38 | 6331 |
| **Intervention terms** | | |
| 40 | angiotensin-converting enzyme inhibitors/ | 35727 |
| 41 | angiotensin converting enzyme inhibit*.mp. | 46354 |
| 42 | (ACE or ACE1 or ACEI or ACE-I or ACEs).mp. | 46844 |
| 43 | captopril/ or cilazapril/ or enalapril/ or enalaprilat/ or fosinopril/ or lisinopril/ or perindopril/ or ramipril/ or teprotide/ | 22531 |
| 44 | (alacepril or benazepril or benazeprila or benazeprilat or captopril or ceranapril or ceronapril or cilazapril or cilazaprilat or deacetylalacepril or delapril or enalapril or enalaprilat or fosinopril or fosinoprilic acid or imidapril or libenzapril or lisinopril or moexipril or perindopril or quinapril or quinaprilat or ramipril or ramiprilat or rentiapril or spirapril or temocapril or teprotide or trandolapril or zofenopril).mp. | 32429 |
| 45 | (innovace* or innozide* or zestril* or carace* or zestoretic* or coversyl* or accupro* or accuretic* or tritace* or triapin* or vascace* or capoten* or capozide* or cozidocapt* or zidocapt* or gopten* or tarka* or tanatril* or perdix*).mp. | 657 |
| 46 | angiotensin ii type 1 receptor blockers/ | 9074 |
| 47 | angiotensin ii type 2 receptor blockers/ | 588 |
| 48 | (angiotensin receptor antagonist or angiotensin 1 receptor antagonist or angiotensin I antagonist or AT 1 receptor blocker or AT 1 receptor antagonist or angiotensin 2 receptor antagonist or angiotensin II antagonist or AT 2 receptor blocker or AT 2 receptor antagonist or ARB or ARBs).mp. | 10701 |
| 49 | losartan/ or saralasin/ or valsartan/ | 11875 |
| 50 | (azilsartan or candesartan or elisartan or embusartan or eprosartan or forasartan or irbesartan or losartan or olmesartan or saprisartan or saralasin or tasosartan or telmisartan or valsartan or vasvalsartan or zolasartan).mp. | 25510 |
| 51 | (amias* or aprovel* or atacand* or avalide* or avapro* or benicar* or coaprovel* or codiovan* or cozaar* or diovan* or edarbi* or miardis* or micardis* or olmetec* or sevikar* or teveten*).mp. | 286 |
| 52 | aldosterone antagonists/ | 5734 |
| 53 | spironolactone/ | 7155 |
| 54 | (eplerenone* or spironolactone*).mp. | 10328 |
| 55 | (alaton* or aldactone* or crl635 or crl635 or coflumactone* or flumactone* or inspra* or lasilactone* or osiren* or osyrol* or prilactone* or sas 1060 or sas1060 or sc 9420 or sc9420 or spiractin* or spiridon* or spiro ct or spiroctan* or spirohexal* or spirolang* or uractone* or verospiron* or xenalon*).mp. | 579 |
| 56 | ("cgp 30 083" or cgp 30083 or cgp30083 or sc 66110 or sc66110).mp. | 1 |
| 57 | (aliskiren* or rasilez*).mp. | 1306 |
| 58 | ((angiotensin* or renin or aldosterone or ACE) adj5 (antagonist* or blocker* or inhibitor*)).mp. | 77669 |
| 59 | (RAAS or RAS or RASI or RAASi or RAAS inhibit* or renin angiotensin aldosterone system inhibit*).mp. | 84233 |
| 60 | (valsartan and sucubitril).mp. | 1 |
| 61 | (entresto or lcz 696 or lcz696 or neparvis or "valsartan plus sucubitril").mp. | 418 |
| 62 | or/40-61 | 206311 |
| **Dosing terms** | | |
| 63 | ((suboptim* or sub-optim*) adj2 (medicine? or medicat* or prescrib* or prescription* or drug*)).mp. | 930 |
| 64 | ((under adj prescrib*) or underprescrib* or under-prescrib*).mp. | 610 |
| 65 | ((reduc* or modif* or discontinu*) adj (medicine? or medicat* or prescrib* or prescription* or drug*)).mp. | 12308 |
| 66 | (down-titration or downtitration).mp. | 178 |
| 67 | or/63-66 | 13992 |
| **Study design terms** | | |
| 68 | Randomized Controlled Trial/ | 591490 |
| 69 | Randomized Controlled Trials as Topic/ | 161658 |
| 70 | Clinical Trial/ | 537833 |
| 71 | Random Allocation/ | 106924 |
| 72 | exp Clinical Trials as topic/ | 381779 |
| 73 | clinical trial, phase i.pt. | 24829 |
| 74 | clinical trial, phase ii.pt. | 39563 |
| 75 | clinical trial, phase iii.pt. | 21627 |
| 76 | clinical trial, phase iv.pt. | 2406 |
| 77 | Single Blind Method/ | 32655 |
| 78 | Double Blind Method/ | 174974 |
| 79 | PLACEBOS/ | 35926 |
| 80 | controlled clinical trial.pt. | 95280 |
| 81 | multicenter study.pt. | 333109 |
| 82 | randomized controlled trial.pt. | 591490 |
| 83 | clinical trial.pt. | 537833 |
| 84 | (clinical adj trial*).tw. | 471346 |
| 85 | (randomi?ed controlled trial* or rct).tw. | 254139 |
| 86 | (random* adj2 allocat*).tw. | 43229 |
| 87 | ((singl* or doubl* or treb* or tripl*) adj (blind$3 or dumm$3 or mask$3)).tw. | 196419 |
| 88 | placebo*.tw. | 245317 |
| 89 | Case-Control Studies/ | 327367 |
| 90 | Longitudinal Studies/ | 164516 |
| 91 | Retrospective Studies/ | 1111948 |
| 92 | Prospective Studies/ | 656858 |
| 93 | observational study/ | 140937 |
| 94 | Registries/ | 107813 |
| 95 | Cohort Studies/ | 327473 |
| 96 | (Case control adj2 stud*).mp. | 369617 |
| 97 | (observational adj2 stud*).mp. | 263205 |
| 98 | (cross sectional adj2 stud*).mp. | 550258 |
| 99 | (rwe or 'real world').mp. | 76183 |
| 100 | ((chart or regist*) adj2 (review* or stud*)).mp. | 93615 |
| 101 | ((case control or prospective or retrospective or longitudinal) adj2 stud*).mp. | 2420941 |
| 102 | (cohort adj2 stud*).mp. | 545672 |
| 103 | (follow up adj2 stud*).mp. | 724834 |
| 104 | or/68-103 | 5147089 |
| 105 | 62 or 67 | 219973 |
| 106 | 39 and 104 and 105 | 783 |
| **Limits** | | |
| 107 | limit 106 to english language | 738 |
| 108 | (((animal$ not human$).mp. or animal/) not (animal/ and human/)) or animal/ or animal experiment/ or animal model/ or animal tissue/ or nonhuman/ | 7377996 |
| 109 | (news or comment or editorial or note or case reports or letter).pt. | 4456691 |
| 110 | 108 or 109 | 11578258 |
| 111 | 107 not 110 | 682 |
| 112 | limit 111 to yr="2000 -Current" | 632 |

Table S8. Suboptimal dosing search strategy (Ovid Medline^®^) – SLR update

| **#** | **Searches** | **Results** |
| --- | --- | --- |
| **Database(s): Ovid MEDLINE(R) and Epub Ahead of Print, In-Process, In-Data-Review & Other Non-Indexed Citations and Daily 1946 to April 05, 2024. Search date: 08 April 2024** | | |
| **Disease terms** | | |
| 1 | renal insufficiency, chronic/ | 38588 |
| 2 | exp kidney failure, chronic/ | 102261 |
| 3 | kidney diseases/ and chronic.ti,ab. | 13330 |
| 4 | ((chronic or progressive) adj2 (renal or kidney)).ti,ab. | 116558 |
| 5 | (chronic adj (kidney or renal) adj insufficienc*).ti,ab. | 5395 |
| 6 | CKD.ti,ab. | 45692 |
| 7 | diabetic nephropathies/ | 30347 |
| 8 | exp glomerulonephritis/ | 53076 |
| 9 | exp proteinuria/ | 43144 |
| 10 | acidosis, renal tubular/ | 2949 |
| 11 | exp hypertension, renal/ | 19798 |
| 12 | (diabetic adj (kidney or renal) adj (disease* or failure)).ti,ab. | 6021 |
| 13 | ((renal or renovascular) adj2 hypertensi*).ti,ab. | 14936 |
| 14 | (glomerulosclerosis or glomerulonephritis or nephropath* or proteinuria* or albuminuria or microalbuminuria).ti,ab. | 142114 |
| 15 | (glomerular adj (sclerosis or nephritis)).ti,ab. | 2297 |
| 16 | ((renal or distal or proximal or tubul*) adj2 acidos*).ti,ab. | 3646 |
| 17 | hyperuricemia/ or hyperuric?emi*.ti,ab. | 12355 |
| 18 | exp hyperparathyroidism, secondary/ | 9136 |
| 19 | (renal adj2 (osteo* or hyperparathyroidism)).ti,ab. | 3888 |
| 20 | or/1-19 | 391356 |
| 21 | ureteral obstruction/ | 14893 |
| 22 | exp urethral obstruction/ | 12242 |
| 23 | ((uropath* or ureter* or urethra*) adj obstruct*).ti,ab. | 8613 |
| 24 | (renal of kidney or chronic).ti,ab. | 1434188 |
| 25 | (21 or 22 or 23) and 24 | 2263 |
| 26 | 20 or 25 | 392253 |
| 27 | (transplant* or donor* or graft* or allograft*).ti. | 489331 |
| 28 | pregnan*.ti. | 273573 |
| 29 | *renal dialysis/ not (predialysis or pre dialysis or ("not" adj4 dialysis)).ti. | 66554 |
| 30 | 26 not (27 or 28 or 29) | 329642 |
| 31 | *hyperkalemia/ or *hyperkalaemia/ | 4610 |
| 32 | (hyperkalemi$ or hyperkalaemi$).tw. | 10355 |
| 33 | (hyperpotassemi$ or hyperpotassaemi$).tw. | 177 |
| 34 | *hypokalemia/ or *hypokalaemia/ | 5605 |
| 35 | (hypokalemi$ or hypokalaemi$).tw. | 13555 |
| 36 | (normokalaemi$ or normokalemi$).tw. | 728 |
| 37 | ((maintenance or maintain$ or achiev$ or increas$ or elevat$ or reduc$ or lower$ or decreas$ or abnormal$ or normal$ or high$) and potassium).tw. | 113831 |
| 38 | or/31-37 | 132342 |
| 39 | 30 and 38 | 6702 |
| **Intervention terms** | | |
| 40 | angiotensin-converting enzyme inhibitors/ | 36422 |
| 41 | angiotensin converting enzyme inhibit*.mp. | 47457 |
| 42 | (ACE or ACE1 or ACEI or ACE-I or ACEs).mp. | 49221 |
| 43 | captopril/ or cilazapril/ or enalapril/ or enalaprilat/ or fosinopril/ or lisinopril/ or perindopril/ or ramipril/ or teprotide/ | 22663 |
| 44 | (alacepril or benazepril or benazeprila or benazeprilat or captopril or ceranapril or ceronapril or cilazapril or cilazaprilat or deacetylalacepril or delapril or enalapril or enalaprilat or fosinopril or fosinoprilic acid or imidapril or libenzapril or lisinopril or moexipril or perindopril or quinapril or quinaprilat or ramipril or ramiprilat or rentiapril or spirapril or temocapril or teprotide or trandolapril or zofenopril).mp. | 32819 |
| 45 | (innovace* or innozide* or zestril* or carace* or zestoretic* or coversyl* or accupro* or accuretic* or tritace* or triapin* or vascace* or capoten* or capozide* or cozidocapt* or zidocapt* or gopten* or tarka* or tanatril* or perdix*).mp. | 668 |
| 46 | angiotensin ii type 1 receptor blockers/ | 9104 |
| 47 | angiotensin ii type 2 receptor blockers/ | 588 |
| 48 | (angiotensin receptor antagonist or angiotensin 1 receptor antagonist or angiotensin I antagonist or AT 1 receptor blocker or AT 1 receptor antagonist or angiotensin 2 receptor antagonist or angiotensin II antagonist or AT 2 receptor blocker or AT 2 receptor antagonist or ARB or ARBs).mp. | 11323 |
| 49 | losartan/ or saralasin/ or valsartan/ | 12158 |
| 50 | (azilsartan or candesartan or elisartan or embusartan or eprosartan or forasartan or irbesartan or losartan or olmesartan or saprisartan or saralasin or tasosartan or telmisartan or valsartan or vasvalsartan or zolasartan).mp. | 26363 |
| 51 | (amias* or aprovel* or atacand* or avalide* or avapro* or benicar* or coaprovel* or codiovan* or cozaar* or diovan* or edarbi* or miardis* or micardis* or olmetec* or sevikar* or teveten*).mp. | 293 |
| 52 | aldosterone antagonists/ | 5914 |
| 53 | spironolactone/ | 7261 |
| 54 | (eplerenone* or spironolactone*).mp. | 10620 |
| 55 | (alaton* or aldactone* or crl635 or crl635 or coflumactone* or flumactone* or inspra* or lasilactone* or osiren* or osyrol* or prilactone* or sas 1060 or sas1060 or sc 9420 or sc9420 or spiractin* or spiridon* or spiro ct or spiroctan* or spirohexal* or spirolang* or uractone* or verospiron* or xenalon*).mp. | 593 |
| 56 | ("cgp 30 083" or cgp 30083 or cgp30083 or sc 66110 or sc66110).mp. | 1 |
| 57 | (aliskiren* or rasilez*).mp. | 1320 |
| 58 | ((angiotensin* or renin or aldosterone or ACE) adj5 (antagonist* or blocker* or inhibitor*)).mp. | 79730 |
| 59 | (RAAS or RAS or RASI or RAASi or RAAS inhibit* or renin angiotensin aldosterone system inhibit*).mp. | 88056 |
| 60 | (valsartan and sucubitril).mp. | 1 |
| 61 | (entresto or lcz 696 or lcz696 or neparvis or "valsartan plus sucubitril").mp. | 459 |
| 62 | or/40-61 | 214545 |
| **Dosing terms** | | |
| 63 | ((suboptim* or sub-optim*) adj2 (medicine? or medicat* or prescrib* or prescription* or drug*)).mp. | 1017 |
| 64 | ((under adj prescrib*) or underprescrib* or under-prescrib*).mp. | 668 |
| 65 | ((reduc* or modif* or discontinu*) adj (medicine? or medicat* or prescrib* or prescription* or drug*)).mp. | 13219 |
| 66 | (down-titration or downtitration).mp. | 201 |
| 67 | or/63-66 | 15064 |
| **Study design terms** | | |
| 68 | Randomized Controlled Trial/ | 610363 |
| 69 | Randomized Controlled Trials as Topic/ | 168770 |
| 70 | Clinical Trial/ | 539664 |
| 71 | Random Allocation/ | 107073 |
| 72 | exp Clinical Trials as topic/ | 390448 |
| 73 | clinical trial, phase i.pt. | 25803 |
| 74 | clinical trial, phase ii.pt. | 41040 |
| 75 | clinical trial, phase iii.pt. | 22560 |
| 76 | clinical trial, phase iv.pt. | 2484 |
| 77 | Single Blind Method/ | 33354 |
| 78 | Double Blind Method/ | 177958 |
| 79 | PLACEBOS/ | 35934 |
| 80 | controlled clinical trial.pt. | 95518 |
| 81 | multicenter study.pt. | 344345 |
| 82 | randomized controlled trial.pt. | 610363 |
| 83 | clinical trial.pt. | 539664 |
| 84 | (clinical adj trial*).tw. | 507567 |
| 85 | (randomi?ed controlled trial* or rct).tw. | 278740 |
| 86 | (random* adj2 allocat*).tw. | 46026 |
| 87 | ((singl* or doubl* or treb* or tripl*) adj (blind$3 or dumm$3 or mask$3)).tw. | 204314 |
| 88 | placebo*.tw. | 255103 |
| 89 | Case-Control Studies/ | 332823 |
| 90 | Longitudinal Studies/ | 170570 |
| 91 | Retrospective Studies/ | 1192714 |
| 92 | Prospective Studies/ | 684087 |
| 93 | observational study/ | 154051 |
| 94 | Registries/ | 110725 |
| 95 | Cohort Studies/ | 339620 |
| 96 | (Case control adj2 stud*).mp. | 378117 |
| 97 | (observational adj2 stud*).mp. | 287930 |
| 98 | (cross sectional adj2 stud*).mp. | 597567 |
| 99 | (rwe or 'real world').mp. | 91589 |
| 100 | ((chart or regist*) adj2 (review* or stud*)).mp. | 104023 |
| 101 | ((case control or prospective or retrospective or longitudinal) adj2 stud*).mp. | 2562250 |
| 102 | (cohort adj2 stud*).mp. | 586760 |
| 103 | (follow up adj2 stud*).mp. | 731995 |
| 104 | or/68-103 | 5428497 |
| 105 | 62 or 67 | 229250 |
| 106 | 39 and 104 and 105 | 846 |
| **Limits** | | |
| 107 | limit 106 to english language | 798 |
| 108 | (((animal$ not human$).mp. or animal/) not (animal/ and human/)) or animal/ or animal experiment/ or animal model/ or animal tissue/ or nonhuman/ | 7532922 |
| 109 | (news or comment or editorial or note or case reports or letter).pt. | 4607171 |
| 110 | 108 or 109 | 11879920 |
| 111 | 107 not 110 | 740 |
| 112 | limit 111 to yr="2023-Current" | 85 |

Table S9. List of conference proceedings searched

| Conference proceeding | URL |
| --- | --- |
| Search date: 25th May 2023 and 23rd April 2014 | |
| World Nephrology Conference | <https://www.theisn.org/wcn/> |
| American Society of Nephrology – Kidney week | <https://www.asn-online.org/education/kidneyweek/> |
| European Renal Association/European Dialysis & Transplant Association | <https://www.era-online.org/> |
| The Renal Association | <https://renal.org/> |
| European Society of Cardiology | <https://esc365.escardio.org/home> |
| British Society of Heart Failure | <https://www.bsh.org.uk/> |
| The Professional Society for Health Economics and Outcomes Research (ISPOR) | <https://www.ispor.org/> |

Table S10. Data extraction items

| Study characteristics | Patient demographics and baseline characteristics | Outcomes (by treatment arms and subgroups, if reported) |
| --- | --- | --- |
| - Publication details (author, year, title) - Study name - Study design - Publication type - Study country - Study setting/data source | - Patient population - Population subgroups - Treatments - Sample size - Age, Years - Gender - Race - Baseline treatment - Baseline comorbidities - CKD stages - eGFR (mL/min/1.73 m2) - sCr (mg/dL) - sAlb (g/dL) - sK^+^ (mEq/L) | **Epidemiology:**   - Definition of hyperkalaemia - Definition of hypokalaemia - Definition of normokalaemia - Time period - Prevalence of hyperkalaemia and hypokalaemia/normokalaemia among patients with CKD - Prevalence of hyperkalaemia and hypokalaemia/ normokalaemia among patients with CKD - Risk factors of hyperkalaemia   **Clinical Burden:***   - Population used for analysis - Time period - Hospitalisation rates - Number of hospitalisations - Hospitalisation duration (LOS) - Mortality/morbidity - Requirement of dialysis   **Economic Burden:***   - Population used for analysis - Country of analysis - Cost year - Currency - Perspective - Time point of assessment - Direct costs - Indirect costs   **Humanistic Burden:***   - Population used for analysis - HRQoL tool - HRQoL subscale - Response rates for HRQoL questionnaire - HRQoL score   **Sub-optimal dosing:**   - Definition of hyperkalaemia - Incidence of RAASi-associated hyperkalaemia - Prevalence of RAASi-associated hyperkalaemia - RAASi discontinuation rates due to hyperkalaemia - Proportion of patients with sub-optimal RAASi dosing due to hyperkalaemia - Hospitalisation due to hyperkalaemia - Impact of sub-optimal RAASi dosing on clinical, economic or QoL outcomes |

*Data were extracted for patients with CKD and hyperkalaemia versus patients with CKD and hypokalaemia/normokalaemia.

**Abbreviations:** CKD: chronic kidney disease; eGFR: estimated glomerular filtration rate; g/dL: grams per decilitre; HRQoL: health-related quality of life; m^2^: square metre; mEq/L: milliequivalents per litre; mg/dL milligrams per decilitre; mL/min: millilitre per minute; millimole per litre; QoL: quality of life; RAASi: renin–angiotensin–aldosterone system inhibitors; sAlb: serum albumin; sCr: serum creatinine.
