# Supplementary material for: The burden of hyperkalaemia in chronic kidney disease: a systematic literature review
Source: Clin Kidney J. 2025 Apr 29;18(5):sfaf127. doi: 10.1093/ckj/sfaf127 (PMC12082095; doi:10.1093/ckj/sfaf127)
Supplement: sfaf127_Supplemental_Files [file sfaf127_supplemental_files.zip › Supp4_Results_Tables_S11-S25.docx]

**Supplementary Data**

Table S11. Risk of bias assessment – RCTs

[Table S12. Breakdown of studies by hyperkalaemia definition](#S12)

[Table S13. Study characteristics and patient demographics](#S13)

[Table S14. Baseline disease characteristics across included studies](#S14)

[Table S15. Risk factors for hyperkalaemia](#S15)

[Table S16. Prevalence of hyper-, hypo- and normokalaemia in CKD patients](#S16)

[Table S17. Prevalence of RAASi-associated hyperkalaemia](#S17)

[Table S18. Incidence of hyper-, hypo- and normokalaemia in CKD patients](#S18)

[Table S19. Incidence of RAASi-associated hyperkalaemia](#S19)

[Table S20. Hospitalisations and length of stay among CKD patients](#S20)

[Table S21. RAASi sub-optimal dosing due to hyperkalaemia](#S21)

[Table S22. RAASi discontinuation due to hyperkalaemia](#S22)

[Table S23. Impact of hyperkalaemia on the health-related quality of life](#S23)

[Table S24. Healthcare costs (medical and pharmacy) associated with hyperkalaemia](#S24)

[Table S25. Impact of sub-optimal RAASi dosing/ discontinuation](#S25)

Table S11. Risk of bias assessment – RCTs

| **Study/ Reference** | **Random sequence generation** | **Allocation concealment** | **Blinding of participants and personnel** | **Blinding of outcome assessment** | **Incomplete outcome data** | **Selective reporting** | **Other sources of bias** |
| --- | --- | --- | --- | --- | --- | --- | --- |
| Edwards 2021 ([1](#_ENREF_1)) |  |  |  |  |  |  |  |
| Straburzynska-Migaj 2021 ([2](#_ENREF_2)) |  |  |  |  |  |  |  |
| Ruilope 2020 ([3](#_ENREF_3)) |  |  |  |  |  |  |  |
| Hammer 2019 ([4](#_ENREF_4)) |  |  |  |  |  |  |  |
| Charytan 2019 ([5](#_ENREF_5)) |  |  |  |  |  |  |  |
| Haynes 2018 ([6](#_ENREF_6)) |  |  |  |  |  |  |  |
| Beldhuis 2019 ([7](#_ENREF_7)) |  |  |  |  |  |  |  |
| Provenzano 2022 ([8](#_ENREF_8)) |  |  |  |  |  |  |  |
| Pitt 2021 ([9](#_ENREF_9)) |  |  |  |  |  |  |  |
| Bakris 2020 ([10](#_ENREF_10)) |  |  |  |  |  |  |  |
| Tang 2021 ([11](#_ENREF_11)) |  |  |  |  |  |  |  |
| Bakris 2021 ([12](#_ENREF_12)) |  |  |  |  |  |  |  |
| Frimodt-Moller 2010 ([13](#_ENREF_13)) |  |  |  |  |  |  |  |
| Taheri 2012 ([14](#_ENREF_14)) |  |  |  |  |  |  |  |
| Espinel 2012 ([15](#_ENREF_15)) |  |  |  |  |  |  |  |
| Boesby 2011 ([16](#_ENREF_16)) |  |  |  |  |  |  |  |
| Edwards 2012 ([17](#_ENREF_17)) |  |  |  |  |  |  |  |
| Ando 2014 ([18](#_ENREF_18)) |  |  |  |  |  |  |  |
| Vukusich_2010 ([19](#_ENREF_19)) |  |  |  |  |  |  |  |
| Walsh 2015 ([20](#_ENREF_20)) |  |  |  |  |  |  |  |
| Tumlin 2022 ([21](#_ENREF_21)) |  |  |  |  |  |  |  |
| Weinberg 2009 ([22](#_ENREF_22)) |  |  |  |  |  |  |  |
| Hayashi 2003 ([23](#_ENREF_23)) |  |  |  |  |  |  |  |
| Abolghasmi 2011 ([24](#_ENREF_24)) |  |  |  |  |  |  |  |
| Eschalier 2013 ([25](#_ENREF_25)) |  |  |  |  |  |  |  |
| Pitt 2013 ([26](#_ENREF_26)) |  |  |  |  |  |  |  |
| Persson 2010 ([27](#_ENREF_27)) |  |  |  |  |  |  |  |
| Bhandari 2021 ([28](#_ENREF_28)) |  |  |  |  |  |  |  |
| Zhou 2023b ([29](#_ENREF_29)) |  |  |  |  |  |  |  |
| Bornstein 2024 ([30](#_ENREF_30)) |  |  |  |  |  |  |  |
| Garcia-Prieto 2024 ([31](#_ENREF_31)) |  |  |  |  |  |  |  |
| Tuttle 2024 ([32](#_ENREF_32)) |  |  |  |  |  |  |  |
| Guney 2009 ([33](#_ENREF_33)) |  |  |  |  |  |  |  |
| Edwards 2009 ([34](#_ENREF_34)) |  |  |  |  |  |  |  |

High

Unclear

Low

Table S12. Breakdown of studies by hyperkalaemia definition

| **Definition (sK+ or pK+ in mmol/L or mEq/L)** | **No. of studies (epidemiology and disease burden search** | **No. of studies (sub-optimal dosing search)** | **Total** | **References** |
| --- | --- | --- | --- | --- |
| >4.7 | 1 | 0 | 1 | ([35](#_ENREF_35)) |
| >5.0 | 13 | 14 | 27 | ([1](#_ENREF_1), [8](#_ENREF_8), [30](#_ENREF_30), [36-57](#_ENREF_36)) ([58](#_ENREF_58), [59](#_ENREF_59)) |
| >5.1 | 1 | 0 | 1 | ([60](#_ENREF_60)) |
| >5.2 | 1 | 0 | 1 | ([52](#_ENREF_52)) |
| >5.3 | 1 | 1 | 2 | ([61](#_ENREF_61), [62](#_ENREF_62)) |
| >5.4 | 1 | 1 | 2 | ([63](#_ENREF_63), [64](#_ENREF_64)) |
| >5.5 | 3 | 20* | 23 | ([7](#_ENREF_7), [10](#_ENREF_10), [13](#_ENREF_13), [16](#_ENREF_16), [18](#_ENREF_18), [21](#_ENREF_21), [22](#_ENREF_22), [24](#_ENREF_24), [25](#_ENREF_25), [27](#_ENREF_27), [33](#_ENREF_33), [52](#_ENREF_52), [57](#_ENREF_57), [65-73](#_ENREF_65)) |
| 5.7 | 0 | 1 | 1 | ([31](#_ENREF_31)) |
| >5.7 | 1 | 0 | 1 | ([14](#_ENREF_14)) |
| >6.0 | 13 | 2 | 15 | ([10](#_ENREF_10), [17](#_ENREF_17), [20](#_ENREF_20), [25](#_ENREF_25), [27](#_ENREF_27), [34](#_ENREF_34), [50](#_ENREF_50), [52](#_ENREF_52), [57](#_ENREF_57), [74-79](#_ENREF_74)) |
| >6.2 | 0 | 1** | 1 | ([73](#_ENREF_73)) |
| >6.5 | 2 | 1 | 3 | ([5](#_ENREF_5), [20](#_ENREF_20), [34](#_ENREF_34)) |
| >7.0 | 1 | 0 | 1 | ([20](#_ENREF_20)) |
| ≥5.0 | 4 | 3 | 7 | ([15](#_ENREF_15), [80-85](#_ENREF_80)) |
| ≥5.1 | 2 | 1 | 3 | ([86-88](#_ENREF_86)) |
| ≥5.5 | 3 | 19*** | 22 | ([6](#_ENREF_6), [11](#_ENREF_11), [17](#_ENREF_17), [29](#_ENREF_29), [31](#_ENREF_31), [89-105](#_ENREF_89)) |
| ≥5.6 | 0 | 2 | 2 | ([12](#_ENREF_12), [106](#_ENREF_106)) |
| ≥5.8 | 1 | 0 | 1 | ([107](#_ENREF_107)) |
| ≥6.0 | 4 | 9 | 13 | ([3](#_ENREF_3), [12](#_ENREF_12), [17](#_ENREF_17), [68](#_ENREF_68), [82](#_ENREF_82), [92](#_ENREF_92), [97](#_ENREF_97), [108-113](#_ENREF_108)) |
| ≥6.5 | 1 | 0 | 1 | ([4](#_ENREF_4)) |
| ≥7.0 | 1 | 0 | 1 | ([114](#_ENREF_114)) |
| 5.0 to 5.4 | 2 | 0 | 2 | ([90](#_ENREF_90), [109](#_ENREF_109)) |
| 5.0 to 5.5 | 1 | 0 | 1 | ([75](#_ENREF_75)) |
| 5.0 to <5.5 | 3 | 0 | 2 | ([108](#_ENREF_108), [115](#_ENREF_115)) |
| 5.5 to 5.9 | 2 | 2 | 4 | ([109](#_ENREF_109), [110](#_ENREF_110), [112](#_ENREF_112), [114](#_ENREF_114)) |
| 5.5 to <6.0 | 3 | 0 | 2 | ([82](#_ENREF_82), [108](#_ENREF_108), [115](#_ENREF_115)) |
| >5.0 to <5.5 | 1 | 0 | 1 | ([82](#_ENREF_82)) |
| >5.0 to ≤5.5 | 1 | 0 | 1 | ([79](#_ENREF_79)) |
| >5.5 to ≤6.0 | 1 | 0 | 1 | ([79](#_ENREF_79)) |
| >5.5 to 6.0 | 1 | 0 | 1 | ([75](#_ENREF_75)) |
| ≥5.5 to 6.0 | 1 | 0 | 1 | ([111](#_ENREF_111)) |
| 5.6 to 5.9 | 1 | 0 | 1 | ([12](#_ENREF_12)) |
| 6.0 to 6.4 | 1 | 0 | 1 | ([114](#_ENREF_114)) |
| 6.0 to <6.5 | 1 | 0 | 1 | ([115](#_ENREF_115)) |
| 6.0 to 6.5 | 1 | 0 | 1 | ([4](#_ENREF_4)) |
| 6.5 to 6.9 | 1 | 0 | 1 | ([114](#_ENREF_114)) |
| 6.5 to 8.0 | 1 | 0 | 1 | ([115](#_ENREF_115)) |
| ICD-10 (E87.5) or ICD-9 (276.7) diagnostic code for hyperkalaemia | 2 | 7 | 9 | ([38](#_ENREF_38), [74](#_ENREF_74), [99](#_ENREF_99), [102](#_ENREF_102), [113](#_ENREF_113), [116-119](#_ENREF_116)) |

*Four studies ([13](#_ENREF_13), [16](#_ENREF_16), [73](#_ENREF_73), [102](#_ENREF_102)) considered pK+ measurements;**Considered PK+ measurements; ***Three studies ([11](#_ENREF_11), [94](#_ENREF_94), [102](#_ENREF_102)) considered pK+ measurements.

**Abbreviations:** ICD-10: International Classification of Diseases, Tenth Revision; ICD-9: International Classification of Diseases, Ninth Revision; mEq/L: milliequivalents per litre; mmol/L: millimoles per litre; pK+: plasma potassium; sK+: serum potassium

Table S13. Study characteristics and patient demographics

| **Author** | **Study design** | **Country** | **Data sources/Study setting** | **Population description** | **Hyper-, hypo-, or normokalaemia definition** | **Treatment** | **Sample size** | **Gender (%)** | **Age Mean (SD) or Median (range)** | **Race (%)** |
| --- | --- | --- | --- | --- | --- | --- | --- | --- | --- | --- |
| Li 2023a ([65](#_ENREF_65)) | Prospective observational study | China | Baoding No. 1, Central Hospital of Hebei Medical University | Patients with stage 4-5 CKD + DM + Hyperkalaemia | sK+ >5.5 mmol/L | NA | 120 | Male: 46.0 Female 54.0 | Mean (SD): 50.1 (9.9) | NR |
|  |  |  |  | Patients with stage 4-5 CKD + DM + Normokalaemia | sK+ ≤5.5 mmol/L | NA | 150 | Male: 46.2 Female 53.3 | Mean (SD): 48.6 (10.1) | NR |
| Wang 2023 ([89](#_ENREF_89)) | Prospective observational study | China | Peking University First Hospital | Patients with stage 1-4 CKD + Instantaneous hyperkalaemia | sK+ ≥5.0 mmol/L only once or lasting <3 months | NA | 1,078 | Male: 54.7 Female 45.3 | Mean (SD): 56.3 (15.6) | NR |
|  |  |  |  | Patients with stage 1-4 CKD + Persistent hyperkalaemia | sK+ ≥5.0 mmol/L lasting for ≥3 months | NA |  |  |  |  |
|  |  |  |  | Patients with stage 1-4 CKD + Normokalaemia | NR | NA |  |  |  |  |
| Calabrese 2022 ([60](#_ENREF_60)) | Prospective observational study | Italy | Unit of Nephrology and Dialysis of the University Hospital of Messina | Patients with stage 1-5 or A1-A3 CKD or with other anatomic or urine sediment abnormalities + Hyperkalaemia | sK+ >5.1 mEq/L | NA | 90 | NR | Median (IQR): 76.0 (66.0 to 82.0) | NR |
|  |  |  |  | Patients with stage 1-5 or A1-A3 CKD or with other anatomic or urine sediment abnormalities + Normokalaemia | sK+ ≤5.1 mEq/L | NA | 180 | NR | Median (IQR): 76.0 (64.0 to 81.0) | NR |
| Zhang 2022a ([36](#_ENREF_36)) | Retrospective observational study | China | Urban Employee Basic Medical Insurance claims | Patients with CKD having ≥1 inpatient or outpatient visit + Hyperkalaemia | sK+ >5.0 mmol/L | NA | 1,015 | Male: 59.2 Female 40.8 | Mean (SD): 67.3 (14.4) | NR |
|  |  |  |  | Patients with CKD having ≥1 inpatient or outpatient visit + Normokalaemia | NR | NA | 25,981 | Male: 63.9 Female 36.1 | Mean (SD): 66.0 (13.3) | NR |
| Sharma 2021 ([120](#_ENREF_120)) | Retrospective observational study | USA | USA health plan | Patients with stage 1-5 CKD and unknown stage CKD + Hyperkalaemia | Two or more K+ lab tests >5.0 mEq/L on different dates; OR Two or more diagnosis codes of hyperkalaemia (ICD-9 code of 276.7 or one ICD-10 diagnostic code of E87.5); OR One diagnosis code of hyperkalaemia and one lab K+ value >5.0 mEq/L; OR Evidence of a National Drug Code number for either patiromer or SPS | NA | 79,084 | NR | NR | NR |
|  |  |  |  | Patients with stage 1-5 CKD and unknown stage CKD + Normokalaemia | sK+ 3.8 to 5.0 mEq/L | NA |  |  |  |  |
| Kohsaka 2021 ([86](#_ENREF_86)) | Retrospective observational study | Japan | Hospital-based cohort study. Data were obtained using a Japanese hospital claims registry, Medical Data Vision | Patients with stage 1-5 CKD + Hyperkalaemia | At least 2 sK+ readings of ≥5.1 mmol/L within a 12-month interval | NA | 16,133 | Male: 56.5 Female 43.5 | Mean (SD): 73.4 (12.6) | NR |
|  |  |  |  | Patients with stage 1-5 CKD + Normokalaemia | Without any record of sK+ levels of ≤3.5 mmol/L and ≥5.1 mmol/L | NA | 11,898 | Male: 57.1 Female 42.9 | Mean (SD): 66.6 (14.3) | NR |
| Grandy 2021 ([37](#_ENREF_37)) | Retrospective observational study | Multinational | Adelphi Real World CKD Disease Specific Programme™ | Patients with stage 3-4 non-dialysis dependent CKD + Hyperkalaemia | sK+ >5.0 mmol/L | NA | 216 | Male: 57.4 Female 42.6 | Mean (SD): 63.3 (15.1)  Median (range): 66.0 (18.0 to 90.0) | White: 60.2 Black: 7.9 Asian: 25.0 Hispanic/Latino: 2.8 Other: 4.2 |
|  |  |  |  | Patients with stage 3-4 non-dialysis dependent CKD + Normokalaemia | sK+ 3.5 to 5.0 mmol/L | NA | 933 | Male: 60.1 Female 39.9 | Mean (SD): 63.7 (13.1)  Median (range): 65.0 (19.0 to 90.0) | White: 62.9 Black: 6.1 Asian: 24.5 Hispanic/Latino: 2.8 Other: 3.7 |
| Sharma 2020 ([38](#_ENREF_38)) | Retrospective observational study | USA | Data included medical and pharmacy claims and lab test results of deidentified individuals continuously enrolled from January 2016 through December 2017 in  a fully insured commercial or Medicare Advantage plan operated by a large national health plan. | Patients with stage 1-5 CKD + Hyperkalaemia | 1. ≥2 sK+ >5.0 mmol/L on different dates (Logical Observation Identifiers Names and Codes) 2. ≥2 claims with principal or secondary diagnosis of hyperkalaemia (ICD-10) 3. 1 claim with hyperkalaemia and 1 K+ >5.0 mmol/L 4. ≥1 dispensed prescription for SPS or patiromer | NA | 6,235 | Male: 52.6 Female 47.4 | Mean (SD): 69.3 (11.8)  Median (range): 71.0 (62.0 to 77.0) | NR |
|  |  |  |  | Patients with stage 1-5 CKD + Normokalaemia | NR | NA | 429,277 | Male: 42.6 Female 57.4 | Mean (SD): 61.1 (16.3)  Median (range): 63.0 (50.0 to 73.0) | NR |
| Kanda 2020 ([87](#_ENREF_87)) | Retrospective observational study | Japan | Data were obtained using a Japanese hospital claims registry, Medical Data Vision | Patients with stage 1-5 CKD + Hyperkalaemia | sK+ ≥5.1 mmol/L | NA | 16,133 | Male: 56.5 Female 43.5 | Mean (SD): 73.0 (13.0) | NR |
|  |  |  |  | Patients with stage 1-5 CKD + Normokalaemia | sK+ 3.5-5.1 mmol/L | NA | 15,287 | Male: 56.4 Female 43.6 | Mean (SD): 68.0 (14.0) | NR |
| Jimenez-Marrero 2020 ([39](#_ENREF_39)) | Retrospective observational study | Spain | Data were obtained using a Japanese hospital claims registry, Medical Data Vision | Patients with CKD + HF + Hyperkalaemia | sK+ >5.0 mEq/L | NA | 2,917 | NR | NR | NR |
|  |  |  |  | Patients with CKD + HF + Hypokalaemia | sK+ <3.5 mEq/L. | NA | 3,146 | NR | NR | NR |
| Betts 2020 ([121](#_ENREF_121)) | Retrospective observational study | USA | US insurance claims database: 1. IBM MarketScan Commercial 2. Medicare-Supplemental Claims database | CKD patients with hyperkalaemia-related hospitalisations | sK+ >5.0 mEq/L | NA | 1,809 | NR | NR | NR |
|  |  |  |  | CKD patients with a hospitalisation without evidence of hyperkalaemia (normokalaemia) | sK+ <5.0 mEq/L | NA | 1,809 | NR | NR | NR |
| Mu 2020 ([122](#_ENREF_122)) | Retrospective observational study | USA | Medicare claims database | Patients with CKD + Hyperkalaemia | NR | NA | 26,809 | NR | NR | NR |
|  |  |  |  | Patients CKD + Normokalaemia | NR | NA | 26,809 | NR | NR | NR |
| DISCOVER CKD  James 2021 ([40](#_ENREF_40))  **Linked study**: James 2022 ([123](#_ENREF_123)) | Retrospective observational study | Multinational | 1. USA TriNetX hospital-EMR 2. UK CPRD linked to hospital data 3. USA DOPPS 4. JMDV databases | Patients with stage 3-5 CKD including renal replacement therapy or two eGFR measures <60 mL/min/1.73 m2 between 90-730 days apart + Hyperkalaemia | sK+ >5.0 mmol/L | NA | 125,196 | Male: 51.5 Female 48.5 | Mean (SD): 68.0 (14.1) | NR |
|  |  |  |  | Patients with stage 3-5 CKD including renal replacement therapy or two eGFR measures <60 mL/min/1.73 m2 between 90-730 days apart + Normokalaemia | sK+ <5.0 mmol/L | NA | 1,672,595 | Male: 42.4 Female 57.6 | Mean (SD): 63.6 (14.0) | NR |
| Thomsen 2018 ([124](#_ENREF_124)) | Retrospective observational study | Denmark | Danish National Patient Registry | Patients with stage 1-5 CKD or on dialysis + Hyperkalaemia | sK+ >5.0 mmol/L | NA | 43,397 | Male: 48.8 Female 51.2 | Median (IQR): 76.0 (67.4 to 83.5) | NR |
|  |  |  |  | Patients with stage 1-5 CKD or on dialysis + Normokalaemia | NR | NA | 43,397 | Male: 48.8 Female 51.2 | Median (IQR): 76.0 (67.4 to 83.5) | NR |
| Furuland 2018 ([108](#_ENREF_108)) | Retrospective observational study | UK | CPRD and linked Hospital Episode Statistics databases | Pre-dialysis patients with stage 3-5 CKD + Hyperkalaemia | sK+ 5.0 to <5.5 mmol/L | NA | 18,454 | Male: 44.0 Female 56.0 | Mean (SD): 72.5 (11.8) | NR |
|  |  |  |  | Pre-dialysis patients with stage 3-5 CKD + Hyperkalaemia | sK+ 5.5 to <6.0 mmol/L | NA | 4,250 | Male: 46.8 Female 53.2 | Mean (SD): 72.7 (11.9) | NR |
|  |  |  |  | Pre-dialysis patients with stage 3-5 CKD + Hyperkalaemia | sK+ ≥6.0 mmol/L | NA | 1,026 | Male: 46.8 Female 53.2 | Mean (SD): 72.0 (13.5) | NR |
|  |  |  |  | Pre-dialysis patients with stage 3-5 CKD + Hypokalaemia | sK+ <3.5 mmol/L | NA | 3,635 | Male: 31.5 Female 68.5 | Mean (SD): 73.5 (13.1) | NR |
|  |  |  |  | Pre-dialysis patients with stage 3-5 CKD + Normokalaemia | sK+ 3.5 to <4.0 mmol/L | NA | 17,662 | Male: 36.0 Female 64.0 | Mean (SD): 72.5 (12.6) | NR |
|  |  |  |  | Pre-dialysis patients with stage 3-5 CKD + Normokalaemia | sK+ 4.0 to <4.5 mmol/L | NA | 50,065 | Male: 37.9 Female 62.1 | Mean (SD): 71.6 (12.4) | NR |
|  |  |  |  | Pre-dialysis patients with stage 3-5 CKD + Normokalaemia | sK+ 4.5 to <5.0 mmol/L | NA | 48,543 | Male: 41.1 Female 58.9 | Mean (SD): 72.0 (12.1) | NR |
| Neuenschwander 2023 ([116](#_ENREF_116)) | Retrospective observational study | USA | Medicare Free-For Service claims database | Patients with CKD + Hyperkalaemia | Hyperkalaemia was defined as ICD-10 diagnostic code of E87.5 in any claim position or Medicare Part D fill for oral K+ binder use within 14 days prior to long-term care stay or during long-term care stay | NA | 145,315 | NR | NR | NR |
|  |  |  |  | Patients with CKD + Normokalaemia | Non-hyperkalaemia was defined as no evidence of hyperkalaemia within 14 days prior to long-term care stay or during long-term care stay | NA | 650,228 | NR | NR | NR |
| Betts 2018 ([125](#_ENREF_125)) | Retrospective observational study | USA | Truven MarketScan claims database | Patients with stage 3-5 CKD and unspecified stage + Hyperkalaemia | sK+ >5.0 mEq/L | NA | 9,620 | NR | NR | NR |
|  |  |  |  | Patients with stage 3-5 CKD and unspecified stage + Normokalaemia | sK+ <5.0 mEq/L | NA | 9,620 | NR | NR | NR |
| Luo 2016 ([109](#_ENREF_109)) | Retrospective observational study | USA | Electronic health records of HealthCare Partners | Patients with CKD with eGFR <60 mL/min/1.73 m2 + Hyperkalaemia | sK+ 5.0 to 5.4 mEq/L | NA | 8,270 | Male: 47.8 Female 52.2 | Mean (SD): 74.7 (11.3) | White: 22.9 Black: 3.2 Asian: 3.0 American Indian/Alaskan native: 0.4 Native Hawaiian/Pacific Islander: 0.3 Unknown: 62.6 |
|  |  |  |  |  | sK+ 5.5 to 5.9 mEq/L | NA | 2,187 | Male: 49.0 Female 51.0 | Mean (SD): 74.3 (11.5) | White: 22.4 Black: 2.3 Asian: 3.0 American Indian/Alaskan native: 0.1 Native Hawaiian/Pacific Islander: 0.6 Unknown: 62.6 |
|  |  |  |  |  | sK+ ≥6.0 mEq/L | NA | 589 | Male: 54.3 Female 45.7 | Mean (SD): 73.9 (12.3) | White: 20.7 Black: 2.4 Asian: 2.9 American Indian/Alaskan native: 0.5 Native Hawaiian/Pacific Islander: 0.2 Unknown: 62.5 |
|  |  |  |  | Patients with CKD with eGFR <60 mL/min/1.73 m2 + Normokalaemia | sK+ 3.5 to 3.9 mEq/L | NA | 5,664 | Male: 32.4 Female 67.6 | Mean (SD): 73.8 (11.5) | White: 21.0 Black: 8.2 Asian: 3.4 American Indian/Alaskan native: 0.2 Native Hawaiian/Pacific Islander: 0.4 Unknown: 58.7 |
|  |  |  |  |  | sK+ 4.0 to 4.4 mEq/L | NA | 18,712 | Male: 37.8 Female 62.2 | Mean (SD): 74.6 (11.3) | White: 21.3 Black: 5.3 Asian: 3.6 American Indian/Alaskan native: 0.2 Native Hawaiian/Pacific Islander: 0.3 Unknown: 61.8 |
|  |  |  |  |  | sK+ 4.5 to 4.9 mEq/L | NA | 18,947 | Male: 44.1 Female 55.9 | Mean (SD): 74.8 (11.1) | White: 22.5 Black: 3.7 Asian: 3.0 American Indian/Alaskan native: 0.2 Native Hawaiian/Pacific Islander: 0.3 Unknown: 62.4 |
|  |  |  |  | Patients with CKD with eGFR <60 mL/min/1.73 m2 + Hypokalaemia | sK+ <3.5 mEq/L | NA | 897 | Male: 33.4 Female 66.6 | Mean (SD): 72.2 (11.9) | White: 21.7 Black: 9.6 Asian: 3.5 American Indian/Alaskan native: 0.1 Native Hawaiian/Pacific Islander: 0.6 Unknown: 57.6 |
| Nakhoul 2015 ([90](#_ENREF_90)) | Retrospective observational study | USA | Electronic health record-based CKD registry | Patients with stage 3-4 non-dialysis dependent CKD + Hyperkalaemia | sK+ 5.0 to 5.4 mmol/L | NA | 3,931 | Male: 52.7 Female 47.3 | Mean (SD): 71.9 (12.1) | Black: 11.2 |
|  |  |  |  |  | sK+ ≥5.5 mmol/L | NA | 1,213 | Male: 53.2 Female 46.8 | Mean (SD): 70.4 (12.9) | Black: 19.3 |
|  |  |  |  | Patients with stage 3-4 non-dialysis dependent CKD + Normokalaemia | sK+ 3.5 to 3.9 mmol/L | NA | 5,392 | Male: 37.1 Female 62.9 | Mean (SD): 72 (11.9) | Black: 18.1 |
|  |  |  |  |  | sK+ 4.0 to 4.9 mmol/L | NA | 24,717 | Male: 45.8 Female 54.2 | Mean (SD): 72.7 (11.7) | Black: 11.6 |
|  |  |  |  | Patients with stage 3-4 non-dialysis dependent CKD + Hypokalaemia | sK+ <3.5 mEq/L | NA | 1,106 | Male: 33.5 Female 66.5 | Mean (SD): 68.7 (13.4) | Black: 26.3 |
| Integrated CKD care program Kaohsiung for delaying Dialysis  Wang 2013 ([41](#_ENREF_41)) | Prospective observational study | Taiwan | The study recruited patients from the nephrology out-patient departments of two hospitals in southern Taiwan | Patients with stage 1-4 CKD not on renal replacement therapy + Hyperkalaemia | sK+ >5.0 mEq/L | NA | 175 | Male: 66.9 Female 33.1 | Mean (SD): 63.8 (14.7) | NR |
|  |  |  |  | Patients with stage 1-4 CKD not on renal replacement therapy + Normokalaemia | sK+ 3.5 to 4 mEq/L | NA | 683 | Male: 63.3 Female 36.7 | Mean (SD): 61.3 (15) | NR |
|  |  |  |  | Patients with stage 1-4 CKD not on renal replacement therapy + Normokalaemia | sK+ 4.0 to 4.5 mEq/L | NA | 974 | Male: 63.7 Female 36.3 | Mean (SD): 62.7 (14.1) | NR |
|  |  |  |  | Patients with stage 1-4 CKD not on renal replacement therapy + Hypokalaemia | sK+ 4.5 to 5 mEq/L | NA | 474 | Male: 65.7 Female 34.3 | Mean (SD): 63.1 (14.3) | NR |
|  |  |  |  | Patients with stage 1-4 CKD not on renal replacement therapy + Hypokalaemia | sK+ <3.5 mEq/L | NA | 194 | Male: 62.4 Female 37.6 | Mean (SD): 62.1 (14.9) | NR |
| Hayes 2012 ([61](#_ENREF_61)) | Retrospective observational study | USA | Nephrology Department at Salem Veteran Affairs Medical Center | Patients with moderate and advanced non-dialysis-dependent CKD + Hyperkalaemia | sK+ >5.3 mEq/L | NA | 95 | Male: 100 Female 0 | Mean (SD): 68.2 (11.0) | Black: 25.0 |
|  |  |  |  | Patients with moderate and advanced non-dialysis-dependent CKD + Normokalaemia | sK+ 3.6 to 4.5 mEq/L | NA | 557 | Male: 100 Female 0 | Mean (SD): 68.1 (11.2) | Black: 27.0 |
|  |  |  |  | Patients with moderate and advanced non-dialysis-dependent CKD + Normokalaemia | sK+ 4.5 to 5.3 mEq/L | NA | 536 | Male: 100 Female 0 | Mean (SD): 69.0 (10.4) | Black: 20.0 |
|  |  |  |  | Patients with moderate and advanced non-dialysis-dependent CKD + Hypokalaemia | sK+ <3.6 mEq/L | NA | 39 | Male: 100 Female 0 | Mean (SD): 65.6 (10.5) | Black: 31.0 |
| Jain 2012 ([42](#_ENREF_42)) | Retrospective observational study | USA | Veterans Affairs North Texas Health Care System electronic medical records | Patients with advanced (stage 3-5) CKD + Hyperkalaemia | sK+ >5.0 mEq/L | NA | 777 | NR | NR | NR |
|  |  |  |  | Patients with advanced (stage 3-5) CKD+ Normokalaemia | sK+ ≤5.0 mEq/L | NA | 607 | NR | NR | NR |
| NephroTest Wagner 2017 ([43](#_ENREF_43)) | Prospective observational study | France | Hospital-based cohort study | Patients with stage 1-5 non-dialysis-dependent CKD + Hyperkalaemia | sK+ >5.0 mmol/L | NA | 172 | Male: 72.7 Female 27.3 | Mean (SD): 56.9 (15.5) | Black: 12.2 |
|  |  |  |  | Patients with stage 1-5 non-dialysis-dependent CKD + Normokalaemia | sK+ 4.0 to 5.0 mmol/L | NA | 1,340 | Male: 67.9 Female 32.1 | Mean (SD): 60.2 (14.8) | Black: 11.0 |
|  |  |  |  | Patients with stage 1-5 non-dialysis-dependent CKD + Hypokalaemia | sK+ <4.0 mmol/L | NA | 566 | Male: 60.7 Female 39.3 | Mean (SD): 56.0 (15.6) | Black: 18.0 |
| Collins 2017 ([115](#_ENREF_115)) | Retrospective observational study | USA | The study utilised de-identified EMR data on approximately 7 million patients collected from multiple USA integrated health delivery networks | Patients with stage 3-5 CKD + Hyperkalaemia | sK+ 5.0 to <5.5 mEq/L | NA | 9,021 | NR | NR | NR |
|  |  |  |  |  | sK+ 5.5 to <6.0 mEq/L | NA | 1,703 | NR | NR | NR |
|  |  |  |  |  | sK+ 6.0 to <6.5 mEq/L | NA | 266 | NR | NR | NR |
|  |  |  |  |  | sK+ 6.5 to 8.0 mEq/L | NA | 224 | NR | NR | NR |
|  |  |  |  | Patients with stage 3-5 CKD + Normokalaemia | sK+ 4.0 to <4.5 mEq/L | NA | 36,208 | NR | NR | NR |
|  |  |  |  |  | sK+ 4.5 to <5.0 mEq/L | NA | 26,731 | NR | NR | NR |
|  |  |  |  | Patients with stage 3-5 CKD + Hypokalaemia | sK+ 2.5 to <3. 0 mEq/L | NA | 257 | NR | NR | NR |
|  |  |  |  |  | sK+ 3.0 to <3.5 mEq/L | NA | 3,397 | NR | NR | NR |
|  |  |  |  |  | sK+ 3.5 to 4.0 mEq/L | NA | 18,968 | NR | NR | NR |
| RRI-CKD Cohort study Korgaonkar 2010 ([91](#_ENREF_91)) | Prospective observational study | USA | The Renal Research Institute CKD Study was conducted at four outpatient nephrology clinics in the USA | Patients with stage 3-5 CKD + Hyperkalaemia | sK+ ≥5.5 mmol/L | NA | 65 | Male: 77.0 Female 23.0 | Mean (SD): 58.4 (14) | Black: 20.0 |
|  |  |  |  | Patients with stage 3-5 CKD + Normokalaemia | sK+ >4.0 and <5.5 mmol/L | NA | 633 | Male: 55.0 Female 45.0 | Mean (SD): 60.7 (15.4) | Black: 19.0 |
|  |  |  |  | Patients with stage 3-5 CKD + Hypokalaemia | sK+ ≤4.0 mmol/L | NA | 122 | Male: 43.0 Female 57.0 | Mean (SD): 60.6 (16.1) | Black: 30.0 |
| Hwang 2011 ([66](#_ENREF_66)) | Retrospective observational study | Taiwan | All the data were recorded from medical charts | Patients with ESRD under maintenance haemodialysis + Hyperkalaemia | sK+ >5.5 mEq/L | NA | 51 | Male: 59.0 Female 41.0 | Mean (SD): 58.0 (10.0); range: 37.0 to 78.0 | NR |
|  |  |  |  | Patients with ESRD under maintenance haemodialysis + Normokalaemia | sK+ 3.5 to 5.5 mEq/L | NA | 326 | Male: 50.0 Female 50.0 | Mean (SD): 58.0 (13.0); range: 19.0 to 87.0 | NR |
|  |  |  |  | Patients with ESRD under maintenance haemodialysis + Hypokalaemia | sK+ <3.5 mEq/L | NA | 46 | Male: 36.0 Female 64.0 | Mean (SD): 63.0 (14.0); range: 25.0 to 86.0 | NR |
| Brookes 2021 ([110](#_ENREF_110)) | Retrospective observational study | Australia | This hospital-based cohort study used Austin Health EMR data extracted from The DARE Centre | Patients with stage 1-5 non-dialysis dependent CKD + Hyperkalaemia | sK+ ≥6.0 mmol/L | NA | 297 | Male: 59.9 Female 40.1 | Mean (SD): 75.4 (12.8)  Median (IQR): 78.0 (67.0 to 84.0) | NR |
|  |  |  |  |  | sK+ 5.5 to 5.9 mmol/L | NA | 473 | Male: 60.9 Female 39.1 | Mean (SD): 76.8 (12.9)  Median (IQR): 79.0 (69.0 to 87.0) | NR |
|  |  |  |  | Patients with stage 1-5 non-dialysis dependent CKD + Normokalaemia | sK+ 5.0 to 5.4 mmol/L | NA | 1,503 | Male: 57.1 Female 42.9 | Mean (SD): 77.7 (11.9)  Median (IQR): 80.0 (71.0 to 86.0) | NR |
|  |  |  |  |  | sK+ 4.0 to 4.9 mmol/L | NA | 7,235 | Male: 48.9 Female 51.1 | Mean (SD): 77.6 (11.7)  Median (IQR): 80.0 (71.0 to 86.0) | NR |
|  |  |  |  |  | sK+ 3.5 to 3.9 mmol/L | NA | 1,341 | Male: 39.3 Female 60.7 | Mean (SD): 76.6 (12.5)  Median (IQR): 79.0 (70.0 to 86.0) | NR |
|  |  |  |  | Patients with stage 1-5 non-dialysis dependent CKD + Hypokalaemia | sK+ <3.5 mmol/L | NA | 307 | Male: 30.3 Female 69.7 | Mean (SD): 72.9 (14.8)  Median (IQR): 76.0 (65.0 to 84.0) | NR |
| Shang 2022 ([35](#_ENREF_35)) | Retrospective observational study | China | This study used data from patients hospitalised with HF, integrating electronic medical records from the PhysioNet restricted health data database and external outcome data | Patients with CKD + HF + Hyperkalaemia | sK+ >4.7 mmol/L | NA | 53 | NR | NR | NR |
|  |  |  |  | Patients with CKD + HF + Normokalaemia | sK+ 3.5 to 4.7 mmol/L | NA | 145 | NR | NR | NR |
|  |  |  |  | Patients with CKD + HF + Hypokalaemia | sK+ <3.5 mmol/L | NA | 26 | NR | NR | NR |
| Jime´nez-Marrero 2021 ([44](#_ENREF_44)) | Retrospective observational study | Spain | This study used administrative, hospital and primary care databases | Patients with CKD + Hyperkalaemia | sK+ >5.0 mEq/L | NA | 815 | NR | NR | NR |
|  |  |  |  | Patients with CKD + Normokalaemia | sK+ ≥3.5 and ≤5 mEq/L | NA | 12,491 | NR | NR | NR |
|  |  |  |  | Patients with CKD + Hypokalaemia | sK+ <3.5 mEq/L | NA | 170 | NR | NR | NR |
| Fukushima CKD Cohort Tanaka 2021 ([80](#_ENREF_80)) | Prospective observational study | Japan | The Fukushima CKD Cohort Study was a prospective survey to investigate the characteristics and outcomes of pre-dialysis patients with CKD at Fukushima Medical University Hospital | Pre-dialysis CKD patients with eGFR <60 mL/min/1.73 m2 + Hyperkalaemia | sK+ ≥5.0 mmol/L | NA | 141 | Male: 63.1 Female 36.9 | Median (IQR): 69 (62.0 to 75.0) | NR |
|  |  |  |  | Pre-dialysis CKD patients with eGFR <60 mL/min/1.73 m2 + Normokalaemia | sK+ 4.5 to 4.9 mmol/L | NA | 433 | Male: 60.3 Female 39.7 | Median (IQR): 67.0 (59.5 to 76.0) | NR |
|  |  |  |  |  | sK+ 4.0 to 4.4 mmol/L | NA | 539 | Male: 56.2 Female 43.8 | Median (IQR): 65.0 (53.0 to 74.0) | NR |
|  |  |  |  | Pre-dialysis CKD patients with eGFR <60 mL/min/1.73 m2 + Hypokalaemia | sK+ <4.0 mmol/L | NA | 217 | Male: 46.1 Female 53.9 | Median (IQR): 62.0 (50.0 to 72.0) | NR |
| Li 2023b ([67](#_ENREF_67)) | Retrospective observational study | China | Web-based Electronic-Medical-Records system in hospital database | Patients with stage 3-5 CKD + HFpEF | sK+ >5.5 mmol/L | MRA (spironolactone) | 154 | Male: 53.9 Female 46.1 | Mean (SD): 76.2 (9.3) | NR |
|  |  |  |  |  |  | No Spironolactone | 233 | Male: 56.2 Female 43.8 | Mean (SD): 74.1 (10.4) | NR |
| Kanda 2023 ([117](#_ENREF_117)) | Retrospective observational study | Japan | Optum’s de-identified Market Clarity Data | Patients with stage 3 or 4 CKD with or without HF | ICD-10 diagnostic code of E87.5 or ICD-9 diagnostic code of 276.7 | ACEi/ARB/ARNi/MRA | 1,427 | Male: 65.7 Female 34.3 | Mean (SD): 76.3 (11.5)  Median (IQR): 78.0 (71.0 to 84.0) | NR |
|  |  | USA | Medical Data Vision |  |  |  | 11,873 | Male: 51.9 Female 48.1 | Mean (SD): 71.2 (12.1)  Median (IQR): 73.0 (63.0 to 81.0) | NR |
| Lin 2023 ([74](#_ENREF_74)) | Retrospective observational study | Taiwan | Taiwan National Health Insurance Research Database | Patients with ESRD under maintenance dialysis + HF | ICD-9 diagnostic code of 276.7 or sK+ >6 mEq/L | MRA | 2,176 | Male: 50.3 Female 49.7 | Mean (SD): 64.7 (13.2) | NR |
|  |  |  |  |  |  | Non-MRA | 6,528 | Male: 50.2 Female 49.8 | Mean (SD): 64.8 (13.4) | NR |
| MRA-ACE Trial Tumlin 2022 ([21](#_ENREF_21)) | RCT | USA | NA | Patients with diabetic kidney disease | sK+ >5.5 mmol/L | Maximum tolerated ACE/ARB | 18 | Male: 47.1 Female 52.9 | 63.0 | NR |
|  |  |  |  |  |  | Maximum tolerated ACE/ARB + MRA (spironolactone -25 mg) | 14 | Male: 62.5 Female 37.5 | 61.3 | NR |
| Sadjadi 2009 ([45](#_ENREF_45)) | Retrospective observational study | USA | Electronic medical records | Patients with stage 1-5 CKD | sK+ >5.0 mEq/L | ACEi (lisinopril/benazepril/fosinopril/enalapril/captopril) | 1,163 | NR | Mean (SD): 67.5 (10.9) | NR |
|  |  |  |  |  |  | ARB (irbesartan/losartan) | 1,168 | NR | Mean (SD): 67.2 (10.7) | NR |
| Khosla 2009 ([68](#_ENREF_68)) | Retrospective observational study | USA | University-based HT clinics | Patients with stage 2 or 3 CKD + Resistant HT | Persistent sK+ >5.5 mEq/L or any single reading sK+ ≥6.0 mEq/L | ACEi (lisinopril/ramipril) or ARB (valsartan/olmesartan) or ACEi + ARB | 46 | Male: 65.2 Female 34.8 | Mean (SD): 64.9 (10.7) | Black: 82.0 |
| AASK Weinberg 2009 ([22](#_ENREF_22)) | RCT | USA | NA | Patients with non-diabetic hypertensive CKD (eGFR 20-65 mL/min/1.73 m2) | sK+ >5.5 mEq/L | ACEi | 417 | Male: 61.2 Female 38.8 | Mean (SD): 54.3 (10.9) | NR |
|  |  |  |  |  |  | β-Blocker | 428 | Male: 61.2 Female 38.8 | Mean (SD): 55.0 (10.3) | NR |
|  |  |  |  |  |  | CCB | 208 | Male: 60.1 Female 39.9 | Mean (SD): 54.3 (10.8) | NR |
| Knoll 2002 ([92](#_ENREF_92)) | Prospective observational study | Canada | The Ottawa Hospital, Ontario | Patients with ESRD under chronic haemodialysis | sK+ ≥5.5 mmol/L or sK+ ≥6.0 mmol/L | ACEi (enalapril/fosinopril/captopril) or ARB (losartan) | 71 | Male: 62.0 Female 38.0 | Mean (SD): 56.6 (17.7) | NR |
|  |  |  |  |  |  | Control | 180 | Male: 62.0 Female 38 | Mean (SD): 62.4 (16.8) | NR |
| Hayashi 2003 ([23](#_ENREF_23)) | RCT | Japan | NA | Patients with chronic renal parenchymal disease + HT | NR | ACEi (enalapril/lisinopril/imidapril) | 20 | Male: 60.0 Female 40.0 | Mean (SD): 57 (13.4) | NR |
|  |  |  |  |  |  | CCB (efonidipine) | 23 | Male: 78.3 Female 21.7 | Mean (SD): 58 (14.4) | NR |
| Adelborg 2019 ([46](#_ENREF_46)) | Retrospective observational study | Denmark | The Danish National Patient Registry | Patients with CKD | sK+ >5.0 mmol/L | ACEi/ARB/MRA (spironolactone) | NR | NR | NR | NR |
| Sengul 2009 ([93](#_ENREF_93)) | Single-arm trial | Turkey | NA | Patients with CKD | sK+ ≥5.5 mEq/L | MRA (spironolactone) | 33 | Male: 51.5 Female 48.5 | Mean (SD): 45.2 (14.4) | NR |
| Abolghasmi 2011 ([24](#_ENREF_24)) | RCT | Iran | NA | Patients with moderately severe CKD (eGFR 25-50 mL/min/1.73m²) + Resistant HT | sK+ >5.5 mEq/L | MRA (spironolactone) | 19 | Male: 52.6 Female 47.4 | Mean (SD): 49.0 (13.2) | NR |
|  |  |  |  |  |  | Control | 22 | Male: 54.5 Female 45.5 | Mean (SD): 50.0 (10.1) | NR |
| Fröhlich 2016 ([69](#_ENREF_69)) | Retrospective observational study | UK, Germany | HF clinics of the Department of Academic Cardiology, University of Hull, UK and University Hospital Heidelberg, Germany | Patients with stable stage 3 or 4 CKD + HF | sK+ >5.5 mmol/L | ACEi/ARB | 722 | Male: 68.0 Female 32.0 | Mean (SD): 69.0 (11.0) | NR |
| EMPHASIS-HF Eschalier 2013 ([25](#_ENREF_25)) | RCT | Multinational | NA | Patients with CKD with eGFR <60 mL/min/1.73 m2 | Mild hyperkalaemia: sK+ >5.5 mmol/L; Severe hyperkalaemia: sK+ >6.0 mmol/L | MRA (eplerenone) | 439 | Male: 72.9 Female 27.1 | Mean (SD): 71.1 (7.5) | NR |
|  |  |  |  |  |  | Placebo | 473 | NR | NR | NR |
| Polson 2017 ([118](#_ENREF_118)) | Retrospective observational study | USA | Regional health plans | Patients with CKD | ICD-10 diagnostic code of E87.5 or ICD-9 diagnostic code of 276.7 | RAASi | 2,774 | Male: 53.1 Female 46.9 | Mean (SD): 69.3 (12.7  Median: 71.0 | NR |
|  |  |  |  |  |  | No RAASi | 3,423 | Male: 53.8 Female 46.2 | Mean (SD): 68.9 (13.4)  Median: 70.0 | NR |
|  |  |  |  | Patients with CKD + HF |  | RAASi | 691 | Male: 56.9 Female 43.1 | Mean (SD): 73.7 (11.5)  Median: 76.0 | NR |
|  |  |  |  |  |  | No RAASi | 986 | Male: 58.1 Female 41.9 | Mean (SD): 74.1 (11.5)  Median: 75.0 | NR |
| Wetmore 2021 ([75](#_ENREF_75)) | Retrospective observational study | UK | Clinical Practice Research Datalink (Hospital Episodes Statistics linked database) | Patients with stage 1-5 CKD with previous history of hyperkalaemia | Mild (sK+ 5.0 to ≤5.5 mmol/L), moderate (sK+ >5.5 to ≤6.0 mmol/L) or severe hyperkalaemia (sK+ >6.0 mmol/L) | RAASi | 46,733 | NR | NR | NR |
|  |  |  |  | Patients with stage 1-5 CKD without previous history of hyperkalaemia |  |  | 39,814 | NR | NR | NR |
| Yang 2023 ([94](#_ENREF_94)) | Retrospective observational study | Hong Kong | Hong Kong-DM Surveillance-Database | Patients with advanced CKD + DM | pK+ ≥5.5 mmol/L | Continued RAASi users | 3,817 | Male: 51.2 Female 48.8 | Mean (SD): 69.3 (11.2) | NR |
|  |  |  |  |  |  | Discontinued RAASi users | 583 | Male: 59.5 Female 40.5 | Mean (SD): 70.3 (12.0) | NR |
| Buckallew 2021 ([95](#_ENREF_95)) | Retrospective observational study | USA | Electronic medical records | Patients with stage 3-5 CKD + HF | sK+ ≥5.5 mEq/L | MRA (spironolactone) | 121 | Male: 48.8 Female 51.2 | 74.2 | White: 73.6 Black: 24.8 Other:3.2 |
| De Rosa 2002 ([76](#_ENREF_76)) | Single-arm trial | Italy | NA | Patients with mild (CrCL 30-60 mL/min/1.73 m2) or moderate to severe (CrCL 10-29 mL/min/1.73 m2) chronic renal insufficiency + HT | sK+ >6.0 mEq/L | ARB (irbesartan with or without concomitant antihypertensive therapy) | 32 | Male: 65.0 Female 34.0 | Mean (SD): 70.4 (4.8) | NR |
| Ren 2022 ([96](#_ENREF_96)) | Retrospective observational study | Canada | 1. The Manitoba Health Insurance Registry  2. Medical Services and Claims  3. Canadian Institute for Health Information Discharge Abstract Database  4. Shared Health Diagnostic Services  5. Drug Program Information Network and Vital Statistics | Patients with stage 3-5 CKD + de novo hyperkalaemia episode | sK+ ≥5.5 mmol/L | RAASi | 10,009 | Male: 51.5 Female 48.5 | Mean (SD): 74.5 (12.8) | NR |
| Riccio 2022 ([70](#_ENREF_70)) | Retrospective observational study | Italy | Department of Nephrology of University “Federico II” of Naples | Patients with stage 1-5 CKD | sK+ >5.5 mmol/L | ACEi/ ARB/ ACEi + ARB combination | 556 | Male: 53.6 Female 46.4 | Median (IQR): 59.5 (26.0) | NR |
| EX-DKD Study Uchida 2022 ([97](#_ENREF_97)) | Single-arm trial | Japan | NA | Patients with diabetic kidney disease + HT | sK+ ≥5.5 mEq/L or sK+ ≥6.0 mEq/L | MRA (esaxerenone) | 109 | Male: 54.1 Female 45.9 | Mean (SD): 72.6 (7) | NR |
| Santoro 2022 ([98](#_ENREF_98)) | Retrospective observational study | Italy | Administrative and laboratory databases of five Local Health Units | Patients with stage ≤3 and >3 CKD and unspecified stage + Hyperkalaemia | sK+ ≥5.5 mmol/L | RAASi | 1,071 | Male: 58.3 Female 41.7 | Mean (SD): 72.8 (13.8) | NR |
| DRINK Tang 2021 ([11](#_ENREF_11)) | RCT | China (Hong Kong) | NA | Patients with stage 3 or 4 non-diabetic CKD | pK+ ≥5.5 mmol/L | ARB (losartan) + DRI (aliskiren) | 37 | Male: 73.0 Female 27.0 | Mean (SD): 55.1 (11.1) | NR |
|  |  |  |  |  |  | ARB (losartan) | 39 | Male: 61.5 Female 38.5 | Mean (SD): 55 (9.4) | NR |
| BLOCK-CKD Bakris 2021 ([12](#_ENREF_12)) | RCT | USA | NA | Patients with stage 3b or 4 CKD + Uncontrolled grade 1 and 2 systolic HT | sK+: 5.6 to 5.9 mmol/L or sK+ ≥5.6 mmol/L or sK+ ≥6.0 mmol/L | MRA (KBP-5074 – 0.25 mg) | 51 | Male: 47.1 Female 52.9 | Mean (SD): 65.4 (10.76) | NR |
|  |  |  |  |  |  | MRA (KBP-5074 – 0.5 mg) | 54 | Male: 57.4 Female 42.6 | Mean (SD): 64.8 (13.04) | NR |
|  |  |  |  |  |  | Placebo | 57 | Male: 59.6 Female 40.4 | Mean (SD): 65.9 (10.64) | NR |
| Einhorn 2009 ([111](#_ENREF_111)) | Retrospective observational study | USA | Veterans’ Health Administration national health care system | Patients with stage 3-5 CKD | Moderate Hyperkalaemia: sK+ ≥5.5 mg/dL to 6.0 mg/dL; Severe hyperkalaemia: sK+ ≥6.0 mg/dL | RAASi/No RAASi | 70,873 | Male: 96.8 Female 3.2 | Mean (SE): 73.0 (1.0) | White: 82.9 Black: 16 Other:1.1 |
| Obertynska 2021 ([112](#_ENREF_112)) | Retrospective observational study | NR | NR | Patients with stage 3 CKD + HFrEF <40% | Hyperkalaemia: sK+ 5.5 to 5.9 mmol/L Severe Hyperkalaemia: sK+ ≥6.0 mmol/L | MRA (spironolactone) | 208 | NR | NR | NR |
| Johnson 2010 ([99](#_ENREF_99)) | Retrospective observational study | USA | Administrative healthcare data from the Kaiser Permanente Northwest region | Patients with stage 3-5 CKD | sK+ ≥5.5 mmol/L or ICD-9 diagnostic code of 276.7 | ACEi (lisinopril) | 5,171 | Male: 37.2 Female 62.8 | Mean (SD): 71.1 (11.6) | NR |
| Raebel 2010 ([113](#_ENREF_113)) | Retrospective observational study | USA | Administrative healthcare data from Kaiser Permanente Colorado, HealthPartners Research Foundation, and Henry Ford Health System | Patients with stage 3-4 CKD + DM | sK+ ≥6.0 mmol/L or ICD-9 diagnostic code of 276.7 | ACEi/ARB/MRA (spironolactone) | 2,176 | NR | NR | NR |
| Frimodt-Moller 2010 ([13](#_ENREF_13)) | RCT | NR | NA | Pre-dialysis stage 3-5 CKD patients | pK+ >5.5mmol/L | ACEi (enalapril) + ARB (candesartan) after 16 weeks of monotherapy with either enalapril or candesartan | 47 | Male: 80.9 Female 19.1 | Mean (SD): 59 (11); range: 31 to 75 | White: 100 |
| Taheri 2012 ([14](#_ENREF_14)) | RCT | Iran | NA | Patients on chronic ambulatory peritoneal dialysis + Advanced HF | sK+ >5.7 mEq/L | MRA (spironolactone) | 9 | Male: 55.6 Female 44.4 | Mean (SD): 50.7 (17.4) | NR |
|  |  |  |  |  |  | Placebo | 9 | Male: 55.6 Female 44.4 | Mean (SD): 57.2 (13.1) | NR |
| Espinel 2012 ([15](#_ENREF_15)) | RCT | NR | NA | Patients with stage 3 CKD | sK+ ≥5.0 mmol/L | ARB (olmesartan) | 17 | Male: 52.9 Female 47.1 | Mean (SD): 60.2 (12.9) | White: 100 |
|  |  |  |  |  |  | ACEi (enalapril) | 13 | Male: 84.6 Female 15.4 | Mean (SD): 59.9 (11.6) | White: 100 |
| Boesby 2011 ([16](#_ENREF_16)) | RCT | Denmark | NA | Patients with stage 1-4 non-diabetic CKD | pK+ >5.5 mEq/L | MRA (eplerenone) vs control | 40 | Male: 67.5 Female 32.5 | Mean: 45.0; range: 21.0 to 71,0 | White: 92.5 African/Asian: 7.5 |
| CRIB-II study Edwards 2012 ([17](#_ENREF_17)) | RCT | UK | NA | Patients with stage 2-3 CKD | sK+ ≥5.5 mmol/L or sK+ ≥6.0 mmol/L | MRA (spironolactone) | 56 | Male: 57.0 Female 43.0 | Mean (SD): 54.0 (12.0) | NR |
|  |  |  |  |  |  | Placebo | 56 | Male: 59.0 Female 41.0 | Mean (SD): 53.0 (12.0) | NR |
| Tseng 2017 ([126](#_ENREF_126)) | Retrospective observational study | Taiwan | National Health Insurance Research Database | Pre-dialysis stage 5 CKD patients | NR | MRA (spironolactone) | 1,363 | Male: 50.3 Female 49.7 | Mean (SD): 68.0 (13.1) | NR |
|  |  |  |  |  |  | No spironolactone | 25,850 | Male: 46.8 Female 53.2 | Mean (SD): 65.0 (13.1) | NR |
| EVALUATE  Ando 2014 ([18](#_ENREF_18)) | RCT | Japan | NA | Patients with stage 1-3 non-diabetic CKD + HT | sK+ >5.5 mmol/L | MRA (eplerenone) | 162 | Male: 70.0 Female 30.0 | Mean (SD): 58.6 (13.0) | NR |
|  |  |  |  |  |  | Placebo | 152 | Male: 66.0 Female 34.0 | Mean (SD): 58.6 (13.8) | NR |
| Maddirala 2008 ([100](#_ENREF_100)) | Retrospective observational study | USA | Oklahoma regional VA hospitals | Patients with stage 1-5 CKD | sK+ ≥5.5 mEq/L | NA | 931 | Male: 98.2 Female 1.8 | Mean (SD): 63.9 (11.7) | White: 86.0 Black: 11.0 Native Americans: 1.9 Asian: 0.1 Hispanic: 1.0 |
| Rysava 2005 ([127](#_ENREF_127)) | Single-arm trial | Czech Republic. | NA | Patients with CKD + HT | NR | ARB (telmisartan) | 92 | Male: 48.9 Female 51.1 | Mean (SD): 56.7 (10.7); range: 22.0 to 79.0 | NR |
| FIGARO-DKD Pitt 2021 ([9](#_ENREF_9)) | RCT | Multinational | NA | Patients with stage 2-4 CKD with moderately elevated albuminuria or stage 1 or 2 CKD with severely elevated albuminuria + DM | NR | ARB (finerenone) | 3,686 | Male: 68.6 Female 31.4 | Mean (SD): 64.1 (9.7) | White: 72.5 Black: 3.1 Asian: 19.4 Other: 4.8 |
|  |  |  |  |  |  | Placebo | 3,666 | Male: 70.3 Female 29.7 | Mean (SD): 64.1 (10.0) | White: 71.1 Black: 4.0 Asian: 20.2 Other: 4.6 Missing data: 0.2 |
| FIDELIO-DKD Bakris 2020 ([10](#_ENREF_10))  **Linked studies:** Agarwal 2022 ([128](#_ENREF_128)) Zhang 2022b ([129](#_ENREF_129)) Filippatos 2021 ([130](#_ENREF_130)) Rossing 2022a ([131](#_ENREF_131)) Rossing 2022b ([132](#_ENREF_132))  Zhang 2023 ([133](#_ENREF_133)) | RCT | Multinational | NA | Patients with advanced CKD + DM | sK+ >5.5 mmol/L | ARB (finerenone) | 2,833 | Male: 68.9 Female 31.1 | Mean (SD): 65.4 (8.9) | White/Caucasians: 62.7 Black or African American: 4.9 Asian: 25.3 Other:7.0 |
|  |  |  |  |  |  | Placebo | 2,841 | Male: 71.5 Female 28.5 | Mean (SD): 65.7 (9.2) | White: 63.9 Black: 4.4 Asian: 25.4 Other:6.3 |
| Fang 2018 ([119](#_ENREF_119)) | Retrospective observational study | USA | Centers for Medicare & Medicaid Services Chronic Conditions Data Warehouse (Medicare service claims database), American Community Survey of the U.S. Census Bureau, and Multum Lexicon Drug database | Patients with CKD hospitalised for MI | Primary or secondary diagnosis code of 276.7 in inpatient claims | ACEi/ARB | 20,620 | Male: 43.1 Female 56.9 | NR | White: 80.9 Black: 11.7 Asian: 2.4 Hispanic: 3.1 Other: 2.0 |
| Silvarino 2019 ([106](#_ENREF_106)) | Retrospective observational study | Uruguay | Uruguayan National Renal Healthcare Program Registry | Patients with stage 1-5 non-dialysis dependent CKD | sK+ ≥5.6 mEq/L | ACEi | 221 | Male: 59.0 Female 41.0 | Mean (SD): 63.0 (15.4) | NR |
|  |  |  |  |  |  | ARB | 196 | Male: 57.0 Female 43.0 | Mean (SD): 67.2 (12.2) | NR |
|  |  |  |  |  |  | No ACEi/ARB | 703 | Male: 55.0 Female 45.0 | Mean (SD): 69.1 (15.2) | NR |
| Linde 2019 ([47](#_ENREF_47)) | Retrospective observational study | UK | Clinical Practice Research Datalink and linked Hospital Episode Statistics databases | Patients with CKD | sK+ >5.0 mmol/L | RAASi <50% ESC recommended dose | 27,935 | Male: 41.0 Female 59.0 | Mean (SD): 74.8 (10.8) | NR |
|  |  |  |  |  |  | RAASi ≥50% ESC recommended dose | 26,596 | Male: 44.4 Female 55.6 | Mean (SD): 71.4 (10.4) | NR |
| Iskandar 2012 ([62](#_ENREF_62)) | Retrospective observational study | Indonesia | Central Army Hospital Gatot Soebroto Jakarta | Patients with ESRD under haemodialysis | sK+ >5.3 mEq/L | ACEi (captopril/lisinopril) or ARB (valsartan) | 44 | Male: 70.5 Female 29.5 | Mean (SD): 51.5 (10.1); range: 41.0 to 74.0 | NR |
| Kashihara 2019 ([88](#_ENREF_88)) | Retrospective observational study | Japan | Medical Data Vision | Patients with stage 1-5 CKD | sK+ ≥5.1 mEq/L | RAASi (ACEi/ARB/MRA) | NR | NR | NR | NR |
| Saito 2017 ([101](#_ENREF_101)) | Retrospective observational study | Japan | Database of St Luke’s International Hospital | Patients with stage 3-5 non-dialysis dependent CKD | sK+ ≥5.5 mEq/L | RAASi (ACEi/ARBs/MRAs/DRI) | 986 | Male: 58.2 Female 41.8 | Mean (SD): 76.0 (11.3) | NR |
| ARTS-HF Pitt 2013 ([26](#_ENREF_26)) | RCT | Multinational | NA | Patients with mild (stage 2) CKD (eGFR 60 to 90 mL/min/1.73 m2) + HFpEF  (Part A) | NR | Finerenone (BAY 94-8862 – 2.5 mg qd) | 16 | Male: 81.3 Female 18.7 | Mean: 65.9; range: 49.0 to 78.0 | NR |
|  |  |  |  |  |  | MRA (finerenone- 5 mg qd) | 16 | Male: 81.3 Female 18.7 | Mean: 66.3; range: 42.0 to 79.0 | NR |
|  |  |  |  |  |  | MRA (finerenone – 10 mg qd) | 17 | Male: 70.6 Female 29.4 | Mean: 68.8; range: 55.0 to 85.0 | NR |
|  |  |  |  |  |  | Placebo | 16 | Male: 87.5 Female 12.5 | Mean: 63.9; range: 50.0 to 75.0 | NR |
|  |  |  |  | Patients with moderate (stage 3) CKD (eGFR 30 to 60 mL/min/1.73 m2) + HFpEF fraction  (Part B) |  | MRA (finerenone – 2.5 mg qd) | 66 | Male: 78.8 Female 21.2 | Mean: 71.2; range: 46.0 to 85.0 | NR |
|  |  |  |  |  |  | MRA (finerenone – 5 mg qd) | 67 | Male: 82.1 Female 17.9 | Mean: 72.0; range: 51.0 to 86.0 | NR |
|  |  |  |  |  |  | MRA (finerenone -10 mg qd) | 67 | Male: 88.1 Female 11.9 | Mean: 72.5; range: 52.0 to 89.0 | NR |
|  |  |  |  |  |  | MRA (finerenone – 5 mg bid) | 64 | Male: 71.9 Female 28.1 | Mean: 71.9; range: 44.0 to 88.0 | NR |
|  |  |  |  |  |  | MRA (spironolactone – 25 or 50 mg qd) | 63 | Male: 79.4 Female 20.6 | Mean: 72.8; range: 40.0 to 89.0 | NR |
|  |  |  |  |  |  | Placebo | 65 | Male: 76.9 Female 23.1 | Mean: 72.4; range: 51 to 85 | NR |
| AVOID Persson 2010 ([27](#_ENREF_27)) | RCT | Multinational | NA | Patients with stage 1 CKD + HT + DM | sK+ >5.5  sK+ >6.0 mmol/L | DRI (aliskiren) | 64 | Male: 73.4 Female 26.6 | Mean (SD): 53 (10.7) | NR |
|  |  |  |  |  |  | Placebo | 51 | Male: 68.6 Female 31.4 | Mean (SD): 55.3 (9.5) | NR |
|  |  |  |  | Patients with stage 2 CKD + HT + DM |  | DRI (aliskiren) | 104 | Male: 71.2 Female 28.8 | Mean (SD): 59.8 (8.3) | NR |
|  |  |  |  |  |  | Placebo | 122 | Male: 78.7 Female 21.3 | Mean (SD): 60.7 (9.2) | NR |
|  |  |  |  | Patients with stage 3 CKD + HT + DM |  | DRI (aliskiren) | 129 | Male: 64.3 Female 35.7 | Mean (SD): 63.1 (8.2) | NR |
|  |  |  |  |  |  | Placebo | 119 | Male: 70.6 Female 29.4 | Mean (SD): 65.4 (8.5) | NR |
| Gwoo 2014 ([81](#_ENREF_81)) | Retrospective observational study | South Korea | Medical records | Patients with stage 2-4 CKD + HT | sK+ ≥5.0 mEq/L | MRA (spironolactone) | 891 | Male: 43.5 Female 56.5 | Mean (SD): 72.3 (11.8) | NR |
| Vukusich 2010 ([19](#_ENREF_19)) | RCT | Chile | NA | Patients with ESRD under haemodialysis | NR | MRA (spironolactone) | 30 | Male: 66.7 Female 33.3 | Mean (SD): 60.1 (5.2) | NR |
|  |  |  |  |  |  | Placebo | 23 | Male: 61 Female 39 | Mean (SD): 55.6 (3.6) | NR |
| PHASE Walsh 2015 ([20](#_ENREF_20)) | RCT | Canada | NA | Patients with ESRD under haemodialysis | sK+ >6.0 mEq/L  sK+ >6.5 mEq/L  sK+ >7 mEq/L | MRA (eplerenone) | 77 | Male: 61 Female 39 | Mean (SD): 62.1 (14.6) | NR |
|  |  |  |  |  |  | Placebo | 77 | Male: 63.6 Female 36.4 | Mean (SD): 63.1 (13.7) | NR |
| An 2021 ([102](#_ENREF_102)) | Retrospective observational study | USA | Administrative and electronic health records from Kaiser Permanente Southern California | Patients with stage 2-4 diabetic kidney disease + HT | ICD-9 diagnostic code of 276.7  ICD-10 diagnostic code of E87.5  pK+/sK+ ≥5.5 mEq/L | Combination therapy (MRA + ACEI/ARB) | 1,282 | Male: 57.3 Female 42.7 | Mean (SD): 65.4 (11.2) | White: 6.4 Black: 19 Asian: 16.4 Hispanic: 23.9 Other: 4.4 |
|  |  |  |  |  |  | Monotherapy (ACEI/ARB only) | 5,484 | Male: 58.9 Female 41.1 | Mean (SD): 65 (12.9) | White: 38.5 Black: 14.1 Asian: 15 Hispanic: 25.1 Other: 7.2 |
| Qu 2021 ([71](#_ENREF_71)) | Retrospective observational study | China | Registry-based study conducted Hospital of Wenzhou Medical University | Patients with stage 3-5 CKD | sK+ >5.5 mmol/L | MRA (spironolactone) | 200 | Male: 64.5 Female 35.5 | Mean (SD): 73.9 (9.5) | NR |
|  |  |  |  |  |  | Standard therapy | 360 | Male: 72.2 Female 27.8 | Mean (SD): 72 (10.6) | NR |
| Edwards 2021 ([1](#_ENREF_1)) | RCT | UK | NA | Patients with stage 2-3 non-diabetic CKD | sK+ >5.0 mEq/L | MRA (spironolactone) | 77 | Male: 69.0 Female 31.0 | Mean (SD): 57.0 (14.0) | NR |
|  |  |  |  |  |  | Diuretic (chlorthalidone) | 77 | Male: 69.0 Female 31.0 | Mean (SD): 56.0 (15.0) | NR |
| TRANSITION Straburzynska-Migaj 2021 ([2](#_ENREF_2)) | RCT | Multinational | NA | Patients with stage 3 CKD + HFpEF | NR | ARNi (sacubitril/valsartan) | 476 | NR | NR | NR |
| Ruilope 2000 ([3](#_ENREF_3)) | RCT | Multinational | NA | Patients with progressive CKD with or without proteinuria + HT | sK+ ≥6.0 mmol/L | ARB (valsartan – 160 mg) | 22 | Male: 73.0 Female 27.0 | Mean (SD): 57.3 (14.8) | White: 96.0 Black: 4.0 |
|  |  |  |  |  |  | ARB (valsartan – 80 mg) + ACEi (benazepril – 5 or 10 mg) | 42 | Male: 70.0 Female 30.0 | Mean (SD): 56.9 (11.7) | White: 100 Black: 0 |
|  |  |  |  |  |  | ARB (valsartan – 160 mg) + ACEi (benazepril – 5 or 10 mg) | 44 | Male: 66.0 Female 34.0 | Mean (SD): 57.6 (12.2) | White: 98.0 Black: 2.0 |
| De Rosa 2001 ([77](#_ENREF_77)) | Single-arm trial | NR | NA | Patients with chronic renal insufficiency + HT | sK+ >6.0 mEq/L | ARB (irbesartan) | 52 | Male: 70.0 Female 30.0 | Mean (SD): 55.4 (14.8) | NR |
| MiREnDa Hammer 2019 ([4](#_ENREF_4)) | RCT | Germany | NA | Patients with ESRD under haemodialysis | sK+ 6.0 to 6.5 mmol/L  sK+ ≥6.5 mmol/L | MRA (spironolactone) | 50 | Male: 80.0 Female 20.0 | Mean (SD): 60.6 (13.1) | NR |
|  |  |  |  |  |  | Placebo | 47 | Male: 74.5 Female 25.5 | Mean (SD): 59.9 (13.4) | NR |
| Spin-D Charytan 2019 ([5](#_ENREF_5)) | RCT | USA | NA | Patients with ESRD under haemodialysis | sK+ >6.5 mEq/L | MRA (spironolactone – 12.5 mg per day) | 27 | Male: 55.6 Female 44.4 | Mean (SD): 55.1 (13.6) | White: 29.6 Black: 66.7 Asian: 0 Hispanic/Latino: 11.1 |
|  |  |  |  |  |  | MRA (spironolactone – 25 mg per day) | 26 | Male: 73.1 Female 26.9 | Mean (SD): 53.3 (13.5) | White: 26.9 Black: 69.2 Asian: 3.8 Hispanic/Latino: 7.7 |
|  |  |  |  |  |  | MRA (spironolactone – 50 mg per day) | 25 | Male: 76.0 Female 24.0 | Mean (SD): 55.5 (9.8) | White: 20.0 Black: 68.0 Asian: 8.0 Hispanic/Latino: 8.0 |
|  |  |  |  |  |  | Placebo | 51 | Male: 62.7 Female 37.3 | Mean (SD): 56.8 (11.5) | White: 13.7 Black: 76.5 Asian: 3.9 Hispanic/Latino: 7.8 |
| UK HARP-III Haynes 2018 ([6](#_ENREF_6)) | RCT | UK | NA | Patients with stage A1-A3 CKD with eGFR 20 to 60 mL/min/1.73 m2 | sK+ ≥5.5 mmol/L | ARNi (sacubitril/valsartan) | 207 | Male: 71.0 Female 29.0 | Mean (SD): 62 (14.1) | White: 90.0 Black: 1.0 Asian: 5.0 Other:3.0 |
|  |  |  |  |  |  | ARB (irbesartan) | 207 | Male: 72.0 Female 28.0 | Mean (SD): 63.6 (13.4) | White: 92.0 Black: 2.0 Asian: 3.0 Other:2.0 |
| Lee 2014 ([103](#_ENREF_103)) | Retrospective observational study | Korea | Medical record-based study conducted at Yonsei University Health System Clinical Trial Center | Patients with stage 3 or 4 CKD with hyperkalaemia | sK+ ≥5.5 mmol/L | Maintenance group (ACEi or ARB without discontinuation) | 150 | Male: 54.7 Female 45.3 | Mean (SD): 61.8 (14.8) | NR |
|  |  |  |  |  |  | Withdrawal group (ACEi or ARB discontinuation for >3 months after hyperkalaemia) | 108 | Male: 58.3 Female 41.7 | Mean (SD): 60.3 (13.2) | NR |
| Agrawal 2016 ([134](#_ENREF_134)) | Prospective observational study | India | Krishna Institute of Medical Sciences Hospital in Nephrology Unit | Patients with CKD | NR | ARB (telmisartan) | 55 | Male: 61.2 Female 38.1 | Mean (SD): 48.2 (14.0) | NR |
| Woo 2013 ([50](#_ENREF_50)) | Retrospective observational study | Singapore | NR | Patients with stage 1-3 non-diabetic CKD | sK+ >5 mmol/L  sK+ >6.0 mmol/L | DRI (aliskiren) + ARB (losartan) | 49 | Male: 67.3 Female 32.7 | Mean (SD): 58.0 (12.0) | NR |
|  |  |  |  |  |  | DRI (aliskiren) | 46 | Male: 52.2 Female 47.8 | Mean (SD): 57.0 (12.0) | NR |
|  |  |  |  |  |  | ARB (losartan – high dose) | 48 | Male: 52.1 Female 47.9 | Mean (SD): 60.0 (11.0) | NR |
| Yildirim 2012 ([135](#_ENREF_135)) | Retrospective observational study | Turkey | Hacettepe University Hospital Nephrology Unit | Patients with stage 3-5 CKD | NR | RAASi | 279 | Male: 59.5 Female 40.5 | Mean (SD): 56.7 (15.2) | NR |
| Pisoni 2012 ([48](#_ENREF_48)) | Retrospective observational study | USA | University of Alabama at Birmingham | Patients with stage 3 CKD + Resistant HT | sK+ >5.0 mEq/L | MRA (spironolactone/eplerenone) | 36 | Male: 64.0 Female 36.0 | Mean (SD): 63.0 (11.0) | Black: 53.0 |
| Hirai 2018 ([72](#_ENREF_72)) | Retrospective observational study | Japan | Tokyo Women’s Medical University Medical Center East | Patients with stage 3-5 CKD and unknown stage CKD | sK+ >5.5 mEq/L | ACEi/ARB | 2,987 | Male: 61.0 Female 39.0 | Mean (SD): 70.1 (12.9) | NR |
| Jun 2019 ([78](#_ENREF_78)) | Retrospective observational study | Australia | National general practice data from the NPS MedicineWise’s MedicineInsight program | Patients with stage 3-5 CKD | sK+ >6.0 mmol/L or coded or free to text recorded diagnosis of hyperkalaemia | RAASi | 20,184 | Male: 45.4 Female 54.5 | Mean (SD): 76.9 (9.6) | NR |
| Belmar Vega 2019 ([114](#_ENREF_114)) | Retrospective observational study | Spain | Hospital Universitario Marqués de Valdecilla Nephrology Department registry | Patients with stage 3-5 CKD | sK+ 5.5 to 5.9 mmol/L  sK+ 6.0 to 6.4 mmol/L  sK+ 6.5 to 6.9 mmol/L  sK+ ≥7.0 mmol/L | RAASi | NR | NR | NR | NR |
| TOPCAT Beldhuis 2019 ([7](#_ENREF_7)) | RCT | Multinational | NA | Patients with 2 CKD + HFpEF | sK+ >5.5 mmol/L | MRA (spironolactone) | 413 | Male: 51.2 Female 48.8 | Mean (SD): 68.9 (9.8) | White: 76.8 |
|  |  |  |  |  |  | Placebo | 410 |  |  |  |
|  |  |  |  | Patients with stage 3a CKD + HFpEF |  | MRA (spironolactone) | 264 | Male: 53.5 Female 46.5 | Mean (SD): 72.6 (8.9) | White: 80.7 |
|  |  |  |  |  |  | Placebo | 269 |  |  |  |
|  |  |  |  | Patients with stage 4 CKD + HFpEF |  | MRA (spironolactone) | 209 | Male: 43.6 Female 56.4 | Mean (SD): 75.4 (9.2) | White: 78.3 |
|  |  |  |  |  |  | Placebo | 202 |  |  |  |
| Heshka 2010 ([104](#_ENREF_104)) | Retrospective observational study | Canada | The Renal HT Clinic | Patients with stage 3 or 4 CKD + Difficult-to-control HT | sK+ ≥5.5 mmol/L | MRA (spironolactone) | 34 | NR | NR | NR |
| Johnson 2023 ([49](#_ENREF_49)) | Retrospective observational study | USA | Primary care practices affiliated with an integrated health care delivery system | Patients with CKD + Hyperkalaemia | sK+ >5.0 mEq/L | RAASi | 4,424 | NR |  | NR |
| ROTATE-3 Provenzano 2022 ([8](#_ENREF_8)) | RCT | Multinational | NA | Patients with stage 2-4 CKD | sK+ >5.0 mmol/L | MRA (eplerenone) | 46 | Male: 76.1 Female 23.9 | Mean (SD): 69.5 (7.6) | NR |
|  |  |  |  |  |  | SGLT2i (dapagliflozin) + MRA (eplerenone) |  |  |  |  |
|  |  |  |  |  |  | SGLT2i (dapagliflozin) |  |  |  |  |
| Leon 2022 ([105](#_ENREF_105)) | Retrospective observational study | Canada (Manitoba cohort) | The Manitoba Centre for Health Policy and ICES | Patients with stage 3-5 CKD survived 90-days after the hyperkalaemia episode | sK+ ≥5.5 mmol/L | RAASi | 7,200 | Male: 52.5 Female 47.5 | Mean (SD): 72.4 (13.4)  Median (range): 74 (64 to 83) | NR |
|  |  | Canada (Ontario cohort) |  |  |  |  | 71,290 | Male: 48.3 Female 51.7 | Mean (SD): 79.5 (7.5) Median (range): 79.0 (74.0 to 85.0) | NR |
| Salik 2022 ([51](#_ENREF_51)) | Retrospective observational study | USA | Electronic health records of 14-hospital healthcare system in Eastern Massachusetts | Patients with CKD | sK+ >5.0 mmol/L | ARB | 186 | Male: 72.6 Female 27.4 | Median (range): 71.0 (61.0 to 78.0) | Non-white: 20.6 Hispanic: 7.5 |
|  |  |  |  |  |  | ACEi | 166 | Male: 69.9 Female 30.1 | Median (range): 69.0 (59.0 to 80.0) | Non-white: 23.5 Hispanic: 10.8 |
| Tokunaga 2010 ([136](#_ENREF_136)) | Retrospective observational study | Japan | NR | Patients with stage 3-4 CKD + HT | NR | ARB (telmisartan) | 36 | Male: 72.2 Female 27.8 | Mean (SD): 63.2 (12.7) | NR |
|  |  |  |  |  | NR | Control | 36 | Male: 66.7 Female 33.3 | Mean (SD): 64.7 (11.3) | NR |
| Bhandari 2021 ([28](#_ENREF_28)) | RCT | UK | NA | Patients with stage 4 or 5 CKD | NR | RAASi discontinuation group | 206 | Male: 68.0  Female: 32 | Mean (SD): 62.7 (12.6)  Median (range): 63.3 (28.8 to 90.6) | White: 83.0  Asian: 6.8  Black: 7.8  Other: 2.5 |
|  |  |  |  |  |  | RAASi continuation group | 205 | Male: 68.8  Female: 31.2 | Mean (SD): 61.4 (13.6)  Median (range): 63.3 (28.3 to 85.8) | White: 87.8  Asian: 7.8  Black: 3.4  Other: 1.0 |
| Fu 2021 ([137](#_ENREF_137)) | Retrospective observational study | Sweden | Swedish Renal Registry, a nationwide registry of patients with CKD G3–5 attending routine nephrologist-specialist care in Sweden | Patients with advanced CKD (eGFR <30 mL/min per 1.73m2) | NR | RAASi | 10,254 | Male: 64.3  Female: 35.7 | Median (range): 72 (63.0 to 79.0) | NR |
| Ahmed 2010 ([138](#_ENREF_138)) | Retrospective observational study | UK | Sheffield Kidney Institute | Patients with stage 4 or 5 CKD preparing for RRT | NR | RAASi | 52 | Male: 59.6  Female: 40.4 | NR | NR |
| Parmar 2024 ([63](#_ENREF_63)) | Retrospective observational study | UK | CKD-HF clinic: Electronic health records | Patients with CKD + HF + Hyperkalaemia | sK+ >5.4 mmol/L | NA | 35 | Male: 65.7 Female: 34.3 | Mean (SD): 73.7 (14.3) Median: 78 | NR |
|  |  |  |  | Patients with CKD + HF + Normokalaemia | NR | NA | 265 | Male: 66 Female: 34 | Mean (SD): 74.4 (12.4) Median: 78 | NR |
| Sevamontree 2024 ([107](#_ENREF_107)) | Retrospective observational study | Thailand | Outpatient department of Medicine, Ramathibodi Hospital, Mahidol University, Bangkok | Patients with stage 3-5 CKD + Hyperkalaemia | sK+ ≥5.8 mmol/L | NA | 118 | NR | NR | NR |
|  |  |  |  | Patients with stage 3-5 CKD + Normokalaemia | sK+ <5.8 mmol/L | NA | 734 | NR | NR | NR |
| NEFRONA Valdivielso 2024 ([52](#_ENREF_52)) | Retrospective observational study | Spain | 81 Spanish hospitals and 9 primary care centers | Patients with 3-5 CKD + Hyperkalaemia | sK+ >5.0 mEq/L | NA | **1690** | NR | NR | NR |
|  |  |  |  | Patients with 3-5 CKD + Hypokalaemia | sK+ <3.6 mEq/L | NA | 57 | NR | NR | NR |
| Gulcicek 2023 ([82](#_ENREF_82)) | Retrospective observational study | Turkey | Electronic database of the tertiary hospital | Patients with stages 1-5 CKD + Hyperkalaemia | sK+ ≥5.0 mmol/L | RAASi | 137 | Male: 45.26 Female: 54.74 | Mean (SD): 66.2 (10.9) | NR |
|  |  |  |  | Patients with stages 1-5 CKD + Normokalaemia | sK+ <5.0 mmol/L |  | 334 | Male: 44.61 Female: 55.39 | Mean (SD): 66.2 (10.9) | NR |
| Zhou 2023a ([53](#_ENREF_53)) | Retrospective observational study | China | China National Heart Failure Registry | Patients with CKD + HF + Hyperkalaemia | sK+ >5.0 mmol/L | NA | 114 | NR | NR | NR |
|  |  |  |  | Patients with CKD + HF + Hypokalaemia | sK+ 0 to 3.5 mmol/L | NA | 88 | NR | NR | NR |
|  |  |  |  | Patients with CKD + HF + Normokalaemia | sK+ >3.5 to 5.0 mmol/L | NA | 564 | NR | NR | NR |
| Qadir 2023 ([54](#_ENREF_54)) | Retrospective observational study | Pakistan | Nephrology Unit, Khyber Teaching Hospital, Peshawar | Patients with stage 1-4 non-dialysis dependent CKD + Hyperkalaemia | sK+ >5.0 mEq/L | NA | 68 | Male: 48.7 Female: 41.4 | NR | NR |
|  |  |  |  | Patients with stage 1-4 non-dialysis dependent CKD + Normokalaemia | NR | NA | 82 | Male: 51.3 Female: 58.6 | NR | NR |
| EQUAL de Rooij 2023 ([79](#_ENREF_79)) | Prospective observational study | Multinational | Web-based clinical record | Patients with stage 4-5 CKD + Hyperkalaemia | sK+ >5.0 to ≤5.5 mmol/L | NA | 294 | Male: 69 Female: 30.6122448979592 | Mean (SD): 75 (7) | NR |
|  |  |  |  |  | sK+ >5.5 to ≤6.0 mmol/L | NA | 84 | Male: 69 Female: 30.952380952381 | Mean (SD): 76 (6) | NR |
|  |  |  |  |  | sK+ >6.0 mmol/L | NA | 31 | Male: 81 Female: 19.3548387096774 | Mean (SD): 77 (7) | NR |
|  |  |  |  | Patients with stage 4-5 CKD + Hypokalaemia | sK+ ≤3.5 mmol/L | NA | 44 | Male: 66 Female: 34.0909090909091 | Mean (SD): 76 (6) | NR |
|  |  |  |  | Patients with stage 4-5 CKD + Normokalaemia | sK+ >3.5 to ≤4.0 mmol/L | NA | 228 | Male: 65 Female: 34.6491228070175 | Mean (SD): 77 (7) | NR |
|  |  |  |  |  | sK+ >4.0 to ≤4.5 mmol/L | NA | 474 | Male: 60 Female: 39.873417721519 | Mean (SD): 77 (7) | NR |
|  |  |  |  |  | sK+ >4.5 to ≤5.0 mmol/L | NA | 559 | Male: 67 Female: 33.2737030411449 | Mean (SD): 76 (7) | NR |
| Perez-Navarro 2023 ([55](#_ENREF_55)) | Retrospective observational study | Mexico | Nephrology service of the Hospital General de México | Patients with stage 1-5 CKD + Hyperkalaemia | sK+ >5.0 mmol/L | NA | **345** | NR | NR | NR |
|  |  |  |  | Patients with stage 1-5 CKD + Normokalaemia | NR |  | **1,016** | NR | NR | NR |
| Wang 2023 ([89](#_ENREF_89)) | Retrospective observational study | China | Peking University First Hospital | Patients with stage 1-4 CKD + Hyperkalaemia | sK+ >5.0 mmol/L | NA | 196 | Male: 54.7 Female: 45.3 | Mean (SD): 56 (16) | NR |
|  |  |  |  | Patients with stage 1-4 CKD + Normokalaemia | NR |  | 331 |  |  |  |
| REVOLUTIONIZE III Bakris 2023 ([84](#_ENREF_84)) | Retrospective observational study | USA | Optum’s de-identified Market Clarity data | Patients with stage 3-4 CKD + hyperkalaemia | sK+ >5.0 mEq/L | NA | 4,549 | NR | NR | NR |
|  |  |  |  | Patients with stage 3-4 CKD + Normokalaemia | sK+ 3.5 to 5.0 mEq/L |  | 4,549 | NR | NR | NR |
| Gaol 2024 ([139](#_ENREF_139)) | Retrospective observational study | Indonesia | One private hospital in Jakarta | Patients with CKD undergoing hemodialysis + Hyperkalaemia | NR | NA | 161 | NR | NR | NR |
|  |  |  |  | Patients with CKD undergoing hemodialysis + Normokalaemia | NR | NA |  | NR | NR | NR |
|  |  |  |  | Patients with CKD undergoing hemodialysis + Hypokalaemia | NR | NA |  | NR | NR | NR |
| Rastogi 2023 ([140](#_ENREF_140)) | Retrospective observational study | USA | hospital records/claims from the USA (Optum’s de-identified Market Clarity Data) and Japan (Medical Data Vision) | Patients with stage 3-4 CKD | NR | RAASi | 7,506 | NR | NR | NR |
|  |  | Japan |  |  |  |  | 1,179 | NR | NR | NR |
| Mårup 2023 ([73](#_ENREF_73)) | Prospective observational study | Denmark | NR | Patients with CKD on maximal tolerated RAASi (ACEi or ARB) with history of at least two hyperkalaemia episodes | pK+ >4.5mmol/L | MRA (spironolactone) | 58 | Male: 81 Female: 19 | Mean (SD): 65 (NR) | NR |
| Zhou 2023b ([29](#_ENREF_29)) | RCT | China | NA | Patients with stage 3b-5 CKD + HT | sK+ ≥5.5 mmol/L | ARNi (sacubitril/valsartan) | 44 | Male: 59.1 Female: 55 | Mean (SD): 60.4 (14.0) | NR |
|  |  |  |  |  |  | Conventional antihypertensive | 40 | Male: 40.9 Female: 45 | Mean (SD): 58.3 (15.3) | NR |
| Rajak 2023 ([141](#_ENREF_141)) | Retrospective observational study | USA | NR | Patients with CKD + DM | NR | ARB (finerenone) | NR | NR | NR | NR |
| Bornstein 2024 ([30](#_ENREF_30)) | RCT | Multinational | NA | Patients with albuminuric CKD + DM | sK+ >5.0 mEq/L | MRA (BI 690517 3 mg) | 18 | Male: 88.9 Female: 11.1 | Mean (SD): 66.6 (10.3) | NR |
|  |  |  |  |  |  | MRA (BI 690517 10 mg) | 13 | Male: 84.6 Female: 15.4 | Mean (SD): 61.2 (14.6) | NR |
|  |  |  |  |  |  | MRA (BI 690517 40 mg) | 14 | Male: 71.4 Female: 28.6 | Mean (SD): 63.4 (11) | NR |
|  |  |  |  |  |  | Placebo | 9 | Male: 77.8 Female: 22.2 | Mean (SD): 64.3 (9) | NR |
| Jimenez-Marrero 2024 ([56](#_ENREF_56)) | Retrospective observational study | Spain | Southern Metropolitan Healthcare area of Barcelona; Authors linked two population-based healthcare databases: the dataset maintained by the Catalan Institute of Health (Institut Catal`a de Salut, ICS) and the Health System database managed by the Catalan Health Department (Servei Catal`a de la Salut, CatSalut) | Patients with CKD | sK+ >5.0 mEq/L | RAASi | 3,868 | NR | NR | NR |
| PROERCAN Garcia-Prieto 2024 ([31](#_ENREF_31)) | RCT | Spain | Hospital General Universitario Gregorio Marañon | Patients with stages 3-4 CKD without proteinuria + HT | sK+ ≥5.5 mmol/L | ACEi | 40 | Male: 50 Female: 50 | Mean (SD): 77.5 (5.7) | NR |
|  |  |  |  |  |  | Non ACEi | 48 | Male: 37.5 Female: 62.5 | Mean (SD): 78.2 (6.4) | NR |
| An 2023 ([85](#_ENREF_85)) | Retrospective observational study | USA | Kaiser Permanente Southern California (KPSC) | Patients with CKD | sK+ ≥5.0 mEq/L | RAASi discontinuation | 776 | Male: 51.7 Female: 48.3 | Mean (SD): 76.4 (11.2) | Asian/Pacific islander: 8.8 Hispanic: 25.6 Non-Hispanic black: 13.1 Non-Hispanic white: 51.3 Other: 1.2 |
|  |  |  |  |  |  | RAASi continuation | 4952 | Male: 45.5 Female: 54.5 | Mean (SD): 75.8 (10.4) | Asian/Pacific islander: 10.3 Hispanic: 27.3 Non-Hispanic black: 10.9 Non-Hispanic white: 50.3 Other: 1.1 |
| OPTIMIZE I Agiro 2023 ([142](#_ENREF_142)) | Retrospective observational study | USA | Two large, closed medical and pharmacy insurance claims datasets from HealthVerity | Patients with stages 3-5 CKD initiating SZC | NR | RAASi optimisation (same dose or with an up-titration) | 314 | Male: 61.5 Female: 38.5 | Mean (SD): 62.1 (12.4) | NR |
|  |  |  |  | Patients with stages 3-5 CKD initiating SZC | NR | Without RAASi optimisation (discontinued or with a down-titration) | 84 | Male: 78.6 Female: 21.4 | Mean (SD): 63.1 (12.4) | NR |
| Gregg 2023 ([143](#_ENREF_143)) | Retrospective observational study | USA | USA veterans seen in the Veterans Affairs (VA) healthcare system | Patients with stage 3-4 CKD | NR | ACEi or ARB: current user | 882,441 | Male: 97.5 Female: 2.5 | NR | White: 70.9 Black or African American: 16.2 Other: 1.6 |
|  |  |  |  |  |  | ACEi or ARB: discontinued user | 326,794 | Male: 97.3 Female: 2.7 | NR | White: 69.7 Black or African American: 16.0 Other: 1.6 |
| Chinnadurai 2023 ([64](#_ENREF_64)) | Prospective observational study | UK | Renal services at Salford Royal Hospital, in the Northern Care Alliance NHS Foundation Trust, Salford | Patients with non-dialysis dependent CKD | sK+ >5.4 mEq/L | RAASi | 321 | Male: 63.6 Female: 36.4 | Median (Range): 69 (61-75) | NR |
| Nicholas 2023 ([144](#_ENREF_144)) | Prospective observational study | Multinational | NR | Patients with CKD + DM | NR | ARB (Finerenone) | 574 | NR | Mean (SD): 66 (11) | NR |
| McFarland 2023 ([57](#_ENREF_57)) | Retrospective observational study | USA | Midwestern 11-hospital health system: electronic medical record | Patients with stage 3-5 CKD + HF | sK+ >5.0 mEq/L | ARNi (Sacubitril/Valsartan) | 50 | Male: 60 Female: 40 | Median (IQR): 70.3 (62.8-77.1) | White: 84 Black: 12 Hispanic: 4 |
| Ding 2023 ([58](#_ENREF_58)) | Retrospective observational study | China | Department of Nephrology, Peking University International Hospital | Patients with ESRD undergoing maintenance dialysis + HT | sK+ >5.0 mmol/L | ARNi (Sacubitril/Valsartan) | 51 | Male: 68.6 Female: 31.4 | Median (Range): 59 (51-69) | NR |
|  |  |  |  |  |  | Not taking sacubitril/ valsartan | 51 | Male: 68.6 Female: 31.4 | Median (Range): 59 (51-68) | NR |
| Jariwala 2023 ([145](#_ENREF_145)) | Retrospective observational study | India | Department of Cardiology, Yashoda Hospitals, Somajiguda, Hyderabad | Patients with CKD + DM | NR | ARB (Finerenone) | 42 | NR | NR | NR |
|  |  |  |  |  |  | No finerenone | 44 | NR | NR | NR |
| Svensson 2023 ([59](#_ENREF_59)) | Retrospective observational study | Sweden | National registries linked with health records from two large Swedish regions (Stockholm and Skåne) | Patients with CKD | sK+ >5.0 mmol/L | Maintained RAASi | NR | NR | NR | NR |
|  |  |  |  |  |  | Reduced RAASi | NR | NR | NR | NR |
| Tuttle 2024 ([32](#_ENREF_32)) | RCT | Multinational | NA | Patients with CKD | NR | Pooled* BI 690517 3 mg | 147 | Male: 68 Female: 32 | Mean (SD): 64.9 (11.5) | White: 62 Black or African American: 8 Asian: 27 American Indian or Alaska Native: 1 Native Hawaiian or Other Pacific Islander: 0  Multiple: 1 |
|  |  |  |  |  |  | Pooled* BI 690517 10 mg | 146 | Male: 64 Female: 36 | Mean (SD): 64.6 (11.1) | White: 55 Black or African American: 10 Asian: 29 American Indian or Alaska Native: 3 Native Hawaiian or Other Pacific Islander: 0  Multiple: 3 |
|  |  |  |  |  |  | Pooled* BI 690517 20 mg | 146 | Male: 68 Female: 32 | Mean (SD): 63·0 (11.5) | White: 58 Black or African American: 11 Asian: 27 American Indian or Alaska Native: 3 Native Hawaiian or Other Pacific Islander: 0 Multiple: 1 |
|  |  |  |  |  |  | Pooled* BI 690517 placebo | 147 | Male: 67 Female: 33 | Mean (SD): 62.8 (10.9) | White: 59 Black or African American: 14 Asian: 24 American Indian or Alaska Native: 2 Native Hawaiian or Other Pacific Islander: 1  Multiple: 1 |
| Guney 2009 ([33](#_ENREF_33)) | RCT | Turkey | NR | Patients with stage 1-3 non-diabetic CKD | sK+ >5.5 mEq/L | ACEIs and/or ARBs + spironolactone | 15 | Male: NR Female: NR | NR | NR |
|  |  |  |  |  |  | ACEIs and/or ARBs | 15 | Male: NR Female: NR | NR | NR |
| Edwards 2009 ([34](#_ENREF_34)) | RCT | UK | Renal clinics at a University teaching hospital | Patients with stage 2-3 non-diabetic CKD | sK+ >6.5 or >6.0 mEq/L | Spironolactone | 56 | Male: 59 Female: 41.1 | Mean (SD): 53 (12) | NR |
|  |  |  |  |  |  | Placebo | 56 | Male: 57 Female: 42.9 | Mean (SD): 54 (12) | NR |

*Pooled groups include participants who received BI 690517 either as monotherapy or in combination with empagliflozin.

**Abbreviations:** β: beta; ACEi: angiotensin-converting enzyme inhibitors; ARB: angiotensin-receptor blockers; ARNi: angiotensin receptor/neprilysin inhibitors; bid: twice a day; CCB: calcium channel blocker; CKD: chronic kidney disease; CrCl: creatinine clearance; CPRD: Clinical Practice Research Datalink; DARE: Data Analytics Research and Evaluation; DM: diabetes mellitus; DOPPS: Dialysis Outcomes and Practice Patterns Study; DRI: direct renin inhibitors; eGFR: estimated glomerular filtration rate; EMR: electronic medical record; ESC: European Society of Cardiology; ESRD: end-stage renal disease; HF: heart failure; HFpEF: heart failure with preserved ejection fraction; HFrEF: heart failure with reduced ejection fraction; HT: hypertension; ICD-10: International Classification of Diseases, Tenth Revision; ICD-9: International Classification of Diseases, Ninth Revision; ICS: Institut Catal`a de Salut; IQR: interquartile range; JMDV: Japan Medical Data Vision; K+: potassium; KPSC: Kaiser Permanente Southern California ; m2: square metre; mEq/L: milliequivalents per litre; mg: milligram; mg/dL: milligrams per decilitre; MI: myocardial infarction; mL/min: millilitre per minute; mmol/L: millimoles per litre; MRA: mineralocorticoid-receptor antagonists; NA: not applicable; NDC: National Drug Code; NR: not reported; NHS: National Health Service; pK+: plasma potassium; qd: once daily; RAASi: renin-angiotensin-aldosterone system inhibitors; RCT: randomised control trial; RRT: renal replacement therapy; SD: standard deviation; SZC: sodium zirconium cyclosilicate; SGLT2i: sodium/glucose cotransporter-2 inhibitors; sK+: serum potassium; UK: United Kingdom; USA: United States of America; vs: versus; VA, veterans Affairs .

Table S14. Baseline disease characteristics across included studies

| **Study** | **Population description** | **Hyper-, hypo-, or normokalaemia definition** | **Treatment** | **Sample size** | **Baseline treatment (%)** | **Baseline Comorbidities (%)** | **CKD stage (%)** | **eGFR, mL/min/1.73 m2 Mean (SD) or Median (range)** | **sCr (mg/dL) Mean (SD) or Median (range)** | **sAlb (g/dL) Mean (SD) or Median (range)** | **sK (mmol/L) Mean (SD) or Median (range)** |
| --- | --- | --- | --- | --- | --- | --- | --- | --- | --- | --- | --- |
| Li 2023a ([65](#_ENREF_65)) | Patients with stage 4-5 CKD + DM + Hyperkalaemia | sK+ >5.5 mmol/L | NA | 120 | Any RAASi: 55.8 β-blockers: 29.8 K+ sparing diuretics: 18.3 Thiazide-loop diuretics: 48.9 Insulin: 87.3 β-2 agonist: 5.9 | HF: 33.1 DM: 100 CAD: 43.1 Stroke: 18.4 PAD: 15.1 | Stage 4: 51.7 Stage 5: 48.3 | Median (IQR): 13.4 (12.5 to 18.5) | NR | Mean (SD): 3.1 (0.4) | NR |
|  | Patients with stage 4-5 CKD + DM + Normokalaemia | sK+ ≤5.5 mmol/L | NA | 150 | Any RAASi: 36.2 β-blockers: 32.1 K+ sparing diuretics: 15.6 Thiazide-loop diuretics: 45.6 Insulin: 85.6 β-2 agonist: 6.3 | HF: 28.4 DM: 100 CAD: 38.5 Stroke: 15.8 PAD: 12.4 | Stage 4: 55.8 Stage 5: 44.2 | Median (IQR): 20.4 (18.3 to 25.3) | NR | Mean (SD): 3.2 (0.5) | NR |
| Wang 2023 ([89](#_ENREF_89)) | Patients with stage 1-4 CKD + Instantaneous hyperkalaemia | sK+ ≥5.0 mmol/L only once or lasting <3 months | NA | 1,078 | NR | NR | Stage 1: 3.6 Stage 2: 12 Stage 3a: 21.3 Stage 3b: 34.2 Stage 4: 29 | Median (IQR): 13.4 (12.5 to 18.5) | NR | NR | NR |
|  | Patients with stage 1-4 CKD + Persistent hyperkalaemia | sK+ ≥5.0 mmol/L lasting for ≥3 months | NA |  |  |  |  |  |  |  |  |
|  | Patients with stage 1-4 CKD + Normokalaemia | NR | NA |  |  |  |  |  |  |  |  |
| Calabrese 2022 ([60](#_ENREF_60)) | Patients with stage 1-5 or A1-A3 CKD or with other anatomic or urine sediment abnormalities according to the KDIGO 2012 Clinical Practice Guideline for the Evaluation and Management of CKD + Hyperkalaemia | sK+ >5.1 mEq/L | NA | 90 | ACEi: 17.8 ARBs: 53.3 Any RAASi: 68.9 K+ sparing diuretics: 7.8 Thiazide-loop diuretics: 51.1 K+ binders: 17.8 | HT: 35.9 HF: 4.4 DM: 38.4 Tumours: 10.0 | Stage 1- 5 or A1-A3 CKD: 100 | Median (IQR): 19.8 (10.5 to 32.5) | Median (IQR): 2.6 (1.7 to 4.6) | NR | NR |
|  | Patients with stage 1-5 or A1-A3 CKD or with other anatomic or urine sediment abnormalities according to the KDIGO 2012 Clinical Practice Guideline for the Evaluation and Management of CKD + Normokalaemia | sK+ ≤5.1 mEq/L | NA | 180 | ACEi: 20.1 ARBs: 40.2 Any RAASi: 58.7 K+ sparing diuretics: 10.6 Thiazide-loop diuretics: 43.0 K+ binders: 20.1 | HT: 77.7 HF: 1.7 DM: 40 Tumours: 8.4 | Stage 1- 5 or A1-A3 CKD: 100 | Median (IQR): 34.9 (16.2 to 58.0) | Median (IQR): 1.7 (1.1 to 3.2) | NR | NR |
| Zhang 2022a ([36](#_ENREF_36)) | Patients with CKD having ≥1 inpatient or outpatient visit + Hyperkalaemia | sK+ >5.0 mmol/L | NA | 1,015 | ACEi: 23.3 ARBs: 48.1 MRAs: 30.9 β-blockers: 42.8 Thiazide-loop diuretics: 54.2 CCB: 63.9 Dialysis: 2.9 K+ binders: 23.3 NSAID: 53.6 Anti-platelet: 47.3 Anti-dyslipidaemia: 38.9 Insulin: 37.6 OAD: 28.6 Bronchodilators: 27.7 Anti-HT: 23.8 | HT: 79.5 HF: 20.7 DM: 43.8 Arrhythmias: 28.1 Angina: 20.9 MI: 9.8 Other CVD: 39.3 Stroke: 31.0 Transient ischemic attack: 3.4 Other CBD: 12.3 PVD: 15.5 Peptic ulcer: 10.2 COPD: 6.0 Rheumatic disease: 4.7 Dyslipidaemia: 46.1 | NR | NR | NR | NR | NR |
|  | Patients with CKD having ≥1 inpatient or outpatient visit + Normokalaemia | NR | NA | 5,981 | ACEi: 16.8 ARBs: 46.5 MRAs: 7.1 β-blockers: 29.9 Thiazide-loop diuretics: 23.1 CCB: 59.8 Dialysis: 0.1 K+ binders: 16.8 NSAID: 56.8 Anti-platelet: 47.8 Anti-dyslipidaemia: 42.4 Insulin: 25.3 OAD: 43.6 Bronchodilators: 13.9 Anti-HT: 12.5 | HT: 78.5 HF: 4.8 DM: 54.1 Arrhythmias: 19.8 Angina: 16.5 MI: 3.7 Other CVD: 16.1 Stroke: 24.3 Transient Ischemic Attack: 2.6 Other CBD: 10.5 PVD: 15.2 Peptic ulcer: 8.5 COPD: 2.4 Rheumatic disease: 2.6 Dyslipidaemia: 57.3 | NR | NR | NR | NR | NR |
| Sharma 2021 ([120](#_ENREF_120)) | Patients with stage 1-5 CKD and unknown stage CKD + Hyperkalaemia | Two or more K+ lab tests >5.0 mEq/L on different dates; OR Two or more diagnosis codes of hyperkalaemia (ICD-9 code of 276.7 or one ICD-10 code of E87.5); OR One diagnosis code of hyperkalaemia and one lab K+ value >5.0 mEq/L; OR Evidence of a National Drug Code number for either patiromer or SPS | NA | 79,084 | NR | NR | Stage 1: 10.9  Stage 2: 34.7  Stage 3: 36.5  Stage 4: 10.3  Stage 5: 6.7  Unknown: 0.9 | NR | NR | NR | NR |
|  | Patients with stage 1-5 CKD and unknown stage CKD + Normokalaemia | sK+ 3.8 to 5.0 mEq/L | NA |  |  |  |  |  |  |  |  |
| Kohsaka 2021 ([86](#_ENREF_86)) | Patients with stage 1-5 CKD + Hyperkalaemia | sK+ ≥5.1 mmol/L | NA | 16,133 | ACEi: 10.2 ARBs: 39.6 MRAs: 14.9 Any RAASi: 74.7 β-blockers: 54.7 Thiazide-loop diuretics: 3 K+ binders: 9 Digoxin: 11.1 Inotrope: 23.0 Antidiabetics: 36.1 Loop diuretics: 22.9 Glucose injection + insulin: 2.1  Calcium gluconate: 1.9 Sodium bicarbonate: 8.3 | HT: 75.6 DM: 47.8 MI: 4.1 PVD: 16.8 CBD: 24.2 Chronic pulmonary disease: 15.9 AFib or flutter: 14.9 Valvular heart disease: 11.0 AKI: 4.2 Sepsis: 10.0 Peripheral oedema: 3.0 | Stage 1: 1.2 Stage 2: 7.1 Stage 3a: 16.5 Stage 3b: 25.6 Stage 4: 29.4 Stage 5: 20.2 | Mean (SD): 32.3 (20.4) | NR | NR | NR |
|  | Patients with stage 1-5 CKD + Normokalaemia | Without any record of sK+ levels of ≤3.5 mmol/L and ≥5.1 mmol/L | NA | 11,898 | ACEi: 5.4 ARBs: 33.7 MRAs: 3.2 Any RAASi: 74.4 β-blockers: 56.2 Thiazide-loop diuretics: 1.9 K+ binders: 0.2 Digoxin: 10.2 Inotrope: 13.1 Antidiabetics: 29.9 Loop diuretics: 4.4 Glucose injection + insulin: 0.0  Calcium gluconate: 0.3 Sodium bicarbonate: 2.6 | HT: 64.5 DM: 46.4 MI: 2.4 PVD: 12.3 CBD: 18.0 Chronic pulmonary disease: 12.1 AFib or flutter: 8.4 Valvular heart disease: 5.9 AKI: 0.4 Sepsis: 7.3 Peripheral oedema: 1.1 | Stage 1: 9.1 Stage 2: 32.1 Stage 3a: 39.1 Stage 3b: 15.3 Stage 4: 3.2 Stage 5: 1.2 | Mean (SD): 59.8 (21.3) | NR | NR | NR |
| Grandy 2021 ([37](#_ENREF_37)) | Patients with stage 3-4 non-dialysis dependent CKD + Hyperkalaemia | sK+ >5.0 mmol/L | NA | 216 | NR | HT: 78.2 DM: 50 Dyslipidaemia: 35.6 CAD: 18.5 PVD: 13.9 Depression: 13.4 Anxiety: 13.4 Benign prostatic hyperplasia: 12.0 Retinopathy: 12.0 Atherosclerosis: 11.1 Arthritis: 10.6 Chronic pain: 11.6 | Stage 3a, 3b and 4: 100 | NR | NR | NR | Mean (SD): 5.49 (0.65) Median (range): 5.3 (5.1 to 12.0) |
|  | Patients with stage 3-4 non-dialysis dependent CKD + Normokalaemia | sK+ 3.5 to 5.0 mmol/L | NA | 933 | NR | HT: 75.6 DM: 39.9 Dyslipidaemia: 32.3 CAD: 18.1 PVD: 9.8 Depression: 10.2 Anxiety:8.9 Benign prostatic hyperplasia: 9.5 Retinopathy: 8.6 Atherosclerosis: 12.0 Arthritis: 5.9 Chronic pain: 6.3 | Stage 3a, 3b and 4: 100 | NR | NR | NR | Mean (SD): 4.4 (0.4)  Median (range): 4.5 (0.0 to 53.0) |
| Sharma 2020 ([38](#_ENREF_38)) | Patients with stage 1-5 CKD + Hyperkalaemia | 1. ≥2 sK+ >5.0 mmol/L on different dates (Logical Observation Identifiers Names and Codes) 2. ≥2 claims with principal or secondary diagnosis of hyperkalaemia (ICD-10) 3. 1 claim with hyperkalaemia and 1 K+ >5.0 mmol/L 4. ≥1 dispensed prescription for SPS or patiromer | NA | 6,235 | ACEi: 40.6 ARBs: 24.6 MRAs: 4.5 K+ binders: 40.6 Other RAASi: 0.3 | HT: 84.5 HF: 21.3 DM: 50.4 Hyperlipidaemia: 86.0 Obesity: 27.4 | Stage 1: 6 Stage 2: 37 Stage 4: 10 Stage 5: 1.9 | NR | NR | NR | Mean (SD): 4.7 |
|  | Patients with stage 1-5 CKD + Normokalaemia | NR | NA | 429,277 | ACEi: 24.6 ARBs: 19.1 MRAs: 2.3 K+ binders: 24.6 Other RAASi: 0.1 | HT: 63.6 HF: 9 DM: 25.5 Hyperlipidaemia: 63.1 Obesity: 20.4 | Stage 1: 24.2 Stage 2: 48.5 Stage 4: 2.2 Stage 5: 0.5 | NR | NR | NR | Mean (SD): 4.3 |
| Kanda 2020 ([87](#_ENREF_87)) | Patients with stage 1-5 CKD + Hyperkalaemia | sK+ ≥5.1 mmol/L | NA | 16,133 | ACEi: 10 ARBs: 40 MRAs: 15 Thiazide diuretics: 3 Loop diuretics: 23 K+ binders: 10 | HT: 78 HF: 40 DM: 48 Dyslipidaemia: 31 | Stage 1: 1 Stage 2: 7 Stage 3a: 16 Stage 3b: 26 Stage 4: 29 Stage 5: 20 | NR | NR | NR | Mean (SD): 5.4 (0.5) |
|  | Patients with stage 1-5 CKD + Normokalaemia | sK+ 3.5 mmol/L to 5.1 mmol/L | NA | 15,287 | ACEi: 5 ARBs: 34 MRAs: 4 Any RAASi: 40 Thiazide diuretics: 2 Loop diuretics: 5 K+ binders: 5 | HT: 65 HF: 21 DM: 45 Dyslipidaemia: 35 | Stage 1: 8 Stage 2: 30 Stage 3a: 43 Stage 3b: 15 Stage 4: 3 Stage 5: 2 | NR | NR | NR | Mean (SD): 4.3 (0.3) |
| Jimenez-Marrero 2020 ([39](#_ENREF_39)) | Patients with CKD + HF + Hyperkalaemia | sK+ >5.0 mEq/L | NA | 2,917 | NR | HF: 100 | NR | NR | NR | NR | NR |
|  | Patients with CKD + HF + Hypokalaemia | sK+ <3.5 mEq/L. | NA | 3,146 | NR | HF: 100 | NR | NR | NR | NR | NR |
| Betts 2020 ([121](#_ENREF_121)) | CKD patients with hyperkalaemia-related hospitalisations | sK+ >5.0 mEq/L | NA | 1,809 | NR | NR | NR | NR | NR | NR | NR |
|  | CKD patients with a hospitalisation without evidence of hyperkalaemia (normokalaemia) | sK+ <5.0 mEq/L | NA | 1,809 | NR | NR | NR | NR | NR | NR | NR |
| Mu 2020 ([122](#_ENREF_122)) | Patients with CKD + Hyperkalaemia | NR | NA | 26,809 | NR | NR | NR | NR | NR | NR | NR |
|  | Patients CKD + Normokalaemia | NR | NA | 26,809 | NR | NR | NR | NR | NR | NR | NR |
| DISCOVER CKD  James 2021 ([40](#_ENREF_40))  Linked study: James 2022 ([123](#_ENREF_123)) | Patients with stage 3-5 CKD including renal replacement therapy or two eGFR measures <60 mL/min/1.73 m2 between 90-730 days apart + Hyperkalaemia | sK+ >5.0 mmol/L | NA | 125,196 | Any RAASi: 49.3 K+ binders: 5.1 Diuretics: 39.8 | HT: 74.7 HF: 27.7 DM: 44.4 AKI: 26.9 Albuminuria: 8 Diabetic Nephropathy: 10.7 | Stage 3-5: 100 | Median (IQR): 42.2 (25.0 to 58.8) | NR | NR | Mean (SD): 5.3 (0.5) |
|  | Patients with stage 3-5 CKD including renal replacement therapy or two eGFR measures <60 mL/min/1.73 m2 between 90-730 days apart + Normokalaemia | sK+ <5.0 mmol/L | NA | 1,672,595 | Any RAASi: 27 K+ binders: 0.1 Diuretics: 19.6 | HT: 54.3 HF: 9.5 DM: 20.6 AKI: 4.7 Albuminuria: 1.5 Diabetic Nephropathy: 2.6 | Stage 3-5: 100 | Median (IQR): 65.7 (57.6 to 71.2) | NR | NR | Mean (SD): 4.2 (0.5) |
| Thomsen 2018 ([124](#_ENREF_124)) | Patients with stage 1-5 CKD or on dialysis + Hyperkalaemia | sK+ >5.0 mmol/L | NA | 43,397 | ACEi: 36.3 ARBs: 18 K+ sparing diuretics: 18.8 Dialysis: 1.2 K+ binders: 36.3 K+ supplements: 30.9 | HT: 70.6 HF: 19.8 DM: 25.4 MI: 15.2 PVD: 12.4 CBD: 18.3 Chronic pulmonary disease: 17.8 Connective tissue disease: 5.7 Ulcer disease: 10.2 Any liver disease: 2.9 Any malignant disease: 22.1 AFib or flutter: 20 Valvular heart disease: 8.9 Alcoholism-related disorders: 9.4 Medical obesity: 6.8 | Stage 1 and 2: 0.9 Stage 3a: 37.6 Stage 3b: 33.3 Stage 4: 20.5 Stage 5: 6 | NR | NR | NR | NR |
|  | Patients with stage 1-5 CKD or on dialysis + Normokalaemia | NR | NA | 43,397 | ACEi: 25 ARBs: 16.9 K+ sparing diuretics: 7.4 Dialysis: 0.3 K+ binders: 25 K+ supplements: 19.5 | HT: 61.8 HF: 8.5 DM: 14.6 MI: 10.3 PVD: 7.3 CBD: 14.7 Chronic pulmonary disease: 10.4 Connective tissue disease: 4.6 Ulcer disease: 6.9 Any liver disease: 1.2 Any malignant disease: 15.9 AFib or flutter: 12.8 Valvular heart disease: 5.2 Alcoholism-related disorders: 5.1 Medical obesity: 4 | Stage 1 and 2: 0.9 Stage 3a: 63.8 Stage 3b: 25.4 Stage 4: 7.1 Stage 5: 1.5 | NR | NR | NR | NR |
| Furuland 2018 ([108](#_ENREF_108)) | Pre-dialysis patients with stage 3-5 CKD + Hyperkalaemia | sK+ 5.0 to <5.5 mmol/L | NA | 18,454 | ACEi: 43.2 ARBs: 15.9 MRAs: 4.0 DRIs: 0.1 Any RAASi: 57.3 β-blockers: 28.6 CCB: Dihydropyridine: 23.6 Non Dihydropyridine: 4.0 K+ binders: 43.2 NSAIDs: 16.2 Statins: 49.9 Bronchodilators: 13.2 Diuretics: 33.5 | DM: 20.2 MI: 4.4 Stroke: 6.5 Arrhythmia: 8.0 PVD: 2.8 Chronic pulmonary disease: 10.4 Metastatic tumour: 2.3 Rheumatic disease: 2.9 Peptic ulcer: 1.0 Dementia: 2.0 Malignancy: 9.8 | Stage 3-5: 100 | Mean (SD): 49.6 (9.5) | NR | NR | Mean (SD): 5.2 (0.1) |
|  | Pre-dialysis patients with stage 3-5 CKD + Hyperkalaemia | sK+ 5.5 to <6.0 mmol/L | NA | 4,250 | ACEi: 45.3 ARBs: 16.1 MRAs: 6.6 DRIs: 0 Any RAASi: 60.4 β-blockers: 30.1 CCB: Dihydropyridine: 26.3 Non Dihydropyridine: 4.3 K+ binders: 45.3 NSAIDs: 16.4 Statins: 49.5 Bronchodilators: 13.7 Diuretics: 34.8 | DM: 24.1 MI: 4.5 Stroke: 6.4 Arrhythmia: 7.7 PVD: 3.5 Chronic pulmonary disease: 9.7 Metastatic tumour: 2.5 Rheumatic disease: 2.8 Peptic ulcer: 0.8 Dementia: 1.9 Malignancy: 9.5 | NR | Mean (SD): 47 (11.5) | NR | NR | Mean (SD): 5.6 (0.1) |
|  | Patients with CKD (stage 3a-5, pre-dialysis) + Hyperkalaemia | sK+ ≥6.0 mmol/L | NA | 1,026 | ACEi: 45.5 ARBs: 14.7 MRAs: 10.0 DRIs: 0 Any RAASi: 59.8 β-blockers: 31.8 CCB: Dihydropyridine: 28.3 Non Dihydropyridine: 3.0 K+ binders: 45.5 NSAIDs: 15.5 Statins: 46.9 Bronchodilators: 12.0 Diuretics: 39.7 | DM: 27.7 MI: 4.5 Stroke: 7.1 Arrhythmia: 8.1 PVD: 3.9 Chronic pulmonary disease: 9.3 Metastatic tumour: 3.0 Rheumatic disease: 2.9 Peptic ulcer: 0.7 Dementia: 3.4 Malignancy: 11.9 | NR | Mean (SD): 42.1 (14.5) | NR | NR | Mean (SD): 6.4 (0.5) |
|  | Patients with CKD (stage 3a-5, pre-dialysis) + Hypokalaemia | sK+ <3.5 mmol/L | NA | 3,635 | ACEi: 30.7 ARBs: 13.3 MRAs: 5.5 DRIs: 0 Any RAASi: 44.8 β-blockers: 27.3 CCB: Dihydropyridine: 33.7 Non Dihydropyridine: 4.1 K+ binders: 30.7 NSAIDs: 12.8 Statins: 40.1 Bronchodilators: 13.6 Diuretics: 78.2 | DM: 9.1 MI: 2.0 Stroke: 6.9 Arrhythmia: 8.6 PVD: 2.1 Chronic pulmonary disease: 10.5 Metastatic tumour: 3.3 Rheumatic disease: 4.3 Peptic ulcer: 0.8 Dementia: 4.9 Malignancy: 12.2 | NR | Mean (SD): 49.9 (8.8) | NR | NR | Mean (SD): 3.2 (0.2) |
|  | Patients with CKD (stage 3a-5, pre-dialysis) + Normokalaemia | sK+ 3.5 to <4.0 mmol/L | NA | 17,662 | ACEi: 33.5 ARBs: 14.0 MRAs: 2.5 DRIs: 0.1 Any RAASi: 46.6 β-blockers: 26.1 CCB: Dihydropyridine: 28.9 Non Dihydropyridine: 4.5 K+ binders: 33.5 NSAIDs: 13.9 Statins: 42.4 Bronchodilators: 11.7 Diuretics: 61.4 | DM: 10.1 MI: 2.4 Stroke: 6.3 Arrhythmia: 8.0 PVD: 2.1 Chronic pulmonary disease: 9.8 Metastatic tumour: 2.6 Rheumatic disease: 4.4 Peptic ulcer: 0.7 Dementia: 3.2 Malignancy: 9.6 | NR | Mean (SD): 51.6 (7.6) | NR | NR | Mean (SD): 3.8 (0.1) |
|  | Patients with CKD (stage 3a-5, pre-dialysis) + Normokalaemia | sK+ 4.0 to <4.5 mmol/L | NA | 50,065 | ACEi: 35.2 ARBs: 13.9 MRAs: 2.2 DRIs: 0.03 Any RAASi: 47.8 β-blockers: 25.3 CCB: Dihydropyridine: 24.4 Non Dihydropyridine: 4.2 K+ binders: 35.2 NSAIDs: 14.5 Statins: 42.9 Bronchodilators: 11.7 Diuretics: 43.0 | DM: 11.3 MI: 2.9 Stroke: 5.9 Arrhythmia: 7.6 PVD: 2.1 Chronic pulmonary disease: 10.0 Metastatic tumour: 2.5 Rheumatic disease:3.8 Peptic ulcer: 0.8 Dementia: 2.5 Malignancy: 9.3 | NR | Mean (SD): 51.9 (7.5) | NR | NR | Mean (SD): 4.2 (0.1) |
|  | Patients with CKD (stage 3a-5, pre-dialysis) + Normokalaemia | sK+ 4.5 to <5.0 mmol/L | NA | 48,543 | ACEi: 38.9 ARBs: 14.1 MRAs: 2.7 DRIs: 0.04 Any RAASi: 51.7 β-blockers: 26.6 CCB: Dihydropyridine: 23.3 Non Dihydropyridine: 3.9 K+ binders: 38.9 NSAIDs: 15.3 Statins: 45.9 Bronchodilators: 12.1 Diuretics: 34.6 | DM: 15.3 MI: 3.4 Stroke: 5.9 Arrhythmia: 7.8 PVD: 2.4 Chronic pulmonary disease: 9.9 Metastatic tumour: 2.2 Rheumatic disease: 3.2 Peptic ulcer: 0.8 Dementia: 2.1 Malignancy: 9.3 | NR | Mean (SD): 51.1 (8.2) | NR | NR | Mean (SD): 4.7 (0.1) |
| Neuenschwander 2023 ([116](#_ENREF_116)) | Patients with CKD + Hyperkalaemia | ICD-10 diagnostic code of E87.5 in any claim position or Medicare Part D fill for oral K+ binder use within 14 days prior to long-term care stay or during long-term care stay | NA | 145,315 | NR | NR | NR | NR | NR | NR | NR |
|  | Patients with CKD + Normokalaemia | No evidence of hyperkalaemia within 14 days prior to long-term care stay or during long-term care stay | NA | 650,228 | NR | NR | NR | NR | NR | NR | NR |
| Betts 2018 ([125](#_ENREF_125)) | Patients with stage 3-5 CKD and unspecified stage + Hyperkalaemia | sK+ >5.0 mEq/L | NA | 9,620 | NR | NR | Stage 3: 60.2 Stage 4: 19.8 Stage 5: 12.4 Unspecified stage: 7.6 | NR | NR | NR | NR |
|  | Patients with stage 3-5 CKD and unspecified stage + Normokalaemia | sK+ <5.0 mEq/L | NA | 9,620 | NR | NR | Stage 3: 60.2 Stage 4: 19.8 Stage 5: 12.4 Unspecified stage: 7.6 | NR | NR | NR | NR |
| Luo 2016 ([109](#_ENREF_109)) | Patients with CKD with eGFR <60 mL/min/1.73 m2 + Hyperkalaemia | sK+ 5.0 to 5.4 mEq/L | NA | 8,270 | Any RAASi: 31.8 β-blockers: 15 Thiazide diuretics: 9.4 CCB: 3.5 Loop diuretics: 7.0 | HF: 8.3 DM: 29.2 CAD: 13.9  CBD: 6.1 | NR | NR | NR | NR | NR |
|  |  | sK+ 5.5 to 5.9 mEq/L | NA | 2,187 | Any RAASi: 34.5 β-blockers: 16.3 Thiazide diuretics: 9.5 CCB: 3.6 Loop diuretics: 8.3 | HF: 8.8 DM: 30.9 CAD: 15.0  CBD: 6.9 | NR | NR | NR | NR | NR |
|  |  | sK+ ≥6.0 mEq/L | NA | 589 | Any RAASi: 37.4 β-blockers: 15.3 Thiazide diuretics: 10.9 CCB: 4.2 Loop diuretics: 10.9 | HF: 11.5 DM: 34.5 CAD: 15.6 CBD: 7.8 | NR | NR | NR | NR | NR |
|  | Patients with CKD with eGFR <60 mL/min/1.73 m2 + Normokalaemia | sK+ 3.5 to 3.9 mEq/L | NA | 5,664 | Any RAASi: 24.8 β-blockers: 17.7 Thiazide diuretics: 26.7 CCB: 5.7 Loop diuretics: 8.6 | HF: 8.9 DM: 22.1 CAD: 12.3 CBD: 6.5 | NR | NR | NR | NR | NR |
|  |  | sK+ 4.0 to 4.4 mEq/L | NA | 18,712 | Any RAASi: 27.3 β-blockers: 15.7 Thiazide diuretics: 16 CCB: 4.7 Loop diuretics: 6.8 | HF: 7.4 DM: 22.5 CAD: 12.2  CBD: 6.6 | NR | NR | NR | NR | NR |
|  |  | sK+ 4.5 to 4.9 mEq/L | NA | 18,947 | Any RAASi: 30.2 β-blockers: 15.6 Thiazide diuretics: 10.9 CCB: 3.9 Loop diuretics: 7.0 | HF: 7.8 DM: 26.4 CAD: 13.4 CBD: 6.2 | NR | NR | NR | NR | NR |
|  | Patients with CKD with eGFR <60 mL/min/1.73 m2 + Hypokalaemia | sK+ <3.5 mEq/L | NA | 897 | Any RAASi: 22.5 β-blockers: 14.3 Thiazide diuretics: 32.6 CCB: 6.8 Loop diuretics: 13.5 | HF: 11.3 DM: 20.4 CAD: 10.9 CBD: 6.9 | NR | NR | NR | NR | NR |
| Nakhoul 2015 ([90](#_ENREF_90)) | Patients with stage 3-4 non-dialysis dependent CKD + Hyperkalaemia | sK+ 5 to 5.4 mmol/L | NA | 3,931 | Any RAASi: 71.2 β-blockers: 61 Diuretics: 63.1 Statins: 60.4 | HT: 86.9 HF: 9.6 DM: 29.5 COPD: 8.5 CBD: 10.3 PVD: 3.6 Malignancy: 24.2 CAD: 27.3 | Stage 3a: 52 Stage 3b: 33.6 Stage 4: 14.4 | Mean (SD): 43.8 (11.4) | NR | Mean (SD): 4.1 (0.5) | NR |
|  |  | sK+ ≥5.5 mmol/L | NA | 1,213 | Any RAASi: 73 β-blockers: 59.9 Diuretics: 67.4 Statins: 56.8 | HT: 83.8 HF: 11.2 DM: 34.2 COPD: 8.7 CBD: 10.6 PVD: 4.1 Malignancy: 24.8 CAD: 24.1 | Stage 3a: 38.8 Stage 3b: 36.8 Stage 4: 24.4 | Mean (SD): 39.9 (12.2) | NR | Mean (SD): 4 (0.5) | NR |
|  | Patients with stage 3-4 non-dialysis dependent CKD + Normokalaemia | sK+ 3.5 to 3.9 mmol/L | NA | 5,392 | Any RAASi: 55.1 β-blockers: 50.8 Diuretics: 75.1 Statins: 47 | HT: 81.9 HF: 7.3 DM: 15.9 COPD: 9.1 CBD: 7.6 PVD: 1.9 Malignancy: 24.2 CAD: 16.9 | Stage 3a: 69.6 Stage 3b: 24.4 Stage 4: 6 | Mean (SD): 48.3 (9.8) | NR | Mean (SD): 4 (0.5) | NR |
|  |  | sK+ 4.0 to 4.9 mmol/L | NA | 24,717 | Any RAASi: 62.2 β-blockers: 54.5 Diuretics: 63.8 Statins: 49.5 | HT: 84.8 HF: 7.9 DM: 21.7 COPD: 8.4 CBD: 9.2 PVD: 2.7 Malignancy: 24.4 CAD: 21.8 | Stage 3a: 66.8 Stage 3b: 25.9 Stage 4: 7.3 | Mean (SD): 47.6 (10) | NR | Mean (SD): 4.1 (0.4) | NR |
|  | Patients with stage 3-4 CKD + Hypokalaemia | sK+ <3.5 mEq/L | NA | 1,106 | Any RAASi: 50.6 β-blockers: 49.6 Diuretics: 75.9 Statins: 56.6 | HT: 76.9 HF: 9.6 DM: 16.3 COPD: 9.3 CBD: 6.1 PVD: 2.1 Malignancy: 24.7 CAD: 14.1 | Stage 3a: 63.3 Stage 3b: 26.4 Stage 4: 10.3 | Mean (SD): 46.2 (11.1) | NR | Mean (SD): 3.9 (0.6) | NR |
| Integrated CKD care program Kaohsiung for delaying Dialysis Wang 2013 ([41](#_ENREF_41)) | Patients with stage 1-4 CKD not on renal replacement therapy + Hyperkalaemia | sK+ >5.0 mEq/L | NA | 175 | ACEi: 30.3 ARBs: 44 β-blockers: 17.7 Thiazide-loop diuretics: 10.3 K+ binders: 30.3 Furosemide: 12.6 Oral hypoglycaemic agents: 38.3 Insulin: 10.3 Phosphate binders: 15.4 | HT: 60.6 DM: 49.1 CVD: 30.3 | Stage 1: 0.6 Stage 2: 9.2 Stage 3: 50.9 Stage 4: 35.9 | Mean (SD): 27.2 (12.3) | NR | Mean (SD): 3.8 (0.5) | Mean (SD): 5.3 (0.3) |
|  | Patients with stage 1-4 CKD not on renal replacement therapy + Normokalaemia | sK+ 3.0 to 5 to 4 mEq/L | NA | 683 | ACEi: 27.2 ARBs: 38.5 β-blockers: 24.7 Thiazide-loop diuretics: 10.5 K+ binders: 27.2 Furosemide: 13.2  Oral hypoglycaemic agents: 24.2 Insulin: 2.5 Phosphate binders: 13.5 | HT: 63.6 DM: 38.1 CVD: 23.6 | Stage 1: 8.9 Stage 2: 14.1 Stage 3: 46.7 Stage 4: 30.3 | Mean (SD): 47.1 (28.2) | NR | Mean (SD): 3.9 (0.6) | Mean (SD): 3.8 (0.1) |
|  | Patients with stage 1-4 CKD not on renal replacement therapy + Normokalaemia | sK+ 4.0–4.5 mEq/L | NA | 974 | ACEi: 30.6 ARBs: 39.8 β-blockers: 21.6 Thiazide-loop diuretics: 8.9 K+ binders: 30.6 Furosemide: 12.3  Oral hypoglycaemic agents: 27.8 Insulin: 6.3 Phosphate binders: 11.5 | HT: 60.2 DM: 41.5 CVD: 21.1 | Stage 1: 3.9 Stage 2: 9.2 Stage 3: 50.9 Stage 4: 35.9 | Mean (SD): 40.5 (21.2) | NR | Mean (SD): 3.9 (0.5) | Mean (SD): 4.3 (0.1) |
|  | Patients with stage 1-4 CKD not on renal replacement therapy + Hypokalaemia | sK+ 4.5 to 5.0 mEq/L | NA | 474 | ACEi: 30.8 ARBs: 46.8 β-blockers: 24.9 Thiazide-loop diuretics: 7.6 K+ binders: 30.8 Furosemide: 11.0 Oral hypoglycaemic agents: 38.0 Insulin: 10.5 Phosphate binders: 13.3 | HT: 59.3 DM: 47.5 CVD: 21.3 | Stage 1: 0.8 Stage 2: 3 Stage 3: 49.4 Stage 4: 46.8 | Mean (SD): 33.4 (14.5) | NR | Mean (SD): 3.9 (0.5) | Mean (SD): 4.7 (0.1) |
|  | Patients with stage 1-4 CKD not on renal replacement therapy + Hypokalaemia | sK+ <3.5 mEq/L | NA | 194 | ACEi: 22.2 ARBs: 27.8 β-blockers: 22.2 Thiazide-loop diuretics: 12.4 K+ binders: 22.2 Furosemide: 14.9 Oral hypoglycaemic agents: 21.6 Insulin: 3.6 Phosphate binders: 8.2 | HT: 67.5 DM: 36.1 CVD: 22.2 | Stage 1: 7.2 Stage 2: 18 Stage 3: 46.9 Stage 4: 27.8 | Mean (SD): 47.9 (28.2) | NR | Mean (SD): 3.8 (0.7) | Mean (SD): 3.3 (0.2) |
| Hayes 2012 ([61](#_ENREF_61)) | Patients with moderate and advanced non-dialysis-dependent CKD + Hyperkalaemia | sK+ >5.3 mEq/L | NA | 95 | Any RAASi: 64 β-blockers: 42 Diuretics: 72 K+ supplements: 11 NSAIDs: 24 | DM: 60 Atherosclerotic CVD: 66 | NR | Mean (SD): 28.8 (10.8) | NR | Mean (SD): 3.5 (0.5) | Mean (SD): 5.7 (0.3) |
|  | Patients with moderate and advanced non-dialysis-dependent CKD + Normokalaemia | sK+ 3.6 to 4.5 mEq/L | NA | 557 | Any RAASi: 59 β-blockers: 46 Diuretics: 65 K+ supplements: 19 NSAIDs: 26 | DM: 53 Atherosclerotic CVD: 55 | NR | Mean (SD): 40.2 (18) | NR | Mean (SD): 3.6 (0.5) | Mean (SD): 4.1 (0.2) |
|  | Patients with moderate and advanced non-dialysis-dependent CKD + Normokalaemia | sK+ 4.5 to 5.3 mEq/L | NA | 536 | Any RAASi: 57 β-blockers: 46 Diuretics: 63 K+ supplements: 11 NSAIDs: 26 | DM: 57 Atherosclerotic CVD:56 | NR | Mean (SD): 36 (16.2) | NR | Mean (SD): 3.6 (0.5) | Mean (SD): 4.8 (0.2) |
|  | Patients with moderate and advanced non-dialysis-dependent CKD + Hypokalaemia | sK+ <3.6 mEq/L | NA | 39 | Any RAASi: 62 β-blockers: 62 Diuretics: 85 K+ supplements: 51 NSAIDs: 29 | DM: 44 Atherosclerotic CVD: 64 | NR | Mean (SD): 42 (21) | NR | Mean (SD): 3.7 (0.4) | Mean (SD): 3.2 (0.3) |
| Jain 2012 ([42](#_ENREF_42)) | Patients with advanced (stage 3-5) CKD + Hyperkalaemia | sK+ >5.0 mEq/L | NA | 777 | NR | NR | Stage 3 to 5: 100 | NR | NR | NR | NR |
|  | Patients with advanced (stage 3-5) CKD+ Normokalaemia | sK+ ≤5.0 mEq/L | NA | 607 | NR | NR | Stage 3 to 5: 100 | NR | NR | NR | NR |
| NephroTest Wagner 2017 ([43](#_ENREF_43)) | Patients with stage 1-5 non-dialysis-dependent CKD + Hyperkalaemia | sK+ >5.0 mmol/L | NA | 172 | Any RAASi: 86.1 β-blockers: 38.4 K+ sparing diuretics: 2.9 Thiazide-loop diuretics: 50.6 K+ binders: 16.9 Bicarbonate: 7.6 K+ binding resins: 16.9 | DM: 37.2 CVD history: 18.6 Primary kidney disease, Diabetic: 18.6 Primary kidney disease, Glomerular: 18.6 Primary kidney disease, Vascular: 23.3 Primary kidney disease, Polycystic: 3.5 Primary kidney disease, Interstitial: 7.6 Primary kidney disease, Other or unknown: 28.5 | Stage 1-5: 100 | Median (IQR): 24.7 (16.4 to 34.3) | NR | NR | NR |
|  | Patients with stage 1-5 non-dialysis-dependent CKD + Normokalaemia | sK+ 4.0 to 5.0 mmol/L | NA | 1,340 | Any RAASi: 76.5 β-blockers: 39.3 K+ sparing diuretics: 4.4 Thiazide-loop diuretics: 45.6 K+ binders: 7.2 Bicarbonate: 4.1 K+ binding resins: 7.2 | DM: 30.9 CVD history: 19.9 Primary kidney disease, Diabetic: 11.1 Primary kidney disease, Glomerular: 14.3 Primary kidney disease, Vascular: 26.4 Primary kidney disease, Polycystic: 4.3 Primary kidney disease, Interstitial: 8.8 Primary kidney disease, Other or unknown: 35.0 | Stage 1-5: 100 | Median (IQR): 37.6 (26.7 to 51.7) | NR | NR | NR |
|  | Patients with stage 1-5 non-dialysis-dependent CKD + Hypokalaemia | sK+ <4.0 mmol/L | NA | 566 | Any RAASi: 65.7 β-blockers: 32.5 K+ sparing diuretics: 3 Thiazide-loop diuretics: 48.2 K+ binders: 2.3 Bicarbonate: 1.6 K+ binding resins: 2.3 | DM: 25.6 CVD history: 10.9 Primary kidney disease, Diabetic: 5.1 Primary kidney disease, Glomerular: 12.4 Primary kidney disease, Vascular: 26.5 Primary kidney disease, Polycystic: 9.0 Primary kidney disease, Interstitial: 9.9 Primary kidney disease, Other or unknown: 37.1 | Stage 1-5: 100 | Median (IQR): 37.6 (26.7 to 51.7) | NR | NR | NR |
| Collins 2017 ([115](#_ENREF_115)) | Patients with stage 3-5 CKD + Hyperkalaemia | sK+ 5.0 to <5.5 mEq/L | NA | 9,021 | NR | NR | Stage 3a: 41.6 Stage 3b: 33.7  Stage 4: 21.8  Stage 5: 3.0 | NR | NR | NR | NR |
|  |  | sK+ 5.5 to <6.0 mEq/L | NA | 1,703 | NR | NR | Stage 3a: 32.5 Stage 3b: 35.6 Stage 4: 27.4 Stage 5: 4.5 | NR | NR | NR | NR |
|  |  | sK+ 6.0 to <6.5 mEq/L | NA | 266 | NR | NR | Stage 3a: 27.8 Stage 3b: 33.4 Stage 4: 33.4 Stage 5: 5.3 | NR | NR | NR | NR |
|  |  | sK+ 6.5 to 8.0 mEq/L | NA | 224 | NR | NR | Stage 3a: 31.6 Stage 3b: 26.8 Stage 4: 33.0 Stage 5: 8.6 | NR | NR | NR | NR |
|  | Patients with stage 3-5 CKD + Normokalaemia | sK+ 4.0 to <4.5 mEq/L | NA | 36,208 | NR | NR | Stage 3a: 53.3 Stage 3b: 31.1 Stage 4: 14.4 Stage 5: 1.1 | NR | NR | NR | NR |
|  |  | sK+ 4.5 to <5.0 mEq/L | NA | 26,731 | NR | NR | Stage 3a: 49.2 Stage 3b: 31.5 Stage 4: 17.7 Stage 5: 1.5 | NR | NR | NR | NR |
|  | Patients with stage 3-5 CKD + Hypokalaemia | sK+ 2.5 to <3. 0 mEq/L | NA | 257 | NR | NR | Stage 3a: 39.2 Stage 3b: 34.0 Stage 4: 20.9 Stage 5: 5.9 | NR | NR | NR | NR |
|  |  | sK+ 3.0 to <3.5 mEq/L | NA | 3,397 | NR | NR | Stage 3a: 50 Stage 3b: 29.2 Stage 4: 18.9 Stage 5: 1.9 | NR | NR | NR | NR |
|  |  | sK+ 3.5 to 4.0 mEq/L | NA | 18,968 | NR | NR | Stage 3a: 52.9 Stage 3b: 31.0 Stage 4: 14.9 Stage 5: 1.1 | NR | NR | NR | NR |
| RRI-CKD Cohort study Korgaonkar 2010 ([91](#_ENREF_91)) | Patients with stage 3-5 CKD + Hyperkalaemia | sK+ ≥5.5 mmol/L | NA | 65 | ACEi: 66 ARBs: 11 β-blockers: 54 Diuretics: 52 CCB: 35 K+ binders: 66 Aspirin: 42 Erythropoietin: 29 Statins: 52 | NR | Stage 3 to 5: 100 | Mean (SD): 23.2 (10.1) | NR | Mean (SD): 3.7 (0.5) | Mean (SD): 5.8 (0.3) |
|  | Patients with stage 3-5 CKD + Normokalaemia | sK+ >4.0 and <5.5 mmol/L | NA | 633 | ACEi: 41 ARBs: 23 β-blockers: 47 Diuretics: 50 CCB: 46 K+ binders: 41 Aspirin: 37 Erythropoietin: 24 Statins: 44 | NR | Stage 3 to 5: 100 | Mean (SD): 25.3 (9.9) | NR | Mean (SD): 3.8 (0.5) | Mean (SD): 4.7 (0.4) |
|  | Patients with stage 3-5 CKD + Hypokalaemia | sK+ ≤4.0 mmol/L | NA | 122 | ACEi: 28 ARBs: 29 β-blockers: 51 Diuretics: 61 CCB: 52 K+ binders: 28 Aspirin: 34 Erythropoietin: 25 Statins: 32 | NR | Stage 3 to 5: 100 | Mean (SD): 27 (13.7) | NR | Mean (SD): 3.7 (0.5) | Mean (SD): 3.7 (0.3) |
| Hwang 2011 ([66](#_ENREF_66)) | Patients with ESRD under maintenance haemodialysis + Hyperkalaemia | sK+ >5.5 mEq/L | NA | 51 | ACEi and/or ARB: 27 | DM: 41 CAD: 20 Congestive HF: 8 PVD: 12 Stroke: 8 Neoplasm: 8 Chronic lung disease: 0 Liver cirrhosis or hepatoma: 2 No comorbidity: 61 | NR | NR | Mean (SD): 10.9 (2.5) | Mean (SD): 3.6 (0.5) | Mean (SD): 6.0 (0.5) |
|  | Patients with ESRD under maintenance haemodialysis + Normokalaemia | sK+ 3.5 to 5.5 mEq/L | NA | 326 | ACEi and/or ARB: 27 | DM: 38 CAD: 17 HF: 2 PVD: 6 Stroke: 11 Neoplasm: 7 Chronic lung disease: 1 Liver cirrhosis or hepatoma: 3 No comorbidity: 60 | NR | NR | Mean (SD): 9.8 (2.6) | Mean (SD): 4.0 (0.4) | Mean (SD): 4.4 (0.6) |
|  | Patients with ESRD under maintenance haemodialysis + Hypokalaemia | sK+ <3.5 mEq/L | NA | 46 | ACEi and/or ARB: 26 | DM: 52 CAD: 28 HF: 7 PVD: 9 Stroke: 20 Neoplasm: 13 Chronic lung disease: 0 Liver cirrhosis or hepatoma: 9 No comorbidity: 41 | NR | NR | Mean (SD): 8.1 (2.4) | Mean (SD): 3.6 (0.5) | Mean (SD): 3.1 (0.4) |
| Brookes 2021 ([110](#_ENREF_110)) | Patients with stage 1-5 non-dialysis dependent CKD + Hyperkalaemia | sK+ ≥6.0 mmol/L | NA | 297 | ACEi: 12.8 ARBs: 7.7 MRAs: 7.1 β-blockers: 37.4 Loop diuretics: 41.4 Thiazide diuretics: 8.4 CCB: 30.6 K+ binders: 12.8 | HF: 12.1 DM: 56.6 MI: 2.4 PVD: 2.4 CBD: 0.7 Dementia: 2.4 Pulmonary disease: 3.7 Rheumatic disease: 0.7 Peptic ulcer disease: 1.0 Liver disease: 7.7 Hemi/paraplegia: 0.7 Malignancy: 7.4 HIV/AIDS: 0.0 | Stage 1: 0.3 Stage 2: 3.4 Stage 3a: 10.8 Stage 3b: 27.6 Stage 4: 32.0 Stage 5: 25.9 | Mean (SD): 27.7 (16.9)  Median (IQR): 26.0 (14 to 38) | NR | NR | NR |
|  |  | sK+ 5.5 to 5.9 mmol/L | NA | 473 | ACEi: 17.3 ARBs: 15.4 MRAs: 9.3 β-blockers: 33.6 Loop diuretics: 32.4 Thiazide diuretics: 6.8 CCB: 24.3 K+ binders: 17.3 | HF: 12.7 DM: 45.5 MI: 4.0 PVD: 0.9 CBD: 2.1 Dementia: 1.3 Pulmonary disease: 5.7 Rheumatic disease: 0.6 Peptic ulcer disease: 1.9 Liver disease: 7.2 Hemi/paraplegia: 1.1 Malignancy: 7.0 HIV/AIDS: 0.0 | Stage 1: 0.2 Stage 2: 3.8 Stage 3a: 17.6 Stage 3b: 38.3  Stage 4: 28.5 Stage 5: 11.6 | Mean (SD): 33.5 (15.6); median (IQR): 33 (22 to 43) | NR | NR | NR |
|  | Patients with stage 1-5 non-dialysis dependent CKD + Normokalaemia | sK+ 5.0 to 5.4 mmol/L | NA | 1,503 | ACEi: 18.4 ARBs: 19.2 MRAs: 10.3 β-blockers: 35.2 Loop diuretics: 33.1 Thiazide diuretics: 7.4 CCB: 21.9 K+ binders: 18.4 | HF: 12.0 DM: 39.2 MI: 2.6 PVD: 1.8 CBD: 2.9 Dementia: 2.6 Pulmonary disease: 6.2 Rheumatic disease: 0.3 Peptic ulcer disease: 1.1 Liver disease: 6.4 Hemi/paraplegia: 1.7 Malignancy: 9.2 HIV/AIDS: 0.0 | Stage 1: 0.4 Stage 2: 8.3 Stage 3a: 31.6 Stage 3b: 31.7 Stage 4: 21.9 Stage 5: 6.1 | Mean (SD): 40.5 (16.3) Median (IQR): 41 (29 to 52) | NR | NR | NR |
|  |  | sK+ 4.0 to 4.9 mmol/L | NA | 7,235 | ACEi: 18.0 ARBs: 18.0 MRAs: 7.6 β-blockers: 32.7 Loop diuretics: 29.8 Thiazide diuretics: 9.7 CCB: 20.3 K+ binders: 18.0 | HF: 10.6 DM: 30.5 MI: 2.9 PVD: 1.8 CBD: 4.1 Dementia: 2.4 Pulmonary disease: 5.3 Rheumatic disease: 0.9 Peptic ulcer disease: 0.6 Liver disease: 4.1 Hemi/paraplegia: 1.7 Malignancy: 8.0 HIV/AIDS: 0.0 | Stage 1: 0.7 Stage 2: 15.5 Stage 3a: 43.2 Stage 3b: 27.9 Stage 4: 10.7 Stage 5: 2.1 | Mean (SD): 47.7 (15.5) Median (IQR): 48 (38 to 57) | NR | NR | NR |
|  |  | sK+ 3.5 to 3.9 mmol/L | NA | 1,341 | ACEi: 16.1 ARBs: 17.8 MRAs: 7.0 β-blockers: 33.4 Loop diuretics: 33.0 Thiazide diuretics: 15.5 CCB: 22 K+ binders: 16.1 | HF: 9.7 DM: 23.3 MI: 2.8 PVD: 1.7 CBD: 4.0 Dementia: 2.8 Pulmonary disease: 5.4 Rheumatic disease: 0.5 Peptic ulcer disease: 1.0 Liver disease: 6.8 Hemi/paraplegia: 1.5 Malignancy: 9.0 HIV/AIDS: 1.0 | Stage 1: 1.0 Stage 2: 17.7 Stage 3a: 43.0 Stage 3b: 26.8 Stage 4: 9.8 Stage 5: 1.8 | Mean (SD): 49.1 (16.5) Median (IQR): 50 (38 to 58) | NR | NR | NR |
|  | Patients with stage 1-5 non-dialysis dependent CKD + Hypokalaemia | sK+ <3.5 mmol/L | NA | 307 | ACEi: 15.3 ARBs: 16.6 MRAs: 9.8 β-blockers: 31.3 Loop diuretics: 37.1 Thiazide diuretics: 18.6 CCB: 26.7 K+ binders: 15.3 | HF: 9.8 DM: 24.1 MI: 0.6 PVD: 2.6 CBD: 2.9 Dementia: 4.2 Pulmonary disease: 6.5 Rheumatic disease: 1.0 Peptic ulcer disease: 0.7 Liver disease: 10.1 Hemi/paraplegia: 1.6 Malignancy: 11.1 HIV/AIDS: 0.0 | Stage 1: 1.6 Stage 2: 19.2 Stage 3a: 34.9 Stage 3b: 26.71 Stage 4: 13.0 Stage 5: 4.6 | Mean (SD): 48.1 (19.1)  Median (IQR): 47 (36 to 58) | NR | NR | NR |
| Shang 2022 ([35](#_ENREF_35)) | Patients with CKD + HF + Hyperkalaemia | sK+ >4.7 mmol/L | NA | 53 | NR | HF: 100 | NR | NR | NR | NR | NR |
|  | Patients with CKD + HF + Normokalaemia | sK+ 3.5 to 4.7 mmol/L | NA | 145 | NR | HF: 100 | NR | NR | NR | NR | NR |
|  | Patients with CKD + HF + Hypokalaemia | sK+ <3.5 mmol/L | NA | 26 | NR | HF: 100 | NR | NR | NR | NR | NR |
| Jime´nez-Marrero 2021 ([44](#_ENREF_44)) | Patients with CKD + Hyperkalaemia | sK+ >5.0 mEq/L | NA | 815 | NR | NR | NR | NR | NR | NR | NR |
|  | Patients with CKD + Normokalaemia | sK+ ≥3.5 and ≤5.0 mEq/L | NA | 12,491 | NR | NR | NR | NR | NR | NR | NR |
|  | Patients with CKD + Hypokalaemia | sK+ <3.5 mEq/L | NA | 170 | NR | NR | NR | NR | NR | NR | NR |
| Fukushima CKD Cohort Tanaka 2021 ([80](#_ENREF_80)) | Pre-dialysis CKD patients with eGFR <60 mL/min/1.73 m2 + Hyperkalaemia | sK+ ≥5.0 mmol/L | NA | 141 | MRAs: 8.5 β-blockers: 18.4 Diuretics: 22 ARBs or ACEi: 72.3 Cation exchange resin: 31.9 | HT: 90.1 DM: 53.2 | NR | Median (IQR): 33.9 (22.0 to 48.4) | Median (IQR): 1.5 (1.1 to 2.3) | Median (IQR): 4 (3.7 to 4.2) | Median (IQR): 5.2 (5.0 to 5.4) |
|  | Pre-dialysis CKD patients with eGFR <60 mL/min/1.73 m2 + Normokalaemia | sK+ 4.5 to 4.9 mmol/L | NA | 433 | MRAs: 8.3 β-blockers: 18.7 Diuretics: 22.6 ARBs or ACEi: 67.9 Cation exchange resin: 7.6 | HT: 86.6 DM: 52.7 | NR | Median (IQR): 48.6 (35.8 to 58.1) | Median (IQR): 1.1 (0.9 to 1.4) | Median (IQR): 4 (3.7 to 4.2) | Median (IQR): 4.7 (4.6 to 4.8) |
|  |  | sK+ 4.0 to 4.4 mmol/L | NA | 539 | MRAs: 7.1 β-blockers: 14.3 Diuretics: 23.7 ARBs or ACEi: 70.1 Cation exchange resin: 2.6 | HT: 85.2 DM: 47.5 | NR | Median (IQR): 55.3 (43.5 to 69.2) | Median (IQR): 1.0 (0.8 to 1.2) | Median (IQR): 4 (3.7 to 4.2) | Median (IQR): 4.2 (4.1 to 4.3) |
|  | Pre-dialysis CKD patients with eGFR <60 mL/min/1.73 m2 + Hypokalaemia | sK+ <4.0 mmol/L | NA | 217 | MRAs: 6.9 β-blockers: 8.8 Diuretics: 28.1 ARBs or ACEi: 58.5 Cation exchange resin: 3.2 | HT: 78.8 DM: 35.9 | NR | Median (IQR): 58.5 (46.9 to 75.1) | Median (IQR): 0.9 (0.7 to 1.1) | Median (IQR): 3.9 (3.6 to 4.2) | Median (IQR): 3.8 (3.6 to 3.9) |
| Li 2023b ([67](#_ENREF_67)) | Patients with stage 3-5 CKD + HFpEF | sK+ >5.5 mmol/L | MRA (spironolactone) | 154 | Any RAASi: 55.2 β-blockers: 55.8 Diuretics: 92.9 Statin: 81.8 | HF:100 HT: 82.5 DM: 29.2 | Stage 3a: 30.5 Stage 3b: 38.3 CKD stage 4/5: 31.2 | Mean (SD): 36.6 (12.5) | Median (range): 1.6 (1.3 to 1.9) | NR | Median (IQR): 4 (3.6 to 4.3) |
|  |  |  | No Spironolactone | 233 | Any RAASi: 39.1 β-blockers: 45.9 Diuretics: 54.9 Statin: 77.7 | HF:100 HT: 82.8 DM: 45.5 | Stage 3a: 23.6 Stage 3b: 25.8 CKD stage 4/5: 50.6 | Mean (SD): 29.3 (16.6) | Median (range): 1.9 (1.4 to 3.6) | NR | Median (IQR): 4.1 (3.7 to 4.5) |
| Kanda 2023 ([117](#_ENREF_117)) | Patients with stage 3 or 4 CKD with or without HF | ICD-10 diagnostic code of E87.5 or ICD-9 diagnostic code of 276.7 | ACEi/ARB/ARNi/MRA | 1,427 | ACEi: 15.9 ARBs: 77.3 MRAs: 27 ARNI: <11 K+ binders: 15.9 | HF: 52.9 DM: 43.3 | Stage 3: 37.4 Stage 4: 62.6 | NR | NR | NR | NR |
|  |  |  |  | 11,873 | ACEi: 56.7 ARBs: 32.2 MRAs: 22.3 ARNI: 3.8 K+ binders: 56.7 | HF: 46.1 DM: 68.5 | Stage 3: 75.2 Stage 4: 24.8 | NR | NR | NR | NR |
| Lin 2023 ([74](#_ENREF_74)) | Patients with ESRD under maintenance dialysis + HF | ICD-9 diagnostic code of 276.7 or  sK+ >6 mEq/L | MRA | 2,176 | Any RAASi: 53.8 β-blockers: 52.4 Loop diuretics: 42.7 Thiazide diuretics: 4.4 Non-dihydropyridine CCB: 46.6 Dihydropyridine CCB: 46.4 Peritoneal dialysis: 15.2 Haemodialysis: 84.8 K+ binders: 9.5 Antiplatelet: 41.7 Anticoagulant: 4.6 Amiodarone/dronedarone: 6.5 Oral hypoglycaemic agents: 22.9 Insulin: 22.8 Statin: 18.3 | HF:100 HT: 77.5 DM: 48 Hyperlipidaemia: 20.2 CAD: 48.2 AFib: 7.0 PAD: 8.2 COPD: 11.1 Cancer: 9.7 Abnormal liver function: 19.7 Liver cirrhosis: 8.0 CVD: 59.9 | NR | NR | NR | NR | NR |
|  |  |  | Non-MRA | 6,528 | Any RAASi: 54.7 β-blockers: 53 Loop diuretics: 43.4 Thiazide diuretics: 4.2 Non-dihydropyridine CCB: 48.1 Dihydropyridine CCB: 47.9 Peritoneal dialysis: 15.1 Haemodialysis: 84.9 Antiplatelet: 42.4 K+ binders: 9.5 Anticoagulant: 4.5 Amiodarone/dronedarone: 6.0 Oral hypoglycaemic agents: 22.8 Insulin: 23.2 Statin: 18.6 | HF:100 HT: 78.4 DM: 48.8 Hyperlipidaemia: 20.6 CAD: 49.0 AFib: 6.8 PAD: 7.9 COPD: 11.4 Cancer: 9.2 Abnormal liver function: 18.9 Liver cirrhosis: 7.6 CVD: 60.0 | NR | NR | NR | NR | NR |
| MRA-ACE Trial Tumlin 2022 ([21](#_ENREF_21)) | Patients with diabetic kidney disease | sK+ >5.5 mmol/L | Maximum tolerated ACE/ARB | 18 | NR | DM: 100 | NR | NR | NR | NR | NR |
|  |  |  | Maximum tolerated ACE/ARB + MRA (spironolactone -25 mg) | 14 | NR | NR | NR | NR | NR | NR | NR |
| Sadjadi 2009 ([45](#_ENREF_45)) | Patients with stage 1-5 CKD | sK+ >5.0 mEq/L | ACEi (lisinopril/benazepril/fosinopril/enalapril/captopril) | 1,163 | ACEi: 100 ARBs: NA K+ sparing diuretics: 6.1 Loop diuretics: 14.7 Thiazide diuretics: 26.7 K+ binders: 100 K+ chloride: 6 NSAIDS: 17 | HT: 91.7 HF: 9.5 DM: 51.5 | Stage 1: 16.2 Stage 2: 51.4 Stage 3: 30.1 Stage 4: 1.7 Stage 5: 0.6 | Mean (SD): 69 (21.4) | Mean (SD): 1.25 (0.6) | NR | NR |
|  |  |  | ARB (irbesartan/losartan) | 1,168 | ACEi: NA ARBs: 100 K+ sparing diuretics: 11.1 Loop diuretics: 24.4 Thiazide diuretics: 28.4 K+ chloride: 13.1 NSAIDS: 17.5 | HT: 92.9 HF: 17.1 DM: 52.1 | Stage 1: 8.2 Stage 2: 46 Stage 3: 39.9 Stage 4: 4.6 Stage 5: 1.3 | Mean (SD): 60.9 (20.7) | Mean (SD): 1.47 (1.0) | NR | NR |
| Khosla 2009 ([68](#_ENREF_68)) | Patients with stage 2 or 3 CKD + Resistant HT | sK+ >5.5 mEq/L sK+ ≥6 mEq/L | ACEi (lisinopril/ramipril) or ARB (valsartan/olmesartan) or ACEi + ARB | 46 | Hydrochlorothiazide or chlorthalidone: 84.8 Furosemide: 15.2 | HT: 100 DM: 86.9 | CKD stage 2 or 3: 100 | Mean (SD): 56.5 (16.2) | Mean (SD): 1.5 (0.4) | NR | Mean (SD): 4.1 (0.3) |
| AASK Weinberg 2009 ([22](#_ENREF_22)) | Patients with non-diabetic hypertensive CKD (eGFR 20-65 mL/min/1.73 m2) | sK+ >5.5 mEq/L | ACEi (ramipril) | 417 | ACEi: 41.5 Diuretics: 64.9 K+ binders: 41.5 NSAID: 10.8 | HT: 100 | NR | Mean (SD): 46.3 (13.5) | Male: 2.2 (0.7) Female: 1.8 (0.6) | NR | Mean (SD): 4.2 (0.6) |
|  |  |  | β-blocker (metoprolol succinate) | 428 | ACEi: 35.3 Diuretics: 62.6 K+ binders: 35.3 NSAID: 12.1 | HT: 100 | NR | Mean (SD): 46.7 (13.9) | Male: 2.1 (0.8) Female: 1.8 (0.6) | NR | Mean (SD): 4.2 (0.6) |
|  |  |  | CCB (amlodipine besylate) | 208 | ACEi: 42.8 Diuretics: 66.7 K+ binders: 42.8 NSAIDS: 8.7 | HT: 100 | NR | Mean (SD): 46.8 (13.1) | Male: 2.3 (0.8) Female: 1.7 (0.6) | NR | Mean (SD): 4.3 (0.7) |
| Knoll 2002 ([92](#_ENREF_92)) | Patients with ESRD under chronic haemodialysis | sK+ ≥5.5 mmol/L sK+ ≥ 6.0 mmol/L | ACEi (enalapril/fosinopril/captopril) or ARB (losartan) | 71 | β-blockers: 39 Insulin or oral hypoglycaemic agent: 23 NSAIDS: 11 β-agonist: 4 Digoxin: 7 Diuretic: 1 | DM: 24 | NR | NR | NR | NR | NR |
|  |  |  | Control | 180 | β-blockers: 31 Insulin or oral hypoglycaemic agent: 15 NSAIDS: 11 β-agonist: 7 Digoxin: 6 Diuretic: 5 | DM: 19 | NR | NR | NR | NR | NR |
| Hayashi 2003 ([23](#_ENREF_23)) | Patients with chronic renal parenchymal disease + HT | NR | ACEi (enalapril/lisinopril/imidapril) | 20 | NR | HT: 100 | NR | NR | Mean (SD): 1.9 (0.4) | NR | Mean (SD): 4.5 (0.4) |
|  |  |  | CCB (efonidipine) | 23 | NR | HT: 100 | NR | NR | Mean (SD): 1.8 (0.5) | NR | Mean (SD): 4.3 (0.5) |
| Adelborg 2019 ([46](#_ENREF_46)) | Patients with CKD | sK+ >5.0 mmol/L | ACEi/ARB/MRA (spironolactone) | NR | NR | NR | NR | NR | NR | NR | NR |
| Sengul 2009 ([93](#_ENREF_93)) | Patients with CKD | sK+ ≥5.5 mEq/L | MRA (spironolactone) | 33 | ACEi: 60.6 ARBs: 33.3 Any RAASi: 6.1 K+ binders: 60.6 | NR | NR | NR | Mean (SD): 1.1 (0.4) | Mean (SD): 3.9 (0.5) | Mean (SD): 4.5 (0.4) |
| Abolghasmi 2011 ([24](#_ENREF_24)) | Patients with moderately severe CKD (eGFR 25-50 mL/min/1.73m²) + Resistant HT | sK+ >5.5 mEq/L | MRA (spironolactone) | 19 | NR | HT: 100 | NR | NR | Mean (SD): 2.2 (0.8) | NR | Mean (SD): 4.3 (0.8) |
|  |  |  | Placebo | 22 | NR | HT: 100 | NR | NR | Mean (SD): 2.3 (0.6) | NR | Mean (SD): 4.5 (0.6) |
| Fröhlich 2016 ([69](#_ENREF_69)) | Patients with stable stage 3 or 4 CKD + HF | sK+ >5.5 mmol/L | ACEi/ARB | 722 | ACEi: 77 ARBs: 23 MRAs: 49 β-blockers: 79 Loop diuretics: 82 K+ binders: 77 Digitalis: 25 Aspirin: 22 Warfarin: 52 Statin: 60 Allopurinol: 17 | HF:100 HT: 59 DM: 30 COPD: 18 | CKD stage 3 or 4: 100 | Mean (SD): 47 (10) | NR | NR | Mean (SD): 5 (1) |
| EMPHASIS-HF Eschalier 2013 ([25](#_ENREF_25)) | Patients with CKD with eGFR <60 mL/min/1.73 m2 | sK+ >5.5 mmol/L  sK+ >6.0 mmol/L | MRA (eplerenone) | 439 | ACEi: 77.9 ARBs: 28.3 Any RAASi: 95.4 β-blockers: 88.2 K+ binders: 77.9 Diuretic: 91.1 | HT: 69.2 DM: 38 MI: 54.9 | NR | Mean (SD): 48.6 (7.9) | Mean (SD): 1.4 (0.3) | NR | Mean (SD): 4.4 (0.4) |
|  |  |  | Placebo | 473 | NR | NR | NR | NR | NR | NR | NR |
| Polson 2017 ([118](#_ENREF_118)) | Patients with CKD | ICD-10 diagnostic code of E87.5  ICD-9 diagnostic code of 276.7 | RAASi | 2,774 | NR | NR | NR | NR | NR | NR | NR |
|  |  |  | No RAASi | 3,423 | NR | NR | NR | NR | NR | NR | NR |
|  | Patients with CKD + HF |  | RAASi | 691 | NR | NR | NR | NR | NR | NR | NR |
|  |  |  | No RAASi | 986 | NR | NR | NR | NR | NR | NR | NR |
| Wetmore 2021 ([75](#_ENREF_75)) | Patients with stage 1-5 CKD with previous history of hyperkalaemia | sK+ 5.0 to ≤5.5 mmol/L  sK+ >5.5 to ≤6.0 mmol/L  sK+ >6.0 mmol/L | RAASi | 46,733 | NR | NR | Stage 1: 2.4 Stage 2: 17.5 Stage 3: 72.6 Stage 4: 6.4 Stage 5: 1.2 | NR | NR | NR | NR |
|  | Patients with stage 1-5 CKD without previous history of hyperkalaemia |  |  | 39,814 | NR | NR | Stage 1: 3.0 Stage 2: 24.7 Stage 3: 69.7 Stage 4: 2.2 Stage 5: 0.4 | NR | NR | NR | NR |
| Yang 2023 ([94](#_ENREF_94)) | Patients with advanced CKD + DM | pK+ ≥5.5 mmol/L | Continued RAASi users | 3,817 | Insulin: 49.0 Metformin: 70.0 Sulfonylureas: 59.0 α-glucosidase inhibitors: 2.4 Thiazolidinediones: 6.2 Dipeptidyl peptidase 4 inhibitors: 17.1 Glucagon-like peptide-1 receptor analogues: 0.7 Sodium-glucose co-transporter 2 inhibitors: 1.1 Glucose-lowering drugs, 0: 5.1 Glucose-lowering drugs, 1: 21.8 Glucose-lowering drugs, 2: 43.5 Glucose-lowering drugs, 3: 22.4 Glucose-lowering drugs, ≥4: 7.1 Statins: 64.2 | DM: 100 HF: 10.3 CVD: 35.6 IHD: 14.0 MI: 4.6 Stroke: 12.8 Cancer: 6.7 PVD: 2.4 | NR | Mean (SD): 26.2 (3.5) | NR | NR | Mean (SD): 4.5 (0.7) |
|  |  |  | Discontinued RAASi users | 583 | Insulin: 42.5 Metformin: 73.2 Sulfonylureas: 61.9 α-glucosidase inhibitors: 0.9 Thiazolidinediones: 5.3 Dipeptidyl peptidase 4 inhibitors: 21.4 Glucagon-like peptide-1 receptor analogues: 0.9 Sodium-glucose co-transporter 2 inhibitors: 3.1 Glucose-lowering drugs, 0: 6.0 Glucose-lowering drugs, 1: 20.1 Glucose-lowering drugs, 2: 42.2 Glucose-lowering drugs, 3: 23.7 Glucose-lowering drugs, ≥4: 8.1 Statins: 59.9 | HF: 10.5 CVD: 36.5 IHD: 14.9 MI: 3.9 Stroke: 14.1 Cancer: 11.3 PVD: 2.2 | NR | Mean (SD): 24.7 (4.3) | NR | NR | Mean (SD): 4.7 (0.8) |
| Buckallew 2021 ([95](#_ENREF_95)) | Patients with stage 3-5 CKD + HF | sK+ ≥5.5 mEq/L | MRA (spironolactone) | 121 | ACEi: 56.5 ARBs: 28.3 ARNI: 15.2 β-blockers: 73.6 Loop diuretics: 90.0 Peritoneal dialysis: 2.5 Haemodialysis: 3.3 K+ binders: 56.5 Hydralazine: 31.4 | HT: 91.7 HF: 100 AFib: 64.5 | Stage 3: 69.4 Stage 4: 27.5 Stage 5: 5.8 | NR | NR | NR | NR |
| De Rosa 2002 ([76](#_ENREF_76)) | Patients with mild (CrCL 30-60 mL/min/1.73 m2) or moderate to severe (CrCL 10-29 mL/min/1.73 m2) chronic renal insufficiency + HT | sK+ >6.0 mEq/L | ARB (irbesartan with or without concomitant antihypertensive therapy) | 32 | NR | HT: 100 DM: 28 | NR | NR | NR | NR | NR |
| Ren 2022 ([96](#_ENREF_96)) | Patients with stage 3-5 CKD + de novo hyperkalaemia episode | sK+ ≥5.5 mmol/L | RAASi | 10,009 | MRAs: Current users: 16.7 Non-users: 83.3 β-blockers: Current users: 48.8 Non-users: 51.2 Other diuretics Current users: 43.4 Non-users: 56.6 Azole  Current users: 0.3 Non-users: 99.7 Calcineurin inhibitors Current users: 0 Non-users: 100 Digoxin Current users: 8.8 Non-users: 91.2 Heparin Current users: 0.7 Non-users: 99.3 Prescription NSAIDs Current users: 4.4 Non-users: 95.6 K+ supplements:  -Current users: 6.1  -Non-users: 93.9 Trimethoprim Current users: 4.3 Non-users: 95.7 | HT: 98.9 HF: 45.7 DM: 61.7 Unstable angina: 19.9 Stroke: 20.5 AFib: 36.0 | Stage 3a: 49.3 Stage 3b: 31.1 Stage 4: 15.8 Stage 5: 3.9 | NR | NR | NR | Median (IQR): 5.7 (5.5 to 10) |
| Riccio 2022 ([70](#_ENREF_70)) | Patients with stage 1-5 CKD | sK+ >5.5 mmol/L | ACEi/ ARB/ ACEi + ARB combination | 556 | ACEi: 45 ARBs: 37 Any RAASi: 18 K+ binders: 45 | HF: 8.8 DM: 14.7 | Stage 1: 10.2 Stage 2: 19.1 Stage 3: 34.9 Stage 4: 20.5 Stage 5: 15.3 | Median (IQR): 40.3 (45.5) | NR | NR | Median (IQR): 4.7 (0.8) |
| EX-DKD Study Uchida 2022 ([97](#_ENREF_97)) | Patients with diabetic kidney disease + HT | sK+ ≥5.5 mEq/L  sK+ ≥6.0 mEq/L | MRA (esaxerenone) | 109 | RAS inhibitor: 33 RAS inhibitor + CCB: 67 | HT: 100 DM: 100 Diabetic retinopathy: 20.2 Diabetic neuropathy: 18.3 Dyslipidaemia: 73.4 Hyperuricemia: 29.4 | NR | Mean (SD): 49.4 (7.6) | NR | NR | Mean (SD): 4.3 (0.3) |
| Santoro 2022 ([98](#_ENREF_98)) | Patients with stage ≤3 and >3 CKD and unspecified stage + Hyperkalaemia | sK+ ≥5.5 mmol/L | RAASi drugs taken into consideration:  1. ACEi – plain 2. ACEi – combinations 3. ARBs -plain  4. ARB combinations 5. Other agents acting on renin-angiotensin system | 1,071 | Previous RAASi use: 92 Previous drug treatments Diuretics: 63.9 Aldosterone antagonists: 13.4 β blocking agents: 41.3 Lipid modifying agents: 50.3 Anti-diabetics: 44.2 | NR | Stage ≤3: 51.5 Stage >3: 37.8 Unspecified: 10.7 | NR | NR | NR | NR |
| DRINK Tang 2021 ([11](#_ENREF_11)) | Patients with stage 3 or 4 non-diabetic CKD | pK+ ≥5.5 mmol/L | ARB (losartan) + DRI (aliskiren) | 37 | ACEi + losartan: 32 K+ binders + losartan: 32 Losartan + thiazide: 8 Losartan + β-blocker: 11 Losartan + CCB: 11 Losartan + β-blocker + CCB: 19 Losartan + thiazide + CCB: 3 Losartan + thiazide + β-blocker: 5 Losartan + thiazide + β-blocker + CCB: 11 | Renal disease: Primary glomerular: 46 Hypertensive: 35 Others: 19 | Stage 3-4: 100 | Mean (SD): 31.9 (9) | Mean (SD): 7.5 (2.4) | Mean (SD): 4.3 (0.3) | Mean (SD): 4.3 (0.5) |
|  |  |  | ARB (Losartan) | 39 | ACEi + Losartan: 31 K+ binders + losartan: 31 Losartan + thiazide: 8 Losartan + β-blocker: 15 Losartan + CCB: 8 Losartan + β-blocker + CCB: 23 Losartan + thiazide + CCB: 5 Losartan + thiazide + β-blocker: 8 Losartan + thiazide + β-blocker + CCB: 3 | Renal disease: Primary glomerular: 44 Hypertensive: 26 Others, unknown: 31 | Stage 3-4: 100 | Mean (SD): 27.7 (9) | Mean (SD): 8.3 (2.5) | Mean (SD): 4.3 (0.4) | Mean (SD): 4.4 (0.6) |
| BLOCK-CKD Bakris 2021 ([12](#_ENREF_12)) | Patients with stage 3b or 4 CKD + Uncontrolled grade 1 and 2 systolic HT | sK+: 5.6 to 5.9 mmol/L  sK+ ≥5.6 mmol/L  sK+ ≥6.0 mmol/L | MRA (KBP-5074 – 0.25 mg) | 51 | β-blockers: 70.6 Thiazide diuretics: 76.5 Loop diuretics: 35.3 CCB: 51 ACEi/ARB: 92.2 α-blockers: 21.6 Drugs used in DM: 56.9 Oral hypoglycaemic agents: 52.9 SGLT2 inhibitors: 0 Insulin: 19.6 GLP-1: 2 Serum lipid-modifying agents: 51 | HT: 100 | Stage 3b: 60.8 Stage 4: 39.2 | NR | NR | NR | Mean (SD): 4.3 (0.4) |
|  |  |  | MRA (KBP-5074 – 0.5 mg) | 54 | β-blockers: 63 Thiazide diuretics: 51.9 Loop diuretics: 51.9 CCB: 70.4 ACEi/ARB: 85.2 α-blockers: 38.9 Drugs used in DM: 61.1 Oral hypoglycaemic agents: 42.6 SGLT2 inhibitors: 0 Insulin: 29.6 GLP-1: 1.9 Serum lipid-modifying agents: 57.4 | HT: 100 | Stage 3b: 64.8 Stage 4: 35.2 | NR | NR | NR | Mean (SD): 4.4 (0.5) |
|  |  |  | Placebo | 57 | β-blockers: 56.1 Thiazide diuretics: 78.9 Loop Diuretics: 43.9 CCB: 56.1 ACEi/ARB: 93 α-blockers: 28.1 Drugs used in DM: 52.6 Oral hypoglycaemic agents: 38.6 SGLT2 inhibitors: 1.8 Insulin: 28.1 GLP-1: 3.5 Serum lipid-modifying agents: 47.4 | HT: 100 | Stage 3b: 56.1 Stage 4: 43.9 | NR | NR | NR | Mean (SD): 4.4 (0.4) |
| Einhorn 2009 ([111](#_ENREF_111)) | Patients with stage 3-5 CKD | sK+ ≥5.5 to 6.0 mg/dL  sK+ ≥6.0 mg/dL | ACEi and/or ARBs/ No RAASi | 70,873 | ACEi/ARB:  Either: 62.1 Both: 3.4 | DM: 50.4 Cancer: 27.5 CVD: 53.2 | Stage 3: 81.6  Stage 4: 11.8 Stage 5: 6.7 | NR | NR | NR | NR |
| Obertynska 2021 ([112](#_ENREF_112)) | Patients with stage 3 CKD + HFrEF <40% | sK+ 5.5 to 5.9 mmol/L or sK+ ≥6.0 mmol/L | MRA (spironolactone) | 208 | NR | HF:100 | Stage 3: 100 | NR | NR | NR | NR |
| Johnson 2010 ([99](#_ENREF_99)) | Patients with stage 3-5 CKD | sK+ ≥5.5 mmol/L or ICD-9 code | ACEi (lisinopril) | 5,171 | ACEi (lisinopril 10 mg per day: 89.1) ACEi (lisinopril >10 mg per day: 10.9 K+ supplements: 25.8 ARBs: 25.0 | HF: 18 DM: 26.2 | Stage 3a: 77.8 Stage 3b: 19.2 Stage 4: 2.8 Stage 5: 0.2 | Mean (SD): 50.2 (8.5) | NR | NR | NR |
| Raebel 2010 ([113](#_ENREF_113)) | Patients with stage 3-4 CKD + DM | sK+ ≥6.0 mmol/L or ICD-9 diagnostic code of 276.7 | ACEi/ARB/MRA (spironolactone) | 2,176 | NR | DM: 100 | Stage 3-4: 100 | NR | NR | NR | NR |
| Frimodt-Moller 2010 ([13](#_ENREF_13)) | Pre-dialysis stage 3-5 CKD patients | pK+ >5.5mmol/L | ACEi (enalapril) + ARB (candesartan) after 16 weeks of monotherapy with either enalapril or candesartan | 47 | ACEi: 36.2 ARBs: 42.6 β-blockers: 40.4 CCB: 51.1 K+ binders: 36.2 Diuretics: 63.8 | DM: 17.0 CVD: 27.7 | Stage 3-5: 100 | NR | NR | NR | NR |
| Taheri 2012 ([14](#_ENREF_14)) | Patients on chronic ambulatory peritoneal dialysis + Advanced HF | sK+ >5.7 mEq/L | MRA (spironolactone) | 9 | NR | HF:100 | NR | NR | NR | NR | Mean (SD): 4.6 (0.4) |
|  |  |  | Placebo | 9 | NR | HF:100 | NR | NR | NR | NR | Mean (SD): 4.4 (0.8) |
| Espinel 2012 ([15](#_ENREF_15)) | Patients with stage 3 CKD | sK+ ≥5.0 mmol/L | ARB (olmesartan) | 17 | NR | DM: 5.8 Dyslipidaemia: 35.3 | Stage 3: 100 | Mean (SD): 42.24 (2) | Mean (SD): 1.6 (0.1) | NR | NR |
|  |  |  | ACEi (enalapril) | 13 | NR | DM: 15 Dyslipidaemia: 38.5 | NR | Mean (SD): 46.2 (1.9) | Mean (SD): 1.6 (0.1) | NR | NR |
| Boesby 2011 ([16](#_ENREF_16)) | Patients with stage 1-4 non-diabetic CKD | pK+ >5.5 mEq/L | MRA (eplerenone) vs control | 40 | ACEi: 57.5 ARBs: 20 Any RAASi: 17.5 β-blockers: 15 CCB: 25 K+ binders: 57.5 Furosemide: 33.3 Diuretic: 15 | Chronic glomerulonephritis: 61.9 Vascular disease: 4.8 ADPKD: 2.4 CKD of unknown aetiology: 26.1 | Stage 1-4: 100 | NR | NR | NR | NR |
| CRIB-II study Edwards 2012 ([17](#_ENREF_17)) | Patients with stage 2-3 CKD | sK+ ≥5.5 mmol/L  sK+ ≥6 mmol/L | MRA (spironolactone) | 56 | ACEi: 66.1 ARBs: 32.1 Any RAASi: 1.8 β-blockers: 26.8 CCB: 23.2 K+ binders: 66.1 Diuretics: 32.1 | NR | Stage 2-3 100 | Mean (SD): 49 (12) | Mean (SD): 5.1 (1.4) | NR | Mean (SD): 4.4 (0.8) |
|  |  |  | Placebo | 56 | ACEi: 66.1 ARBs: 30.4 Any RAASi: 3.6 β-blockers: 14.3 CCB: 30.4 K+ binders: 66.1 Diuretics: 23.2 | NR | Stage 2-3 100 | Mean (SD): 53 (11) | Mean (SD): 4.8 (1.3) | NR | Mean (SD): 4.3 (0.3) |
| Tseng 2017 ([126](#_ENREF_126)) | Pre-dialysis stage 5 CKD patients | NR | MRA (spironolactone) | 1,363 | ACEi: 32.1 ARBs: 41.4 β-blockers: 52 Thiazide-loop diuretics: Thiazide: 18.3 Loop diuretics: 91.7 Miscellaneous: 20 CCB: Non-DHP: 15.7 DHP: 72.9 K+ binders: 32.1 Antidiabetic medication Sulfonylurea: 26.3 Meglitinide: 20.5 α-glucosidase inhibitor: 10.1 Biguanide: 7.9 Thiazolidinedione: 7.5 Insulin: 46.4 Statin: 22.7  Aspirin: 31.5 Acetaminophen: 34.9 NSAIDs Selective: 6.9 Non-selective: 53.3 K+ lowering agent: 23.9 NaHCO3: 26.4 | Prior HF  - None: 71.3  - Mild: 19.4  - Severe: 9.2 DM: 71.2 Stroke: 25.3 AFib: 4.6 Cirrhosis: 17.8 PAOD: 2.5 Cancer: 14.4 CAD: 35.3 | Stage 5: 100 | NR | NR | NR | NR |
|  |  |  | No spironolactone | 25,850 | ACEi: 21.2 ARBs: 37.7 β-blockers: 44 Thiazide diuretics: 8.8 Loop diuretics: 58 Miscellaneous: 11.3 CCB: Non-DHP: 11.6 DHP: 75.7 K+ binders: 21.2 Antidiabetic medication Sulfonylurea: 16.2 Meglitinide: 12.6 α-glucosidase inhibitor: 5.1 Biguanide: 3.3 Thiazolidinedione: 4.1 Insulin: 23.6 Statin: 17.6 Aspirin: 20.1 Acetaminophen: 52.3 NSAIDs Selective: 4.4 Non-selective: 34.5 K+ lowering agent: 18.2 NaHCO3: 15.4 | Prior HF  - None: 87  - Mild: 10.1  - Severe: 2.9 DM: 52.7 Stroke: 18 AFib: 2 Cirrhosis: 7.3 PAOD: 1.2 Cancer: 8.9 CAD: 25.2 | Stage 5: 100 | NR | NR | NR | NR |
| EVALUATE  Ando 2014 ([18](#_ENREF_18)) | Patients with stage 1-3 non-diabetic CKD + HT | sK+ >5.5 mmol/L | MRA (eplerenone) | 162 | ACEi: 12 ARBs: 91 β-blockers: 12 Thiazide diuretics: 14 Loop diuretics: 1  CCB: 62 K+ binders: 12 α-blocker: 6 Lipid-lowering drugs:  Statins: 32 Fibrates: 5 Aspirin: 6 Others: 1 | HT: 100 HF/ Arrythmia: 4 Other atherosclerotic diseases: 1 | Stage 1: 8 Stage 2: 54 Stage 3a: 37 Stage 3b: 1 | Mean (SD): 67.7 (14.3) | Mean (SD): 3.0 (0.7) | NR | Mean (SD): 4.2 (0.4) |
|  |  |  | Placebo | 152 | ACEi: 12 ARBs: 89 β-blockers: 14 Thiazide diuretics: 11 Loop diuretics: <1 CCB: 62 K+ binders: 12 α-blocker: 5 Lipid-lowering drugs Statins: 32 Fibrates: 1 Aspirin: 7 Others: <1 | HT: 100 HF/ Arrythmia: 5 Other atherosclerotic diseases: 1 | Stage 1: 9 Stage 2: 58 Stage 3a: 30 Stage 3b: 3 | Mean (SD): 68.6 (13.6) | Mean (SD): 2.9 (0.6) | NR | Mean (SD): 4.15 (0.4) |
| Maddirala 2008 ([100](#_ENREF_100)) | Patients with stage 1 to 5 CKD | sK+ ≥5.5 mEq/L | ACEi/ARB + other combinations | 931 | NR | NR | Stage 1: 9.2 Stage 2: 50.4 Stage 3: 34.2 Stage 4: 5.5 Stage 5: 0.7 | Mean (SD): 64.6 (21.3) | NR | NR | Mean (SD): 4.1 (0.4) |
| Rysava 2005 ([127](#_ENREF_127)) | Patients with CKD + HT | NR | ARB (telmisartan) | 92 | ACEi: 73.9 β-blockers: 45.7 CCB: 65.2 K+ binders: 73.9 Diuretics: 42.4 Centrally acting imidazoline-1 receptor agonists: 26.1 Antihypertensive drugs: 33.7 Three to five additional drugs to manage HT: 45.6 | HT: 100 DM: 65.2 Diabetic nephropathy: 45.6 Tubulointerstitial nephritis: 9.78 Chronic glomerulonephritis: 15.2 ADPKD: 2.17 Vascular nephrosclerosis: 7.6 Congenital kidney disease: 2.17 | NR | NR | NR | NR | NR |
| FIGARO-DKD Pitt 2021 ([9](#_ENREF_9)) | Patients with stage 2-4 CKD with moderately elevated albuminuria or stage 1 or 2 CKD with severely elevated albuminuria + DM | NR | ARB (finerenone) | 3,686 | ACEi: 42.8 ARBs: 57.2 DRIs: 99.9 β-blockers: 48.1 Thiazide diuretics: 47.4 K+ binders: 42.8 Statin: 69.2 Loop diuretics: 16.2 Thiazide diuretics: 24.7 Platelet aggregation inhibitors: 55.5 K+ binding agents: 0.7 K+ supplements:3 α-blockers: 18.2 Calcium antagonists: 51.3 Glucose-lowering therapy: 97.9 Insulin: 54.9 GLP-1 receptor agonist: 8.4 SGLT2i: 8.5 Metformin: 69.5 Sulfonylurea: 28.1 α-glucosidase inhibitor: 4.3 DPP-4 inhibitors: 24.3 | DM: 100 HT: 96.1 HF: 7.9 CVD: 45.5 Diabetic retinopathy: 32.4 Diabetic neuropathy: 28.4 History of CVD: 45.5 CAD: 31.1 MI: 17.4 PAD: 15.9 Ischemic stroke: 12 | NR | Mean (SD): 67.6 (21.7) | NR | NR | Mean (SD): 4.3 (0.4) |
|  |  |  | Placebo | 3,666 | ACEi: 42.6 ARBs: 57.4 DRIs: 99.9 β-blockers: 48.1 Thiazide diuretics: 47.7 K+ binders: 42.6 Statin: 71.8 Loop diuretics: 16 Thiazide diuretics: 24.2 Platelet aggregation inhibitors: 55.3 K+ binding agents: 0.6 K+ supplements: 2.8 α-blockers: 18.9 Calcium antagonists: 51.3 Glucose-lowering therapy: 97.9 Insulin: 53.7 GLP-1 receptor agonist: 6.6 SGLT2i: 8.3 Metformin: 68.4 Sulfonylurea: 28 α-glucosidase inhibitor: 4.7 DPP-4 inhibitors: 23.5 | HT: 95.9 HF: 7.7 CVD: 45.1 Diabetic retinopathy: 30 Diabetic neuropathy: 27 History of CVD: 45.1 CAD: 31.3 MI: 16.8 PAD: 15.7 Ischemic stroke: 11.6 | NR | Mean (SD): 68 (21.7) | NR | NR | Mean (SD): 4.3 (0.4) |
| FIDELIO-DKD Bakris 2020 ([10](#_ENREF_10)) **Linked studies:** Agarwal 2022 ([128](#_ENREF_128)) Zhang 2022b ([129](#_ENREF_129))  Filippatos 2021 ([130](#_ENREF_130)) Rossing 2022a ([131](#_ENREF_131))  Rossing 2022b ([132](#_ENREF_132))  Zhang 2023 ([133](#_ENREF_133)) | Patients with advanced CKD + DM | sK+ >5.5 mmol/L | ARB (finerenone) | 2,833 | ACEi: 33.5 ARBs: 66.3 β-blockers: 51.6 Thiazide diuretics: 55.7 K+ binders: 33.5 Statin: 74.3 K+ lowering agent: 2.5 α-blockers: 24.5 Calcium antagonists: 62.6 Loop diuretics: 27.7 Thiazide diuretics: 24.7 Platelet aggregation inhibitors: 57.6 Glucose-lowering therapies: 97 Insulin: 65.1 GLP-1 receptor agonist: 6.7 SGLT2i: 4.4 Metformin: 44.2 Sulfonylurea: 23.1 α-glucosidase inhibitor: 5.8 DPP4 inhibitors: 27 | HT: 96.6 HF: 6.9 Diabetic retinopathy: 46.3 Diabetic neuropathy: 26.1 History of CVD: 46 CAD: 29.7 MI: 13.3 PAD: 6.6 Ischemic stroke: 11.6 | NR | Mean (SD): 44.4 (12.5) | NR | NR | Mean (SD): 4.4 (0.5) |
|  |  |  | Placebo | 2,841 | ACEi: 34.9 ARBs: 65 β-blockers: 53 Thiazide diuretics: 57.6 K+ binders: 34.9 Statin: 74.3 K+ lowering agent: 2.3 α-blockers: 25.2 Calcium antagonists: 63.8 Loop diuretics: 29.3 Thiazide diuretics: 23.1 Platelet aggregation inhibitors: 56.1 Glucose-lowering therapies: 97.7 Insulin: 63.1 GLP-1 receptor agonist: 7.2 SGLT2i: 4.8 Metformin: 43.6 Sulfonylurea: 23.7 α-glucosidase inhibitor: 5.7 DPP4 inhibitors: 26.7 | HT: 97.4 HF: 8.5 Diabetic retinopathy: 47.6 Diabetic neuropathy: 25.2 History of CVD: 45.8 CAD: 30.3 MI: 13.7 PAD: 15.9 Ischemic stroke: 12.7 | NR | Mean (SD): 44.3 (12.6) | NR | NR | Mean (SD): 4.4 (0.5) |
| Fang 2018 ([119](#_ENREF_119)) | Patients with CKD hospitalised for MI | Primary or secondary diagnosis code of 276.7 in inpatient claims | ACEi/ARB | 20,620 | ACEi: 71.4 ARBs: 28.6 K+ binders: 71.4 | HT: 88.4 HF: 42.8 DM: 54.9 MI: 9.5 Cancer: 11.7 CBD: 21.2 COPD: 28.3 Dementia: 6.4 AIDS/HIV: 0.1 Metastatic carcinoma: 1.4 Liver disease: 2.1 Paralysis: 1.4 Peptic ulcer disease: 2 PVD: 26.9 Connective tissue disease-rheumatic disease: 3.9 | NR | NR | NR | NR | NR |
| Silvarino 2019 ([106](#_ENREF_106)) | Patients with stage 1-5 non-dialysis dependent CKD | sK+ ≥5.6 mEq/L | ACEi | 221 | β-blockers: 13.1 Diuretics: 19.4 CCB: 17.1 α-blockers: 0.9 | DM: 36 CV comorbidities ≥1: 21 | CKD stage 1 to 2 CKD Stage 3 to 5: CKD stage 1 to 2: 29.0 CKD Stage 3 to 5: 71.0 | Mean (SD): 55.3 (26.1) | NR | NR | NR |
|  |  |  | ARB | 196 | β-blockers: 24.5 Diuretics: 34.7 CCB: 17 α-blockers: 2 | DM: 36 CV comorbidities ≥1: 38 | CKD stage 1 to 2: 20.0 CKD Stage 3 to 5: 80.0 | Mean (SD): 47.5 (19.9) | NR | NR | NR |
|  |  |  | No ACEi/ARB | 703 | β-blockers: 26.7 Diuretics: 36.4 CCB: 36.1 α-blockers: 2.8 | DM: 21 CV comorbidities ≥1: 30 | CKD stage 1 to 2: 9.0 CKD Stage 3 to 5: 91.0 | Mean (SD): 36.3 (19.4) | NR | NR | NR |
| Linde 2019 ([47](#_ENREF_47)) | Patients with CKD | sK+ >5.0 mmol/L | RAASi <50% ESC recommended dose | 27,935 | β-blockers: 31.3 Diuretics: 46.2 CCB: 27.8 Statins: 55.7 Bronchodilators: 12.1 NSAIDs: 8.7 Oral antidiabetics: 11.6 Insulin: 2.9 | DM: 15.8 MI: 5.3 PVD: 2.7 Stroke: 6.5 Arrhythmia: 9.5 Cardiopulmonary disease: 10.1 Metastatic tumor: 2.1 Rheumatic disease: 3.4 Peptic ulcer: 0.9 Cancer: 9 | NR | Mean (SD): 51.2 (10.5) | NR | NR | Mean (SD): 4.5 (0.5) |
|  |  |  | RAASi ≥50% ESC recommended dose | 26,596 | β-blockers: 33.3 Diuretics: 52.6 CCB: 39.7 Statins: 59.2 Bronchodilators: 9.8 NSAIDs: 9.5 Oral antidiabetics: 15.7 Insulin: 4.1 | DM: 22.0 MI: 4.1 PVD: 2.9 Stroke: 5.4 Arrhythmia: 7.6 Cardiopulmonary disease: 9.5 Metastatic tumor: 1.91 Rheumatic disease: 2.8 Peptic ulcer: 0.8 Cancer: 7.8 | NR | Mean (SD): 52.0 (8.6) | NR | NR | Mean (SD): 4.5 (0.5) |
| IsK+andar 2012 ([62](#_ENREF_62)) | Patients with ESRD under haemodialysis | sK+ >5.3 mEq/L | ACEi (captopril/lisinopril) or ARB (valsartan) | 44 | NR | NR | NR | NR | NR | NR | NR |
| Kashihara 2019 ([88](#_ENREF_88)) | Patients with stage 1-5 CKD | sK+ ≥5.1 mEq/L | RAASi (ACEi/ARB/MRA) | NR | NR | NR | Stage 1-5: 100 | NR | NR | NR | NR |
| Saito 2017 ([101](#_ENREF_101)) | Patients with stage 3-5 non-dialysis dependent CKD | sK+ ≥5.5 mEq/L | RAASi (ACEi/ARBs/MRAs/DRI) | 986 | DRIs: 1.3 Any RAASi: 17.8 β-blockers: 47.1 Thiazide-loop diuretics: 41.1 K+ binders: 2.0 ACE inhibitor/ARB: 90.2 Aldosterone antagonist: 25.4 NSAIDs: 6.0 | DM: 50.8 Malignancy: 11.1 | Stage 3a: 61.3 Stage 3b: 26.9 CKD stage 4 to 5: 24.0 | Mean (SD): 45.4 (11.7) | NR | NR | Mean (SD): 4.3 (0.5) |
| ARTS-HF Pitt 2013 ([26](#_ENREF_26)) | Patients with mild (stage 2) CKD (eGFR 60 to 90 mL/min/1.73 m2) + HFpEF (Part A) | NR | MRA (finerenone – 2.5 mg qd) | 16 | Any RAASi: 100 β-blockers: 100 Diuretics: 68.8 | HF:100 HT: 31.3 DM: 25.0 IHD: 18.8 AFib: 50.0 | Stage 2: 100 | Mean (SD): 69.3 (8.2) | Median (range): 1.0 (0.8 to 1.2) | NR | Mean (SD): 4.2 (0.4) |
|  |  |  | MRA (finerenone- 5 mg qd) | 16 | Any RAASi: 100 β-blockers: 100 Diuretics: 68.8 | HF:100 HT: 62.5 DM: 12.5 IHD: 37.5 AFib: 37.5 | Stage 2: 100 | Mean (SD): 69.7 (9.3) | Median (range): 1.0 (0.8 to 1.3) | NR | Mean (SD): 4.3 (0.4) |
|  |  |  | MRA (finerenone – 10 mg qd) | 17 | Any RAASi: 100 β-blockers: 88.2 Diuretics: 76.5 | HF:100 HT: 35.3 DM: 5.9 IHD: 52.9 AFib: 41.2 | Stage 2: 100 | Mean (SD): 67.8 (8.5) | Median (range): 1.0 (0.7 to 1.3) | NR | Mean (SD): 4.3 (0.3) |
|  |  |  | Placebo | 16 | Any RAASi: 93.8 β-blockers: 100 Diuretics: 68.8 | HF:100 HT: 43.8 DM: 12.5 IHD: 37.5 AFib: 18.8 | Stage 2: 100 | Mean (SD): 69.6 (8.3) | Median (range): 1.0 (0.8 to 1.3) | NR | Mean (SD): 4.2 (0.3) |
|  | Patients with moderate (stage 3) CKD (eGFR 30 to 60 mL/min/1.73 m2) + HFpEF (Part B) |  | MRA (finerenone – 2.5 mg qd) | 66 | Any RAASi: 97 β-blockers: 98.5 Diuretics: 95.5 | HF:100 HT: 62.1 DM: 30.3 IHD: 54.5 AFib: 53 | Stage 3: 100 | Mean (SD): 47.9 (8.7) | Median (range): 1.5 (0.8 to 2.3) | NR | Mean (SD): 4.4 (0.5) |
|  |  |  | MRA (finerenone – 5 mg qd) | 67 | Any RAASi: 91 β-blockers: 89.6 Diuretics: 85.1 | HF:100 HT: 71.6 DM: 31.3 IHD: 67.2 AFib: 44.8 | Stage 3:100 | Mean (SD): 45.9 (8.6) | Median (range): 1.5 (0.9 to 2.9) | NR | Mean (SD): 4.3 (0.4) |
|  |  |  | MRA (finerenone -10 mg qd) | 67 | Any RAASi: 97 β-blockers: 86.6 Diuretics: 92.5 | HF:100 HT: 79.1 DM: 37.3 IHD: 62.7 AFib: 43.3 | Stage 3:100 | Mean (SD): 47 (10.9) | Median (range): 1.4 (0.9 to 3.1) | NR | Mean (SD): 4.3 (0.4) |
|  |  |  | MRA (finerenone – 5 mg bid) | 64 | Any RAASi: 93.8 β-blockers: 93.8 Diuretics: 81.3 | HF:100 HT: 62.5 DM: 28.1 IHD: 65.6 AFib: 37.5 | Stage 3:100 | Mean (SD): 47.7 (12.9) | Median (range): 1.4 (0.8 to 2.3) | NR | Mean (SD): 4.4 (0.4) |
|  |  |  | MRA (spironolactone – 25 or 50 mg qd) | 63 | Any RAASi: 95.2 β-blockers: 96.8 Diuretics: 92.1 | HF:100 HT: 57.1 DM: 38.1 IHD: 57.1 AFib: 47.6 | Stage 3:100 | Mean (SD): 46.8 (10.3) | Median (range): 1.4 (0.9 to 2.4) | NR | Mean (SD): 4.2 (0.5) |
|  |  |  | Placebo | 65 | Any RAASi: 95.4 β-blockers: 95.4 Diuretics: 87.7 | HF:100 HT: 66.2 DM: 40 IHD: 76.9 AFib: 44.6 | Stage 3:100 | Mean (SD): 46.9 (8.2) | Median (range): 1.4 (1.0 to 2.2) | NR | Mean (SD): 4.3 (0.4) |
| AVOID Persson 2010 ([27](#_ENREF_27)) | Patients with stage 1 CKD + HT + DM | sK+ >5.5 mmol/L sK+ >6.0 mmol/L | DRI (aliskiren) | 64 | NR | HT: 100 DM: 100 CVD: 12.5 | Stage 1: 100 | Median (range): 102.5 (90.4 to 171.2) | Mean (SD): 2.7 (0.4) | NR | Mean (SD): 4.4 (0.46) |
|  |  |  | Placebo | 51 | NR | HT: 100 DM: 100 CVD: 13.7 | Stage 1: 100 | Median (range): 100.4 (90.7 to 155.8) | Mean (SD): 2.6 (0.4) | NR | Mean (SD): 4.4 (0.38) |
|  | Patients with stage 2 CKD + HT + DM |  | DRI (aliskiren) | 104 | NR | HT: 100 DM: 100 CVD: 19.2 | Stage 2: 100 | Median (range): 73.6 (60.8 to 89.9) | Mean (SD): 3.5 (0.5) | NR | Mean (SD): 4.5 (0.49) |
|  |  |  | Placebo | 122 | NR | HT: 100 DM: 100 CVD: 19.7 | Stage 2: 100 | Median (range): 72.4 (60.0 to 89.7) | Mean (SD): 3.6 (0.5) | NR | Mean (SD): 4.5 (0.48) |
|  | Patients with stage 3 CKD + HT + DM |  | DRI (aliskiren) | 129 | NR | HT: 100 DM: 100 CVD: 24.8 | Stage: 100 | Median (range): 47.1 (22.8 to 59.9) | Mean (SD): 5.5 (1.4) | NR | Mean (SD): 4.6 (0.54) |
|  |  |  | Placebo | 119 | NR | HT: 100 DM: 100 CVD: 21.8 | Stage: 100 | Median (range): 44.7 (24.6 to 59.7) | Mean (SD): 5.7 (1.3) | NR | Mean (SD): 4.5 (0.51) |
| Gwoo 2014 ([81](#_ENREF_81)) | Patients with stage 2-4 CKD + HT | sK+ ≥5.0 mEq/L | MRA (spironolactone) | 891 | ACEi: 17.6 ARBs: 9.5 Diuretics: 54.1 K+ binders: 17.6 Polystyrene sulfonate calcium: 34.5 | HT: 100 | Stage 2: 2.5 Stage 3: 90.2 Stage 4: 7.3 | Mean (SD): 47.1 (11.7) | Mean (SD): 1.5 (0.9) | NR | Mean (SD): 4.2 (0.6) |
| Vukusich 2010 ([19](#_ENREF_19)) | Patients with ESRD under haemodialysis | NR | MRA (spironolactone) | 30 | Thiazide-loop diuretics: 3.3 CCB: 36.7 Dialysis: 100 Digitalis: 3.3 Aspirin: 33.3 Statins: 3.3 Nitrates: 3.3 Calcium: 66.7 Amiodarone: 6.7 Erythropoietin: 20.0 | NR | NR | NR | NR | NR | Mean (SD): 4.7 (0.9) |
|  |  |  | Placebo | 23 | Thiazide-loop diuretics: 4.3 CCB: 43.5 Dialysis: 100 Digitalis: 0 Aspirin: 39.0 Statins: 4.3 Nitrates: 4.3 Calcium: 87.0 Amiodarone: 0 Erythropoietin: 21.7 | NR | NR | NR | NR | NR | Mean (SD): 4.7 (0.7) |
| PHASE Walsh 2015 ([20](#_ENREF_20)) | Patients with ESRD under haemodialysis | sK+ >6.0 mEq/L sK+ >6.5 mEq/L sK+ >7 mEq/L | MRA (eplerenone) | 77 | Any RAASi: 42.9 β-blockers: 50.6 Dialysis: 100 | HF: 10.4 DM: 51.9 CAD: 20.8 Previous stroke: 5.2 PVD: 6.5 | NR | NR | NR | NR | Mean (SD): 4.7 (0.6) |
|  |  |  | Placebo | 77 | Any RAASi: 41.6 β-blockers: 50.6 Dialysis: 100 | HF: 7.8 DM: 51.9 CAD: 16.9 Previous stroke: 5.2 PVD: 11.7 | NR | NR | NR | NR | Mean (SD): 4.9 (0.6) |
| An 2021 ([102](#_ENREF_102)) | Patients with stage 2-4 diabetic kidney disease + HT | ICD-9 diagnostic code of 276.7  ICD-10 diagnostic code of E87.5  pK+/sK+ ≥5.5 mEq/L | Combination therapy (MRA + ACEI/ARB) | 1,282 | NR | HT: 100 HF: 8.1 DM: 100 | Stage 2: 42.4 Stage 3a: 25.4 Stage 3b: 22.9 Stage 4: 9.3 | NR | NR | NR | NR |
|  |  |  | Monotherapy (ACEI/ARB only) | 5,484 | NR | HT: 100 HF: 3.4 DM: 100 | Stage 2: 49.1 Stage 3a: 25 Stage 3b: 17.9 Stage 4: 8 | NR | NR | NR | NR |
| Qu 2021 ([71](#_ENREF_71)) | Patients with stage 3-5 CKD | sK+ >5.5 mmol/L | MRA (spironolactone) | 200 | Any RAASi: 54 β-blockers: 59.5 Statins: 90.5 | HT: 69 DM: 30.5 CAD: 8.0 Stroke: 10.0 | Stage 3-5: 100 | Mean (SD): 41.5 (13.3) | NR | NR | Mean (SD): 4.2 (0.6) |
|  |  |  | Standard therapy | 360 | Any RAASi: 31.7 β-blockers: 41.1 Statins: 76.1 | HT: 68.3 DM: 30.3 CAD: 8.3 Stroke: 9.4 | Stage 3-5: 100 | Mean (SD): 35.6 (16.6) | NR | NR | Mean (SD): 4.4 (0.8) |
| Edwards 2021 ([1](#_ENREF_1)) | Patients with stage 2-3 non-diabetic CKD | sK+ >5.0 mEq/L | MRA (spironolactone) | 77 | ACEi: 55 ARBs: 45 β-blockers: 17 CCB: 43 K+ binders: 55 α-blockers: 17 Statins: 40 | HT: 87 MI: 1 Stroke: 1 | Stage 2-3: 100 | Mean (SD): 52 (16) | Mean (SD): 1.5 (0.5) | NR | Mean (SD): 4.4 (0.4) |
|  |  |  | Diuretic (chlorthalidone) | 77 | ACEi: 56 ARBs: 44 β-blockers: 17 CCB: 30 K+ binders: 56 α-blockers: 8 Statins: 45 | HT: 83 MI: 5 Stroke: 1 | Stage 2-3: 100 | Mean (SD): 57 (15) | Mean (SD): 1.3 (0.4) | NR | Mean (SD): 4.5 (0.3) |
| TRANSITION Straburzynska-Migaj 2021 ([2](#_ENREF_2)) | Patients with stage 3 CKD + HFpEF | NR | ARNi (sacubitril/valsartan) | 476 | NR | HF: 100 | Stage 3:100 | NR | NR | NR | NR |
| Ruilope 2000 ([3](#_ENREF_3)) | Patients with progressive CKD with or without proteinuria + HT | sK+ ≥ 6.0 mmol/L | ARB (valsartan – 160 mg) | 22 | NR | HT: 100 Glomerulonephritis IgA: 5 Other glomerulonephritis: 23 Nephrosclerosis: 27 Other: 45 | NR | NR | NR | NR | NR |
|  |  |  | ARB (valsartan – 80 mg) + ACEi (benazepril – 5 or 10 mg) | 42 | NR | HT: 100 Glomerulonephritis IgA: 19 Other glomerulonephritis: 23 Nephrosclerosis: 19 Other: 40 | NR | NR | NR | NR | NR |
|  |  |  | ARB (valsartan – 160 mg) + ACEi (benazepril – 5 or 10 mg) | 44 | NR | HT: 100 Glomerulonephritis IgA: 9 Other glomerulonephritis: 25 Nephrosclerosis: 39 Other: 28 | NR | NR | NR | NR | NR |
| De Rosa 2001 ([77](#_ENREF_77)) | Patients with chronic renal insufficiency + HT | sK+ >6.0 mEq/L | ARB (irbesartan) | 52 | Diuretics: 6.6 CCB: 3.4 | HT: 100 DM: 23 | NR | NR | NR | NR | NR |
| MiREnDa Hammer 2019 ([4](#_ENREF_4)) | Patients with ESRD under haemodialysis | sK+ 6.0 to 6.5 mmol/L  sK+ ≥6.5 mmol/L | MRA (spironolactone) | 50 | Any RAASi: 58 β-blockers: 68 Thiazide-loop diuretics: 70 CCB: 44 Dialysis: 100 K+ binders: 4.0 Statins: 16 Antiplatelets: 64  Phosphate binder: 92 Erythropoietin-stimulating agent: 64 Antihypertensive drugs: 70 | HT: 78 HF: 6 DM: 32 CAD: 38 AFib: 10 LVH: 50 COPD: 8 PVD: 20 | NR | NR | NR | NR | NR |
|  |  |  | Placebo | 47 | Any RAASi: 55.3 β-blockers: 72.3 Thiazide-loop diuretics: 57.5 CCB: 53.2 Dialysis: 100 K+ binders: 2.1 Statins: 29.8 Antiplatelets: 55.3  Phosphate binder: 93.6  Erythropoietin-stimulating agent: 76.6 Antihypertensive drugs: 80.9 | HT: 97.9 HF: 2 DM: 34 CAD: 36.2 AFib: 14.9 LVH: 40.4 COPD: 6.4 PVD: 21.3 | NR | NR | NR | NR | NR |
| Spin-D Charytan 2019 ([5](#_ENREF_5)) | Patients with ESRD under haemodialysis | sK+ >6.5 mEq/L | MRA (spironolactone – 12.5 mg per day) | 27 | Any RAASi: 33.3 β-blockers: 44.4 Dialysis, <1 year: 14.8 Dialysis, ≥1 year: 85.2 Statins: 40.7 Antiplatelets: 33.3 | HT: 88.9 HF: 7.4 DM: 48.1 CAD: 3.7 AFib: 3.7 Stroke: 14.8 PAD: 7.4 Hyperlipidaemia: 33.3 | NR | NR | NR | Mean (SD): 3.5 (0.8) | Mean (SD): 4.7 (0.5) |
|  |  |  | MRA (spironolactone – 25 mg per day) | 26 | Any RAASi: 30.8 β-blockers: 46.2 Dialysis, <1 year: 11.5 Dialysis, ≥1 year: 88.5 Statins: 50.0 Antiplatelets: 38.5 | HT: 88.5 HF: 15.4 DM: 53.8 CAD: 30.8 AFib: 7.7 Stroke: 3.8 PAD: 15.4 Hyperlipidaemia: 38.5 | NR | NR | NR | Mean (SD): 3.4 (0.8) | Mean (SD): 4.8 (0.6) |
|  |  |  | MRA (spironolactone – 50 mg per day) | 25 | Any RAASi: 28 β-blockers: 60 Dialysis <1 year: 8.0 Dialysis ≥1 year: 92.0 Statins: 36.0 Antiplatelets: 32.0 | HT: 92 HF: 20 DM: 60 CAD: 28.0 AFib: 0 Stroke: 12.0 PAD: 16.0 Hyperlipidaemia: 40.0 | NR | NR | NR | Mean (SD): 3.6 (0.9) | Mean (SD): 4.8 (0.7) |
|  |  |  | Placebo | 51 | Any RAASi: 29.4 β-blockers: 43.1 Dialysis <1 year: 7.8 Dialysis ≥1 year: 92.2 Statins: 41.2 Antiplatelets: 33.3 | HT: 98 HF: 19.6 DM: 47.1 CAD: 23.5 AFib: 13.7 Stroke: 27.5 PAD: 11.8 Hyperlipidaemia: 39.2 | NR | NR | NR | Mean (SD): 3.6 (0.8) | Mean (SD): 4.8 (0.6) |
| UK HARP-III  Haynes 2018 ([6](#_ENREF_6)) | Patients with stage A1-A3 CKD with eGFR 20 to 60 mL/min/1.73 m2 | sK+ ≥ 5.5 mmol/L | ARNi (sacubitril/valsartan) | 207 | Any RAASi: 84 β-blockers: 24 Diuretics: 38 CCB: 50 Antiplatelet therapy: 31 Oral anticoagulant: 6 α-Blocker: 28 LDL-lowering agent: 61 | HF: 4 DM: 39 CAD: 10 CBD: 8 PVD: 11 | A1: 14.5  A2: 20.8  A3: 64.7 | Mean (SD): 35.4 (1) | NR | NR | NR |
|  |  |  | ARB (irbesartan) | 207 | Any RAASi: 80 β-blockers: 30 Diuretics: 41 CCB: 50 Antiplatelet therapy: 36 Oral anticoagulant: 7 α-Blocker: 27 LDL-lowering agent: 66 | HF: 3 DM: 40 CAD: 16 CBD: 7 PVD: 11 | A1: 13.5  A2: 21.7  A3: 64.7 | Mean (SD): 35.5 (1) | NR | NR | NR |
| Lee 2014 ([103](#_ENREF_103)) | Patients with stage 3 or 4 CKD with hyperkalaemia | sK+ ≥ 5.5 mmol/L | Maintenance group (ACEi or ARB without discontinuation) | 150 | NR | HT: 24 DM: 48 Primary renal disease: 43.3 Diabetic nephropathy: 16.0 Chronic glomerulonephritis: 16.7 | Stage 3-4: 100 | Mean (SD): 28.3 (13.2) | Mean (SD): 2.4 (1.2) | Mean (SD): 3.8 (1.1) | Mean (SD): 5.8 (0.4) |
|  |  |  | Withdrawal group (ACEi or ARB discontinuation for >3 months after hyperkalaemia) | 108 | NR | HT: 22.2 DM: 45.4 Primary renal disease: 42.6 Diabetic nephropathy: 13.0 Chronic glomerulonephritis: 18.5 | Stage 3-4: 100 | Mean (SD): 30.1 (12.9) | Mean (SD): 2.3 (1.2) | Mean (SD): 3.7 (0.7) | Mean (SD): 5.8 (0.5) |
| Agrawal 2016 ([134](#_ENREF_134)) | Patients with CKD | NR | ARB (telmisartan) | 55 | NR | HT: 92.7 DM: 63.6 DM + HT: 60 | NR | Mean (SD): 52.1 (17.6) | Mean (SD): 1.9 (0.7) | NR | NR |
| Woo 2013 ([50](#_ENREF_50)) | Patients with stage 1-3 non-diabetic CKD | sK+ >5 mmol/L  sK+ >6.0 mmol/L | DRI (aliskiren) + ARB (losartan) | 49 | NR | HT: 32.7 | Stage 1: 2.0 Stage 2: 16.3 Stage 3: 81.6 | Mean (SD): 49 (19) | Mean (SD): 1.6 (0.4) | NR | NR |
|  |  |  | DRI (aliskiren) | 46 | NR | HT: 32.6 | Stage 2: 10.9 Stage 3: 89.1 | Mean (SD): 48 (14) | Mean (SD): 1.6 (0.5) | NR | NR |
|  |  |  | ARB (losartan – high dose) | 48 | NR | HT: 35.4 | Stage 2: 8.3 Stage 3: 91.7 | Mean (SD): 48 (14) | Mean (SD): 1.6 (0.4) | NR | NR |
| Yildirim 2012 ([135](#_ENREF_135)) | Patients with stage 3-5 CKD | NR | RAASi | 279 | NR | HT: 70.6 HF: 4.6 DM: 23.3 CAD: 18.6 Nephrolithiasis: 11.5 Glomerulonephritis: 7.5 Malignancy: 7.3 PKD: 5.0 Pyelonephritis: 4.6 Solitary kidney: 4.3 Chronic lung disease: 2.5 Familial Mediterranean fever: 0.4 | Stage 3-5: 100 | Mean (SD): 33.3 (15.1) | Mean (SD): 2.5 (1.4) | NR | Mean (SD): 4.7 (0.6) |
| Pisoni 2012 ([48](#_ENREF_48)) | Patients with stage 3 CKD + Resistant HT | sK+ >5.0 mEq/L | MRA (spironolactone/eplerenone) | 36 | NR | HT: 100 DM: 31 Obesity: 56 CAD: 25 Hyperlipidaemia: 81 | Stage 3: 100 | Mean (SD): 48.6 (8.7) | Mean (SD): 1.5 (0.3) | NR | Mean (SD): 4 (0.5) |
| Hirai 2018 ([72](#_ENREF_72)) | Patients with stage 3-5 CKD and unknown stage CKD | sK+ >5.5 mEq/L | ACEi/ARB | 2,987 | ACEi: 22.6 ARBs: 75 MRAs: 12.4 DRIs: 0.1 Any RAASi: 2.3 β-blockers: 25.5 Thiazide diuretics: 9.2 Loop diuretics: 21.8 Dialysis: 3.7 K+ binders: 22.6 Trimethoprim-sulfamethoxazole: 1.7 NSAIDs: 8.8 Hypokalaemia diuretics: 28.9 | HF: 12.9 DM: 27.9 | Stage 1: 8.4 Stage 2: 35.6 Stage 3: 40.0 Stage 4: 7.6 Stage 5: 8 CKD stage unknown: 1.0 | Mean (SD): 56.5 (27.4) | Median (range): 0.9 (0.7 to 1.3) | NR | Mean (SD): 4.4 (0.7) |
| Jun 2019 ([78](#_ENREF_78)) | Patients with stage 3-5 CKD | sK+ >6.0 mmol/L or coded or free-text recorded diagnosis of hyperkalaemia | RAASi | 20,184 | ACEi: 30.1 ARBs: 41.3 MRAs: 8.2 ACEi + other combinations: 4.4 ARB + other combinations: 15.8 K+ binders: 30.1 | HF: 26.7 DM: 40.8 AFib: 22.5 CVD: 55.6 LVH: 1.4 Stroke: 13.3 | Stage 3a: 46.8 Stage 3b: 38.3 Stage 4: 13 Stage 5: 1.8 | Mean (SD): 42.1 (11.4) | NR | NR | Mean (SD): 4.6 (0.5) |
| Belmar Vega 2019 ([114](#_ENREF_114)) | Patients with stage 3-5 CKD | sK+ 5.5 to 5.9 mmol/L  sK+ 6.0 to 6.4 mmol/L  sK+ 6.5-6.9 mmol/L  sK+ ≥7.0 mmol/L | RAASi | NR | NR | NR | Stage 3-5: 100 | NR | NR | NR | NR |
| TOPCAT Beldhuis 2019 ([7](#_ENREF_7)) | Patients with stage 2 CKD + HFpEF | sK+ >5.5 mmol/L | MRA (spironolactone) | 413 | Any RAASi: 79.7 Diuretics: 87 Aspirin: 55.6 | HF:100 DM: 38.4 MI: 16.2 Coronary revascularisation: 16.5 Stroke: 7.1 AFib: 38.8  PAD: 8.6 | Stage 2: 100 | Mean (SD): 77.8 (13.1) | NR | NR | Median (IQR): 4.2 (3.9 to 4.4) |
|  |  |  | Placebo | 410 |  |  |  |  |  |  |  |
|  | Patients with stage 3a CKD + HFpEF |  | MRA (spironolactone) | 264 | Any RAASi: 53.1 Diuretics: 89.3 Aspirin: 61.1 | HF:100 DM: 47.6 MI: 24.2 Coronary revascularisation: 22.2 Stroke: 10.9 AFib: 45.5 PAD: 13.3 | Stage 3a: 100 | Mean (SD): 52.4 (4.2) | NR | NR | Median (IQR): 4.3 (3.9 to 4.5) |
|  |  |  | Placebo | 269 |  |  |  |  |  |  |  |
|  | Patients with stage 4 CKD + HFpEF |  | MRA (spironolactone) | 209 | Any RAASi: 59.6 Diuretics: 93.2 Aspirin: 59.6 | HF:100 DM: 53.3 MI: 23.6 Coronary revascularisation: 21.9 Stroke: 10.2 AFib: 44.3  PAD: 15.8 | Stage 4: 100 | Mean (SD): 37.2 (5.1) | NR | NR | Median (IQR): 4.2 (3.9 to 4.6) |
|  |  |  | Placebo | 202 |  |  |  |  |  |  |  |
| Heshka 2010 ([104](#_ENREF_104)) | Patients with stage 3 or 4 CKD + Difficult-to-control HT | sK+ ≥5.5 mmol/L | MRA (spironolactone) | 34 | NR | HT: 100 | Stage 3 or 4: 100 | NR | NR | NR | NR |
| Johnson 2023 ([49](#_ENREF_49)) | Patients with CKD + Hyperkalaemia | sK+ >5.0 mEq/L | RAASi | 4,424 | NR | NR | NR | NR | NR | NR | NR |
| ROTATE-3 Provenzano 2022 ([8](#_ENREF_8)) | Patients with stage 2-4 CKD | sK+ >5 mmol/L | MRA (eplerenone) | 46 | ACEi: 37 ARBs: 63 β-blockers: 37 Thiazide diuretics: 26.1 Loop diuretics: 23.9 CCB: 52.2 K+ binders: 37 Metformin: 47.8 Insulin: 23.9 Statin: 82.6 | DM: 69.6 | Stage 2-4: 100 | Mean (SD): 58.1 (18.6) | NR | NR | NR |
|  |  |  | SGLT2i (dapagliflozin) + MRA (eplerenone) |  |  |  |  |  |  |  |  |
|  |  |  | SGLT2i (dapagliflozin) |  |  |  |  |  |  |  |  |
| Leon 2022 ([105](#_ENREF_105)) | Patients with stage 3-5 CKD survived 90-days after the hyperkalaemia episode (Manitoba cohort) | sK+ ≥5.5 mmol/L | RAASi | 7,200 | MRAs: 10.1 β-blockers: 47.3 K+ binders: 5.6 Antifungal azoles: 0.3 Cyclosporine: 1.3 Digoxin: 7.6 NSAIDs: 5.6 Low-molecular-weight heparin: 0.5 Tacrolimus: 1.0 Trimethoprim: 4.4 | HT: 100 HF: 37.2 DM: 63.1 Stroke/Transient ischemic heart: 14.9 AFib: 28.4 Angina: 13.5 | Stage 3a: 46.1 Stage 3b: 30.6 Stage 4: 18.3 Stage 5: 4.9 | Mean (SD): 40.9 (13.8) | NR | NR |  |
|  | Patients with stage 3-5 CKD survived 90-days after the hyperkalaemia episode (Ontario cohort) |  |  | 71,290 | MRAs: 12.9 β-blockers: 48.5 K+ binders: 1.1 Antifungal azoles: 0.3 Cyclosporine: 0.2 Digoxin: 5.9 NSAIDs: 11.3 LMW heparin: 1.0 Tacrolimus: 0.3 Trimethoprim: 4.5 | HT: 88.9 HF: 21.8 DM: 58 Stroke/Transient ischemic heart: 6.8 AFib: 45.7 Angina: 4.8 | Stage 3a: 43.4 Stage 3b: 35.5 Stage 4: 18.7 Stage 5: 2.4 | Mean (SD): 41.2 (12.6) | NR | NR | Mean (SD): 5.8 (0.4) |
| Salik 2022 ([51](#_ENREF_51)) | Patients with CKD | sK+ >5 mmol/L | ARB | 186 | Thiazide-loop diuretics: 18.3 β blocker/K-sparing diuretic: 18.3 NSAID: 4.3 | HF: 22.6 DM: 38.7 | NR | NR | Median (range): 1.9 (1.7 to 2.3) | NR | Median (IQR): 4.4 (4.1 to 4.8) |
|  |  |  | ACEi | 166 | Thiazide-loop diuretics: 15.1 β blocker/K-sparing diuretic: 18.1 NSAID: 6.0 | HF: 18.7 DM: 22.9 | NR | NR | Median (range): 1.8 (1.7 to 2.1) | NR | Median (IQR): 4.4 (4.1 to 4.7) |
| Tokunaga 2010 ([136](#_ENREF_136)) | Patients with stage 3-4 CKD + HT | NR | ARB (telmisartan) | 36 | β-blockers: 19.4 Thiazide-loop diuretics: 50 CCB: 63.9 K+ binders: 13.9 α-blockers: 13.9 Statins: 27.8 Erythropoietin: 61.1 | HT: 100 Glomerular disease: 19.4 Diabetic nephropathy: 42.7 Others: 16.7 | Stage 3: 16.7 Stage 4: 83.3 | Mean (SD): 21.6 (10.5) | Mean (SD): 3.4 (1.6) | NR | NR |
|  |  | NR | Control | 36 | β-blockers: 25 Thiazide-loop diuretics: 61.1 CCB: 100 K+ binders: 25.0 α-blockers: 16.7 Statins: 19.4 Erythropoietin: 58.3 | HT: 100 Glomerular disease: 22.2 Diabetic nephropathy: 38.9 Others: 16.7 | Stage 3: 13.9 Stage 4: 86.1 | Mean (SD): 21.1 (10.3) | Mean (SD): 3.4 (1.6) | NR | NR |
| Bhandari 2021 ([28](#_ENREF_28)) | Patients with stage 4 or 5 CKD | NR | RAASi discontinuation group | 206 | NR | DM: 36.0 | Stage 4 or 5: 100.0 | Mean (SD): 17.7 (5.4)  Median (range): 18 (6 to 30) | Mean (SD): 3.6 (1.2)  Median (range): 3.4 (1.7 to 9.9) | Mean (SD): 4 (0.5)  Median (range): 4 (2.6 to 5.0) | Mean (SD): 4.9 (0.6)  Median (range): 5 (2.9 to 6.3) |
|  |  |  | RAASi continuation group | 205 | NR | DM: 38.0 | Stage 4 or 5: 100.0 | Mean (SD): 17.9 (5.0)  Median (range): 18 (7 to 30) | Mean (SD): 3.6 (1.1)  Median (range): 3.4 (1.6 to 7.8) | Mean (SD): 4.0 (0.4)  Median (range): 4.0 (2.6 to 5.0) | Mean (SD): 5 (0.6)  Median (range): 5 (3.3 to 6.6) |
| Fu 2021 ([137](#_ENREF_137)) | Patients with advanced CKD (eGFR <30 mL/min per 1.73m2) | NR | RAASi | 10,254 | β-blockers: 67.6  CCB: 60.5  Diuretics: 79.3  Statins: 61.6  Antiplatelets: 46.2  K+ binders: 9.2 | HF: 28.0  HT: 88.7  MI: 21.6  IHD: 33.1  Arrhythmia: 22.4  PVD: 12.4  CBD: 15.8 | Stage 4: 100 | Median (range): 23 (18 to 27) | NR | NR | Mean (SD): 4.5 (0.6) |
| Ahmed 2010 ([138](#_ENREF_138)) | Patients with stage 4 or 5 CKD preparing for RRT | NR | ACEi/ARB | 52 | NR | NR | Stage 4 or 5: 100 | Mean (SD): 16.4 (1.0) | NR | NR | NR |
| Parmar 2024 ([63](#_ENREF_63)) | Patients with CKD + HF + Hyperkalaemia | sK+ >5.4 mmol/L | NA | 35 | NR | HF: 100 DM: 62.9 | NR | Mean (SD): 26.9 (13.5) Median: 26.5 | NR | NR | NR |
|  | Patients with CKD + HF + Normokalaemia (never hyperkalaemia) | NR | NA | 265 | NR | HF: 100 DM: 53.6 | NR | Mean (SD): 28.3 (12.6) Median: 27 | NR | NR | NR |
| Sevamontree 2024 ([107](#_ENREF_107)) | Patients with stage 3-5 CKD + Hyperkalaemia | sK+ ≥5.8 mmol/L | NA | 118 | NR | NR | Stage 3a: 6.8 Stage 3b: 18.6 Stage 4: 22.9 Stage 5: 51.7 | NR | NR | NR | NR |
|  | Patients with stage 3-5 CKD + Normokalaemia | sK+ <5.8 mmol/L | NA | 734 | NR | NR | Stage 3a: 52.3 Stage 3b: 30.9 Stage 4: 8.3 Stage 5: 8.4 | NR | NR | NR | NR |
| NEFRONA Valdivielso 2024 ([52](#_ENREF_52)) | Patients with 3-5 CKD + Hyperkalaemia | sK+ >5.0 mEq/L | NA | **1690** | NR | NR | Stage 3-5: 100 | NR | NR | NR | NR |
|  | Patients with 3-5 CKD + Hypokalaemia | sK+ <3.6 mEq/L | NA | 57 | NR | NR | Stage 3-5: 100 | NR | NR | NR | NR |
| Gulcicek 2023 ([82](#_ENREF_82)) | Patients with stages 1-5 CKD + Hyperkalaemia | sK+ ≥5.0 mmol/L | RAASi | 137 | ACEi: 44.5 ARBs: 55.5 β-blockers: 52.6 Thiazide-loop diuretics: 34.3 CCB: 51.8 α-blockers: 14.6 Allopurinol: 5.8 Furosemide: 13.9 Spironolactone: 2.2 ASA: 37.2 Statin: 29.9 Oral antidiabetics: 47.4 Insulin: 16.8 SGLT2 inhibitor: 27.7 | HT: 98.54 DM: 62.04 CVD: 25.5 CVE: 0.73 | Stage 2: 16.8 Stage 3: 64.2 Stage 4: 16.1 Stage 5: 2.9 | Mean (SD): 45.1 (15.0) | Median (range): 1.3 (0.7-8.2) | Mean (SD): 4.4 (0.3) | Median (range): 5.3 (5.0-6.4) |
|  | Patients with stages 1-5 CKD + Normokalaemia | sK+ <5.0 mmol/L |  | 334 | ACEi: 40.1 ARBs: 59 β-blockers: 41 Thiazide-loop diuretics: 46.7 CCB: 17.7 α-blocker: 1.5 Allopurinol: 4.5 Furosemide: 8.7 Spironolactone: 2.7 ASA: 9.9 Statin: 8.4 Oral antidiabetics: 17.4 Insulin: 5.7 SGLT2 inhibitor: 29.6 | HT: 98.2 DM: 55.39 CVD: 7.5 CBE: 1.2 | Stage 1: 2.7 Stage 2: 7.8 Stage 3: 81.1 Stage 4: 7.8 Stage 5: 0.6 | Mean (SD): 49.2 (16.9) | Median (range): 1.3 (0.6-4.0) | Mean (SD): 4.4 (0.4) | Median (range): 4.6 (2.9-5.0) |
| Zhou 2023a ([53](#_ENREF_53)) | Patients with CKD + HF + Hyperkalaemia | sK+ >5.0 mmol/L | NA | 114 | NR | HF: 100 | NR | NR | NR | NR | NR |
|  | Patients with CKD + HF + Hypokalaemia | sK+ 0 to 3.5 mmol/L | NA | 88 | NR | HF: 100 | NR | NR | NR | NR | NR |
|  | Patients with CKD + HF + Normokalaemia | sK+ >3.5 to 5.0 mmol/L | NA | 564 | NR | HF: 100 | NR | NR | NR | NR | NR |
| Qadir 2023 ([54](#_ENREF_54)) | Patients with stage 1-4 non-dialysis dependent CKD + Hyperkalaemia | sK+ >5.0 mEq/L | NA | 68 | NR | NR | Stage 1-2: 27 Stage 3-4: 69.2 | NR | NR | NR | NR |
|  | Patients with stage 1-4 non-dialysis dependent CKD + Normokalaemia | NR | NA | 82 | NR | NR | Stage 1-2: 73 Stage 3-4: 30.8 | NR | NR | NR | NR |
| EQUAL de Rooij 2023 ([79](#_ENREF_79)) | Patients with stage 4-5 CKD + Hyperkalaemia | sK+ >5.0 to ≤5.5 mmol/L | NA | 294 | Any RAASi: 59 | DM: 47 CVD: 42  Chronic lung disease: 15  Malignancy: 18 | Stage 4-5: 100 | NR | NR | NR | NR |
|  |  | sK+ >5.5 - ≤6.0 mmol/L | NA | 84 | Any RAASi: 59 | DM: 51 CVD: 55  Chronic lung disease: 22  Malignancy: 13 | Stage 4-5: 100 | NR | NR | NR | NR |
|  |  | sK+ >6.0 mmol/L | NA | 31 | Any RAASi: 52 | DM: 40 CVD: 38 Chronic lung disease: 13 Malignancy: 23 | Stage 4-5: 100 | NR | NR | NR | NR |
|  | Patients with stage 4-5 CKD + Hypokalaemia | sK+ ≤3.5 mmol/L | NA | 44 | Any RAASi: 48 | DM: 49 CVD: 33  Chronic lung disease: 26  Malignancy: 21 | Stage 4-5: 100 | NR | NR | NR | NR |
|  | Patients with stage 4-5 CKD + Normokalaemia | sK+ >3.5 to ≤4.0 mmol/L | NA | 228 | Any RAASi: 49 | DM: 37 CVD: 49  Chronic lung disease: 19  Malignancy: 25 | Stage 4-5: 100 | NR | NR | NR | NR |
|  |  | sK+ >4.0 to ≤4.5 mmol/L | NA | 474 | Any RAASi: 50 | DM: 40 CVD: 49  Chronic lung disease: 5  Malignancy: 21 | Stage 4-5: 100 | NR | NR | NR | NR |
|  |  | sK+ >4.5 to ≤5.0 mmol/L | NA | 559 | Any RAASi: 56 | DM: 42 CVD: 48  Chronic lung disease: 14  Malignancy: 23 | Stage 4-5: 100 | NR | NR | NR | NR |
| Perez-Navarro 2023 ([55](#_ENREF_55)) | Patients with stage 1-5 CKD + Hyperkalaemia | sK+ >5.0 mmol/L | NA | **345** | NR | NR | Stage 1: 3 Stage 2: 2 Stage 3a: 4 Stage 3b: 14 Stage 4: 21 Stage 5: 56 | NR | NR | NR | NR |
|  | Patients with stage 1-5 CKD + Normokalaemia | NR |  | **1,016** | NR | NR | Stage 1: 97 Stage 2: 98 Stage 3a: 96 Stage 3b: 86 Stage 4: 79 Stage 5: 44 | NR | NR | NR | NR |
| Wang 2023 ([89](#_ENREF_89)) | Patients with stage 1-4 CKD + Hyperkalaemia | sK+ >5.0 mmol/L | NA | 196 | NR | NR | Stage 1: 3.6 Stage 2: 12 Stage 3: 55.4 Stage 4: 29 | NR | NR | NR | NR |
|  | Patients with stage 1-4 CKD + Normokalaemia | NR |  | 331 |  |  |  |  |  |  |  |
| REVOLUTIONIZE III Bakris 2023 ([84](#_ENREF_84)) | Patients with stage 3-4 CKD + hyperkalaemia | sK+ >5.0 mEq/L | NA | 4,549 | NR | NR | Stage 3-4: 100 | NR | NR | NR | NR |
|  | Patients with stage 3-4 CKD + Normokalaemia | sK+ 3.5 to 5.0 mEq/L |  | 4,549 | NR | NR | Stage 3-4: 100 | NR | NR | NR | NR |
| Gaol 2024 ([139](#_ENREF_139)) | Patients with CKD undergoing hemodialysis + Hyperkalaemia | NR | NA | 161 | ARBs: 55.8 | NR | NR | NR | NR | NR | NR |
|  | Patients with CKD undergoing hemodialysis + Normokalaemia | NR | NA |  | ARBs: 46 | NR | NR | NR | NR | NR | NR |
|  | Patients with CKD undergoing hemodialysis + Hypokalaemia | NR | NA |  | ARBs: 60 | NR | NR | NR | NR | NR | NR |
| Rastogi 2023 ([140](#_ENREF_140)) | Patients with stage 3-4 CKD | NR | RAASi | 7,506 | NR | NR | Stage 3-4: 100 | NR | NR | NR | NR |
|  |  |  |  | 1,179 | NR | NR | Stage 3-4: 100 | NR | NR | NR | NR |
| Mårup 2023 ([73](#_ENREF_73)) | Patients with CKD on maximal tolerated RAASi (ACEi or ARB) with history of at least two hyperkalaemia episodes | pK+ >4.5mmol/L | MRA (spironolactone) | 58 | NR | DM: 39.7 | NR | NR | NR | NR | NR |
| Zhou 2023b ([29](#_ENREF_29)) | Patients with stage 3b-5 CKD + HT | sK+ ≥5.5 mmol/L | ARNi (sacubitril/valsartan) | 44 | β-blockers: 18.2 CCB: 52.3 Diuretic agent: 6.8 α-blocker: 9 | HT: 100 | Stage 3b: 45.5 Stage 4: 45.5 Stage 5: 9.1 | Mean (SD): 29.9 (11.8) | NR | NR | Mean (SD): 4.1 (0.6) |
|  |  |  | Conventional antihypertensive | 40 | β -blockers: 27.5 CCB: 87.5 Diuretic agent: 5 α-blocker: 7.5 | HT: 100 | Stage 3b: 40 Stage 4: 47.5 Stage 5: 13.8 | Mean (SD): 27.9 (10.1) | NR | NR | Mean (SD): 4.2 (0.5) |
| Rajak 2023 ([141](#_ENREF_141)) | Patients with CKD + DM | NR | ARB (finerenone) | NR | NR | DM: 100 | NR | NR | NR | NR | NR |
| Bornstein 2024 ([30](#_ENREF_30)) | Patients with albuminuric CKD + DM | sK+ >5.0 mEq/L | MRA (BI 690517 3 mg) | 18 | β -blockers: 61.1 High-ceiling diuretics: 27.8 Low-ceiling diuretics, thiazides: 27.8 Statins: 88.9 | DM: 100 | NR | Median (range): 41 (22.0–65.0) | NR | NR | Mean (SD): 4.5 (0.4) |
|  |  |  | MRA (BI 690517 10 mg) | 13 | β -blockers: 46.2 High-ceiling diuretics: 30.8 Low-ceiling diuretics, thiazides: 23.1 Statins: 92.3 | DM: 100 | NR | Median (range): 35 (23.0–60.0) | NR | NR | Mean (SD): 4.5 (0.5) |
|  |  |  | MRA (BI 690517 40 mg) | 14 | β -blockers: 57.1 High-ceiling diuretics: 35.7 Low-ceiling diuretics, thiazides: 21.4 Statins: 71.4 | DM: 100 | NR | Median (range): 44 (19.0–70.0) | NR | NR | Mean (SD): 4.4 (0.4) |
|  |  |  | Placebo | 9 | β -blockers: 33.3 High-ceiling diuretics: 11.1 Low-ceiling diuretics, thiazides: 33.3 Statins: 77.8 | DM: 100 | NR | Median (range): 45 (19.0–69.0) | NR | NR | Mean (SD): 4.4 (0.2) |
| Jimenez-Marrero 2024 ([56](#_ENREF_56)) | Patients with CKD | sK+ >5.0 mEq/L | RAASi | 3,868 | NR | NR | NR | NR | NR | NR | NR |
| PROERCAN Garcia-Prieto 2024 ([31](#_ENREF_31)) | Patients with stages 3-4 CKD without proteinuria + HT | sK+ ≥5.5 mmol/L | ACEi | 40 | NR | HT: 100 | Stage 3-4: 100 | Median (range): 42.3 (34.4-47.8) | Median (range): 1.4 (1.2-1.7) | NR | NR |
|  |  |  | Non ACEi | 48 | NR | HT: 100 | Stage 3-4: 100 | Median (range): 37.4 (26.5-40.7) | Median (range): 1.6 (1.4-1.8) | NR | NR |
| An 2023 ([85](#_ENREF_85)) | Patients with CKD | sK+ ≥5.0 mEq/L | RAASi discontinuation | 776 | NR | HT: 90.5 DM: 60.4 MI: 4.9 Stroke: 5.3 Hyperlipidemia: 85.1 Unstable angina: 22.4 Liver disease: 2.6 | NR | Mean (SD): 42.6 (10.7) | NR | NR | Mean (SD): 5.5 (0.5) |
|  |  |  | RAASi continuation | 4952 | NR | HT: 93.5 DM: 59.9 MI: 2.6 Stroke: 2.3 Hyperlipidemia: 87.9 Unstable angina: 19.3 Liver disease: 2.9 | NR | Mean (SD): 44 (10.2) | NR | NR | Mean (SD): 5.3 (0.4) |
| OPTIMIZE I Agiro 2023 ([142](#_ENREF_142)) | Patients with stages 3-5 CKD initiating SZC | NR | RAASi optimisation (same dose or with an up-titration) | 314 | β -blockers: 58.9 Patiromer: 18.5% Sodium polystyrene sulfonate: 15.9% Loop diuretics: 42.7% Thiazides and thiazide-like diuretics: 19.4% NSAIDs: 8.6% | HT: 96.8 HF: 22 DM: 77.4 CAD: 25.8 | Stage 3-5: 100 | NR | NR | NR | NR |
|  | Patients with stages 3-5 CKD initiating SZC | NR | Without RAASi optimisation (discontinued or with a down-titration) | 84 | β -blockers: 8.6 Patiromer: 15 (17.9%) Sodium polystyrene sulfonate: 17 (20.2%) Loop diuretics: 42.7% Thiazides and thiazide-like diuretics: 19.4% NSAIDs: 8.6% | HT: 98.8 HF: 21.4 DM: 81 CAD: 34.5 | NR | NR | NR | NR | NR |
| Gregg 2023 ([143](#_ENREF_143)) | Patients with stage 3-4 CKD | NR | ACEi or ARB: current user | 882,441 | ACEi: 81 ARBs: 19 β -blockers: 50.2 Potassium sparing diuretics: 8.8 Thiazide-loop diuretics: 47.2 CCB: 33.1 Statin: 66.2 SGLT2 inhibitor: 0.3 | HT without complications: 91.2 HT with complications: 2.9 HF: 16.6 DM without complications: 33.4 DM with complications: 19.3 Atherosclerotic CVD: 30.3 CBD: 14.8 PVD: 14.3 Coronary heart disease: 7.4 | Stage 3a: 72.4 Stage 3b: 23.1 Stage 4: 4.5 | NR | NR | NR | NR |
|  |  |  | ACEi or ARB: discontinued user | 326,794 | ACEi: 84.5 ARBs: 15.5 β -blockers: 36.9 K+ sparing diuretics: 7.5 Thiazide-loop diuretics: 31.5 CCB: 25.9 Statin: 45.4 SGLT2 inhibitor: 0.2 | HT without complications: 90.4 HT with complications: 3.1 HF: 16.6 DM without complications: 29.7 DM with complications: 17.5 Atherosclerotic CVD: 31.7 CBD: 15.7 PVD: 15.2 Coronary heart disease: 7.5 | Stage 3a: 70.1 Stage 3b: 23.9 Stage 4: 6.1 | NR | NR | NR | NR |
| Chinnadurai 2023 ([64](#_ENREF_64)) | Patients with non-dialysis dependent CKD | sK+ >5.4 mEq/L | RAASi | 321 | Any RAASi: 100 | HF: 20.9 | NR | Median (range): 24 (18–34) | NR | NR | Median (range): 5.3 (5.2–5.5) |
| Nicholas 2023 ([144](#_ENREF_144)) | Patients with CKD + DM | NR | ARB (Finerenone) | 574 | ACEi: 17 ARBs: 35 MRAs: 2 Any RAASi: 52 ACEi + ARB <1 Insulin: 35 Sodium–glucose cotransporter 2 inhibitor: 35 Glucagon-like peptide-1 receptor agonist: 26 Dipeptidyl peptidase-4 inhibitor: 6 Statin: 24 | DM: 100 | NR | Mean (SD): 53 (23) | NR | NR | NR |
| McFarland 2023 ([57](#_ENREF_57)) | Patients with stage 3-5 CKD + HF | sK+ >5.0 mEq/L | ARNi (Sacubitril/Valsartan) | 50 | ACEi: 22 ARBs: 14 MRAs: 34 β -blockers: 80 Thiazide-loop diuretics: 16 Dialysis: 4 SGLT2i: 4 Hydralazine: 10 Nitrates: 18 Loops: 72 Digoxin: 6 Midodrine: 2 Potassium supplement: 16 Thiazides: 16 | HT: 92 HF: 100 DM: 68 | Stage 3a: 56 Stage 3b: 38 Stage 4: 2 Stage 5: 4 | Median (range): 45.5 (39.8-54.3) | NR | NR | Mean (SD): 4 (0.4) |
| Ding 2023 ([58](#_ENREF_58)) | Patients with ESRD undergoing maintenance dialysis + HT | sK+ >5.0 mmol/L | ARNi (Sacubitril/Valsartan) | 51 | ACEi:  ARBs:  β-blockers: 60.8 Thiazide-loop diuretics: 78.4 CCB: 84.3 Dialysis: 100 α-blocker: 35.3 | HT: 100 DM: 62.7 CAD: 60.8 Heart infarctus: 33.3 | NR | NR | NR | NR | NR |
|  |  |  | Not taking sacubitril/ valsartan | 51 | ACEi:  ARBs:  β-blockers: 52.9 Thiazide-loop diuretics: 62.7 CCB: 58.8 Dialysis: 100 α-blocker: 21.6 | HT: 100 DM: 49 CAD: 43.1 Heart infarctus: 23.5 | NR | NR | NR | NR | NR |
| Jariwala 2023 ([145](#_ENREF_145)) | Patients with CKD + DM | NR | ARB (finerenone) | 42 | NR | DM: 100 | NR | NR | NR | NR | NR |
|  |  |  | No finerenone | 44 | NR | DM: 100 | NR | NR | NR | NR | NR |
| Svensson 2023 ([59](#_ENREF_59)) | Patients with CKD | sK+ >5.0 mmol/L | Maintained RAASi | NR | NR | NR | Stage 3: 50.9 | NR | NR | NR | NR |
|  |  |  | Reduced RAASi | NR | NR | NR | Stage 3: 51.5 | NR | NR | NR | NR |
| Tuttle 2024 ([32](#_ENREF_32)) | Patients with CKD | NR | Pooled* BI 690517 3 mg | 147 | ACEi: 36 ARBs: 65 Others: GLP-1 receptor agonists: 10 | DM: 78 | NR | Mean (SD): 51 (16.5) | NR | NR | Mean (SD): 4.32 (0.4) |
|  |  |  | Pooled* BI 690517 10 mg | 146 | ACEi: 22 ARBs: 76 Others: GLP-1 receptor agonists: 7 | DM: 67 | NR | Mean (SD): 53.1 (19.6) | NR | NR | Mean (SD): 4.26 (0.47) |
|  |  |  | Pooled* BI 690517 20 mg | 146 | ACEi: 31 ARBs: 68 Others: GLP-1 receptor agonists: 10 | DM: 69 | NR | Mean (SD): 51.6 (16.5) | NR | NR | Mean (SD): 4.35 (0.39) |
|  |  |  | Pooled* BI 690517 placebo | 147 | ACEi: 33 ARBs: 67 Others: GLP-1 receptor agonists: 8 | DM: 68 | NR | Mean (SD): 52.1 (18.2) | NR | NR | Mean (SD): 4.3 (0.45) |
| Guney 2009 ([33](#_ENREF_33)) | Patients with stage 1-3 non-diabetic CKD | sK+ >5.5 mEq/L | ACEIs and/or ARBs + spironolactone | 15 | ACEIs and/or ARBs: 100 | DM: 0 | Stage 1-3: 100 | NR | NR | NR | NR |
|  |  |  | ACEIs and/or ARBs | 15 | ACEIs and/or ARBs: 100 | DM: 0 | Stage 1-3: 100 | NR | NR | NR | NR |
| Edwards 2009 ([34](#_ENREF_34)) | Patients with stage 2-3 non-diabetic CKD | sK+ >6.5 or >6.0 mEq/L | Spironolactone | 56 | ACEi: 69.6 ARBs: 33.9 CCB: 30.4 Statins: 30.4 Diuretics: 23.2 | DM: 0 | Stage 2-3: 100 | Mean (SD): 53 (11) | Mean (SD): 1.4 (0.38) | NR | Mean (SD): 4.3 (0.3) |
|  |  |  | Placebo | 56 | ACEi: 67.9 ARBs: 33.9 β -blockers: 26.8 CCB: 23.2 Statins: 48.2 Diuretics: 32.1 | DM: 0 | Stage 2-3: 100 | Mean (SD): 49 (12) | Mean (SD): 1.5 (0.39) | NR | Mean (SD): 4.4 (0.8) |

*Pooled groups include participants who received BI 690517 either as monotherapy or in combination with empagliflozin.

**Abbreviations**: α: alpha; β: beta; ACEi: angiotensin-converting enzyme inhibitors; ADPKD: Autosomal dominant polycystic kidney disease AFib: atrial fibrillation; AIDS: acquired immune deficiency virus; AKI: acute kidney injury; ARB: angiotensin-receptor blockers; ARNi: angiotensin receptor/neprilysin inhibitors; ASA: Acetylsalicylic acid; CAD: coronary artery disease; CBD: cerebrovascular disease; CCB: calcium channel blocker; CKD: chronic kidney disease; COPD: chronic obstructive pulmonary disease; CVE: Cerebrovascular event; CVD: cardiovascular disease; DHP: dihydropyridine; DM: DM mellitus; DPP-4 inhibitors: dipeptidyl peptidase 4; DRI: direct renin inhibitors; eGFR: estimated glomerular filtration rate; ESRD: end-stage renal disease; g/dl: gram per decilitre GLP-1: glucagon-like peptide 1; HF: heart failure; HFpEF: heart failure with preserved ejection fraction; HFrEF: heart failure with reduced ejection fraction; HIV: human immune deficiency virus; HT: hypertension; ICD-10: International Classification of Diseases, Tenth Revision; ICD-9: International Classification of Diseases, Ninth Revision; IgA: immunoglobulin A IHD: ischemic heart disease; IQR: interquartile range; K+: potassium; KDIGO: kidney disease improving global outcomes; LDL: low density lipoprotein; LVH: left ventricular hypertrophy; m2: square metre; mEq/L: milliequivalents per litre; mg/dL: milligram per decilitre; mg: milligram; MI: myocardial infarction; mL/min: millilitre per minute; mmol/L: millimoles per litre; MRA: mineralocorticoid-receptor antagonists; NA: not applicable; NaHCO3: sodium bicarbonate; NR: not reported; NSAID: non-steroidal anti-inflammatory drugs; OAD: oral antidiabetic drugs; PAD: peripheral arterial disease; PAOD: peripheral artery occlusive disease; pK+: plasma potassium; PKD: polycystic kidney disease PVD: peripheral vascular disease; qd: once daily; RAASi: renin-angiotensin-aldosterone system inhibitors; RRT: renal replacement therapy; sAlb: serum albumin; sCr: serum creatinine; SD: standard deviation; SGLT2i: sodium/glucose cotransporter-2 inhibitors; sK+: serum potassium; SPS: sodium polystyrene sulfonate; vs: versus; SZC: sodium zirconium cyclosilicate.

Table S15. Risk factors for hyperkalaemia

| **Study** | **Population** | **Risk factor** | **Hyperkalaemia definition** | **Effect estimate (e.g., Odds ratio, Risk ratio, prevalence ratio etc.)** | **Value (95% CI)** | **p-value** |
| --- | --- | --- | --- | --- | --- | --- |
| Thomsen 2018 ([124](#_ENREF_124)) | Patients with stage 1-5 CKD or on dialysis + Hyperkalaemia | Females | sK+ >5.0 mmol/L | Prevalence ratio | 1.0 (0.99-1.01) | NR |
|  |  | Stage 1 and 2 CKD |  |  | 1.05 (0.91-1.21) | NR |
|  |  | Stage 3a CKD |  |  | 0.59 (0.58-0.6) | NR |
|  |  | Stage 3b CKD |  |  | 1.31 (1.28-1.34) | NR |
|  |  | Stage 4 CKD |  |  | 2.88 (2.78-3) | NR |
|  |  | Stage 5 CKD |  |  | 3.99 (3.67-4.34) | NR |
|  |  | Dialysis |  |  | 3.93 (3.25-4.75) | NR |
|  |  | No eGFR measurements available |  |  | 0.44 (0.38-0.52) | NR |
|  |  | DM |  |  | 1.74 (1.69-1.79) | NR |
|  |  | HF |  |  | 2.31 (2.23-2.4) | NR |
|  |  | HT |  |  | 1.14 (1.13-1.15) | NR |
|  |  | MI |  |  | 1.47 (1.42-1.53) | NR |
|  |  | PVD |  |  | 1.69 (1.62-1.76) | NR |
|  |  | CBD |  |  | 1.25 (1.21-1.28) | NR |
|  |  | Chronic pulmonary disease |  |  | 1.71 (1.66-1.77) | NR |
|  |  | Connective tissue disease |  |  | 1.23 (1.16-1.31) | NR |
|  |  | Ulcer disease |  |  | 1.48 (1.42-1.55) | NR |
|  |  | Any liver disease |  |  | 2.42 (2.19-2.68) | NR |
|  |  | Any malignant disease |  |  | 1.39 (1.35-1.43) | NR |
|  |  | AFib or flutter |  |  | 1.57 (1.52-1.62) | NR |
|  |  | Valvular heart disease |  |  | 1.71 (1.63-1.8) | NR |
|  |  | Alcoholism-related disorders |  |  | 1.84 (1.75-1.93) | NR |
|  |  | Medical obesity |  |  | 1.68 (1.59-1.78) | NR |
|  |  | Use of ACEi |  |  | 1.45 (1.42-1.48) | NR |
|  |  | Use of ARBs |  |  | 1.06 (1.03-1.1) | NR |
|  |  | Use K+ sparing diuretics (spironolactone) |  |  | 2.53 (2.44-2.63) | NR |
|  |  | Use of K+ supplement |  |  | 1.59 (1.55-1.62) | NR |
| Nakhoul 2015 ([90](#_ENREF_90)) | Patients with stage 3-4 non-dialysis dependent CKD | Age (per 10 years incremental increase) | sK+ >5.0 mmol/L | Odds ratio | 0.9 (0.87-0.92) | NR |
|  |  | Male sex |  |  | 1.37 (1.28-1.47) | NR |
|  |  | African American race |  |  | 0.92 (0.83-1.02) | NR |
|  |  | eGFR (per 5 mL/min decrease) |  |  | 1.25 (1.23-1.27) | NR |
|  |  | BMI <18.5 kg/m2 |  |  | 1.6 (1.23-2.08) | NR |
|  |  | 18.5-24.9 kg/ m2 |  |  | Reference | NA |
|  |  | BMI 25-29.9 kg/m2 |  |  | 0.87 (0.8-0.96) | NR |
|  |  | BMI ≥30 kg/ m2 |  |  | 0.77 (0.7-0.85) | NR |
|  |  | BMI Missing |  |  | 0.97 (0.81-1.16) | NR |
|  |  | DM |  |  | 1.53 (1.41-1.66) | NR |
|  |  | HT |  |  | 1.03 (0.93-1.15) | NR |
|  |  | Malignancy |  |  | 1.12 (1.03-1.21) | NR |
|  |  | CAD |  |  | 1.09 (1-1.19) | NR |
|  |  | Congestive HF |  |  | 0.95 (0.84-1.07) | NR |
|  |  | COPD |  |  | 1.02 (0.9-1.16) | NR |
|  |  | Use of ACEi/ARBs |  |  | 1.4 (1.29-1.52) | NR |
|  |  | Use of β-blockers |  |  | 1.06 (0.98-1.14) | NR |
| NephroTest  Wagner 2017 ([43](#_ENREF_43)) | Patients with stage 1-5 non-dialysis-dependent CKD | Age (per year) | sK+ >5.0 mmol/L | Odds ratio | 0.98 (0.96-0.99) | NR |
|  |  | Women vs men |  |  | 0.47 (0.3-0.72) | NR |
|  |  | Sub-Saharan vs other ethnicity |  |  | 1.15 (0.66-1.99) | NR |
|  |  | mGFR (mL/min/1.73 m2): <15 |  |  | 29.65 (10.87-80.88) | NR |
|  |  | mGFR (mL/min/1.73 m2): 15-30 |  |  | 13.58 (5.71-32.3) | NR |
|  |  | mGFR (mL/min/1.73 m2: 30-45 |  |  | 5.7 (2.42-13.45) | NR |
|  |  | mGFR (mL/min/1.73 m2): 45-60 |  |  | 2.7 (1.07-6.85) | NR |
|  |  | mGFR (mL/min/1.73 m2): >60 |  |  | Reference | NA |
|  |  | Cardio-vascular history |  |  | 0.81 (0.51-1.28) | NR |
|  |  | ACR (mg/mmol): <3 |  |  | Reference | NA |
|  |  | ACR (mg/mmol): 3-30 |  |  | 1.25 (0.76-2.08) | NR |
|  |  | ACR (mg/mmol): >30 |  |  | 1.13 (0.67-1.9) | NR |
|  |  | DM |  |  | 1.56 (1.04-2.34) | NR |
|  |  | Urine K+ |  |  | 1.01 (1.0-1.01) | NR |
|  |  | Serum albumin: ≥35 |  |  | Reference | NA |
|  |  | Serum albumin: <35 |  |  | 1.23 (0.76-1.98) | NR |
|  |  | sK+ increasing drugs |  |  | 2.5 (1.17-5.35) | NR |
|  |  | sK+ lowering drugs |  |  | 1.01 (0.69-1.49) | NR |
|  |  | BMI: <19 |  |  | 1.49 (0.65-3.43) | NR |
|  |  | BMI: 19-25 |  |  | Reference | NA |
|  |  | BMI: 25-30 |  |  | 0.78 (0.51-1.2) | NR |
|  |  | BMI: >30 |  |  | 1.05 (0.65-1.73) | NR |
|  |  | Never smoked |  |  | Reference | NA |
|  |  | Smoking status: Former smoker |  |  | 1.02 (0.67-1.54) | NR |
|  |  | Smoking status: Active smoker |  |  | 1.66 (1.03-2.67) | NR |
| RRI-CKD Cohort study  Korgaonkar 2010 ([91](#_ENREF_91)) | Patients with stage 3-5 CKD | Male gender | sK+ ≥5.5 mmol/L | Estimate | 0.18 (NR) | p<0.0001 |
|  |  | Serum CO2 (mEq/L) |  |  | -0.05 (NR | p<0.0001 |
|  |  | eGFR (per 10 mL/min/1.73 m2 |  |  | -0.02 (NR) | p=0.39 |
|  |  | DM |  |  | 0.1 (NR) | p=0.029 |
|  |  | ACEi use |  |  | 0.21 (NR) | p<0.0001 |
|  |  | Diuretic use |  |  | -0.07 (NR) | p=0.76 |
|  |  | CCB use |  |  | -0.06 (NR) | p=0.12 |
|  |  | Statin use |  |  | 0.11 (NR) | p=0.001 |
|  |  | Male gender |  | Estimate | 0.18 (NR) | p<0.0001 |
|  |  | Serum CO2 (mEq/L) |  |  | -0.03 (NR) | p<0.0001 |
|  |  | eGFR (per 10 mL/min/1.73 m2 |  |  | -0.06 (NR) | p<0.0001 |
|  |  | DM |  |  | 0.06 (NR) | p=0.03 |
|  |  | ACEi use |  |  | 0.17 (NR) | p<0.0001 |
|  |  | Diuretic use |  |  | -0.12 (NR) | p<0.0001 |
|  |  | CCB use |  |  | 0.09 (NR) | p<0.0001 |
|  |  | Statin use |  |  | 0.12 (NR) | p<0.0001 |
| Hwang 2011 ([66](#_ENREF_66)) | Patients with ESRD on maintenance haemodialysis | Age at start of study, years | NR | NR | NR | NR |
|  |  | Age at start of haemodialysis, years |  |  | NR | NR |
|  |  | Serum concentrations, Albumin, g/dL |  |  | NR | NR |
|  |  | Serum concentrations, Albumin, g/dL |  |  | NR | NR |
|  |  | Serum concentrations, Creatinine, mg/dL |  |  | NR | NR |
| Brookes 2021 ([110](#_ENREF_110)) | Patients with stage 1-5 non-dialysis dependent CKD | Male | sK+ ≥5.5 mmol/L | Unadjusted Odds ratio (Univariate analysis) | 1.49 (1.37-1.61) | p<0.001 |
|  |  | Female |  |  | Reference | NA |
|  |  | Age: ≥65 years |  |  | 0.86 (0.78-0.96) | p=0.008 |
|  |  | Age: <65 years |  |  | Reference | NA |
|  |  | ATSI |  |  | 1.10 (0.56-2.18) | p=0.78 |
|  |  | Not ATSI or unknown |  |  | Reference | NA |
|  |  | eGFR (per 5 mL/min/1.73 m2 decline) |  |  | 1.32 (1.3-1.34) | p<0.001 |
|  |  | Acute MI |  |  | 1.28 (1-1.63) | p=0.048 |
|  |  | HF |  |  | 1.28 (1.15-1.42) | p<0.001 |
|  |  | PVD |  |  | 1.2 (0.9-1.6) | p=0.209 |
|  |  | CBD |  |  | 0.63 (0.48-0.83) | p=0.001 |
|  |  | Dementia |  |  | 0.78 (0.6-1.01) | p=0.064 |
|  |  | Chronic pulmonary disease |  |  | 0.81 (0.68-0.96) | p=0.013 |
|  |  | Rheumatic disease |  |  | 0.68 (0.38-1.21) | p=0.19 |
|  |  | Peptic ulcer disease |  |  | 1.53 (0.96-2.43) | p=0.072 |
|  |  | Liver disease |  |  | 1.52 (1.32-1.75) | p<0.001 |
|  |  | DM |  |  | 1.94 (1.79-2.1) | p<0.001 |
|  |  | Hemiplegia/paraplegia |  |  | 0.85 (0.59-1.2) | p=0.353 |
|  |  | Malignancy |  |  | 0.77 (0.66-0.89) | p=0.001 |
|  |  | AIDS/HIV |  |  | 2.91 (0.84-10.14) | p=0.093 |
|  |  | Infectious disease |  |  | 0.88 (0.72-1.08) | p=0.216 |
|  |  | Neoplasm |  |  | 0.68 (0.56-0.83) | p<0.001 |
|  |  | Haematology |  |  | 1.12 (0.89-1.41) | p=0.344 |
|  |  | Endocrine/metabolic |  |  | 3.64 (3.2-4.15) | p<0.001 |
|  |  | Mental/behavioural |  |  | 0.68 (0.52-0.89) | p=0.003 |
|  |  | Neurological |  |  | 1.03 (0.93-1.13) | p=0.005 |
|  |  | Cardiovascular |  |  | 1.03 (0.93-1.13) | p=0.622 |
|  |  | Respiratory |  |  | 0.77 (0.68-0.87) | p<0.001 |
|  |  | Gastrointestinal |  |  | 0.8 (0.71-0.91) | p=0.001 |
|  |  | Dermatological |  |  | 0.65 (0.47-0.89) | p=0.008 |
|  |  | Musculoskeletal |  |  | 0.63 (0.5-0.78) | p<0.001 |
|  |  | Genitourinary |  |  | 2.15 (1.91-2.43) | p<0.001 |
|  |  | Injury/poisoning |  |  | 0.76 (0.66-0.88) | p<0.001 |
|  |  | ACEi |  |  | 0.82 (0.73-0.93) | p=0.001 |
|  |  | ARB |  |  | 0.72 (0.63-0.82) | p<0.001 |
|  |  | MRA |  |  | 0.99 (0.87-1.12) | p=0.853 |
|  |  | β-blocker |  |  | 1.1 (1.01-1.19) | p=0.022 |
|  |  | CCB |  |  | 1.3 (1.18-1.43) | p<0.001 |
|  |  | Loop diuretic |  |  | 1.19 (1.1-1.29) | p<0.001 |
|  |  | Thiazide diuretic |  |  | 0.82 (0.7-0.96) | p=0.013 |
|  |  | Male |  | Adjusted Odds ratio (Multivariate analysis) | 1.54 (1.41-1.67) | p<0.001 |
|  |  | Female |  |  | Reference | NA |
|  |  | Age: ≥65 years |  |  | 0.94 (0.83-1.06) | p=0.303 |
|  |  | Age: <65 years |  |  | Reference | NA |
|  |  | ATSI |  |  | NA | NA |
|  |  | Not ATSI or unknown |  |  | Reference | NA |
|  |  | eGFR (per 5 mL/min/1.73 m2 decline) |  |  | 1.31 (1.29-1.32) | p<0.001 |
|  |  | Acute MI |  |  | 1.12 (0.86-1.45) | p=0.408 |
|  |  | HF |  |  | 1.15 (1.01-1.31) | p=0.042 |
|  |  | PVD |  |  | NA | NA |
|  |  | CBD |  |  | 0.94 (0.7-1.25) | p=0.663 |
|  |  | Dementia |  |  | NA | NA |
|  |  | Chronic pulmonary disease |  |  | 1 (0.82-1.21) | p=0.967 |
|  |  | Rheumatic disease |  |  | NA | NA |
|  |  | Peptic ulcer disease |  |  | NA | NA |
|  |  | Liver disease |  |  | 1.6 (1.35-1.9) | p<0.001 |
|  |  | DM |  |  | 1.4 (1.28-1.52) | p<0.001 |
|  |  | Hemiplegia/paraplegia |  |  | NA | NA |
|  |  | Malignancy |  |  | 0.94 (0.77-1.15) | p=0.641 |
|  |  | AIDS/HIV |  |  | NA | NA |
|  |  | Infectious disease |  |  | NA | NA |
|  |  | Neoplasm |  |  | 1.07 (0.81-1.41) | p=0.641 |
|  |  | Haematology |  |  | NR (NR-NR) | NR |
|  |  | Endocrine/metabolic |  |  | 2.93 (2.5-3.43) | p<0.001 |
|  |  | Mental/behavioural |  |  | 0.87 (0.66-1.14) | p=0.312 |
|  |  | Neurological |  |  | 1.03 (0.77-1.36) | p=0.864 |
|  |  | Cardiovascular |  |  | NA | NA |
|  |  | Respiratory |  |  | 0.99 (0.85-1.14) | p=0.858 |
|  |  | Gastrointestinal |  |  | 0.98 (0.84-1.14) | p=0.784 |
|  |  | Dermatological |  |  | 0.74 (0.53-1.04) | p=0.079 |
|  |  | Musculoskeletal |  |  | 0.87 (0.68-1.11) | p=0.261 |
|  |  | Genitourinary |  |  | 1.3 (1.12-1.51) | p<0.001 |
|  |  | Injury/poisoning |  |  | 1 (0.85-1.18) | p=0.987 |
|  |  | ACEi |  |  | 1.01 (0.89-1.15) | p=0.876 |
|  |  | ARB |  |  | 0.9 (0.78-1.03) | p=0.136 |
|  |  | MRA |  |  | NA | NA |
|  |  | β-blocker |  |  | 0.91 (0.83-1) | p=0.058 |
|  |  | CCB |  |  | 0.99 (0.89-1.1) | p=0.85 |
|  |  | Loop diuretic |  |  | 0.89 (0.81-0.99) | p=0.029 |
|  |  | Thiazide diuretic |  |  | 0.76 (0.64-0.91) | p=0.003 |
| Fukushima CKD Cohort  Tanaka 2021 ([80](#_ENREF_80)) | Pre-dialysis CKD patients with eGFR <60 mL/min/1.73 m2 | Age (years) | sK+ ≥5.0 mmol/L | Odds ratio | 1.01 (0.99-1.03) | p=0.597 |
|  |  | Male gender |  |  | 1.48 (0.95-2.3) | p=0.08 |
|  |  | Body mass index (kg/ m2) |  |  | 1.02 (0.98-1.07) | p=0.341 |
|  |  | History of cardiovascular disease |  |  | 0.71 (0.44-1.14) | p=0.157 |
|  |  | History of malignancy |  |  | 1.51 (0.94-2.42) | p=0.088 |
|  |  | HT |  |  | 1.1 (0.49-2.47) | p=0.812 |
|  |  | DM |  |  | 1.47 (0.94-2.31) | p=0.089 |
|  |  | eGFR (mL/min./1.73 m2 |  |  | 0.95 (0.94-0.97) | p<0.001 |
|  |  | Serum albumin (g/dL) |  |  | 1 (0.92-1.08) | p=0.906 |
|  |  | Na-Cl <34 mmol/L |  |  | 1.76 (1.09-2.83) | p=0.02 |
|  |  | ARB or ACEi use |  |  | 0.75 (0.43-1.31) | p=0.312 |
|  |  | Diuretic use |  |  | 0.51 (0.31-0.86) | p=0.012 |
|  |  | Cation exchange resin |  |  | 2.79 (1.58-4.94) | p<0.001 |
| Belmar Vega 2019 ([114](#_ENREF_114)) | Patients with stage 3-5 CKD | GFR | sK+ >5.5 mmol/L | Odds ratio | 0.96 (0.94-0.98) | p<0.001 |
|  |  | Plasma sodium |  |  | 0.906 (0.847-0.968) | p=0.004 |
|  |  | ACEi or ARB use |  |  | 2.04 (1.223-3.402) | p=0.006 |
|  |  | Metformin use |  |  | 6.027 (2.07-17.547) | p=0.001 |
| Sharma 2020 ([38](#_ENREF_38)) | Patients with stage 1-5 CKD | CKD stage 2 vs 1 | 1. ≥2 sK+ >5.0 mmol/L on different dates (Logical Observation Identifiers Names and Codes) 2. ≥2 claims with principal or secondary diagnosis of hyperkalaemia (ICD-10) 3. 1 claim with hyperkalaemia and sK+ >5.0 mmol/L 4. ≥1 dispensed prescription for SPS or patiromer | Odds ratio | 1.65 (1.38-1.98) | p<0.0001 |
|  |  | CKD stage 3 vs 1 |  |  | 3.34 (2.77-4.02) | p<0.0001 |
|  |  | CKD stage 4 vs 1 |  |  | 7.47 (6.02-9.28) | p<0.0001 |
|  |  | CKD stage 5 vs 1 |  |  | 11.62 (7.9-17.07) | p<0.0001 |
|  |  | K+ per 0.1mmol/L higher |  |  | 8.59 (7.85-9.4) | p<0.0001 |
|  |  | Calcineurin inhibitors use |  |  | 4.43 (2.98-6.97) | p<0.0001 |
|  |  | DM |  |  | 1.51 (1.39-1.63) | p<0.0001 |
|  |  | Northeast vs Midwest |  |  | 1.14 (1.01-1.29) | p=0.0170 |
|  |  | South vs Midwest |  |  | 1.57 (1.4-1.77) | p<0.0001 |
|  |  | West vs Midwest |  |  | 1.32 (1.09-1.58) | p=0.0102 |
|  |  | Suburban vs Rural |  |  | 1.05 (0.96-1.17) | p=0.0568 |
|  |  | Urban vs Rural |  |  | 1.33 (1.22-1.46) | p<0.0001 |
|  |  | Hyperlipidaemia |  |  | 1.47 (1.28-1.67) | p<0.0001 |
|  |  | Osteoporosis |  |  | 1.27 (1.12-1.45) | p<0.0001 |
|  |  | MRA use |  |  | 1.26 (1.08-1.46) | p<0.0001 |
|  |  | ACEi use |  |  | 1.25 (1.16-1.35) | p<0.0001 |
|  |  | PAD |  |  | 1.19 (1.09-1.31) | p=0.0002 |
|  |  | Malignant neoplasms |  |  | 1.16 (1.04-1.29) | p=0.0107 |
|  |  | COPD |  |  | 1.11 (1-1.23) | p=0.0498 |
|  |  | Number of comorbid conditions |  |  | 1.07 (1.05-1.08) | p<0.0001 |
|  |  | Haemoglobin, per 1g/dL higher |  |  | 0.91 (0.89-0.94) | p=0.0028 |
|  |  | Optimal RAASi dose |  |  | 0.91 (0.83-0.99) | p=0.0097 |
|  |  | Inpatient admission-all cause |  |  | 0.87 (0.78-0.97) | p=0.0072 |
|  |  | Commercial vs Medicare plan |  |  | 0.85 (0.75-0.95) | p=0.0035 |
|  |  | Primary care visit |  |  | 0.68 (0.62-0.74) | p<0.0001 |
|  |  | Female vs Male |  |  | 0.63 (0.57-0.69) | p<0.0001 |
|  |  | K+ sparing diuretics use |  |  | 0.53 (0.37-0.74) | p=0.0003 |
| Lin 2023 ([74](#_ENREF_74)) | Patients with ESRD under maintenance dialysis + HF | MRA use | sK+ >6.0 mEq/L | Hazard ratio | 1.34 (1.17-1.54) | p<0.001 |
| Sadjadi 2009 ([45](#_ENREF_45)) | Patients with stage 1-5 CKD | ARB use | sK+ >5.0 mEq/L | Odds ratio (Crude) | 1.74 (NR) | p<0.001 |
|  |  |  |  | Odds ratio (After adjusting for blood glucose and GFR) | 1.43 (NR) | p=0.001 |
|  |  |  |  | Odds ratio (After adjusting for blood glucose and serum creatinine) | 1.56 (NR) | p<0.001 |
| Khosla 2009 ([68](#_ENREF_68)) | Patients with stage 2 or 3 CKD + Resistant HT | Baseline eGFR ≤45 ml/min/1.73 m2 | sK+ >5.5 mEq/L | Odds ratio | 2.97 (1.14-21.3) | p<0.03 |
|  |  | Baseline eGFR ≤45 mL/min/1.73 m2 + sK+ >4.5 mEq/l |  |  | 8.71 (2.89-24.8) | p<0.01 |
|  |  | Baseline eGFR ≤45 mL/min/1.73 m2 + >30% reduction in eGFR |  |  | 7.76 (2.13-22.8) | p<0.01 |
|  |  | Baseline eGFR ≤45 mL/min/1.73 m2 + >15 mm Hg in systolic BP |  |  | 3.98 (0.89-27.1) | p=0.18 |
| AASK  Weinberg 2009 ([22](#_ENREF_22)) | Patients with non-diabetic hypertensive CKD (eGFR 20-65 mL/min/1.73 m2) | β-blocker vs CCB | sK+ >5.5 mEq/L | Hazard ratio (Univariate analysis) | 2.07 (0.7-6.16) | p=0.19 |
|  |  | ACEi vs CCB |  |  | 3.84 (1.35-10.89) | p=0.01 |
|  |  | ACEI vs β-blocker |  |  | 1.85 (1.02-3.36) | p=0.04 |
|  |  | Low vs usual blood pressure |  |  | 1.1 (0.64-1.91) | p=0.72 |
|  |  | Age at randomisation, 10 years |  |  | 1.26 (0.95-1.67) | p=0.1 |
|  |  | Female sex |  |  | 0.85 (0.48-1.53) | p=0.6 |
|  |  | Baseline NSAID use |  |  | 1.02 (0.43-2.38) | p=0.97 |
|  |  | Mean baseline GFR ≤30 vs >50 mL/min/1.73 m2 |  |  | 13.09 (5.83-29.39) | p<0.001 |
|  |  | Mean baseline GFR 31-40 vs >50 mL/min/1.73 m2 |  |  | 5.44 (2.3-12.85) | p<0.001 |
|  |  | Mean baseline GFR 41-50 vs >50 mL/min/1.73 m2 |  |  | 0.85 (0.23-3.22) | p=0.82 |
|  |  | BMI ≤25 vs >25 to ≤30 kg/m2 |  |  | 2.68 (1.36-5.29) | p=0.004 |
|  |  | BMI >30 vs >25 to ≤30 kg/m2 |  |  | 0.77 (0.38-1.59) | p=0.48 |
|  |  | Baseline UP/Cr 0.08-0.22 vs ≤0.08 |  |  | 2.7 (1.23-5.93) | p=0.01 |
|  |  | Baseline UP/Cr 0.22-0.66 vs ≤0.08 |  |  | 1.83 (0.73-4.6) | p=0.2 |
|  |  | Baseline UP/Cr >0.66 vs ≤0.08 |  |  | 5.86 (2.88-11.92) | p<0.001 |
|  |  | Baseline glucose level 100-115 vs <100 mg/dL |  |  | 0.63 (0.3-1.35) | p=0.24 |
|  |  | Baseline glucose level >115 vs <100 mg/dL |  |  | 0.14 (0.02-1.04) | p=0.05 |
|  |  | Baseline K+ level 4-5 vs <4 mEq/L |  |  | 18.48 (2.53-135.1) | p=0.004 |
|  |  | Baseline K+ level >5 vs <4 mEq/L |  |  | 85.54 (11.36-644.2) | p<0.001 |
|  |  | β-blocker vs CCB |  | Hazard ratio (Multivariable analysis) | 2.45 (0.79-7.65) | p=0.12 |
|  |  | ACEI vs CCB |  |  | 7 (2.29-21.39) | p<0.001 |
|  |  | ACEI vs β-blocker |  |  | 2.85 (1.5-5.42) | p=0.001 |
|  |  | Low vs usual BP |  |  | 1.28 (0.72-2.29) | p=0.4 |
|  |  | Age at randomisation, 10 years |  |  | 1.4 (1.05-1.88) | p=0.02 |
|  |  | Female sex |  |  | 0.52 (0.28-0.98) | p=0.04 |
|  |  | Baseline NSAID use |  |  | 0.93 (0.38-2.32) | p=0.88 |
|  |  | Mean baseline GFR ≤30 vs >50 mL/min/1.73 m2 |  |  | 6.81 (2.67-17.35) | p<0.001 |
|  |  | Mean baseline GFR 31-40 vs >50 mL/min/1.73 m2 |  |  | 3.61 (1.42-9.18) | p=0.007 |
|  |  | Mean baseline GFR 41-50 vs >50 mL/min/1.73 m2 |  |  | 0.61 (0.16-2.35) | p=0.47 |
|  |  | BMI ≤25 vs >25 to ≤30 kg/m2 |  |  | 1.92 (0.95-3.89) | p=0.07 |
|  |  | BMI >30 vs >25 to ≤30 kg/m2 |  |  | 0.82 (0.39-1.74) | p=0.61 |
|  |  | Baseline UP/Cr 0.08-0.22 vs ≤0.08 |  |  | 2.27 (0.96-5.36) | p=0.06 |
|  |  | Baseline UP/Cr 0.22-0.66 vs ≤0.08 |  |  | 1.15 (0.42-3.14) | p=0.78 |
|  |  | Baseline UP/Cr >0.66 vs ≤0.08 |  |  | 3.63 (1.58-8.34) | p=0.002 |
|  |  | Baseline glucose level 100-115 vs <100 mg/dL |  |  | 1.08 (0.5-2.36) | p=0.84 |
|  |  | Baseline glucose level >115 vs <100 mg/dL |  |  | 0.22 (0.03-1.59) | p=0.13 |
|  |  | Baseline K+ level 4-5 vs <4 mEq/L |  |  | 14.81 (2.01-109.1) | p=0.008 |
|  |  | Baseline K+ level >5 vs <4 mEq/L |  |  | 53.72 (6.97-414.2) | p<0.001 |
| Knoll 2002 ([92](#_ENREF_92)) | Patients with ESRD on chronic haemodialysis | RAASi Yes vs No | sK+ ≥5.5 mmol/L | Unadjusted odds ratio | 1.7 (1.4-2.2) | NR |
|  |  | Anuria Yes vs No |  |  | 2.3 (1.8-3.0) | NR |
|  |  | Anuric (RAASi use) vs Anuric (control) |  |  | 1.7 (1.2-2.2) | p=0.064 |
|  |  | Non-anuric (RAASi use) vs Non-anuric (control) |  |  | 2.0 (1.3-3.2) | NR |
|  |  | RAASi Yes vs No |  | Adjusted odds ratio | 2.2 (1.4-3.4) | NR |
|  |  | Anuria Yes vs No |  |  | 3.0 (1.8-5.0) | NR |
|  |  | Anuric (RAASi use) vs Anuric (control) |  |  | 2.3 (1.3-4.2) | NR |
|  |  | Non-anuric (RAASi use) vs Non-anuric (control) |  |  | 2.1 (1.0-4.1) | NR |
|  |  | RAASi use vs Control | sK+ ≥6.0 mmol/L | Unadjusted odds ratio | 1.5 (1.0-2.1) | NR |
|  |  |  |  | Adjusted odds ratio | 1.5 (0.9-2.6) | NR |
|  |  | Anuric vs Non-anuric |  | Adjusted odds ratio | 2.0 (1.1-3.7) | NR |
| Riccio 2022 ([70](#_ENREF_70)) | Patients with stage 1-5 CKD | Male vs Female | sK+ >5.5 mmol/L | Odds ratio | 0.48 (0.24-0.93) | p=0.0299 |
|  |  | DM |  |  | 2.2 (1.33-3.63) | p<0.01 |
|  |  | Hearth failure |  |  | 2.13 (0.97-4.69) | p=0.0597 |
|  |  | Age |  |  | 1 (0.98-1.01) | p=0.641 |
|  |  | Smoker vs previous smoker |  |  | 0.23 (0.11-0.49) | p<0.001 |
|  |  | Smoker vs non-smoker |  |  | 0.3 (0.19-0.5) | p<0.001 |
|  |  | CKD stage 1 vs CKD stage 2 |  |  | 1.52 (0.55-4.2) | p=0.4205 |
|  |  | CKD stage 1 vs CKD stage 3 |  |  | 3.63 (1.43-9.25) | p<0.01 |
|  |  | CKD stage 1 vs CKD stage 4 |  |  | 10.67 (3.98-28.61) | p<0.01 |
|  |  | CKD stage 1 vs CKD stage 5 |  |  | 6.72 (2.47-18.28) | p<0.01 |
| DRINK  Tang 2021 ([11](#_ENREF_11)) | Patients with stage 3 or 4 non-diabetic CKD | Add-on aliskiren use | pK+ ≥5.5 mmol/L | Adjusted Hazard ratio | 7.71 (1.14-52.3) | p=0.04 |
|  |  | Higher LDL level |  |  | 2.69 (1.19-6.11) | p=0.02 |
| Fang 2018 ([119](#_ENREF_119)) | Patients with CKD hospitalised for MI | Age 66-74 vs 75-84 years | Primary or secondary diagnosis code of 276.7 in inpatient claims | Crude Hazard ratio (ITT analysis) | 1.21 (1-1.47) | NR |
|  |  | Age 66-74 vs ≥85 years |  |  | 1.36 (1.11-1.67) | NR |
|  |  | Female vs Male |  |  | 1.19 (1.01-1.41) | NR |
|  |  | Race: White vs Asian |  |  | 1.07 (0.59-1.94) | NR |
|  |  | Race: White vs Black |  |  | 2.29 (1.85-2.85) | NR |
|  |  | Race: White vs Hispanic |  |  | 1.87 (1.27-2.73) | NR |
|  |  | Race: White vs Other |  |  | 1.97 (1.23-3.16) | NR |
|  |  | Baseline CKD |  |  | 2.52 (2.14-2.97) | NR |
|  |  | Age 66-74 vs 75-84 |  | Adjusted Hazard ratio (ITT analysis) | 1.2 (0.98-1.47) | NR |
|  |  | Age 66-74 vs ≥ 85 |  |  | 1.33 (1.05-1.68) | NR |
|  |  | Female vs Male |  |  | 0.96 (0.8-1.14) | NR |
|  |  | Race: White vs Asian |  |  | 0.89 (0.49-1.64) | NR |
|  |  | Race: White vs Black |  |  | 1.57 (1.24-1.98) | NR |
|  |  | Race: White vs Hispanic |  |  | 1.24 (0.83-1.86) | NR |
|  |  | Race: White vs Other |  |  | 1.54 (0.96-2.47) | NR |
|  |  | Baseline CKD |  |  | 1.41 (1.11-1.77) | NR |
|  |  | Age 66-74 vs 75-84 |  | Crude Hazard ratio (As-treated analysis) | 1.37 (1.04-1.81) | NR |
|  |  | Age 66-74 vs ≥85 |  |  | 1.45 (1.07-1.97) | NR |
|  |  | Female vs Male |  |  | 1.09 (0.86-1.38) | NR |
|  |  | Race: White vs Asian |  |  | 0.97 (0.4-2.35) | NR |
|  |  | Race: White vs Black |  |  | 1.84 (1.31-2.57) | NR |
|  |  | Race: White vs Hispanic |  |  | 1.99 (1.18-3.36) | NR |
|  |  | Race: White vs Other |  |  | 1.96 (1.01-3.81) | NR |
|  |  | Baseline CKD |  |  | 2.72 (2.16-3.43) | NR |
|  |  | Age 66-74 vs 75-84 |  | Adjusted Hazard ratio (As-treated analysis) | 1.4 (1.05-1.87) | NR |
|  |  | Age 66-74 vs ≥85 |  |  | 1.51 (1.08-2.11) | NR |
|  |  | Female vs Male |  |  | 0.88 (0.69-1.13) | NR |
|  |  | Race: White vs Asian |  |  | 0.71 (0.29-1.73) | NR |
|  |  | Race: White vs Black |  |  | 1.25 (0.88-1.78) | NR |
|  |  | Race: White vs Hispanic |  |  | 1.32 (0.75-2.32) | NR |
|  |  | Race: White vs Other |  |  | 1.52 (0.78-2.95) | NR |
|  |  | Baseline CKD |  |  | 1.62 (1.22-2.16) | NR |
| Saito 2017 ([101](#_ENREF_101)) | Patients with stage 3-5 non-dialysis dependent CKD | Male | sK+ ≥5.5 mEq/L | Odds ratio (Univariable analysis) | 1.06 (0.72-1.57) | NR |
|  |  | Age: 20-64 years vs 65-79 years |  |  | 0.93 (0.53-1.63) | NR |
|  |  | Age: 20-64 years vs >80 years |  |  | 0.88 (0.5-1.56) | NR |
|  |  | CKD stage 3a vs CKD stage 3b |  |  | 1.98 (1.28-3.08) | NR |
|  |  | CKD stage 3a vs CKD stage 4-5 |  |  | 3.57 (2.15-5.94) | NR |
|  |  | DM |  |  | 1.06 (0.72-1.55) | NR |
|  |  | Combined use of RAASi |  |  | 2.41 (1.58-3.69) | NR |
|  |  | Malignancy |  |  | 1.49 (0.86-2.57) | NR |
|  |  | NSAIDs |  |  | 0.8 (0.34-1.9) | NR |
|  |  | β-blockers |  |  | 1.26 (0.86-1.84) | NR |
|  |  | K+ binders |  |  | 1.27 (0.37-4.39) | NR |
|  |  | K+ salt substitutes |  |  | 0.84 (0.33-2.18) | NR |
|  |  | Loop and/or thiazide diuretics |  |  | 2.17 (1.48-3.19) | NR |
|  |  | Baseline sK+ (unit: 1.0 mEq/L) |  |  | 1.66 (1.09-2.52) | NR |
|  |  | Ejection fraction ≤35% |  |  | 1.13 (0.62-2.06) | NR |
|  |  | Male |  | Odds ratio (Multivariable analysis Model 1) | 1.11 (0.73-1.7) | NR |
|  |  | Age: 20-64 years vs 65-79 years |  |  | 0.82 (0.45-1.48) | NR |
|  |  | Age: 20-64 years vs >80 years |  |  | 0.78 (0.42-1.44) | NR |
|  |  | CKD stage 3a vs CKD stage 3b |  |  | 1.88 (1.2-2.97) | NR |
|  |  | CKD stage 3a vs CKD stage 4-5 |  |  | 3.4 (1.99-5.81) | NR |
|  |  | DM |  |  | 1.01 (0.67-1.53) | NR |
|  |  | Combined use of RAASi |  |  | 1.92 (1.19-3.1) | NR |
|  |  | Malignancy |  |  | 2.1 (1.14-3.86) | NR |
|  |  | NSAIDs |  |  | 0.83 (0.33-2.08) | NR |
|  |  | β-blockers |  |  | 1.09 (0.71-1.67) | NR |
|  |  | K+ binders |  |  | 1.01 (0.28-3.65) | NR |
|  |  | K+ salt substitutes |  |  | 1.02 (0.38-2.73) | NR |
|  |  | Loop and/or thiazide diuretics |  |  | 1.8 (1.16-2.79) | NR |
|  |  | Baseline sK+ (unit: 1.0 mEq/L) |  |  | 1.91 (1.23-2.97) | NR |
|  |  | Male |  | Odds ratio (Multivariable analysis Model 2) | 1.38 (0.84-2.27) | NR |
|  |  | Age: 20-64 years vs 65-79 years |  |  | 0.74 (0.37-1.46) | NR |
|  |  | Age: 20-64 years vs >80 years |  |  | 0.81 (0.4-1.63) | NR |
|  |  | CKD stage 3a vs CKD stage 3b |  |  | 2.24 (1.33-3.77) | NR |
|  |  | CKD stage 3a vs CKD stage 4-5 |  |  | 3.73 (2-6.94) | NR |
|  |  | DM |  |  | 1.22 (0.76-1.97) | NR |
|  |  | Combined use of RAASi |  |  | 1.85 (1.08-3.19) | NR |
|  |  | Malignancy |  |  | 2.6 (1.17-5.81) | NR |
|  |  | NSAIDs |  |  | 0.69 (0.2-2.41) | NR |
|  |  | β-blockers |  |  | 1.11 (0.67-1.82) | NR |
|  |  | K+ binders |  |  | 1.21 (0.31-4.69) | NR |
|  |  | K+ salt substitutes |  |  | 0.99 (0.36-2.7) | NR |
|  |  | Loop and/or thiazide diuretics |  |  | 1.46 (0.88-2.42) | NR |
|  |  | Baseline sK+ (unit: 1.0 mEq/L) |  |  | 1.67 (1.02-2.74) | NR |
|  |  | Ejection fraction ≤35% |  |  | 0.99 (0.51-1.91) | NR |
| Gwoo 2014 ([81](#_ENREF_81)) | Patients with stage 2-4 CKD + HT | Age >65 years | sK+ ≥5.0 mEq/L | Odds ratio | 1.17 (0.78-1.75) | p=0.452 |
|  |  | CKD |  |  | 3.03 (1.84-4.98) | p=0.001 |
|  |  | Males |  |  | 1.26 (0.84-1.88) | p=0.261 |
|  |  | Baseline sK+ >5.0 mEq/L |  |  | 1.76 (0.95-3.24) | p=0.071 |
|  |  | >30% reduction in eGFR |  |  | 3.48 (2.14-5.64) | p=0.001 |
|  |  | DM |  |  | 1.9 (1.23-2.94) | p=0.004 |
| Hirai 2018 ([72](#_ENREF_72)) | Patients with stage 3-5 CKD and unknown stage CKD | Age >65 years | sK+ >5.5 mEq/L | Odds ratio (Univariate logistic regression analysis) | 1.49 (1.08-2.06) | p<0.05 |
|  |  | Male gender |  |  | 1.27 (0.96-1.68) | p<0.10 |
|  |  | Height (per cm) |  |  | 1 (0.99-1.02) | p=0.57 |
|  |  | Body weight (per kg) |  |  | 0.98 (0.97-0.99) | p<0.01 |
|  |  | BMI (per kg/m2) |  |  | 0.92 (0.89-0.95) | p<0.01 |
|  |  | DM |  |  | 1.61 (1.22-2.12) | p<0.01 |
|  |  | HF |  |  | 1.21 (0.83-1.76) | p=0.32 |
|  |  | Haemodialysis |  |  | 5.32 (3.45-8.22) | p<0.01 |
|  |  | History of treatment for hyperkalaemia |  |  | 10.6 (7.17-15.76) | p<0.01 |
|  |  | ALT |  |  | 0.99 (0.99-1) | p<0.10 |
|  |  | AST |  |  | 0.99 (0.99-1) | p<0.05 |
|  |  | Serum sodium <135 mEq/L |  |  | 2.63 (1.83-3.78) | p<0.01 |
|  |  | eGFR <30 mL/min/1.73 m2 |  |  | 6.99 (5.28-9.26) | p<0.01 |
|  |  | Hyperkalaemic diuretics |  |  | 2.12 (1.62-2.79) | p<0.01 |
|  |  | Hyperkalaemia-inducing medications |  |  | 1.29 (0.95-1.74) | p<0.10 |
|  |  | β-blockers |  |  | 1.17 (0.87-1.57) | p=0.3 |
|  |  | Age >65 years |  | Odds ratio (Multivariable logistic regression analysis) | 1.49 (1.03-2.16) | p=0.03 |
|  |  | BMI (per kg/m2) |  |  | 0.94 (0.91-0.98) | p<0.01 |
|  |  | DM |  |  | 1.66 (1.21-2.27) | p<0.01 |
|  |  | History of treatment for hyperkalaemia |  |  | 4.94 (3.14-7.76) | p<0.01 |
|  |  | Serum sodium <135 mEq/L |  |  | 2.02 (1.35-3.04) | p<0.01 |
|  |  | eGFR <30 mL/min/1.73 m2 |  |  | 4.69 (3.38-6.49) | p<0.01 |
|  |  | Hyperkalaemia-inducing medications |  |  | 1.88 (1.34-2.63) | p<0.01 |
| Jun 2019 ([78](#_ENREF_78)) | Patients with stage 3-5 CKD | Sex (female vs male) | sK+ >6.0 mmol/L or coded or free-text recorded diagnosis of hyperkalaemia | Hazard ratio (Univariate analysis) | 0.68 (0.61-0.73) | p<0.001 |
|  |  | Age (per 1 year increase) |  |  | 0.99 (0.99-0.99) | p=0.032 |
|  |  | Smoking status: Non-smoker (reference) |  |  | Reference | p<0.001 |
|  |  | Smoking status: Previous smoker |  |  | 1.31 (1.19-1.44) |  |
|  |  | Smoking status: Smoker |  |  | 1.55 (1.26-1.91) |  |
|  |  | eGFR category: 45-59 (reference) |  |  | Reference | p<0.001 |
|  |  | eGFR category: 30-44 |  |  | 1.6 (1.44-1.77) |  |
|  |  | eGFR category: 15-29 |  |  | 3.08 (2.72-3.48) |  |
|  |  | eGFR category: <15 |  |  | 5.14 (4.09-6.46) |  |
|  |  | sK+ (per 0.1 mmol/L increase) |  |  | 1.14 (1.13-1.15) | p<0.001 |
|  |  | Atrial fibrillation (yes vs no) |  |  | 1.22 (1.11-1.36) | p<0.001 |
|  |  | Cardiovascular disease (yes vs no) |  |  | 1.6 (1.45-1.76) | p<0.001 |
|  |  | DM (Yes vs No) |  |  | 1.62 (1.48-1.77) | p<0.001 |
|  |  | HF (Yes vs No) |  |  | 1.7 (1.55-1.87) | p<0.001 |
|  |  | Left ventricular hypertrophy (yes vs no) |  |  | 0.99 (0.69-1.42) | p=0.962 |
|  |  | Stroke (Yes vs No) |  |  | 0.99 (0.86-1.13) | p=0.855 |
|  |  | ACEi (reference) |  |  | Reference | p<0.001 |
|  |  | ARB |  |  | 0.81 (0.72-0.9) |  |
|  |  | Aldosterone antagonist |  |  | 1.5 (1.29-1.75) |  |
|  |  | ACEi + other combinations |  |  | 1 (0.81-1.24) |  |
|  |  | ARB + other combinations |  |  | 0.61 (0.52-0.7) |  |
|  |  | Sex (female vs male) |  | Hazard ratio (Multivariable analysis) | 0.76 (0.67-0.86) | p<0.001 |
|  |  | Age (per 1 year increase) |  |  | 0.99 (0.98-0.99) | p=0.044 |
|  |  | Smoking status: Non-smoker (reference) |  |  | Reference | p=0.025 |
|  |  | Smoking status: Previous smoker |  |  | 1.12 (0.99-1.27) |  |
|  |  | Smoking status: Smoker |  |  | 1.38 (1.05-1.8) |  |
|  |  | eGFR category: 45-59 (reference) |  |  | Reference | p<0.001 |
|  |  | eGFR category: 30-44 |  |  | 1.42 (1.25-1.63) |  |
|  |  | eGFR category: 15-29 |  |  | 2.45 (2.09-2.86) |  |
|  |  | eGFR category: <15 |  |  | 3.73 (2.71-5.13) |  |
|  |  | sK+ (per 0.1 mmol/L increase) |  |  | 1.13 (1.12-1.14) | p<0.001 |
|  |  | Atrial fibrillation (Yes vs No) |  |  | 1.1 (0.96-1.26) | p=0.183 |
|  |  | Cardiovascular disease (yes vs no) |  |  | 1.12 (0.96-1.3) | p=0.153 |
|  |  | DM (Yes vs No) |  |  | 1.26 (1.12-1.41) | p<0.001 |
|  |  | HF (Yes vs No) |  |  | 1.38 (1.19-1.6) | p<0.001 |
|  |  | Left ventricular hypertrophy (Yes vs No) |  |  | 0.86 (0.54-1.36) | p=0.52 |
|  |  | Stroke (Yes vs No) |  |  | 0.93 (0.78-1.1) | p=0.407 |
|  |  | ACEi (reference) |  |  | Reference | p<0.001 |
|  |  | ARB |  |  | 0.95 (0.83-1.09) |  |
|  |  | Aldosterone antagonist |  |  | 1.53 (1.25-1.87) |  |
|  |  | ACEi + other combinations |  |  | 1.2 (0.93-1.57) |  |
|  |  | ARB + other combinations |  |  | 0.81 (0.67-0.98) |  |
| Perez-Navarro 2023 ([55](#_ENREF_55)) | Patients with stage 3a CKD | DM | sK >5.0 mmol/L | Odds ratio (95% CI) | 7.5 (1.6-34.7) | NR |
|  |  | Antihypertensives (ACEi) |  |  | 3.7 (12-11.2) | NR |
|  |  | HT |  |  | 3.5 (1.1-10.9) | NR |
|  | Patients with stage 3b CKD | DM |  |  | 2.06 (1.1-3.8) | NR |
|  |  | Antihypertensives (IECAS) |  |  | 2.5 (1.04-6.2) | p=0.04 (reference group not specified) |
|  |  | Glucose > 100 ml/dL |  |  | 2.01 (1.04-3.8) | NR |
|  | Patients with stage 4 CKD | Antihypertensives (ARBs) |  |  | 3.9 (1.3-11.3) | NR |
|  | Patients with stage 5 CKD | Gender: male |  |  | 1.6 (1.13-2.45) | NR |
|  |  | Being on hemodialysis |  |  | 1.8 (1.2-2.7) | NR |
| Gaol 2024 ([139](#_ENREF_139)) | Patients with CKD undergoing hemodialysis | Age | NR | Odds ratio (95% CI) | 0.979 (0.04-0.99) | p=0.046 |
|  |  | High levels of serum phosphate | NR |  | 0.68 (0.54-0.85) | p=0.001 |

**Abbreviations:** β: beta; ACEi: angiotensin-converting enzyme inhibitors; ACR: albumin-to-creatinine ratio; AFib: atrial fibrillation; AIDS: acquired immunodeficiency virus; ALT: alanine transaminase; ARBs: angiotensin-receptor blockers; AST: aspartate aminotransferase; ATSI: aboriginal or Torres Strait Islander; BMI: body mass index; BP: blood pressure; CAD: coronary artery disease; CCB: calcium channel blocker; CI: confidence interval; CKD: chronic kidney disease; CO2: carbon dioxide; COPD: chronic obstructive pulmonary disease; DM: diabetes mellitus; eGFR: estimated glomerular filtration rate; ESRD: end stage renal disease; g/dl: gram per decilitre; HF: heart failure; HIV: human immunodeficiency virus; HT: hypertension; ICD-10: International Classification of Diseases, Tenth Revision; ITT: Intention-to-treat; K+: potassium; kg/m2: kilogram per square metre; LDL: low density lipoprotein; m2: square metre; mEq/L: milliequivalents per litre; mg/dL: milligrams per decilitre; mg/mmol: milligrams per millimoles; mGFR: measured glomerular filtration rate; MI: myocardial infarction; mL/min: millilitres per minute; mmHg: millimetre of mercury; mmol/L: millimoles per litre; MRA: mineralocorticoid-receptor antagonist; NA: not applicable; Na-Cl: sodium chloride; NR: not reported; NSAID: non-steroidal anti-inflammatory drugs; PAD: peripheral artery disease; PVD: peripheral vascular disease; RAASi: renin-angiotensin-aldosterone system inhibitors; sK+: serum potassium; UP/Cr: urinary protein to creatinine ratio; vs: versus.

Table S16. Prevalence of hyper-, hypo- and normokalaemia in CKD patients

| Study | Country | Study setting/data source | Time Period | Population type | Population description | Sample size | Hyperkalaemia | | Hypokalaemia | | Normokalaemia | |  |
| --- | --- | --- | --- | --- | --- | --- | --- | --- | --- | --- | --- | --- | --- |
|  |  |  |  |  |  |  | **Definition** | **Prevalence rate*** | **Definition** | **Prevalence rate*** | **Definition** | **Prevalence rate*** |  |
| Li 2023a ([65](#_ENREF_65)) | China | Patients hospitalised at Baoding No. 1, Central Hospital of Hebei Medical University | 2020-2021 | Overall | Patients with stage 4-5 CKD + DM | 270 | sK+ >5.5 mmol/L | 44.4% | NR | NR | sK+ ≤5.5 mmol/L | 55.6% |  |
|  |  |  |  | Subgroup | Patients with stage 4-5 CKD + DM + HF | 82 |  | 48.6% | NR | NR |  | 52.2% |  |
| Wang 2023 ([89](#_ENREF_89)) | China | Patients referred in Peking University First Hospital | 2010-2020 | Overall | Patients with stage 1-4 CKD | 1,078 | sK+ ≥5.5 mmol/L | Instantaneous hyperkalaemia: 16.1%  Persistent hyperkalaemia: 21.1% | NR | NR | NR | 62.8% |  |
| Neuenschwander 2023 ([116](#_ENREF_116)) | USA | Medicare FFS claims database | 2017-2019 | Overall | Patients with stage 3-4 CKD | 795,543 | ICD-10 diagnostic code of E87.5 in any claim position or Medicare Part D fill for oral K+ binder use within 14 days prior or during long-term care stay | 18.3% | NR | NR | No evidence of hyperkalaemia within 14 days prior to long-term care stay or during long-term care stay | 81.7% |  |
| Calabrese 2022 ([60](#_ENREF_60)) | Italy | Patients admitted at the Unit of Nephrology and Dialysis of the University Hospital of Messina | 1st Apr 2018-31st Dec 2018 | Overall | Patients with stage 1-5 or A1-A3 CKD or with other anatomic or urine sediment abnormalities according to the KDIGO 2012 Clinical Practice Guideline for the Evaluation and Management of CKD | 270 | sK+ >5.1 mEq/L | 33.3% | NR | NR | sK+ ≤5.1 mEq/L | 66.7% |  |
|  |  |  |  | Subgroup | Patients with stage 1-5 or A1-A3 CKD or with other anatomic or urine sediment abnormalities according to the KDIGO 2012 Clinical Practice Guideline for the Evaluation and Management of CKD + DM | 103 |  | 32.0% | NR | NR |  | 68.0% |  |
|  |  |  |  | Subgroup | Patients with stage 1-5 or A1-A3 CKD or with other anatomic or urine sediment abnormalities according to the KDIGO 2012 Clinical Practice Guideline for the Evaluation and Management of CKD + HF | 7 |  | 57.1% | NR | NR |  | 42.9% |  |
|  |  |  |  | Subgroup | CKD patients with eGFR 6-30 mL/min/1.73 m2 | 121 |  | 38.0% | NR | NR |  | 62.0% |  |
|  |  |  |  | Subgroup | CKD patients with eGFR 6-30 mL/min/1.73 m2 + DM | 52 |  | 28.8% | NR | NR |  | 71.2% |  |
|  |  |  |  | Subgroup | CKD patients with eGFR 6-30 mL/min/1.73 m2 + HF | 5 |  | 80.0% | NR | NR |  | 20.0% |  |
| Zhang 2022a ([36](#_ENREF_36)) | China | Data collected from the UEBMI claims of Tianjin | 2012-2016 | Overall | Patients with CKD having ≥1 inpatient or outpatient visit (Before propensity score matching) | 26,996 | sK+ >5.0 mmol/L | 3.8% | NR | NR | NR | 96.2% |  |
|  |  |  |  | Subgroup | Patients with CKD having ≥1 inpatient or outpatient visit + HT (Before propensity score matching) | 21,204 |  | 3.8% | NR | NR |  | 96.2% |  |
|  |  |  |  | Subgroup | Patients with CKD having ≥1 inpatient or outpatient visit + DM (Before propensity score matching) | 14,503 |  | 3.1% | NR | NR |  | 96.9% |  |
|  |  |  |  | Subgroup | Patients with CKD having ≥1 inpatient or outpatient visit + HF (Before propensity score matching) | 1,453 |  | 14.5% | NR | NR |  | 85.5% |  |
| DISCOVER CKD  James 2021 ([40](#_ENREF_40)) | Multinational (Japan, UK, USA) | USA TriNetX hospital-electronic medical records, UK CPR linked to hospital data, USA DOPPS and JMDV databases | Jan 2008- Mar 2020 | Overall | Patients with stages 3-5 CKD including RRT or two eGFR measures <60 mL/min/1.73 m2 between 90-730 days apart | 1,797,791 | sK+ >5.0 mmol/L | 7.0% | NR | NR | sK+ <5.0 mmol/L | 93.0% |  |
|  |  |  |  | Subgroup | Patients with stages 3-5 CKD including RRT or two eGFR measures <60 mL/min/1.73 m2 between 90-730 days apart + DM | NR |  | 44.4% | NR | NR |  | 20.6% |  |
|  |  |  |  | Subgroup | Patients with stages 3-5 CKD including RRT or two eGFR measures <60 mL/min/1.73 m2 between 90-730 days apart + HF | NR |  | 27.7% | NR | NR |  | 9.5% |  |
|  |  |  |  | Subgroup | Patients with stages 3-5 CKD including RRT or two eGFR measures <60 mL/min/1.73 m2 between 90-730 days apart + DM + HF | NR |  | 15.3% | NR | NR |  | 3.8% |  |
| Shang 2022 ([35](#_ENREF_35)) | China | Data used from patients hospitalised with HF, integrating EMR from the PhysioNet restricted health data database and external outcome data | 8th Jun 2020 | Overall | Patients with CKD + HF | 224 | sK+ >4.7 mmol/L | 23.7% | sK+ <3.5 mmol/L | 11.6% | sK+ 3.5-4.7 mmol/L | 64.7% |  |
| Kohsaka 2021 ([86](#_ENREF_86)) | Japan | Data obtained using a Japanese hospital claims registry, Medical Data Vision (Hospital based cohort study) | 1st Apr 2008-30th Sept 2018 | Overall | Patients with CKD based on ICD-10 codes or an average eGFR <60 mL/min/1.73 m2 | 28,031 | At least 2 sK+ readings of ≥5.1 mmol/L within a 12-month interval | 57.6% | NR | NR | Without any record of sK+ levels of ≤3.5 mmol/L and ≥5.1 mmol/L | 42.4% |  |
|  |  |  |  | Subgroup | Patients with CKD Stage 1 | 1,286 |  | 15.6% | NR | NR |  | 84.4% |  |
|  |  |  |  | Subgroup | Patients with CKD Stage 2 | 4,961 |  | 23.0% | NR | NR |  | 77.0% |  |
|  |  |  |  | Subgroup | Patients with CKD Stage 3 | 7,307 |  | 36.3% | NR | NR |  | 93.8% |  |
|  |  |  |  | Subgroup | Patients with CKD Stage 3b | 5,943 |  | 69.5% | NR | NR |  | 24.8% |  |
|  |  |  |  | Subgroup | Patients with CKD Stage 4 | 5,124 |  | 92.6% | NR | NR |  | 6.4% |  |
|  |  |  |  | Subgroup | Patients with CKD Stage 5 | 3,410 |  | 95.7% | NR | NR |  | 2.8% |  |
|  |  |  |  | Subgroup | Patients with CKD having ≥1 inpatient or outpatient visit + DM | 13,238 |  | 58.3% | NR | NR |  | 41.7% |  |
| Grandy 2021 ([37](#_ENREF_37)) | China, France, Germany, Italy, Spain, USA | Adelphi Real World CKD Disease Specific Programme™ | Jul 2015-Oct 2015 and Oct 2017-Feb 2018 | Overall | Patients with stage 3-4 non-dialysis dependent CKD | 1,149 | sK+ >5.0 mmol/L | 18.8% | NR | NR | sK+ 3.5 to 5.0 mmol/L | 81.2% |  |
|  | USA |  |  | Subgroup |  | 376 |  | 17.0% | NR | NR |  | 83.0% |  |
|  | EU5 |  |  | Subgroup |  | 490 |  | 20.0% | NR | NR |  | 80.0% |  |
|  | China |  |  | Subgroup |  | 283 |  | 19.1% | NR | NR |  | 80.9% |  |
|  | China, France, Germany, Italy, Spain, USA |  |  | Subgroup | Patients with stage 3-4 non-dialysis dependent CKD + HT | 874 |  | 19.3% | NR | NR |  | 80.7% |  |
|  |  |  |  | Subgroup | Patients with stage 3-4 non-dialysis dependent CKD + DM | 451 |  | 21.5% | NR | NR |  | 78.5% |  |
| Brookes 2021 ([110](#_ENREF_110)) | Australia | Study used Austin Health EMR data extracted from the DARE Centre | 1st Jan 2014-31st Dec 2018 | Overall | Patients with stage 1-5 non-dialysis dependent CKD | 11,156 | sK+ ≥6.0 mmol/L | 2.7% | sK+ <3.5 mmol/L | 2.8% | sK+ 5.0 to 5.4 mmol/L | 13.5% |  |
|  |  |  |  |  |  |  | sK+ 5.5 to 5.9 mmol/L | 4.2% |  |  | sK+ 4.0 to 4.9 mmol/L | 64.9% |  |
|  |  |  |  |  |  |  | NR | NR |  |  | sK+ 3.5 to 3.9 mmol/L | 12.0% |  |
|  |  |  |  | Subgroup | Patients with stage 1 non-dialysis dependent CKD | 80 | sK+ ≥6.0 mmol/L | 0.3% |  | 1.6% | sK+ 5.0 to 5.4 mmol/L | 0.4% |  |
|  |  |  |  |  |  |  | sK+ 5.5 to 5.9 mmol/L | 0.2% |  |  | sK+ 4.0 to 4.9 mmol/L | 0.7% |  |
|  |  |  |  |  |  |  | NR | NR |  |  | sK+ 3.5 to 3.9 mmol/L | 1.0% |  |
|  |  |  |  | Subgroup | Patients with stage 2 non-dialysis dependent CKD | 1,569 | sK+ ≥6.0 mmol/L | 3.4% |  | 19.2% | sK+ 5.0 to 5.4 mmol/L | 8.3% |  |
|  |  |  |  |  |  |  | sK+ 5.5 to 5.9 mmol/L | 3.8% |  |  | sK+ 4.0 to 4.9 mmol/L | 15.5% |  |
|  |  |  |  |  |  |  | NR | NR |  |  | sK+ 3.5 to 3.9 mmol/L | 17.7% |  |
|  |  |  |  | Subgroup | Patients with stage 3a non-dialysis dependent CKD | 4,395 | sK+ ≥6.0 mmol/L | 10.8% |  | 34.9% | sK+ 5.0 to 5.4 mmol/L | 31.6% |  |
|  |  |  |  |  |  |  | sK+ 5.5 to 5.9 mmol/L | 17.6% |  |  | sK+ 4.0 to 4.9 mmol/L | 43.2% |  |
|  |  |  |  |  |  |  | NR | NR |  |  | sK+ 3.5 to 3.9 mmol/L | 43.0% |  |
|  |  |  |  | Subgroup | Patients with stage 3b non-dialysis dependent CKD | 3,196 | sK+ ≥6.0 mmol/L | 27.6% |  | 26.7% | sK+ 5.0 to 5.4 mmol/L | 31.7% |  |
|  |  |  |  |  |  |  | sK+ 5.5 to 5.9 mmol/L | 38.3% |  |  | sK+ 4.0 to 4.9 mmol/L | 27.9% |  |
|  |  |  |  |  |  |  | NR | NR |  |  | sK+ 3.5 to 3.9 mmol/L | 26.8% |  |
|  |  |  |  | Subgroup | Patients with stage 4 non-dialysis dependent CKD | 1,501 | sK+ ≥6.0 mmol/L | 32.0% |  | 13.0% | sK+ 5.0 to 5.4 mmol/L | 21.9% |  |
|  |  |  |  |  |  |  | sK+ 5.5 to 5.9 mmol/L | 28.5% |  |  | sK+ 4.0 to 4.9 mmol/L | 10.7% |  |
|  |  |  |  |  |  |  | NR | NR |  |  | sK+ 3.5 to 3.9 mmol/L | 9.8% |  |
|  |  |  |  | Subgroup | Patients with stage 5 non-dialysis dependent CKD | 415 | sK+ ≥6.0 mmol/L | 25.9% |  | 4.6% | sK+ 5.0 to 5.4 mmol/L | 6.1% |  |
|  |  |  |  |  |  |  | sK+ 5.5 to 5.9 mmol/L | 11.6% |  |  | sK+ 4.0 to 4.9 mmol/L | 2.1% |  |
|  |  |  |  |  |  |  | NR | NR |  |  | sK+ 3.5 to 3.9 mmol/L | 1.8% |  |
|  |  |  |  | Subgroup | Patients with stage 1-5 non-dialysis dependent CKD + HF | 1,200 | sK+ ≥6.0 mmol/L | 12.1% |  | 9.8% | sK+ 5.0 to 5.4 mmol/L | 12.0% |  |
|  |  |  |  |  |  |  | sK+ 5.5 to 5.9 mmol/L | 12.7% |  |  | sK+ 4.0 to 4.9 mmol/L | 10.6% |  |
|  |  |  |  |  |  |  | NR | NR |  |  | sK+ 3.5 to 3.9 mmol/L | 9.7% |  |
|  |  |  |  | Subgroup | Patients with stage 1-5 non-dialysis dependent CKD + DM | 3,566 | sK+ ≥6.0 mmol/L | 56.6% |  | 24.1% | sK+ 5.0 to 5.4 mmol/L | 39.2% |  |
|  |  |  |  |  |  |  | sK+ 5.5 to 5.9 mmol/L | 45.5% |  |  | sK+ 4.0 to 4.9 mmol/L | 30.5% |  |
|  |  |  |  |  |  |  | NR | NR |  |  | sK+ 3.5 to 3.9 mmol/L | 23.3% |  |
| Jime´nez-Marrero 2021 ([44](#_ENREF_44)) | Spain | Administrative, hospital and primary care databases | 1st Jan 2016 | Overall | Patients with CKD | 7,242 | sK+ >5.0 mEq/L | 5.7% | sK+ <3.5 mEq/L | 1.1 | sK+ ≥3.5 and ≤5 mEq/L | 93.2% |  |
|  |  |  |  | Subgroup | Patients with CKD who were using at least one RAASI medication between 1st Oct 2015-1st Jan 2016 | 4,418 |  | 6.0 |  | 0.9 |  | 93.2% |  |
| Fukushima CKD Cohort  Tanaka 2021 ([80](#_ENREF_80)) | Japan | Fukushima Medical University Hospital cohort | 2016 | Overall | Pre-dialysis patients with CKD with eGFR <60 mL/min/1.73 m2 | 1,330 | sK+ ≥5.0 mmol/L | 10.6% | sK+ <4.0 mmol/L | 16.3% | sK+ 4.0 to 4.4 mmol/L | 40.5% |  |
|  |  |  |  |  |  |  |  |  |  |  | sK+ 4.5 to 4.9 mmol/L | 32.6% |  |
|  |  |  |  | Subgroup | Pre-dialysis patients with CKD with eGFR <60 mL/min/1.73 m2 + DM | 637 |  | 53.2% |  | 35.9% | sK+ 4.0 to 4.4 mmol/L | 47.5% |  |
|  |  |  |  |  |  |  |  |  |  |  | sK+ 4.5 to 4.9 mmol/L | 52.7% |  |
|  |  |  |  | Subgroup | Pre-dialysis patients with CKD with eGFR <60 mL/min/1.73 m2 + HT | 1,132 |  | 90.1% |  | 78.8% | sK+ 4.0 to 4.4 mmol/L | 85.2% |  |
|  |  |  |  |  |  |  |  |  |  |  | sK+ 4.5 to 4.9 mmol/L | 86.6% |  |
|  |  |  |  | Subgroup | Pre-dialysis patients with CKD stage 1 and 2 + HT | NR |  | 2.0% |  | 24.3% | sK+ 4.0 to 4.4 mmol/L | 50.7% |  |
|  |  |  |  |  |  |  |  |  |  |  | sK+ 4.5 to 4.9 mmol/L | 23.0% |  |
|  |  |  |  | Subgroup | Pre-dialysis patients with CKD stage 3a CKD + HT | NR |  | 6.8% |  | 16.4% | sK+ 4.0 to 4.4 mmol/L | 40.7% |  |
|  |  |  |  |  |  |  |  |  |  |  | sK+ 4.5 to 4.9 mmol/L | 36.1% |  |
|  |  |  |  | Subgroup | Pre-dialysis patients with CKD stage 3b + HT | NR |  | 15.8% |  | 11.1% | sK+ 4.0 to 4.4 mmol/L | 36.8% |  |
|  |  |  |  |  |  |  |  |  |  |  | sK+ 4.5 to 4.9 mmol/L | 36.3% |  |
|  |  |  |  | Subgroup | Pre-dialysis patients with CKD stage 4 + HT | NR |  | 27.9% |  | 6.8% | sK+ 4.0 to 4.4 mmol/L | 26.4% |  |
|  |  |  |  |  |  |  |  |  |  |  | sK+ 4.5 to 4.9 mmol/L | 38.9% |  |
|  |  |  |  | Subgroup | Pre-dialysis patients with CKD s stage 5 + HT | NR |  | 37.1% |  | 15.8% | sK+ 4.0 to 4.4 mmol/L | 16.0% |  |
|  |  |  |  |  |  |  |  |  |  |  | sK+ 4.5 to 4.9 mmol/L | 31.1% |  |
| Sharma 2020 ([38](#_ENREF_38)) | USA | Data included medical and pharmacy claims and lab test results of deidentified individuals continuously enrolled in a fully insured commercial or Medicare Advantage plan operated by a large national health plan | 2017 | Overall | Patients with stage 1-5 CKD | 435,512 | 1. ≥2 sK+ >5.0 mmol/L on different dates (Logical Observation Identifiers Names and Codes) 2. ≥2 claims with principal or secondary diagnosis of hyperkalaemia (ICD-10) 3. 1 claim with hyperkalaemia and 1 sK+ >5.0 mmol/L 4. ≥1 dispensed prescription for SPS or patiromer | 1.4% | NR | NR | NR | 98.6% |  |
|  |  |  |  | Subgroup | Patients with stage 1-5 CKD + HT | 278,336 |  | 1.9% | NR | NR | NR | 98.0% |  |
|  |  |  |  | Subgroup | Patients with stage 1-5 CKD + DM | 110,881 |  | 2.8% | NR | NR | NR | 99.0% |  |
| Kanda 2020 ([87](#_ENREF_87)) | Japan | Japanese hospital claims registry, Medical Data Vision | 1st Apr 2008-30th Sept 2018 | Overall | Patients with stage 1-5 CKD | 31,420 | sK+ ≥5.1 mmol/L | 51.3% | NR | NR | sK+ 3.5 mmol/L to 5.1 mmol/L | 48.7% |  |
|  |  |  |  | Subgroup | Patients with stage 1-5 CKD + DM | 14,610 |  | 52.8% | NR | NR |  | 47.2% |  |
|  |  |  |  | Subgroup | Patients with stage 1-5 CKD + HT | 22,111 |  | 55.2% | NR | NR |  | 44.8% |  |
|  |  |  |  | Subgroup | Patients with stage 1 CKD | 1,373 |  | 14.6% | NR | NR |  | 85.4% |  |
|  |  |  |  |  | Patients with stage 2 CKD | 5,654 |  | 20.2% | NR | NR |  | 79.8% |  |
|  |  |  |  |  | Patients with stage 3a CKD | 9,241 |  | 28.7% | NR | NR |  | 71.3% |  |
|  |  |  |  |  | Patients with stage 3b CKD | 6,473 |  | 63.8% | NR | NR |  | 36.2% |  |
|  |  |  |  |  | Patients with stage 4 CKD | 5,175 |  | 91.7% | NR | NR |  | 8.3% |  |
|  |  |  |  |  | Patients with stage 5 CKD | 3,504 |  | 93.2% | NR | NR |  | 6.8% |  |
| Jimenez-Marrero 2020 ([39](#_ENREF_39)) | Spain | Population-based healthcare database of the Catalan Institute of Health | 2016 | Overall | Patients with CKD + HF | 5,610 | sK+ >5.0 mEq/L | 23.3% | sK+ <3.5 mEq/L | 1.9% | NR | NR |  |
|  |  |  | 2017 |  |  | 6,538 |  | 24.6% |  | 2.1% | NR | NR |  |
| Furuland 2018 ([108](#_ENREF_108)) | UK | CPRD and linked Hospital Episode Statistics databases | 1st Jan 2006-31st Dec 2015 | Overall | Pre-dialysis patients with stage 3-5 CKD | 143,635 | sK+ 5.0 to <5.5 mmol/L | 12.8% | sK+ <3.5 mmol/L | 2.5% | sK+ 3.5 to <4.0 mmol/L | 12.3% |  |
|  |  |  |  |  |  |  | sK+ 5.5 to <6.0 mmol/L | 3.0% |  |  | sK+ 4.0 to <4.5 mmol/L | 34.9% |  |
|  |  |  |  |  |  |  | sK+ ≥6.0 mmol/L | 0.7% |  |  | sK+ 4.5 to <5.0 mmol/L | 33.8% |  |
| NephroTestWagner 2017 ([43](#_ENREF_43)) | France | Hospital-based cohort study | Jan 2000-Dec 2012 | Overall | Patients with stage 1-5 non-dialysis-dependent CKD | 2,078 | sK+ >5.0 mmol/L | 8.3% | sK+ <3.6 mmol/L | 27.2% | sK+ 4-5 mmol/L | 64.5% |  |
|  |  |  |  | Subgroup | Patients with stage 1-5 non-dialysis-dependent CKD + DM | 621 |  | 37.2% |  | 25.6% |  | 30.9% |  |
| Collins 2017 ([115](#_ENREF_115)) | USA | De-identified EMR data on ~7 million patients collected from multiple USA integrated health delivery networks | 2007-2012 | Subgroup | Patients with stage 3a CKD | 48,749 | sK+ 5.0 to <5.5 mEq/L | 7.7% | sK+ 2.5 - <3. 0 mEq/L | 0.2% | sK+ 4.0 to <4.5 mEq/L | 39.6% |  |
|  |  |  |  |  |  |  | sK+ 5.5 to <6.0 mEq/L | 1.1% | sK+ 3.0 - <3.5 mEq/L | 3.5% |  |  |  |
|  |  |  |  |  |  |  | sK+ 6.0 to <6.5 mEq/L | 0.2% | sK+ 3.5 - 4.0 mEq/L | 20.6% | sK+ 4.5 to <5.0 mEq/L | 27.0% |  |
|  |  |  |  |  |  |  | sK+ 6.5 to 8.0 mEq/L | 0.1% | NR | NR |  |  |  |
|  |  |  |  | Subgroup | Patients with stage 3b CKD | 30,455 | sK+ 5.0 to <5.5 mEq/L | 10.0% | sK+ 2.5 - <3. 0 mEq/L | 0.3% | sK+ 4.0 to <4.5 mEq/L | 37.0% |  |
|  |  |  |  |  |  |  | sK+ 5.5 to <6.0 mEq/L | 2.0% | sK+ 3.0 - <3.5 mEq/L | 3.3% |  |  |  |
|  |  |  |  |  |  |  | sK+ 6.0 to <6.5 mEq/L | 0.3% | sK+ 3.5 - 4.0 mEq/L | 19.3% | sK+ 4.5 to <5.0 mEq/L | 27.7% |  |
|  |  |  |  |  |  |  | sK+ 6.5 to 8.0 mEq/L | 0.2% | NR | NR |  |  |  |
|  |  |  |  | Subgroup | Patients with stage 4 CKD | 16,083 | sK+ 5.0 to <5.5 mEq/L | 12.2% | sK+ 2.5 - <3. 0 mEq/L | 0.3% | sK+ 4.0 to <4.5 mEq/L | 32.5% |  |
|  |  |  |  |  |  |  | sK+ 5.5 to <6.0 mEq/L | 2.9% | sK+ 3.0 - <3.5 mEq/L | 4.0% |  |  |  |
|  |  |  |  |  |  |  | sK+ 6.0 to <6.5 mEq/L | 0.6% | sK+ 3.5 - 4.0 mEq/L | 17.6% | sK+ 4.5 to <5.0 mEq/L | 29.4% |  |
|  |  |  |  |  |  |  | sK+ 6.5 to 8.0 mEq/L | 0.5% | NR | NR |  |  |  |
|  |  |  |  | Subgroup | Patients with stage 5 CKD | 1,488 | sK+ 5.0 to <5.5 mEq/L | 18.0% | sK+ 2.5 - <3. 0 mEq/L | 1.0% | sK+ 4.0 to <4.5 mEq/L | 27.0% |  |
|  |  |  |  |  |  |  | sK+ 5.5 to <6.0 mEq/L | 5.1% | sK+ 3.0 - <3.5 mEq/L | 4.3% |  |  |  |
|  |  |  |  |  |  |  | sK+ 6.0 to <6.5 mEq/L | 0.9% | sK+ 3.5 - 4.0 mEq/L | 14.7% | sK+ 4.5 to <5.0 mEq/L | 27.6% |  |
|  |  |  |  |  |  |  | sK+ 6.5 to 8.0 mEq/L | 1.3% | NR | NR |  |  |  |
| Luo 2016 ([109](#_ENREF_109)) | USA | Electronic health records | 1st Jan 2009-30th Jun 2013 | Overall | Patients with CKD with eGFR <60 mL/min/1.73 m2 | 55,266 | sK+ 5.0 to 5.4 mEq/L | 15.0% | sK+ <3.5 mEq/L | 1.6% | sK+ 3.5 to 3.9 mEq/L | 10.2% |  |
|  |  |  |  |  |  |  | sK+ 5.5 to 5.9 mEq/L | 4.0% |  |  | sK+ 4.0 to 4.4 mEq/L | 33.9% |  |
|  |  |  |  |  |  |  | sK+ ≥6.0 mEq/L | 1.1% |  |  | sK+ 4.5 to 4.9 mEq/L | 34.3% |  |
|  |  |  |  | Subgroup | Patients with CKD with eGFR <60 mL/min/1.73 m2 + DM | 13,946 | sK+ 5.0 to 5.4 mEq/L | 17.3% |  | 1.3% | sK+ 3.5 to 3.9 mEq/L | 9.0% |  |
|  |  |  |  |  |  |  | sK+ 5.5 to 5.9 mEq/L | 4.8% |  |  | sK+ 4.0 to 4.4 mEq/L | 30.2% |  |
|  |  |  |  |  |  |  | sK+ ≥6.0 mEq/L | 1.5% |  |  | sK+ 4.5 to 4.9 mEq/L | 35.9% |  |
| Nakhoul 2015 ([90](#_ENREF_90)) | USA | Electronic health record-based CKD registry | 1st Jan 2005-15th Sept 2009 | Overall | Patients with stage 3-4 CKD | 36,359 | sK+ 5.0 to 5.4 mmol/L | 10.8% | sK+ <3.5 mEq/L | 3.0% | sK+ 3.5 to 3.9 mmol/L | 14.8% |  |
|  |  |  |  |  |  |  | sK+ ≥5.5 mmol/L | 3.3% |  |  | sK+ 4.0 to 4.9 mmol/L | 68.0% |  |
|  |  |  |  | Subgroup | Patients with stage 3-4 CKD + DM | 7,976 | sK+ 5.0 to 5.4 mmol/L | 14.5% |  | 2.3% | sK+ 3.5 to 3.9 mmol/L | 10.7% |  |
|  |  |  |  |  |  |  | sK+ ≥5.5 mmol/L | 5.2% |  |  | sK+ 4.0 to 4.9 mmol/L | 67.3% |  |
|  |  |  |  | Subgroup | Patients with stage 3-4 CKD + HT | 30,659 | sK+ 5.0 to 5.4 mmol/L | 11.1% |  | 2.8% | sK+ 3.5 to 3.9 mmol/L | 14.4% |  |
|  |  |  |  |  |  |  | sK+ ≥5.5 mmol/L | 3.3% |  |  | sK+ 4.0 to 4.9 mmol/L | 68.4% |  |
|  |  |  |  | Subgroup | Patients with stage 3-4 CKD + HF | 2,966 | sK+ 5.0 to 5.4 mmol/L | 12.7% |  | 3.6% | sK+ 3.5 to 3.9 mmol/L | 13.3% |  |
|  |  |  |  |  |  |  | sK+ ≥5.5 mmol/L | 4.6% |  |  | sK+ 4.0 to 4.9 mmol/L | 65.9% |  |
| Integrated CKD care program Kaohsiung for delaying Dialysis Wang 2013 ([41](#_ENREF_41)) | Taiwan | Patients recruited from the nephrology out-patient departments of two hospitals in southern Taiwan | 11th Nov 2002- 31st Jul 2010 | Overall | Patients with stage 1-4 CKD not on RRT | 2,500 | sK+ >5.0 mEq/L | 7.0% | sK+ <3.5 mEq/L | 7.8% | sK+ 3.5 to 4.0 mEq/L | 27.3% |  |
|  |  |  |  |  |  |  |  |  |  |  | sK+ 4.0 to 4.5 mEq/L | 39.0% |  |
|  |  |  |  |  |  |  |  |  |  |  | sK+ 4.5 to 5.0 mEq/L | 19.0% |  |
|  |  |  |  | Subgroup | Patients with stage 1-4 CKD not on RRT + HT | 1,538 |  | 6.9% |  | 8.5% | sK+ 3.5 to 4.0 mEq/L | 28.2% |  |
|  |  |  |  |  |  |  |  |  |  |  | sK+ 4.0 to 4.5 mEq/L | 38.1% |  |
|  |  |  |  |  |  |  |  |  |  |  | sK+ 4.5 to 5.0 mEq/L | 18.3% |  |
|  |  |  |  | Subgroup | Patients with stage 1-4 CKD not on RRT + DM | 1,045 |  | 8.2% |  | 6.7% | sK+ 3.5 to 4.0 mEq/L | 24.9% |  |
|  |  |  |  |  |  |  |  |  |  |  | sK+ 4.0 to 4.5 mEq/L | 38.7% |  |
|  |  |  |  |  |  |  |  |  |  |  | sK+ 4.5 to 5.0 mEq/L | 21.5% |  |
|  |  |  |  | Subgroup | Patients with stage 1 CKD not on RRT | 118 |  | 0.8% |  | 11.9% | sK+ 3.5 to 4.0 mEq/L | 51.7% |  |
|  |  |  |  |  |  |  |  |  |  |  | sK+ 4.0 to 4.5 mEq/L | 32.2% |  |
|  |  |  |  |  |  |  |  |  |  |  | sK+ 4.5 to 5.0 mEq/L | 3.4% |  |
|  |  |  |  | Subgroup | Patients with stage 2 CKD not on RRT | 238 |  | 37.8% |  | 14.7% | sK+ 3.5 to 4.0 mEq/L | 40.3% |  |
|  |  |  |  |  |  |  |  |  |  |  | sK+ 4.0 to 4.5 mEq/L | 37.8% |  |
|  |  |  |  |  |  |  |  |  |  |  | sK+ 4.5 to 5.0 mEq/L | 5.9% |  |
|  |  |  |  | Subgroup | Patients with stage 3 CKD not on RRT | 1,183 |  | 41.9% |  | 7.7% | sK+ 3.5 to 4.0 mEq/L | 27.0% |  |
|  |  |  |  |  |  |  |  |  |  |  | sK+ 4.0 to 4.5 mEq/L | 41.9% |  |
|  |  |  |  |  |  |  |  |  |  |  | sK+ 4.5 to 5.0 mEq/L | 19.8% |  |
|  |  |  |  | Subgroup | Patients with stage 4 CKD not on RRT | 961 |  | 36.4% |  | 5.6% | sK+ 3.5 to 4.0 mEq/L | 21.5% |  |
|  |  |  |  |  |  |  |  |  |  |  | sK+ 4.0 to 4.5 mEq/L | 36.4% |  |
|  |  |  |  |  |  |  |  |  |  |  | sK+ 4.5 to 5.0 mEq/L | 23.1% |  |
| Hayes 2012 ([61](#_ENREF_61)) | USA | Nephrology Department at Salem Veteran Affairs Medical Centre | 1st Jan 1990-30th Jun 2007 | Overall | Patients with moderate and advanced non-dialysis-dependent CKD | 1,227 | sK+ >5.3 mEq/L | 7.7% | sK+ <3.6 mEq/L | 3.2% | sK+ 3.6 to 4.5 mEq/L | 45.4% |  |
|  |  |  |  |  |  |  |  |  |  |  | sK+ 4.51 to 5.3 mEq/L | 43.7% |  |
|  |  |  |  | Subgroup | Patients with moderate and advanced non-dialysis-dependent CKD + DM | 672 | sK+ >5.3 mEq/L | 8.5% |  | 2.5% | sK+ 3.6 to 4.5 mEq/L | 43.6% |  |
|  |  |  |  |  |  |  |  |  |  |  | sK+ 4.51 to 5.3 mEq/L | 45.4% |  |
| Jain 2012 ([42](#_ENREF_42)) | USA | Veterans Affairs North Texas Health Care System EMR | Jan 2007-Jun 2010 | Overall | Patients with advanced (stage 3-5) CKD | 1,384 | sK+ >5.0 mmol/L | 20.1% | NR | NR | sK+ ≤5.0 mEq/L | 5.1% |  |
| Hwang 2011 ([66](#_ENREF_66)) | Taiwan | Data recorded from medical charts | Jan 2004- Feb 2004 | Overall | Patients with ESRD on maintenance haemodialysis | 423 | sK+ >5.5 mEq/L | 12.1% | sK+ <3.5 mEq/L | 10.9% | sK+ 3.5 to 5.5 mEq/L | 77.1% |  |
|  |  |  |  | Subgroup | Patients with ESRD on maintenance haemodialysis + DM | 169 |  | 24.3% |  | 30.8% |  | 22.5% |  |
| RRI-CKD Cohort study  Korgaonkar 2010 ([91](#_ENREF_91)) | USA | Four outpatient nephrology clinics | NR | Overall | Patients with stage 3-5 CKD | 820 | sK+ ≥5.5 mmol/L | 7.9% | sK+ ≤4.0 mmol/L | 14.9% | sK+ >4.0 to <5.5 mmol/L | 77.2% |  |
| Parmar 2024 ([63](#_ENREF_63)) | UK | CKD-HF clinic: Electronic health records | 12th April 2019 and 11th September 2021 | Overall | Patients with CKD + HF | 300 | sK+ >5.4 mmol/L | 11.7% | NR | 88.3% | NR | NR |  |
| Sevamontree 2024 ([107](#_ENREF_107)) | Thailand | Outpatient department of Medicine, Ramathibodi Hospital, Mahidol University, Bangkok | January 1st to December 31st, 2021 | Overall | Patients with stage 3-5 CKD | 852 | sK+ ≥5.8 mmol/L | 13.8% | sK+ <5.8 mmol/L | 86.2% | NR | NR |  |
|  |  |  |  | Subgroup | Patients with stage 3a CKD | 392 |  | 2% |  | 98% | NR | NR |  |
|  |  |  |  | Subgroup | Patients with stage 3b CKD | 249 |  | 8.8% |  | 91.2% | NR | NR |  |
|  |  |  |  | Subgroup | Patients with stage 4 CKD | 88 |  | 30.7% |  | 69.3% | NR | NR |  |
|  |  |  |  | Subgroup | Patients with stage 5 CKD | 123 |  | 49.6% |  | 50.4% | NR | NR |  |
| NEFRONA Valdivielso 2024 ([52](#_ENREF_52)) | Spain | 81 Spanish hospitals and 9 primary care centers | October 2009 and June 2011 | Overall | Patients with stage 3-5 CKD | 2408 | sK+ >5.0 mEq/L | 33.6% | sK+ <3.6 mEq/L | 2.4% | NR | NR |  |
|  |  |  |  |  |  |  | sK+ >5.2 mEq/L | 22.7% |  |  |  |  |  |
|  |  |  |  |  |  |  | sK+ >5.5 mEq/L | 10.8% |  |  |  |  |  |
|  |  |  |  |  |  |  | sK+ >6.0 mEq/L | 3.2% |  |  |  |  |  |
|  |  |  |  | Subgroup | Patients with stage 3 CKD | NR | sK+ >5.0 mEq/L | 22.3% |  | 1.9% | sK+ <3.6-5.0 mEq/L | 75.7% |  |
|  |  |  |  | Subgroup | Patients with stage 4-5 CKD | NR |  | 41.3% |  | 2.1% |  | 56.6% |  |
| Gulcicek 2023 ([82](#_ENREF_82)) | Turkey | Electronic database of the tertiary hospital | 31 Jan 2021 to 31 Jan 2022 | Overall | Patients with stages 1-5 CKD | 471 | sK+ ≥5.0 mmol/L | 29.1% | NR | NR | sK+ < 5.0 mmol/L | 70.9% |  |
|  |  |  |  |  |  |  | sK+ >5.0 to <5.5 mmol/L | 21.7% | NR | NR |  |  |  |
|  |  |  |  |  |  |  | sK+ 5.5 to <6.0 mmol/L | 6.2% | NR | NR |  |  |  |
|  |  |  |  |  |  |  | sK+ ≥6.0 mmol/L | 1.3% | NR | NR |  |  |  |
|  |  |  |  |  | Patients with stages 1 and 5 CKD + DM | 270 | sK+ ≥5.0 mmol/L | 31.5% | NR | NR | sK+ < 5.0 mmol/L | 68.5% |  |
| Zhou 2023a ([53](#_ENREF_53)) | China | China National Heart Failure (CN‐HF) Registry | January 1, 2013 and June 30, 2015 | Overall | Patients with CKD + HF | 766 | sK+ >5.0 mmol/L | 37% | sK+ 0-3.5 mmol/L | 7.9% | sK+ >3.5-5.0 mmol/L | 10.2% |  |
| Qadir 2023 ([54](#_ENREF_54)) | Pakistan | Nephrology Unit, Khyber Teaching Hospital, Peshawar | September 6, 2020, to March 5, 2021 | Overall | Patients with stage 1-4 non-dialysis dependent CKD | 150 | sK+ >5.0 mmol/L | 45.3% | NR | NR | NR | 54.7% |  |
| EQUAL study de Rooij 2023 ([79](#_ENREF_79)) | Multinational | Web-based clinical record | NR | Overall | Patients with stage 4-5 CKD | 1,714 | sK+ >5.0 to ≤5.5 mmol/L | 17.2% | sK+ ≤3.5 mmol/L | 2.6% | sK+ >3.5 to ≤4.0 mmol/L | 13.3% |  |
|  |  |  |  |  |  |  | sK+ >5.5 to ≤6.0 mmol/L | 4.9% |  |  | sK+ >4.0 to ≤4.5 mmol/L | 27.7% |  |
|  |  |  |  |  |  |  | sK+ >6.0 mmol/L | 1.8% |  |  | sK+ >4.5 to ≤5.0 mmol/L | 32.6% |  |
|  |  |  | NR | Subgroup | Patients with stage 4-5 CKD + DM | 708 | sK+ >5.0 to ≤5.5 mmol/L | 19.1% | sK+ ≤3.5 mmol/L | 3% | sK+ >3.5 to ≤4.0 mmol/L | 11.6% |  |
|  |  |  |  |  |  |  | sK+ >5.5 to ≤6.0 mmol/L | 5.9% |  |  | sK+ >4.0 to ≤4.5 mmol/L | 26.4% |  |
|  |  |  |  |  |  |  | sK+ >6.0 mmol/L | 1.7% |  |  | sK+ >4.5 to ≤5.0 mmol/L | 32.3% |  |
| Perez-Navarro 2023 ([55](#_ENREF_55)) | Mexico | Nephrology service of the Hospital General de México | Feb 2019 to August 2022 | Overall | Patients with stage 1-5 CKD | 1,361 | sK+ >5.0 mmol/L | 25.3% | NR | NR | NR | 74.7% |  |
|  |  |  |  | Subgroup | Patients with stage 1 CKD | 117 |  | 3% | NR | NR |  | 97% |  |
|  |  |  |  |  | Patients with stage 2 CKD | 150 |  | 2% | NR | NR |  | 98% |  |
|  |  |  |  |  | Patients with stage 3a CKD | 130 |  | 4% | NR | NR |  | 96% |  |
|  |  |  |  |  | Patients with stage 3b CKD | 242 |  | 14% | NR | NR |  | 86% |  |
|  |  |  |  |  | Patients with stage 4 CKD | 301 |  | 21% | NR | NR |  | 79% |  |
|  |  |  |  |  | Patients with stage 5 CKD | 421 |  | 56% | NR | NR |  | 44% |  |
| Wang 2023 ([89](#_ENREF_89)) | China | Peking University First Hospital | Between 2010 and 2020 | Overall | Patients with stage 1-4 CKD | 527 | Instantaneous hyperkalaemia: sK+ ≥5.0 mmol/L for once or lasting for < 3 months | 16.1% | NR | NR | NR | 62.8% |  |
|  |  |  |  |  |  |  | Persistent hyperkalaemia: repeated occurrence lasting for ≥3 months | 21.1% | NR | NR |  |  |  |

*Prevalence was calculated using patients with hyper-, hypo-, or normokalaemia as the numerator and total study population as the denominator, multiplied by 100 to give a percentage.

**Abbreviations:** CKD: chronic kidney disease; CPRD: Clinical Practice Research Datalink; DARE: Data Analytics Research and Evaluation; DM: diabetes mellitus; DOPPS: Dialysis Outcomes and Practice Patterns Study; eGFR: estimated glomerular filtration rate; EMR: electronic medical record; ESRD: end stage renal disease; EU: European union; FFS: Free-For Service; HF: heart failure; HT: hypertension; ICD-10: International Classification of Diseases, Tenth Revision; JMDV: Japan Medical Data Vision; m2: square metre; mEq/L: milliequivalents per litre; mL/min: millilitres per minute; mmol/L: millimoles per litre; NR: not reported; pK+: plasma potassium; RAASi: renin-angiotensin-aldosterone system inhibitors; RRT: renal replacement therapy; sK+: serum potassium; UEBMI: Urban Employee Basic Medical Insurance; UK: United Kingdom; USA: United States of America.

Table S17. Prevalence of RAASi-associated hyperkalaemia

| Study | Country | Time Period | Population type (overall or subgroup) | Population description | Sample size | Treatment* | Hyperkalaemia definition | Prevalence rate |
| --- | --- | --- | --- | --- | --- | --- | --- | --- |
| Johnson 2023 ([49](#_ENREF_49)) | USA | 2000 and 2019 | Overall | Patients with CKD + Hyperkalaemia | 42,714 | RAASi | sK+ >5.0 mEq/L | 10.4% |
| Santoro 2022 ([98](#_ENREF_98)) | Italy | 2010-2017 | Overall | Patients with stage ≤3 and >3 CKD and unspecified stage + Hyperkalaemia | 4,451 | RAASi drugs taken into consideration:  1. ACEi - plain  2. ACEi - combinations  3. ARBs -plain  4. ARB combinations  5. Other agents acting on renin-angiotensin system | sK+ ≥5.5 mmol/L | 24.0% |
| Adelborg 2019 ([46](#_ENREF_46)) | Denmark | 1st Jan 2000-31st Dec 2012 | Overall | CKD patients with first hyperkalaemia event | NR | RAASi | sK+ >5.0 mmol/L | 48.2% |
|  |  |  |  | CKD patients with repeated hyperkalaemia |  |  | ≥2 sK+ tests >5.0 mmol/L | 53.6% |
|  |  |  |  | CKD patients with first hyperkalaemia event |  | ACEi | sK+ >5.0 mmol/L | 34.3% |
|  |  |  |  | CKD patients with repeated hyperkalaemia |  |  | ≥2 sK+ tests >5.0 mmol/L | 39.1% |
|  |  |  |  | CKD patients with first hyperkalaemia event |  | ARB | sK+ >5.0 mmol/L | 17.2% |
|  |  |  |  | CKD patients with repeated hyperkalaemia |  |  | ≥2 sK+ tests >5.0 mmol/L | 19.0% |
|  |  |  |  | CKD patients with first hyperkalaemia event |  | MRA (spironolactone) | sK+ >5.0 mmol/L | 16.6% |
|  |  |  |  | CKD patients with repeated hyperkalaemia |  |  | ≥2 sK+ tests >5.0 mmol/L | 22.0% |
| Kashihara 2019 ([88](#_ENREF_88)) | Japan | 2016 | Overall | Patients with stage 1-5 CKD | NR | RAASi | sK+ ≥5.1 mEq/L | Rate (95% CI): 279.1 (273.5-284.8) per 1000 population |
|  |  |  | Subgroup | Patients with stage 1 CKD |  |  |  | Rate (95% CI): 85.8 (65.8-109.5) per 1000 population |
|  |  |  |  | Patients with stage 2 CKD |  |  |  | Rate (95% CI): 113.9 (104.6-123.8) per 1000 population |
|  |  |  |  | Patients with stage 3a CKD |  |  |  | Rate (95% CI): 156.2 (147.9-164.7) per 1000 population |
|  |  |  |  | Patients with stage 3b CKD |  |  |  | Rate (95% CI): 288 (276.5-299.7) per 1000 population |
|  |  |  |  | Patients with stage 4 CKD |  |  |  | Rate (95% CI): 510 (492.9-527.1) per 1000 population |
|  |  |  |  | Patients with stage 5 CKD |  |  |  | Rate (95% CI): 627.9 (609.0-646.4) per 1000 population |
| Belmar Vega 2019 ([114](#_ENREF_114)) | Spain | 1971-2017 | Overall | Patients with stage 3-5 CKD | NR | RAASi | Mild sK+ 5.5 to 5.9 mmol/L; moderate sK+ 6.0–6.4 mmol/L; severe sK+ 6.5 to 6.9 mmol/L; very severe sK+ ≥7.0 mmol/L | 12.5% |
|  |  |  |  |  |  | Without RAASi |  | 7.7% |
|  |  |  | Subgroup | Patients with stage 3 CKD |  | RAASi |  | 9.3% |
|  |  |  |  |  |  | Without RAASi |  | 5.2% |
|  |  |  |  | Patients with stage 3a CKD |  | RAASi |  | 7.2% |
|  |  |  |  |  |  | Without RAASi |  | 4.3% |
|  |  |  |  | Patients with stage 3b CKD |  | RAASi |  | 11.5% |
|  |  |  |  |  |  | Without RAASi |  | 6.0% |
|  |  |  |  | Patients with stage 4 CKD |  | RAASi |  | 16.1% |
|  |  |  |  |  |  | Without RAASi |  | 5.8% |
|  |  |  |  | Patients with stage 5 CKD |  | RAASi |  | 23.7% |
|  |  |  |  |  |  | Without RAASi |  | 15.6% |
|  |  |  | Overall | Patients with stage 3-5 CKD + DM |  | RAASi |  | 17.3% |
|  |  |  |  |  |  | Without RAASi |  | 7.6% |
|  |  |  |  | Patients with stage 3-5 CKD + HF |  | RAASi |  | 11.5% |
|  |  |  |  |  |  | Without RAASi |  | 3.4% |
| Sadjadi 2009 ([45](#_ENREF_45)) | USA | Data were reviewed over a 12-month period | Overall | Patients with stage 1-5 CKD | 1,163 | ACEi | sK+ >5.0 mEq/L | 20.4% |
|  |  |  |  |  |  |  | sK+ 5-5.5 mEq/L | 16.6% |
|  |  |  |  |  |  |  | sK+ 5.6-6 mEq/L | 4.9% |
|  |  |  |  |  |  |  | sK+ 6 mEq/L | 0.8% |
|  |  |  |  |  | 1,168 | ARB | sK+ >5.0 mEq/L | 31.0% |
|  |  |  |  |  |  |  | sK+ 5-5.5 mEq/L | 22.3% |
|  |  |  |  |  |  |  | sK+ 5.6-6 mEq/L | 5.8% |
|  |  |  |  |  |  |  | sK+ 6 mEq/L | 2.8% |
|  |  |  | Subgroup |  | 175 | ACEi + ARB | sK+ >5.0 mEq/L | 27.4% |
|  |  |  |  |  |  |  | sK+ >5.5 mEq/L | 8.0% |
|  |  |  |  |  |  |  | sK+ ≥6 mEq/L | 2.2% |
|  |  |  | Subgroup |  | 11 | ACEi (captopril) | sK+ >5.0 mEq/L | 27.2% |
|  |  |  |  |  | 84 | ACEi (fosinopril) |  | 21.7% |
|  |  |  |  |  | 831 | ACEi (lisinopril) |  | 21.5% |
|  |  |  |  |  | 225 | ACEi (benazepril) |  | 16.5% |
|  |  |  | Subgroup |  | 1,126 | ARB (irbesartan) |  | 30.6% |
|  |  |  |  |  | 42 | ARB (losartan) |  |  |
|  |  |  | Subgroup | Patients with stage 1-5 CKD + DM | 599 | ACEi |  | 24.0% |
|  |  |  |  |  | 609 | ARB |  | 37.7% |
| NEFRONA study Valdivielso 2024 ([52](#_ENREF_52)) | Spain | October 2009 and June 2011 | Overall | Patients with CKD | 1669 | RAASi | sK+ >5.0 mEq/L | 34.4% |
|  |  |  |  |  | 738 | No RAASi |  | 31.7% |
|  |  |  |  |  | 1668 | RAASi | sK+ >5.2 mEq/L | 22.9% |
|  |  |  |  |  | 739 | No RAASi |  | 22.2% |
|  |  |  |  |  | 1667 | RAASi | sK+ >5.5 mEq/L | 10.5% |
|  |  |  |  |  | 737 | No RAASi |  | 11.4% |
|  |  |  |  |  | 1655 | RAASi | sK+ >6.0 mEq/L | 2.9% |
|  |  |  |  |  | 744 | No RAASi |  | 3.9% |
|  |  |  | Subgroup | Patients with stage 3-5 CKD | 1371 | RAASi | sK+ >5.0 mEq/L | 33.7% |
|  |  |  |  |  | 358 | No RAASi |  | 21.2% |
|  |  |  |  |  | 1367 | RAASi | sK+ >5.2 mEq/L | 21.8% |
|  |  |  |  |  | 358 | No RAASi |  | 12% |
|  |  |  |  |  | 1374 | RAASi | sK+ >5.5 mEq/L | 9.1% |
|  |  |  |  |  | 359 | No RAASi |  | 3.9% |
|  |  |  |  |  | 1389 | RAASi | sK+ >6.0 mEq/L | 1.8% |
|  |  |  |  |  | 0 | No RAASi |  | 0% |

*To comprehensively understand the specific impact of RAASi treatment on patients with CKD, non-RAASi treatment comparisons were also included in the review.

**Abbreviations:** ACEi: angiotensin converting enzyme inhibitors; ARB: angiotensin receptor blocker; CI: confidence interval; CCB: calcium channel blocker; CKD: chronic kidney disease; DM: diabetes mellitus; eGFR: estimated glomerular filtration rate; ESRD: end stage renal disease; HF: heart failure; mEq/L: milliequivalents per litre; mmol/l: millimoles per litre; MRA: mineralocorticoid receptor antagonist; NR: not reported; pK+: plasma potassium; RAASi: renin-angiotensin-aldosterone system inhibitors; sK+: serum potassium; USA: United States of America.

.

Table S18. Incidence of hyper-, hypo- and normokalaemia in CKD patients

| Study | Country | Study setting/data source | Time Period | Population type | Population description | Sample size | Hyperkalaemia | | Hypokalaemia | | Normokalaemia | |
| --- | --- | --- | --- | --- | --- | --- | --- | --- | --- | --- | --- | --- |
|  |  |  |  |  |  |  | **Definition** | **Incidence rate*** | **Definition** | **Incidence rate*** | **Definition** | **Incidence rate*** |
| Hayes 2012 ([61](#_ENREF_61)) | USA | Nephrology Department at Salem Veteran Affairs Medical Centre | 30th Jun 2007 to 1Apr 2009 | Overall | Patients with moderate and advanced non-dialysis-dependent CKD | 1,227 | sK+ >5.3 mEq/L | 21.7 cases per 1000 person-years | sK+ <3.6 mEq/L | 9.7 cases per 1000 person-years | NR | NR |
| Jime´nez-Marrero 2021 ([44](#_ENREF_44)) | Spain | Administrative, hospital and primary care databases | 2nd Jan 2016 to 30th Sept 2017 | Overall | Patients with CKD | 1,129 | sK+ >5.0 mEq/L | 41.9 cases per 1000 person-years | sK+ <3.5 mEq/L | 18.9 cases per 1000 person-years | sK+ ≥3.5 to ≤5.0 mEq/L | 515.6 cases per 1000 person-years |
|  |  |  |  | Subgroup | Patients with CKD who had a first recording of RAASI medication use during January 2nd, 2016, and September 30th, 2017 | 687 |  | 46.1 cases per 1000 person-years |  | 12.6 cases per 1000 person-years |  | 517.6cases per 1000 person-years |

*Incidence rate was calculated (if not already reported) by using population number, number of cases of hyper-, hypo-, or normokalaemia and total patient years included in the study and was expressed as a rate per 1000 person years.

**Abbreviations:** CKD: chronic kidney disease; mEq/L: milliequivalents per litre; NR: not reported; RAASi: renin-angiotensin-aldosterone system inhibitors; sK+: serum potassium; USA: United States of America.

Table S19. Incidence of RAASi-associated hyperkalaemia

| Study | Time Period | Population type | Population description | Sample size | Treatment* | Hyperkalaemia definition | Incidence | |
| --- | --- | --- | --- | --- | --- | --- | --- | --- |
|  |  |  |  |  |  |  | Rate | p-value |
| Li 2023b ([67](#_ENREF_67)) | Within 36 months of follow-up | Overall | Patients with stage 3-5 CKD + HFpEF | 154 | MRA (spironolactone) | sK+ >5.5 mmol/L | 8.4% | p=0.21 |
|  |  |  |  | 233 | No spironolactone |  | 6.4% |  |
|  |  | Subgroup | Patients with stage 3-5 CKD + HFpEF | 22 | MRA (spironolactone - >40 mg) |  | 5.1 per 100 patient-years | p=0.11 |
|  |  |  |  | 41 | MRA (spironolactone - ≤40 mg) |  | 2.7 per 100 patient-years |  |
|  |  | Subgroup | Patients with stage 3a CKD + HFpEF | 47 | MRA (spironolactone) |  | 6.4% | p=0.934 |
|  |  |  | Patients with stage 3b CKD + HFpEF | 59 | MRA (spironolactone) |  | 10.2% |  |
|  |  |  | Patients with stage 4/5 CKD + HFpEF | 48 | MRA (spironolactone) |  | 8.3% |  |
|  |  |  |  | 118 | No spironolactone |  | 11.9% | p=0.002 |
| Lin 2023 ([74](#_ENREF_74)) | Within 2.5 years of mean follow-up | Overall | Patients with ESRD on maintenance dialysis + HF | 2,176 | MRA | ICD-9 diagnostic code of 276.7 | 13.3% (event rate [95% CI]: 5.8 [5.1-6.5] per 100-patient years) | p<0.001 |
|  | Within 2.1 years of mean follow-up |  |  | 6,528 | Non-MRA |  | 9.8% (event rate [95% CI]: 4.9 [4.6-5.3] events per 100-patient years) |  |
|  | NR | Subgroup | Patients with ESRD on maintenance dialysis + HF (without excluding patients with prior exposure to MRA before the initiation of chronic dialysis) | 3,114 | MRA |  | 13.4% (event rate [95% CI]: 6.0 [5.4-6.6] events per 100-patient years) | p<0.001 |
|  |  |  |  | 9,342 | Non-MRA |  | 10.1% (event rate [95% CI]: 5.4 [5.1-5.7] events per 100-patient years) |  |
|  |  |  | Patients with ESRD + HF (Single-centre hospital cohort) | 163 | MRA | sK+ >6.0 mEq/L | 30.7% | p=0.0001 |
|  |  |  |  | 2,421 | Non-MRA |  | 18.4% |  |
| Yang 2023 ([94](#_ENREF_94)) | Within median follow-up of 5.0 years to death | Overall | Patients with advanced CKD + DM | 3,817 | Continued RAASi users | pK+ ≥5.5 mmol/L | 59.4% (crude-incidence of first hyperkalaemia event [95% CI]: 99.6 [95.6-103.8] per 1000 person-years) | NR |
|  |  |  |  | 583 | Discontinued RAASi users |  | 51.1% (crude-incidence of first hyperkalaemia event [95% CI]: 101.2 [90.1-113.4] per 1000 person-years) |  |
| MRA-ACE  Tumlin 2022 ([21](#_ENREF_21)) | NR | Overall | Patients with diabetic kidney disease | 18 | Maximum tolerated ACE/ARB | sK+ >5.5 mmol/L | 22.2% | p<0.027 |
|  |  |  |  | 14 | Maximum tolerated ACE/ARB + MRA (spironolactone - 25 mg) |  | 57.1% |  |
| Riccio 2022 ([70](#_ENREF_70)) | Within follow-up (time of enrolment, until the earliest transfer out of the practice, loss to follow-up, dialysis initiation, death, or end of the study period (December 2019) | Overall | Patients with stage 1 to 5 CKD | 556 | ACEi/ ARB/ ACEi and ARB combination | sK+ >5.1 mmol/L | 9.2% | p<0.0001 |
|  |  | Subgroup | Patients with stage 1 CKD | 57 |  |  | 0% | NR |
|  |  |  | Patients with stage 2 CKD | 106 |  |  | 2.8% | NR |
|  |  |  | Patients with stage 3 CKD | 194 |  |  | 5.1% | NR |
|  |  |  | Patients with stage 4 CKD | 114 |  |  | 18.4% | p<0.05 compared to stage 1, 2 and 3 CKD |
|  |  |  | Patients with stage 5 CKD | 85 |  |  | 20.0% | p<0.05 compared to stage 1, 2 and 3 CKD |
| EX-DKD  Uchida 2022 ([97](#_ENREF_97)) | Within 12 weeks of treatment | Overall | Patients with diabetic kidney disease + HT | 112 | MRA (esaxerenone) | sK+ ≥5.5 mEq/L | 2.7% | NR |
|  |  |  |  |  |  | sK+ ≥6.0 mEq/L | 0.0% | NR |
|  |  | Subgroup | Patients with diabetic kidney disease + HT (UACR <30 mg/gCr) | 46 |  | sK+ ≥5.5 mEq/L | 4.3% | NR |
|  |  |  |  |  |  | sK+ ≥6.0 mEq/L | 0.0% | NR |
|  |  |  | Patients with diabetic kidney disease + DM (UACR 30 to <300 mg/gCr) | 42 |  | sK+ ≥5.5 mEq/L | 2.4% | NR |
|  |  |  |  |  |  | sK+ ≥6.0 mEq/L | 0.0% | NR |
|  |  |  | Patients with diabetic kidney disease + DM (UACR 300 to <1000 mg/gCr) | 24 |  | sK+ ≥5.5 mEq/L | 0.0% | NR |
|  |  |  |  |  |  | sK+ ≥6.0 mEq/L | 0.0% | NR |
| BLOCK-CKD  Bakris 2021 ([12](#_ENREF_12)) | Within 12 weeks | Overall | Patients with stage 3b or 4 CKD + Uncontrolled grade 1 and 2 systolic HT | 51 | MRA (KBP-5074 - 0.25 mg) | ≥5.6 mmol/L (Central laboratory assessment) | 9.8% | NR |
|  |  |  |  |  |  | 5.6-5.9 mmol/L (Investigator-reported hyperkalaemia) | 7.8% |  |
|  |  |  |  |  |  | ≥6.0 mmol/L (Investigator-reported hyperkalaemia) | 0.0% |  |
|  |  |  |  | 54 | MRA (KBP-5074 - 0.5 mg) | ≥5.6 mmol/L (Central laboratory assessment) | 13.0% |  |
|  |  |  |  |  |  | 5.6 to 5.9 mmol/L (Investigator-reported hyperkalaemia) | 12.9% |  |
|  |  |  |  |  |  | ≥6.0 mmol/L (Investigator-reported hyperkalaemia) | 0.0% |  |
|  |  |  |  | 57 | Placebo | ≥5.6 mmol/L (Central laboratory assessment) | 8.8% |  |
|  |  |  |  |  |  | 5.6 to 5.9 mmol/L (Investigator-reported hyperkalaemia) | 3.5% |  |
|  |  |  |  |  |  | ≥6.0 mmol/L (Investigator-reported hyperkalaemia) | 0.0% |  |
| ROTATE-3  Provenzano 2022 ([8](#_ENREF_8)) | Within 4 weeks of treatment | Overall | Patients with stage 2-4 CKD | 46 | MRA (eplerenone) | sK+ >5 mmol/L | 17.4% | p<0.003 |
|  |  |  |  |  | SGLT2i (dapagliflozin) + MRA (eplerenone) |  | 4.3% |  |
|  |  |  |  |  | SGLT2i (dapagliflozin) |  | 0.0% |  |
|  | After 4 weeks (post hoc exploratory end point) |  |  | 46 | MRA (eplerenone) |  | 26.1% | p<0.002 |
|  |  |  |  |  | SGLT2i (dapagliflozin) + MRA (eplerenone) |  | 10.9% |  |
|  |  |  |  |  | SGLT2i (dapagliflozin) |  | 2.2% |  |
| Salik 2022 ([51](#_ENREF_51)) | Within 28 days of treatment | Overall | Patients with CKD | 186 | ARB | sK+ >5.0 mmol/L | 9.7% | NR |
|  |  |  |  | 166 | ACEi |  | 24.7% |  |
| Wetmore 2021 ([75](#_ENREF_75)) | Follow-up until earliest of death, transfer out, or 31st Dec 2015 | Subgroup | Patients with stage 1 CKD with previous history hyperkalaemia | 1,117 | RAASi | Moderate hyperkalaemia: sK+ >5.5 to ≤6.0 mmol/L; Severe hyperkalaemia: sK+ >6.0 mmol/L | 6.1 per 100 patient-years | NR |
|  |  |  | Patients with stage 1 CKD without previous history hyperkalaemia | 1,186 |  |  | 1.0 per 100 patient-years |  |
|  |  |  | Patients with stage 2 CKD with previous history hyperkalaemia | 8,173 |  |  | Rate (95% CI): 3.0 (2.7-3.2) per 100 patient-years |  |
|  |  |  | Patients with stage 2 CKD without previous history hyperkalaemia | 9,812 |  |  | Rate (95% CI): 0.6 (0.6-0.7) per 100 patient-years |  |
|  |  |  | Patients with stage 3 CKD with previous history hyperkalaemia | 33,907 |  |  | Rate (95% CI): 5.8 (5.6-6.0) per 100 patient-years |  |
|  |  |  | Patients with stage 3 CKD without previous history hyperkalaemia | 27,742 |  |  | Rate (95% CI): 1.1 (1.0-1.2) per 100 patient-years |  |
|  |  |  | Patients with stage 4 CKD with previous history hyperkalaemia | 2,977 |  |  | Rate (95% CI): 16.1 (14.7-17.4) per 100 patient-years |  |
|  |  |  | Patients with stage 4 CKD without previous history hyperkalaemia | 867 |  |  | Rate (95% CI): 3.5 (2.6-4.3) per 100 patient-years |  |
|  |  |  | Patients with stage 5 CKD with previous history hyperkalaemia | 535 |  |  | Rate (95% CI): 14.2 (11.7-16.7) per 100 patient-years |  |
|  |  |  | Patients with stage 5 CKD without previous history hyperkalaemia | 158 |  |  | Rate (95% CI): 8.0 (5.2-10.9) per 100 patient-years |  |
|  |  |  | Patients unknown stage CKD with previous history hyperkalaemia | 24 |  |  | 8.4 per 100 patient-years |  |
|  |  |  | Patients unknown stage CKD without previous history hyperkalaemia | 49 |  |  | 2.9 per 100 patient-years |  |
| Buckallew 2021 ([95](#_ENREF_95)) | Feb 2018-Aug 2019 | Overall | Patients with stage 3-5 CKD + HF | 121 | MRA (spironolactone) | sK+ ≥5.5 mEq/L | 6.6% | NR |
|  |  | Subgroup | Patients with stage 3 CKD + HF | 84 |  |  | 4.8% | p<0.05 |
|  |  |  | Patients with stage 4 CKD + HF | 30 |  |  | 3.3% |  |
|  |  |  | Patients with stage 5 CKD + HF | 7 |  |  | 42.9% |  |
| DRINK  Tang 2021 ([11](#_ENREF_11)) | Within 3 years of treatment | Overall | Patients with stage 3 or 4 non-diabetic CKD | 37 | ARB (Losartan) + DRI (aliskiren) | pK+ ≥5.5 mmol/L | 18.9% | NR |
|  |  |  |  | 39 | ARB (Losartan) |  | 5.1% |  |
| Obertynska 2021 ([112](#_ENREF_112)) | At week 12 | Overall | Patients with stage 3 CKD + HFrEF <40% | 101 | MRA (spironolactone) | sK+ 5.5 to 5.9 mmol/L | 13.0% | NR |
|  |  |  |  |  |  | sK+ ≥6.0 mmol/L (Severe Hyperkalaemia) | <5% |  |
| An 2021 ([102](#_ENREF_102)) | 2008-2018 | Overall | Patients with stage 2-4 diabetic kidney disease + HT (After matching) | 1,282 | Combination therapy (MRA + ACEI/ARB) | ICD-9 diagnostic code of 276.7 or ICD-10 diagnostic code of E87.5, or pK+/sK+ ≥5.5 mEq/L | 22.3 per 100 person-years | NR |
|  |  |  |  | 1,282 | Monotherapy (ACEI/ARB only) |  | 10.9 per 100 person-years |  |
| Qu 2021 ([71](#_ENREF_71)) | Within 30 months of follow-up | Overall | Patients with stage 3-5 CKD | 200 | MRA (spironolactone) | sK+ >5.5 mmol/L | 9.0% | p=0.68 |
|  |  |  |  | 360 | Standard treatment |  | 5.0% |  |
|  |  |  |  | 44 | MRA (spironolactone - >40 mg per day) |  | 20.5% | p=0.719 |
|  |  |  |  | 156 | MRA (spironolactone - ≤40 mg per day) |  | 5.8% |  |
|  |  |  |  | 44 | MRA (spironolactone - >40 mg per day) | Life-threatening sK+ | 6.8% | NR |
|  |  |  |  | 44 | MRA (spironolactone - ≤40 mg per day) | Severe sK+ | 22.2% | p=0.527 |
|  |  |  |  | 156 | MRA (spironolactone - ≤40 mg per day) |  | 11.1% |  |
| Edwards 2021 ([1](#_ENREF_1)) | Within 40 weeks of treatment | Overall | Patients with stage 2 or 3 non-diabetic CKD | 77 | MRA (spironolactone) | sK+ >5.0 mEq/L | 15.6% | p=0.02 |
|  |  |  |  | 77 | Diuretic (chlorthalidone) |  | 2.6% |  |
| TRANSITION  Straburzynska-Migaj 2021 ([2](#_ENREF_2)) | At week 10 of treatment | Overall | Patients with stage 3 CKD + HFpEF | 476 | ARNi (sacubitril/valsartan) | NR | 16.3% | NR |
| FIGARO-DKD  Pitt 2021 ([9](#_ENREF_9)) | NR | Overall | Patients with stage 2-4 CKD with moderately elevated albuminuria or stage 1 or 2 CKD with severely elevated albuminuria + DM | 3,683 | MRA (finerenone) | sK+ >5.5 mmol/L or sK+ >6.0 mmol/L (Investigator-reported) | Treatment emergent: 10.8%  Drug-related: 6.6%  Serious treatment emergent: 0.7% | NR |
|  |  |  |  | 3,658 | Placebo |  | Treatment emergent: 5.3 %  Drug-related: 3.1%  Serious treatment emergent: 0.1% |  |
|  |  |  |  | 3,677 | MRA (finerenone) | sK+ >5.5 mmol/L (Central laboratory assessment) | Treatment emergent: 13.5% | NR |
|  |  |  |  | 3,655 | Placebo |  | Treatment emergent: 6.4% |  |
|  |  |  |  | 3,677 | MRA (finerenone) | sK+ >6.0 mmol/L (Central laboratory assessment) | Treatment emergent: 2.3% | NR |
|  |  |  |  | 3,655 | Placebo |  | Treatment emergent: 1.2% |  |
| FIDELIO-DKD  Bakris 2020 ([10](#_ENREF_10)) | NR | Overall | Patients with advanced CKD + DM | 2,827 | MRA (finerenone) | >5.5 mmol/L | 11.8% | NR |
|  |  |  |  | 2,831 | Placebo |  | 4.8% |  |
| FIDELIO-DKD  Agarwal 2022 ([128](#_ENREF_128)) | Within 2.6 years of median follow-up | Overall | Patients with advanced CKD + DM | 2,802 | MRA (finerenone) | Moderate hyperkalaemia: sK+ >6.0 mmol/L | 4.5% | NR |
|  |  |  |  | 2,785 |  | Mild hyperkalaemia: sK+ >5.5 mmol/L | 21.4% |  |
|  |  |  |  | 2,796 | Placebo | Moderate hyperkalaemia: sK+ >6.0 mmol/L | 1.4% |  |
|  |  |  |  | 2,775 |  | Mild hyperkalaemia: sK+ >5.5 mmol/L | 9.2% |  |
| FIDELIO-DKD  Filippatos 2021 ([130](#_ENREF_130)) | NR | Subgroup | Patients with advanced CKD and DM with history of CVD at baseline | 1,301 | MRA (finerenone) | NR | Treatment-emergent:18.3%  Treatment emergent (in ≥5% in patients): 15.7%  Any: 18.3%  Drug-related: 11.8%  Hyperkalaemia SAE: 1.6% | NR |
|  |  |  |  | 1,299 | Placebo |  | Treatment-emergent: 8.5%  Treatment emergent  (in ≥5% in patients): 7.4%  Any: 8.5%  Drug-related: 4.2%  Hyperkalaemia SAE: 0.5% |  |
|  |  |  | Patients with advanced CKD and DM without history of CVD at baseline | 1,526 | MRA (finerenone) | NR | Treatment-emergent: 18.2%  Treatment emergent  (in ≥5% in patients): 15.9%  Any: 18.2%  Drug-related: 11.8%  Hyperkalaemia SAE: 1.5% | NR |
|  |  |  |  | 1,532 | Placebo |  | Treatment-emergent: 9.5%  Treatment emergent (in ≥5% in patients): 8.2%  Any: 9.5%  Drug-related: 5.3%  Hyperkalaemia SAE: 0.4% |  |
| FIDELIO-DKD  Rossing 2022a ([131](#_ENREF_131)) | NR | Subgroup | Patients with advanced CKD and DM with concomitant SGLT2i therapy at baseline | 124 | MRA (finerenone) | sK+ >6.0 mmol/L (central laboratory assessments) | Treatment-emergent: 0% | NR |
|  |  |  |  |  |  | sK+ >5.5 mmol/L (central laboratory assessments) | Treatment-emergent: 6.5% |  |
|  |  |  |  | 135 | Placebo | sK+ >6.0 mmol/L (central laboratory assessments) | Treatment-emergent: 0% |  |
|  |  |  |  |  |  | sK+ >5.5 mmol/L (central laboratory assessments) | Treatment-emergent: 3% |  |
|  |  |  | Patients with advanced CKD and DM without concomitant SGLT2i therapy at baseline | 2,703 | MRA (finerenone) | sK+ >6.0 mmol/L (central laboratory assessments) | Treatment-emergent: 4.7% | NR |
|  |  |  |  |  |  | sK+ >5.5 mmol/L (central laboratory assessments) | Treatment-emergent: 21.8% |  |
|  |  |  |  | 2,696 | Placebo | sK+ >6.0 mmol/L (central laboratory assessments) | Treatment-emergent: 1.4% |  |
|  |  |  |  |  |  | sK+ >5.5 mmol/L (central laboratory assessments) | Treatment-emergent: 9.3% |  |
| FIDELIO-DKD  Rossing 2022b ([132](#_ENREF_132)) | NR | Overall | Patients with advanced CKD and DM with baseline HbA1C <7.5% | 1,382 | MRA (finerenone) | sK+ >6.0 mmol/L (central laboratory assessment) | 4.4% | NR |
|  |  |  |  |  |  | sK+ >5.5 mmol/L (central laboratory assessment) | 21.3% |  |
|  |  |  |  | 1,407 | Placebo | sK+ >6.0 mmol/L (central laboratory assessment) | 1.3% |  |
|  |  |  |  |  |  | sK+ >5.5 mmol/L (central laboratory assessment) | 8.6% |  |
|  |  |  | Patients with advanced CKD and DM with baseline HbA1C >7.5% | 1,439 | MRA (finerenone) | sK+ >6.0 mmol/L (central laboratory assessment) | 4.6% | NR |
|  |  |  |  |  |  | sK+ >5.5 mmol/L (central laboratory assessment) | 21.4% |  |
|  |  |  |  | 1,421 | Placebo | sK+ >6.0 mmol/L (central laboratory assessment) | 1.4% |  |
|  |  |  |  |  |  | sK+ >5.5 mmol/L (central laboratory assessment) | 9.9% |  |
|  |  |  | Patients with advanced CKD and DM without insulin use at baseline | 989 | MRA (finerenone) | sK+ >6.0 mmol/L (central laboratory assessment) | 4.6% | NR |
|  |  |  |  |  |  | sK+ >5.5 mmol/L (central laboratory assessment) | 21.5% |  |
|  |  |  |  | 1,041 | Placebo | sK+ >6.0 mmol/L (central laboratory assessment) | 1.2% |  |
|  |  |  |  |  |  | sK+ >5.5 mmol/L (central laboratory assessment) | 8.2% |  |
|  |  |  | Patients with advanced CKD and DM with insulin use at baseline | 1,838 | MRA (finerenone) | sK+ >6.0 mmol/L (central laboratory assessment) | 4.5% | NR |
|  |  |  |  |  |  | sK+ >5.5 mmol/L (central laboratory assessment) | 21.8% |  |
|  |  |  |  | 1,790 | Placebo | sK+ >6.0 mmol/L (central laboratory assessment) | 1.5% |  |
|  |  |  |  |  |  | sK+ >5.5 mmol/L (central laboratory assessment) | 10.7% |  |
| FIDELIO-DKD (Chinese subgroup) Zhang 2023 ([133](#_ENREF_133)) | Median follow-up of 30 months | Subgroup | Patients with CKD + DM | 188 | ARB (finerenone) | sK+ >5.5 mmol/L | 20.7% | NR |
|  |  |  |  |  |  | sK+ >6.0 mmol/L | 3.7% | NR |
|  |  |  |  | 884 | Placebo | sK+ >5.5 mmol/L | 10.5% | NR |
|  |  |  |  |  |  | sK+ >6.0 mmol/L | 2.2% | NR |
| Kashihara 2019 ([88](#_ENREF_88)) | Within follow-up (from the index date until the date of emigration from the database, date of death, or end of the study period | Overall | Patients with stage 1-5 CKD | NR | RAASi (ACEi/ARB/MRA) | sK+ ≥5.1 mEq/L | Rate (95% CI): 19.1 (18.9-19.4) per 100 patient-year-at-risk | NR |
|  |  | Subgroup | Patients with stage 1 CKD |  |  |  | Rate (95% CI): 5.9 (5.3-6.5) per 100 patient-year-at-risk | NR |
|  |  |  | Patients with stage 2 CKD |  |  |  | Rate (95% CI): 8.7 (8.4-9.1) per 100 patient-year-at-risk | NR |
|  |  |  | Patients with stage 3a CKD |  |  |  | Rate (95% CI): 10.9 (10.6-11.2) per 100 patient-year-at-risk | NR |
|  |  |  | Patients with stage 3b CKD |  |  |  | Rate (95% CI): 23.6 (23.0-24.3) per 100 patient-year-at-risk | NR |
|  |  |  | Patients with stage 4 CKD |  |  |  | Rate (95% CI): 57.6 (56.0-58.3) per 100 patient-year-at-risk | NR |
|  |  |  | Patients with stage 5 CKD |  |  |  | Rate (95% CI): 85.5 (83.1-88.1) per 100 patient-year-at-risk | NR |
|  | Over 3 years |  | Patients with stage 1-5 CKD | NR |  | sK+ ≥5.1 mEq/L | Cumulative incidence of first hyperkalaemia episode (95% CI): 40.1% (39.6-40.5) | NR |
|  |  |  |  |  |  | sK+ ≥5.5 mEq/L | Cumulative incidence of first hyperkalaemia episode (95% CI): 29.7% (29.3-30.2) | NR |
|  |  |  |  |  |  | sK+ ≥6.0 mEq/L | Cumulative incidence of first hyperkalaemia episode (95% CI): 14.9% (14.6-15.2) | NR |
|  | Observation year 1 |  |  |  |  | sK+ ≥5.1 mEq/L | Cumulative incidence of first hyperkalaemia episode: 29.7% | NR |
|  |  |  |  |  |  | sK+ ≥5.5 mEq/L | Cumulative incidence of first hyperkalaemia episode: 18.9% | NR |
|  |  |  |  |  |  | sK+ ≥6.0 mEq/L | Cumulative incidence of first hyperkalaemia episode: 8.4% | NR |
|  | Observation year 2 |  |  |  |  | sK+ ≥5.1 mEq/L | Cumulative incidence of first hyperkalaemia episode: 36.0% | NR |
|  |  |  |  |  |  | sK+ ≥5.5 mEq/L | Cumulative incidence of first hyperkalaemia episode: 25.0% | NR |
|  |  |  |  |  |  | sK+ ≥6.0 mEq/L | Cumulative incidence of first hyperkalaemia episode: 12.1% | NR |
|  | Observation year 3 |  |  |  |  | sK+ ≥5.1 mEq/L | Cumulative incidence of first hyperkalaemia episode: 40.2% | NR |
|  |  |  |  |  |  | sK+ ≥5.5 mEq/L | Cumulative incidence of first hyperkalaemia episode: 29.9% | NR |
|  |  |  |  |  |  | sK+ ≥6.0 mEq/L | Cumulative incidence of first hyperkalaemia episode: 14.9% | NR |
|  | Observation year 4 |  |  |  |  | sK+ ≥5.1 mEq/L | Cumulative incidence of first hyperkalaemia episode: 43.4% | NR |
|  |  |  |  |  |  | sK+ ≥5.5 mEq/L | Cumulative incidence of first hyperkalaemia episode: 33.5% | NR |
|  |  |  |  |  |  | sK+ ≥6.0 mEq/L | Cumulative incidence of first hyperkalaemia episode: 17.5% | NR |
|  | Observation year 5 |  |  |  |  | sK+ ≥5.1 mEq/L | Cumulative incidence of first hyperkalaemia episode: 45.9% | NR |
|  |  |  |  |  |  | sK+ ≥5.5 mEq/L | Cumulative incidence of first hyperkalaemia episode: 36.7% | NR |
|  |  |  |  |  |  | sK+ ≥6.0 mEq/L | Cumulative incidence of first hyperkalaemia episode: 19.7% | NR |
|  | Observation year 6 |  |  |  |  | sK+ ≥5.1 mEq/L | Cumulative incidence of first hyperkalaemia episode: 47.9% | NR |
|  |  |  |  |  |  | sK+ ≥5.5 mEq/L | Cumulative incidence of first hyperkalaemia episode: 39.3% | NR |
|  |  |  |  |  |  | sK+ ≥6.0 mEq/L | Cumulative incidence of first hyperkalaemia episode: 21.8% | NR |
| Silvarino 2019 ([106](#_ENREF_106)) | 1st Sept 2004- 31st Aug 2016 | Overall | Patients with stage 1-5 non-dialysis dependent CKD | 221 | ACEi | sK+ ≥5.6 mEq/L | 12.7% | p=0.17 |
|  |  |  |  | 196 | ARB |  | 7.1% |  |
|  |  |  |  | 703 | No ACEI/ARB |  | 10.9% |  |
|  |  | Subgroup | Patients with stage 1-2 non-dialysis dependent CKD | 63 | ACEi |  | 6.3% | p=0.9 |
|  |  |  |  | 39 | ARB |  | 5.1% |  |
|  |  |  |  | 65 | No ACEI/ARB |  | 4.6% |  |
|  |  |  | Patients with stage 3-5 non-dialysis dependent CKD | 158 | ACEi |  | 15.2% | p=0.11 |
|  |  |  |  | 157 | ARB |  | 7.6% |  |
|  |  |  |  | 638 | No ACEI/ARB |  | 11.6% |  |
| Jun 2019 ([78](#_ENREF_78)) | After follow-up 3.9 years | Overall | Patients with stage 3-5 CKD | 20,184 | RAASi | >6.0 mmol/L or coded or free-text recorded diagnosis of hyperkalaemia | Rate (95% CI): 3.1 (2.9-3.2) per 100 person-years | NR |
|  |  | Subgroup | Patients with stage 3a CKD | 9,444 | RAASi |  | 1. Unadjusted rate (95% CI): 2.0 (1.9-2.2) per 100 person-years 2. Adjusted rate (95% CI): 1.8 (1.6-2.0) per 100 person-years | p<0.001 (for adjusted rate) |
|  |  |  | Patients with stage 3b CKD | 7,739 |  |  | 1. Unadjusted rate (95% CI): 3.3 (3.0-3.5) per 100 person-years 2. Adjusted rate (95% CI): 3.0 (2.7-3.3) per 100 person-years |  |
|  |  |  | Patients with stage 4 CKD | 2,632 |  |  | 1. Unadjusted rate (95% CI): 6.2 (5.7-6.8) per 100 person-years 2. Adjusted rate (95% CI): 5.5 (4.9-6.2) per 100 person-years |  |
|  |  |  | Patients with stage 5 CKD | 369 |  |  | 1. Unadjusted rate (95% CI): 10.4 (8.4-12.7) per 100 person-years 2. Adjusted rate (95% CI): 9.3 (7.3-11.9) per 100 person-years |  |
| TOPCAT  Beldhuis 2019 ([7](#_ENREF_7)) | 4-year follow up | Overall | Patients with stage 1 and 2 CKD + HFpEF | 413 | MRA (spironolactone) | sK+ >5.5 mmol/L | Rate (95% CI): 6.8 (5.4-8.6) per 100 patient-years | p<0.001 |
|  |  |  |  | 410 | Placebo |  | Rate (95% CI): 1.4 (0.9-2.3) per 100 patient-years |  |
|  |  |  | Patients with stage 3a CKD + HFpEF | 264 | MRA (spironolactone) |  | Rate (95% CI): 11.9 (9.5-14.9) per 100 patient-years | p<0.001 |
|  |  |  |  | 269 | Placebo |  | Rate (95% CI): 3.6 (2.5-5.2) per 100 patient-years |  |
|  |  |  | Patients with stage 3b CKD + HFpEF | 209 | MRA (spironolactone) |  | Rate (95% CI): 14.7 (11.5-18.8) per 100 patient-years | p<0.001 |
|  |  |  |  | 202 | Placebo |  | Rate (95% CI): 5.3 (3.7-7.8) per 100 patient-years |  |
| MiREnDa  Hammer 2019 ([4](#_ENREF_4)) | Week 40 | Overall | Patients with ESRD on haemodialysis | 50 | MRA (spironolactone) | Moderate sK+ 6.0 to 6.5 mmol/L | 66.0% (155 events) | p=0.602 (events: p=0.034) |
|  |  |  |  | 47 | Placebo |  | 61.7% (80 events) |  |
|  |  |  |  | 50 | MRA (spironolactone) | Severe sK+ ≥6.5 mmol/L | 24.0% (14 events) | p=0.72 (events: p=0.225) |
|  |  |  |  | 47 | Placebo |  |  |  |
|  |  |  |  |  |  |  | 21.3% (24 events) |  |
| Spin-D  Charyatan 2019 ([5](#_ENREF_5)) | 36 weeks of treatment | Overall | Patients with ESRD on haemodialysis | 27 | MRA (spironolactone - 12.5 mg per day) | >6.5 mEq/L | 14.8% (event rate: 0.3 per patient-year) | Event rate: p=0.9 |
|  |  |  |  | 26 | MRA (spironolactone - 25 mg per day) |  | 15.4% (event rate: 0.2 per patient-year) |  |
|  |  |  |  | 25 | MRA (spironolactone - 50 mg per day) |  | 32.0% (event rate: 0.9 per patient-year) |  |
|  |  |  |  | 51 | Placebo |  | 17.6.0% (event rate: 0.5 per patient-year) |  |
| UK HARP-III  Haynes 2018 ([6](#_ENREF_6)) | Within 12 months of treatment | Overall | Patients with stage A1-A3 CKD with eGFR 20 to 60 mL/min/1.73 m2 | 207 | ARNi (sacubitril/valsartan) | sK+ ≥5.5 mmol/L | 32.0 | p=0.01 |
|  |  |  |  |  |  | sK+ ≥5.5 to <6.0 mmol/L | 21.0% |  |
|  |  |  |  |  |  | sK+ ≥6.0 to <6.5 mmol/L | 10.0% |  |
|  |  |  |  |  |  | sK+ ≥6.5 mmol/L | 1.0% |  |
|  |  |  |  | 207 | ARB (irbesartan) | sK+ ≥5.5 mmol/L | 24.0% |  |
|  |  |  |  |  |  | sK+ ≥5.5 to <6.0 mmol/L | 18.0% |  |
|  |  |  |  |  |  | sK+ ≥6.0 to <6.5 mmol/L | 3.0% |  |
|  |  |  |  |  |  | sK+ ≥6.5 mmol/L | 2.0% |  |
| Hirai 2018 ([72](#_ENREF_72)) | 2015-2017 | Overall | Patients with stage 3-5 CKD and unknown stage CKD | 2,987 | ACEi/ARB | sK+ >5.5 mEq/L | 7.8% | NR |
| Fang 2018 ([119](#_ENREF_119)) | Within 6 months after hospital discharge | Overall | Patients with CKD hospitalised for MI | 20,620 | ACEi/ARB | Primary or secondary diagnosis code of 276.7 in inpatient claims | Cumulative incidence: 0.6 per 100 patients at risk | NR |
|  | After 12 months of hospital discharge |  |  |  |  | Primary or secondary diagnosis code of 276.7 in inpatient claims | Cumulative incidence: 0.9 per 100 patients at risk | NR |
| Saito 2017 ([101](#_ENREF_101)) | Jul 2011-Dec 2015 | Overall | Patients with stage 3-5 non-dialysis dependent CKD | 986 | RAASi (ACEi/ARBs/MRAs/DRI) | sK+ ≥5.5 mEq/L | 12.3% | NR |
|  |  | Subgroup | Patients with stage 3a non-dialysis dependent CKD | 604 |  |  | 8.4% |  |
|  |  |  | Patients with stage 3b non-dialysis dependent CKD | 265 |  |  | 15.5% |  |
|  |  |  | Patients with stage 4-5 non-dialysis dependent CKD | 117 |  |  | 24.8% |  |
|  |  | Subgroup | Patients with stage 3-5 non-dialysis dependent CKD + DM | 501 |  |  | 12.6% |  |
| Fröhlich 2016 ([69](#_ENREF_69)) | Within 12 months of follow-up | Overall | Patients with stable stage 3 or 4 CKD + HF | 722 | ACEi/ARB | sK+ >5.5 mmol/L | 3.0% | NR |
| PHASE  Walsh 2015 ([20](#_ENREF_20)) | NR | Overall | Patients with ESRD on haemodialysis | 77 | MRA (eplerenone) | sK+ >6.0 mEq/L | 24.7% | NR |
|  | Within 4 weeks following treatment |  |  |  |  | sK+ >6.5 mEq/L | 11.7% |  |
|  | NR |  |  |  |  | sK+ >7.0 mEq/L | 5.2% |  |
|  | NR |  |  | 77 | Placebo | sK+ >6.0 mEq/L | 18.2% |  |
|  | Within 4 weeks following randomisation |  |  |  |  | sK+ >6.5 mEq/L | 2.6% |  |
|  | NR |  |  |  |  | sK+ >7.0 mEq/L | 0.0% |  |
| Lee 2014 ([103](#_ENREF_103)) | During 45.7 months of mean follow-up | Overall | Patients with stage 3 or 4 CKD with hyperkalaemia | 150 | Maintenance group (ACEi or ARB without discontinuation) | NR | Episode rate: 0.6 per patient-year | NR |
|  |  |  |  | 108 | Withdrawal group (ACEi or ARB discontinuation for >3 months after hyperkalaemia) |  | Episode rate: 0.5 per patient-year | NR |
| EVALUATE  Ando 2014 ([18](#_ENREF_18)) | 2009-2012 | Overall | Patients with stage 1-3 non-diabetic CKD + HT | 162 | MRA (eplerenone) | sK+ >5.5 mmol/L | 0.0% | NR |
|  |  |  |  | 152 | Placebo |  | 0.0% |  |
| Gwoo 2014 ([81](#_ENREF_81)) | 4 weeks following treatment | Overall | Patients with stage 2-4 CKD + HT | 553 | MRA (spironolactone) | sK+ ≥5.0 mEq/L | 50.4% | p=0,001 compared to non-CKD group |
|  | 8 weeks following treatment |  |  | 421 |  |  | 3.8% | p=0.371 compared to non-CKD group |
| ARTS-HF  Pitt 2013 ([26](#_ENREF_26)) | NR | Overall | Patients with mild (stage 2) CKD (eGFR 60 to 90 mL/min/1.73 m2) + HFpEF (Part A) | 16 | MRA (finerenone - 2.5 mg qd) | NR | 0.0% | NR |
|  | NR |  |  | 16 | MRA (finerenone - 5 mg qd) |  | 0.0% |  |
|  | Visit 5 (Day 22 ± 2) |  |  | 17 | MRA (finerenone - 10 mg qd) |  | 5.9% |  |
|  | NR |  |  | 16 | Placebo |  | 0.0% |  |
|  | NR |  | Patients with moderate (stage 3) CKD (eGFR 30 to 60 mL/min/1.73 m2) + HFpEF  (Part B) | 66 | MRA (finerenone - 2.5 mg qd) |  | 4.5% | NR |
|  | NR |  |  | 67 | MRA (finerenone - 5 mg qd) |  | 1.5% |  |
|  | NR |  |  | 67 | MRA (finerenone - 10 mg qd) |  | 4.5% |  |
|  | NR |  |  | 64 | MRA (finerenone - 5 mg bid) |  | 7.8% |  |
|  | NR |  |  | 63 | MRA (spironolactone - 25 or 50 mg qd) |  | 11.1% |  |
|  | NR |  |  | 65 | Placebo |  | 1.5% |  |
| EMPHASIS-HF  Eschalier 2013 ([25](#_ENREF_25)) | Within 18 months of treatment | Overall | Patients with CKD with eGFR <60 ml/min/1.73 m2 | 422 | MRA (eplerenone) | sK+ >5.5 mmol/L | 16.6% | p=0.002 |
|  |  |  |  | 461 | Placebo |  | 9.3% |  |
|  |  |  |  | 422 | MRA (eplerenone) | sK+ >6.0 mmol/L | 1.9% | p=0.29 |
|  |  |  |  | 461 | Placebo |  | 3.3% |  |
| Woo 2013 ([50](#_ENREF_50)) | Over 3 years of study period | Overall | Patients with stage 1-3 non-diabetic CKD | 49 | DRI (aliskiren) + ARB (losartan) | sK+ >5.0 mmol/L | 14.2% | p<0.001 |
|  |  |  |  | 46 | DRI (aliskiren) |  | 8.7% |  |
|  |  |  |  | 48 | ARB (High dose losartan) |  | 6.3% |  |
|  |  |  |  | 49 | DRI (aliskiren) + ARB (losartan) | Severe sK+ >6.0 mmol/L | 4.1% | p<0.07 |
|  |  |  |  | 46 | DRI (aliskiren) |  | 0.0% |  |
|  |  |  |  | 48 | ARB (High dose losartan) |  | 2.1% |  |
| Pisoni 2012 ([48](#_ENREF_48)) | Within median follow-up of 312 days | Overall | Patients with stage 3 CKD + Resistant HT | 36 | MRA (spironolactone/eplerenone) | sK+ >5.0 mEq/L | 22.0% | NR |
|  |  |  |  |  |  | sK+ >5.5 mEq/L | 8.0% | NR |
| Taheri 2012 ([14](#_ENREF_14)) | At month two of treatment | Overall | Patients on chronic ambulatory peritoneal dialysis + Advanced HF | 9 | MRA (spironolactone) | sK+ >5.5 mEq/L | 11.1% | NR |
|  |  |  |  | 9 | Placebo |  | NR |  |
| Espinel 2012 ([15](#_ENREF_15)) | Before cross-over | Overall | Patients with stage 3 CKD | 17 | ARB (olmesartan) | sK+ ≥5.0 mmol/L | 41.0% | NR |
|  |  |  |  | 13 | ACEi (enalapril) |  | 38.0% |  |
|  | After cross-over |  |  | 10 | ARB (olmesartan) |  | 42.0% | NR |
|  |  |  |  | 7 | ACEi (enalapril) |  | 42.0% |  |
|  | Overall |  |  | 17 | ARB (olmesartan) |  | 37.0% | NR |
|  |  |  |  | 13 | ACEi (enalapril) |  | 40.0% |  |
| CRIB-II  Edwards 2012 ([17](#_ENREF_17)) | Within 40 weeks of treatment | Overall | Patients with stage 2 or 3 CKD | 56 | MRA (spironolactone) | sK+ ≥6.0 mmol/L | 0.0% | NR |
|  |  |  |  |  |  | sK+ ≥5.5 mmol/L | 3.6% |  |
|  |  |  |  |  |  | sK+ ≥5.5 mmol/L (at least one occasion over 40 weeks of treatment) | 16.0% |  |
|  |  |  |  | 56 | Placebo | sK+ ≥6 mmol/L | 0.0% |  |
|  |  |  |  |  |  | sK+ ≥5.5 mmol/L | 3.6% |  |
|  |  |  |  |  |  | sK+ ≥5.5 mmol/L (at least one occasion over 40 weeks of treatment) | 3.6% |  |
| Iskandar 2012 ([62](#_ENREF_62)) | Jan 2011-Dec 2011 | Overall | Patients with ESRD undergoing haemodialysis | 44 | ACEi (captopril/lisinopril) or ARB (valsartan) | sK+ >5.3 mEq/L | 22.7% | NR |
| Abolghasmi 2011 ([24](#_ENREF_24)) | Within 12 weeks | Overall | Patients with moderately severe CKD (eGFR 25-50 mL/min/1.73 m²) + Resistant HT | 19 | MRA (spironolactone) | sK+ >5.5 mEq/L | 5.3% | NR |
|  |  |  |  | 22 | Placebo |  | NR |  |
| Johnson 2010 ([99](#_ENREF_99)) | within 90 days of treatment | Overall | Patients with stage 3-5 CKD | 5,171 | ACEi (lisinopril) | sK+ ≥5.5 mmol/L | Rate (95% CI): 2.8% (2.4-3.3) | NR |
| Raebel 2010 ([113](#_ENREF_113)) | 2001-2006 | Overall | Patients with stage 3 or 4 CKD + DM | 2,176 | ACEi or ARB or MRA (spironolactone) | sK+ ≥6 mmol/l and/or a coded diagnosis of hyperkalaemia (ICD-9 diagnostic code of 276.7) | 66.8 per 1000 person- years | NR |
|  |  |  |  | 1,482 | ACE or ARB alone or in combination |  | 41.3 per 1000 person- years |  |
|  |  |  |  | 42 | MRA (spironolactone) |  | 179.0 per 1000 person- years |  |
|  |  |  | Patients with stage 3 or 4 CKD + DM + HF | 541 | ACE or ARB alone or in combination |  | 115.1 per 1000 person- years |  |
|  |  |  |  | 112 | MRA (spironolactone) |  | 180.0 per 1000 person- years |  |
| Frimodt-Moller 2010 ([13](#_ENREF_13)) | 2005 | Overall | Pre-dialysis stage 3-5 CKD patients | 47 | ACEi (enalapril) + ARB (candesartan) after 16 weeks of monotherapy with either enalapril or candesartan | pK >5.5mmol/L | 15.0% | NR |
| Vukusich 2010 ([19](#_ENREF_19)) | 24 months | Overall | Patients with ESRD on haemodialysis | 30 | MRA (spironolactone) | NR | 0.0% |  |
|  |  |  |  | 23 | Placebo |  | 0.0% |  |
| Heshka 2010 ([104](#_ENREF_104)) | 2005-2009 | Overall | Patients with stage 3 or 4 CKD + Difficult-to-control HT | 34 | MRA (spironolactone) | sK+ ≥5.5 mmol/L | 5.7% | p=0.07 |
| Tokunaga 2010 ([136](#_ENREF_136)) | Within 15 months of observation period | Overall | Patients with Stage 3-4 CKD + HT | 36 | ARB (telmisartan) | NR | 0.0% | NR |
|  | Within 13.1 months of observation period |  |  | 36 | Control | NR | 2.8% |  |
| AVOID  Persson 2010 ([27](#_ENREF_27)) | NR | Overall | Patients with stage 1 CKD + HT + DM | 64 | DRI (aliskiren) | sK+ >6.0 mmol/L | 0.0% | NR |
|  |  |  |  | 51 | Placebo |  | 0.0% |  |
|  |  |  |  | 64 | DRI (aliskiren) | sK+ >5.5 mmol/L | 3.2% | p=1.000 |
|  |  |  |  | 51 | Placebo |  | 3.9% |  |
|  |  |  | Patients with stage 2 CKD + HT + DM | 104 | DRI (aliskiren) | sK+ >6.0 mmol/L | 2.9% | p=0.336 |
|  |  |  |  | 122 | Placebo |  | 0.8% |  |
|  |  |  |  | 104 | DRI (aliskiren) | sK+ >5.5 mmol/L | 9.6% | p=0.651 |
|  |  |  |  | 122 | Placebo |  | 11.5% |  |
|  |  |  | Patients with stage 3 CKD + HT + DM | 129 | DRI (aliskiren) | sK+ >6.0 mmol/L | 8.5% | p=0.113 |
|  |  |  |  | 119 | Placebo |  | 3.4% |  |
|  |  |  |  | 129 | DRI (aliskiren) | sK+ >5.5 mmol/L | 22.5% | p=0.007 |
|  |  |  |  | 119 | Placebo |  | 13.6% |  |
| AASK  Weinberg 2009 ([22](#_ENREF_22)) | NR | Overall | Patients with non-diabetic hypertensive CKD (eGFR 20-65 mL/min/1.73 m2) | 417 | ACEi (ramipril) | sK+ ≥5.5 mEq/L | Event rate (95% CI): 2.5 (1.7-3.5) per 100 patient-years | NR |
|  |  |  |  | 428 | β-Blocker (metoprolol succinate) |  | Event rate (95% CI): 1.3 (0.8-2.1) per 100 patient-years | NR |
|  |  |  |  | 208 | CCB (amlodipine besylate) |  | Event rate (95% CI): 0.7 (0.2-1.7) per 100 patient-years | NR |
|  |  | Subgroup | Patients with non-diabetic hypertensive CKD with baseline eGFR ≤30 mL/min/1.73 m2 | NR | ACEi (ramipril) |  | Event rate (95% CI): 8.6 (4.2-14.9) per 100 patient-years | NR |
|  |  |  |  |  | β-Blocker (metoprolol succinate) |  | Event rate (95% CI): 6.7 (3.3-12.5) per 100 patient-years | NR |
|  |  |  |  |  | CCB (amlodipine besylate) |  | Event rate (95% CI): 3.0 (0.4-11.2) per 100 patient-years | NR |
|  |  |  | Patients with non-diabetic hypertensive CKD with baseline eGFR >30 to ≤40 mL/min/1.73 m2 + HT |  | ACEi (ramipril) |  | Event rate (95% CI): 4.5 (2.2-8.9) per 100 patient-years | NR |
|  |  |  |  |  | β-Blocker (metoprolol succinate) |  | Event rate (95% CI): 1.6 (0.5-4.4) per 100 patient-years | NR |
|  |  |  |  |  | CCB (amlodipine besylate) |  | Event rate (95% CI): 1.7 (0.3-6.6) per 100 patient-years | NR |
|  |  |  | Patients with non-diabetic hypertensive CKD with baseline eGFR >40 to ≤50 mL/min/1.73 m2 + HT |  | ACEi (ramipril) |  | Event rate (95% CI): 1.0 (0.3-3.2) per 100 patient-years | NR |
|  |  |  |  |  | β-Blocker (metoprolol succinate) |  | Event rate (95% CI): NR (NR-1.5) per 100 patient-years | NR |
|  |  |  |  |  | CCB (amlodipine besylate) |  | Event rate (95% CI): NR (NR-2.7) per 100 patient-years | NR |
|  |  |  | Patients with non-diabetic hypertensive CKD with baseline eGFR >50 mL/min/1.73 m2 + HT |  | ACEi (ramipril) |  | Event rate (95% CI): 0.7 (0.3-2.0) per 100 patient-years | NR |
|  |  |  |  |  | β-Blocker (metoprolol succinate) |  | Event rate (95% CI): 0.3 (0.2-1.4) per 100 patient-years | NR |
|  |  |  |  |  | CCB (amlodipine besylate) |  | Event rate (95% CI): NR (NR-1.3) per 100 patient-years | NR |
| Sengul 2009 ([93](#_ENREF_93)) | Within 8 weeks of treatment | Overall | Patients with CKD | 33 | MRA (spironolactone) | sK+ ≥5.5 mEq/L | 18.2% | NR |
| Khosla 2009 ([68](#_ENREF_68)) | NR | Overall | Patients with stage 2 or 3 CKD + Resistant HT | 46 | ACEi (lisinopril/ramipril) or ARB (valsartan/olmesartan) or ACEi + ARB | sK+ >5.5 mEq/l | 17.3% | NR |
|  |  |  |  |  |  | sK+ ≥6.0 mEq/l | 2.2% | NR |
| Einhorn 2009 ([111](#_ENREF_111)) | 2005 | Overall | Patients with stage 3-5 CKD | 46,463 | ACEi and/or ARB | sK+ ≥5.5 mg/dL | Rate (95% CI): 7.7 (7.6-7.8) per 100 patient months | p<0.0001 |
|  |  |  |  |  |  | sK+ ≥5.5 to <6.0 mg/dL | Rate (95% CI): 5.1 (5.0-5.1) per 100 patient months |  |
|  |  |  |  |  |  | sK+ ≥6.0 mg/dL | Rate (95% CI): 2.6 (2.5-2.7) per 100 patient months |  |
|  |  |  |  | 24,410 | No RAASi | sK+ ≥5.5 mg/dL | Rate (95% CI): 8.2 (8.1-8.4) per 100 patient months | p<0.0001 |
|  |  |  |  |  |  | sK+ ≥5.5 to <6.0 mg/dL | Rate (95% CI): 5.4 (5.3-5.6) per 100 patient months |  |
|  |  |  |  |  |  | sK+ ≥6.0 mg/dL | Rate (95% CI): 2.8 (2.7-2.9) per 100 patient months |  |
| Maddirala 2008 ([100](#_ENREF_100)) | Within 52 weeks of follow-up | Overall | Patients with stage 1 to 5 CKD | 931 | ACEi/ARB + Combination drugs | sK+>5.5 mmol/L | 2.5% | NR |
|  |  |  |  | 395 | ACEi/ARB only |  | 1.5% | NR |
|  |  |  |  | 140 | ACEi/ARB + β blocker |  | 0.0% | NR |
|  |  |  |  | 100 | ACEi/ARB + NSAID |  | 0.0% | NR |
|  |  |  |  | 24 | ACEi/ARB + Insulin |  | 6.6% | NR |
|  |  |  |  | 15 | ACEi/ARB + K+ supplements |  | 0.0% | NR |
|  |  |  |  | 142 | ACEi/ARB + K+ depleting diuretics |  | 3.5% | NR |
|  |  |  |  | 6 | ACEi/ARB + Calcineurin inhibitors |  | 16.7% | NR |
|  |  | Subgroup | Patients with stage 1 CKD | 86 | ACEi/ARB + Combination drugs |  | 1.2% | NR |
|  |  |  | Patients with stage 2 CKD | 469 |  |  | 1.1% | p<0.05 vs stage 3 and 4 CKD |
|  |  |  | Patients with stage 3 CKD | 318 |  |  | 3.1% | p<0.05 |
|  |  |  | Patients with stage 4 CKD | 51 |  |  | 13.7% |  |
|  |  |  | Patients with stage 5 CKD | 7 |  |  | 0.0% | NR |
| Hayashi 2003 ([23](#_ENREF_23)) | Within 48 weeks of treatment | Overall | Patients with chronic renal parenchymal disease + HT | 20 | ACEi (enalapril/lisinopril/imidapril) | NR | 25.0% | NR |
|  |  |  |  | 23 | CCB (efonidipine) |  | 0.0% | NR |
| Knoll 2002 ([92](#_ENREF_92)) | 1st May 1999-2nd Dec 1999 | Overall | Patients with ESRD on chronic haemodialysis | 71 | ACEi (enalapril/fosinopril/captopril) or ARB (losartan) | sK+ ≥5mmol/L | Episode rate: 26.0 per 100 person-months | NR |
|  |  |  |  | 180 | Control |  | Episode rate: 17.0 per 100 person-months | NR |
| Ruilope 2000 ([3](#_ENREF_3)) | After Visit 2 | Overall | Patients with progressive CKD with or without proteinuria and HT | 22 | ARB (valsartan - 160 mg) | sK+ ≥6.0 mmol/L | 4.5% | NR |
|  |  |  |  | 42 | ARB (valsartan - 80 mg) + ACEi (benazepril - 5 or 10 mg) |  | 11.9% | NR |
|  |  |  |  | 44 | ARB (valsartan - 160 mg) + ACEi (benazepril - 5 or 10 mg) |  | 4.5% | NR |
| Mårup 2023 ([73](#_ENREF_73)) | NR | Overall | Patients with CKD on maximal tolerated RAASi (ACEi or ARB) with history of at least two hyperkalaemia episodes | 58 | MRA (spironolactone) | pK+ >5.5mmol/L | 29.3% | NR |
|  | NR |  |  |  |  | pK+ >6.2mmol/L | 6.9% | NR |
| Zhou 2023b ([29](#_ENREF_29)) | NR | Overall | Patients with stage 3b-5 CKD + HT | 44 | ARNi (sacubitril/valsartan) | sK+ ≥5.5 mmol/L | 4.5% | NR |
|  | NR |  |  | 40 | Conventional antihypertensive |  | 7.5% | NR |
| Rajak 2023 ([141](#_ENREF_141)) | 30th June 2022 | Overall | Patients with CKD + DM | NR | ARB (finerenone) | NR | 9% | NR |
| Bornstein 2024 ([30](#_ENREF_30)) | NR | Overall | Patients with albuminuric CKD + DM | 18 | MRA (BI 690517 3 mg) | sK+ >5.0 mEq/L | 5.6% | NR |
|  | NR |  |  | 13 | MRA (BI 690517 10 mg) |  | 7.7% | NR |
|  | NR |  |  | 14 | MRA (BI 690517 40 mg) |  | 0% | NR |
|  | NR |  |  | 9 | Placebo |  | 0% | NR |
| PROERCAN Garcia-Prieto 2024 ([31](#_ENREF_31)) | 3-year follow- up | Overall | Patients with stages 3-4 CKD without proteinuria + HT | 40 | ACEi | sK+ 5.7 mmol/L | 2.5% | NR |
|  |  |  |  | 48 | Non ACEi | NR | 0% | NR |
| Gregg 2023 ([143](#_ENREF_143)) | NR | Overall | Patients with stage 3-4 CKD | 1,209,235 | ACEi or ARB: current or past user | NR | 0.2% | NR |
|  | NR | Subgroup | Patients with stage 3a CKD | 868,168 |  | NR | 0.2% | NR |
|  | NR |  | Patients with stage 3b CKD | 281,921 |  | NR | 0.2% | NR |
|  | NR |  | Patients with stage 4 CKD | 59,146 |  | NR | 0.3% | NR |
| Nicholas 2023 ([144](#_ENREF_144)) | Interim Results: data cut-off date: April 28, 2023 | Overall | Patients with CKD + type 2 diabetes | 574 | MRA (Finerenone) | NR | 3% | NR |
| McFarland 2023 ([57](#_ENREF_57)) | 180 days | Overall | Patients with stage 3-5 CKD + HF | 50 | ARNi (Sacubitril/Valsartan) | sK+ >5.0 mEq/L | 12% | NR |
|  |  |  |  |  |  | sK+ >5.5 mEq/L | 4% | NR |
|  |  |  |  |  |  | sK+ >6.0 mEq/L | 0% | NR |
| Ding 2023 ([58](#_ENREF_58)) | January 2015 to April 2022 | Overall | Patients with ESRD undergoing maintenance dialysis + HT | 51 | ARNi (Sacubitril/Valsartan) | sK+ >5.0 mmol/L | 27.5% | NR |
| Jariwala 2023 ([145](#_ENREF_145)) | August 2022 to December 2022 | Overall | Patients with CKD + DM | 42 | ARB (Finerenone) | NR | 1.3% | NR |
|  |  |  |  | 44 | No finerenone |  | 0.9% | NR |
| Tuttle 2024 ([32](#_ENREF_32)) | NR | Overall | Patients with CKD | 147 | Pooled** BI 690517 3 mg | NR | 10% | NR |
|  |  |  |  | 146 | Pooled** BI 690517 10 mg | NR | 15% | NR |
|  |  |  |  | 146 | Pooled** BI 690517 20 mg | NR | 18% | NR |
|  |  |  |  | 147 | Pooled** BI 690517 placebo | NR | 6% | NR |

*To comprehensively understand the specific impact of RAASi treatment on patients with CKD, non-RAASi treatment comparisons were also included in the review, **Pooled groups include participants who received BI 690517 either as monotherapy or in combination with empagliflozin.

**Abbreviations:** β: beta; ACEi: angiotensin converting enzyme inhibitors; ARB: angiotensin receptor blocker; ARNi: angiotensin receptor/neprilysin inhibitors; bid: twice a day; CCB: calcium channel blocker; CI: confidence interval; CKD: chronic kidney disease; CVD: cardiovascular disease; DM: diabetes mellitus; DRI: direct renin inhibitors; eGFR: estimated glomerular filtration rate; ESRD: end stage renal disease; HbA1C: haemoglobin A1C; HF: heart failure; HFpEF: heart failure with preserved ejection fraction; HFrEF: heart failure with reduced ejection fraction; HT: hypertension; ICD-10: International Classifications of Diseases, Tenth Revision; ICD-9; International Classifications of Diseases, Ninth Revision; IQR: interquartile range; m2: square metre; mEq/L: milliequivalents per litre; mg/gCr: milligram per gram creatinine; mg: milligram; mL/min: millilitres per minute; mmol/L: millimoles per litre; MRA: mineralocorticoid receptor antagonist; K+: potassium; NA: not applicable; NR: not reported; NSAIDs: non-steroidal anti-inflammatory drugs; pK+: plasma potassium; qd; once daily; RAASi: renin-angiotensin-aldosterone system inhibitors; SAE: serious adverse event; SGLT2i: sodium/glucose cotransporter-2 inhibitors; sK+: serum potassium; UACR: urinary albumin-to-creatinine ratio; USA: United States of America; vs: versus

Table S20. Hospitalisations and length of stay among CKD patients

| Study | Study setting | Population type | Population description | Sample size | Definition | Hospitalisations | | | Length of stay | |
| --- | --- | --- | --- | --- | --- | --- | --- | --- | --- | --- |
|  |  |  |  |  |  | **Time point** | **No. of hospitalisations** | **Hospitalisation rate** | **Time point** | **Value** |
| Li 2023a ([65](#_ENREF_65)) | Baoding No. 1, Central Hospital of Hebei Medical University | Overall | Patients with stage 4-5 CKD + DM + Hyperkalaemia | 120 | NR | NR | NR | NR | Baseline | Median (range): 12.1 (8.2-16.5) days |
|  |  |  | Patients with stage 4-5 CKD + DM + Normokalaemia | 150 | NR | NR | NR | NR |  | Median (range): 7.4 (5.3-11.2) days |
| Neuenschwander 2023 ([116](#_ENREF_116)) | Medicare FFS claims database | Overall | Patients with CKD/ESRD + Hyperkalaemia (matched cohort) | 134,241 | NR | During long-term care stay | Mean (SD), visits per patient: 0.5 (0.9) | 32% | During long-term care stay | Mean (SD), per episode: 6.8 (5.1) days |
|  |  |  | Patients with CKD/ESRD + Normokalaemia (matched cohort) | 126,205 | NR |  | Mean (SD), visits per patient: 0.2 (0.6) | 18% |  | Mean (SD), per episode: 6.2 (4.9) days |
|  |  |  | Patients with CKD/ESRD + Hyperkalaemia (matched cohort) | 59,455 | NR | Up to 365 days post-discharge | Mean (SD), visits per patient: 1.5 (1.9) | 60% | Up to 365 days post-discharge | Mean (SD), per episode: 5.6 (5.2) days |
|  |  |  | Patients with CKD/ESRD + Normokalaemia (matched cohort) | 58,359 | NR |  | Mean (SD), visits per patient: 0.9 (1.3) | 47% |  | Mean (SD), per episode: 4.9 (4.4) days |
| Calabrese 2022 ([60](#_ENREF_60)) | Unit of Nephrology and Dialysis of the University Hospital of Messina | Overall | Patients with stage 1-5 or A1-A3 CKD or with other anatomic or urine sediment abnormalities according to the KDIGO 2012 Clinical Practice Guideline for the Evaluation and Management of CKD + Hyperkalaemia | 90 | Sum of hospitalisation days (number of days on which the patients were hospitalised; Added up the days of hospitalisation for all admissions for each patient) | NR | NR | NR | Baseline | Median (IQR): 11 (7-15) days |
|  |  |  | Patients with stage 1-5 or A1-A3 CKD or with other anatomic or urine sediment abnormalities according to the KDIGO 2012 Clinical Practice Guideline for  the Evaluation and Management of CKD + Normokalaemia | 180 |  | NR | NR | NR |  | Median (IQR): 8 (6-10) days |
|  |  | Subgroup | Patients with stage 4-5 CKD + Hyperkalaemia | 59 |  | NR | NR | NR |  | Median (IQR): 10 (9-17) days |
|  |  |  | Patients with stage 4-5 CKD + Normokalaemia | 84 |  | NR | NR | NR |  | Median (IQR): 9 (7-15) days |
|  |  |  | CKD patients with eGFR 6-30 mL/min/1.73 m2 + Hyperkalaemia | 46 |  | NR | NR | NR |  | Median (IQR): 10 (9-17) days |
|  |  |  | CKD patients with eGFR 6-30 mL/min/1.73 m2) + Normokalaemia | 75 |  | NR | NR | NR |  | Median (IQR): 9 (7-15) days |
|  |  |  | Patients with stage 1-5 or A1-A3 CKD + HT + Hyperkalaemia | 78 |  | NR | NR | NR |  | Median (IQR): 11 (7-15) days |
|  |  |  | Patients with stage 1-5 or A1-A3 CKD + HT + Normokalaemia | 139 |  | NR | NR | NR |  | Median (IQR): 8 (6-11) days |
|  |  |  | Patients with stage 1-5 or A1-A3 CKD without HT + Hyperkalaemia | 12 |  | NR | NR | NR |  | Median (IQR): 12 (7-15) days |
|  |  |  | Patients with stage 1-5 or A1-A3 CKD without HT + Normokalaemia | 41 |  | NR | NR | NR |  | Median (IQR): 7 (7-15) days |
| Zhang 2022a ([36](#_ENREF_36)) | UEBMI claims | Overall | Patients with CKD having ≥1 inpatient or outpatient visit + Hyperkalaemia (Before propensity score matching) | 1,015 | Patients having ≥1 inpatient visit | Baseline | Mean (SD): 1.3 (1.8) | NR | Baseline | Mean (SD): 18.7 (31.4) days |
|  |  |  | Patients with CKD having ≥1 inpatient or outpatient visit + Normokalaemia (Before propensity score matching) | 25,981 |  |  | Mean (SD): 0.4 (1.0) | NR |  | Mean (SD): 5.1 (14.7) days |
|  |  |  | Patients with CKD having ≥1 inpatient or outpatient visit + Hyperkalaemia (After propensity score matching) | 1,003 |  |  | Mean (SD): 1.3 (1.7) | NR |  | Mean (SD): 18.1 (30.7) days |
|  |  |  | Patients with CKD having ≥1 inpatient or outpatient visit + Normokalaemia (After propensity matching) | 1,003 |  |  | Mean (SD): 1.3 (2.2) | NR |  | Mean (SD): 16.8 (30.7) days |
|  |  |  | Patients with CKD having ≥1 inpatient or outpatient visit + Hyperkalaemia (After propensity score matching) | 1,003 |  | 12-Month follow up | Mean (95% CI), number of admissions per patient: 1.9 (1.8-2.1) | 86.5% | 12-month follow up | Mean (95% CI), total length of stay per patient: 28.6 (25-31.7) days |
|  |  |  | Patients with CKD having ≥1 inpatient or outpatient visit + Normokalaemia (After propensity score matching) | 1,003 |  |  | Mean (95% CI), number of admissions per patient: 0.7 (0.5-0.9) | 32.1% |  | Mean (95% CI), total length of stay per patient: 8.7 (6.5-10.5) days |
| DISCOVER CKD  James 2021 ([40](#_ENREF_40)) | 1. USA TriNetX hospital-EMR  2. UK CPRD linked to hospital data  3. USA DOPPS  4. JMDV databases | Overall | Patients with stage 3 CKD + Hyperkalaemia (From UK CPRD database) | 24,365 | NR | NR | NR | Rate (95% CI): 71 (69.8-72.3) per 100 person years | NR | Median (IQR): 6 (2-20) days |
|  |  |  | Patients with stage 3 CKD + Normokalaemia (From UK CPRD database) | 24,365 | NR | NR | NR | Rate (95% CI): 53.6 (52.7-54.5) per 100 person years |  | Median (IQR): 6 (2-20) days |
|  |  |  | Patients with stage 3 CKD + Hyperkalaemia (From Japan MDV database) | 3,161 | NR | NR | NR | Rate (95% CI): 47.6 (45.5-49.9) per 100 person years |  | Median (IQR): 22 (9-49) days |
|  |  |  | Patients with stage 3 CKD + Normokalaemia (From Japan MDV database) | 3,161 | NR | NR | NR | Rate (95% CI): 41.5 (39.7-43.3) per 100 person years |  | Median (IQR): 15 (6-36) days |
|  |  |  | Patients with stage 3 CKD + Hyperkalaemia (From USA LCED database) | 1,609 | NR | NR | NR | Rate (95% CI): 30.7 (28.8-32.8) per 100 person years |  | Median (IQR): 6 (3-11) days |
|  |  |  | Patients with stage 3 CKD + Normokalaemia (From USA LCED database) | 1,609 | NR | NR | NR | Rate (95% CI): 28.3 (26.6-30.1) per 100 person years |  | Median (IQR): 5 (3-9) days |
|  |  |  | Patients with stage 3 CKD + Hyperkalaemia (From USA TriNetX database) | 46,420 | NR | NR | NR | Rate (95% CI): 101.4 (100.8-102.1) per 100 person years |  | Median (IQR): 10 (4-27) days |
|  |  |  | Patients with stage 3 CKD + Normokalaemia (From USA TriNetX database) | 46,420 | NR | NR | NR | Rate (95% CI): 46.8 (46.4-47.3) per 100 person years |  | Median (IQR): 7 (3-15) days |
| Jime´nez-Marrero 2021 ([44](#_ENREF_44)) | This study used administrative, hospital and primary care databases | Overall | Patients with CKD + Hyperkalaemia (Prevalent case analysis) | 415 | NR | NR | Median (range): 1.1 (1.0-1.4) | NR | NR | NR |
|  |  |  | Patients with CKD + Hypokalaemia (Prevalent case analysis) | 80 | NR | NR | Median (range): 1.2 (0.8-1.8) | NR | NR | NR |
|  |  |  | Patients with CKD + Hyperkalaemia (Incident case analysis) | 82 | NR | NR | Median (range): 1.2 (1.0-1.5) | NR | NR | NR |
|  |  |  | Patients with CKD + Hypokalaemia (Incident case analysis) | 37 | NR | NR | NR | NR | NR | NR |
|  |  |  | Patients with CKD + Hyperkalaemia (prevalent case-prevalent (RAASI) user analysis) | 263 | NR | NR | Median (range): 1.2 (1.0-1.5) | NR | NR | NR |
|  |  |  | Patients with CKD + Hypokalaemia (prevalent case-prevalent (RAASI) user analysis) | 38 | NR | NR | NR | NR | NR | NR |
| Kohsaka 2021 ([86](#_ENREF_86)) | Hospital based cohort study. Data were obtained using a Japanese hospital claims registry, Medical Data Vision | Overall | Patients with stage 1-5 CKD + Hyperkalaemia + Hyperkalaemia (After matching) | 5,859 | Hospitalisation for HF | Year 1 after index | NR | Cumulative incidence, 5.3%, | NR | NR |
|  |  |  | Patients with stage 1-5 CKD + Normokalaemia (After matching) | 5,859 |  |  | NR | Cumulative incidence, 1.1% | NR | NR |
|  |  |  | Patients with stage 1-5 CKD + Hyperkalaemia (After matching) | 5,859 |  | Year 2 after index | NR | Cumulative incidence, 6.9% | NR | NR |
|  |  |  | Patients with stage 1-5 CKD + Normokalaemia (After matching) | 5,859 |  |  | NR | Cumulative incidence, 1.3% | NR | NR |
|  |  |  | Patients with stage 1-5 CKD + Hyperkalaemia (After matching) | 5,859 |  | Year 3 after index | NR | Cumulative incidence, 8.4% | NR | NR |
|  |  |  | Patients with stage 1-5 CKD + Normokalaemia (After matching) | 5,859 |  |  | NR | Cumulative incidence, 1.6% | NR | NR |
|  |  |  | Patients with stage 1-5 CKD + Hyperkalaemia (After matching) | 5,859 |  | Year 4 after index | NR | Cumulative incidence, 10.1% | NR | NR |
|  |  |  | Patients with stage 1-5 CKD + Normokalaemia (After matching) | 5,859 |  |  | NR | Cumulative incidence, 2% | NR | NR |
|  |  |  | Patients with stage 1-5 CKD + Hyperkalaemia (After matching) | 5,859 |  | Year 5 after index | NR | Cumulative incidence, 11.3% | NR | NR |
|  |  |  | Patients with stage 1-5 CKD + Normokalaemia (After matching) | 5,859 |  |  | NR | Cumulative incidence, 2.4% | NR | NR |
|  |  |  | Patients with stage 1-5 CKD + Hyperkalaemia (After matching) | 5,859 |  | Year 6 after index | NR | Cumulative incidence, 12.8% | NR | NR |
|  |  |  | Patients with stage 1-5 CKD + Normokalaemia (After matching) | 5,859 |  |  | NR | Cumulative incidence, 2.7% | NR | NR |
|  |  |  | Patients with stage 1-5 CKD + Hyperkalaemia (After matching) | 5,859 | Hospitalisation for cardiac event | Year 1 after index | NR | Cumulative incidence, 2.9% | NR | NR |
|  |  |  | Patients with stage 1-5 CKD + Normokalaemia (After matching) | 5,859 |  |  | NR | Cumulative incidence, 1.3% | NR | NR |
|  |  |  | Patients with stage 1-5 CKD + Hyperkalaemia (After matching) | 5,859 |  | Year 2 after index | NR | Cumulative incidence, 3.5% | NR | NR |
|  |  |  | Patients with stage 1-5 CKD + Normokalaemia (After matching) | 5,859 |  |  | NR | Cumulative incidence, 1.8% | NR | NR |
|  |  |  | Patients with stage 1-5 CKD + Hyperkalaemia (After matching) | 5,859 |  | Year 3 after index | NR | Cumulative incidence, 4.2% | NR | NR |
|  |  |  | Patients with stage 1-5 CKD + Normokalaemia (After matching) | 5,859 |  |  | NR | Cumulative incidence, 2.2% | NR | NR |
|  |  |  | Patients with stage 1-5 CKD + Hyperkalaemia (After matching) | 5,859 |  | Year 4 after index | NR | Cumulative incidence, 4.7% | NR | NR |
|  |  |  | Patients with stage 1-5 CKD + Normokalaemia (After matching) | 5,859 |  |  | NR | Cumulative incidence, 2.7% | NR | NR |
|  |  |  | Patients with stage 1-5 CKD + Hyperkalaemia (After matching) | 5,859 |  | Year 5 after index | NR | Cumulative incidence, 5.2% | NR | NR |
|  |  |  | Patients with stage 1-5 CKD + Normokalaemia (After matching) | 5,859 |  |  | NR | Cumulative incidence, 3% | NR | NR |
|  |  |  | Patients with stage 1-5 CKD + Hyperkalaemia (After matching) | 5,859 |  | Year 6 after index | NR | Cumulative incidence, 5.9% | NR | NR |
|  |  |  | Patients with stage 1-5 CKD + Normokalaemia (After matching) | 5,859 |  |  | NR | Cumulative incidence, 3.1% | NR | NR |
| Kanda 2020 ([87](#_ENREF_87)) | Data were obtained using a Japanese hospital claims registry, Medical Data Vision | Overall | Patients with stage 1-5 CKD + Hyperkalaemia | 16,133 | NR | Within 12 months | Mean (SD), number of hospitalisations per patient: 1.7 (1.2) | Patients requiring hospitalisation: 49%  Patients requiring rehospitalisation: 10% | Within 12 months of first hyperkalaemia episode | Mean (SD), length of hospital stay per hospitalisation: 23.4 (35.6) days |
|  |  |  | Patients with stage 1-5 CKD + Normokalaemia | 15,287 |  |  | Mean (SD), number of hospitalisations per patient: 1.4 (0.8) | Patients requiring hospitalisation: 17%  Patients requiring rehospitalisation: 2% |  | Mean (SD), length of hospital stay per hospitalisation: 11.6 (18.5) days |
|  |  |  | Patients with stage 1-5 CKD + Hyperkalaemia | 14,193 |  | After 12 months | Mean (SD), number of hospitalisations per patient: 1.1 (1.0) | Patients requiring hospitalisation: 56.7%  Patients requiring rehospitalisation: 14.6% | After 12 months of first hyperkalaemia episode | Mean (SD), per hospitalisation: 21 (31.8) days |
|  |  |  | Patients with stage 1-5 CKD + Normokalaemia | 15,159 |  |  | Mean (SD), number of hospitalisations per patient: 0.6 (0.5) | Patients requiring hospitalisation: 24%  Patients requiring rehospitalisation: 3% |  | Mean (SD), per hospitalisation: 11.7 (18) days |
| Polson 2019 ([118](#_ENREF_118)) | Regional health plans | Overall | Patients with CKD + Hyperkalaemia | 321 | NR | NR | Mean (SD): 1.4 (0.8); Median: 1 | NR | Jan 1, 2014, to Dec 31, 2015 | Mean (SD): 5.3 (3.6) days |
|  |  |  | Patients with CKD + Normokalaemia | 3,102 | NR |  | Mean (SD): 1.3 (0.7); Median: 1 | NR |  | Mean (SD): 6.1 (6.5) days |
|  |  |  | Patients with CKD + HF + Hyperkalaemia | 178 | NR |  | Mean (SD): 2.8 (2.6); Median: 2 | NR |  | Mean (SD): 5.5 (2.1) days |
|  |  |  | Patients with CKD + HF + Normokalaemia | 808 | NR |  | Mean (SD): 1.6 (0.9) Median: 1 | NR |  | Mean (SD): 5.4 (2.9) days |
| Thomsen 2018 ([124](#_ENREF_124)) | Danish National Patient Registry | Overall | Patients with stage 1-5 CKD or on dialysis + Hyperkalaemia | 43,397 | Any acute hospitalisation | 6 months before hyperkalaemia event | NR | 799 per 1000 person years; HR (95% CI): 1.8 (1.7-1.8)* | NR | NR |
|  |  |  | Patients with stage 1-5 CKD or on dialysis + Normokalaemia | 43,397 |  | 6 months before index date | NR | 451 per 1000 person years | NR | NR |
|  |  |  | Patients with stage 1-5 CKD or on dialysis + Hyperkalaemia | 43,397 | Any non-acute hospitalisation | 6 months before hyperkalaemia event | NR | 362 per 1000 person years; HR (95% CI): 1.6 (1.5-1.7)* | NR | NR |
|  |  |  | Patients with stage 1-5 CKD or on dialysis + Normokalaemia | 43,397 |  | 6 months before index date | NR | 227 per 1000 person years | NR | NR |
|  |  |  | Patients with stage 1-5 CKD or on dialysis + Hyperkalaemia | 43,397 | Hospitalisation with any cardiac diagnosis | 6 months before hyperkalaemia event | NR | 373 per 1000 person years; HR (95% CI): 1.7 (1.6-1.7)* | NR | NR |
|  |  |  | Patients with stage 1-5 CKD or on dialysis + Normokalaemia | 43,397 |  | 6 months before index date | NR | 226 per 1000 person years | NR | NR |
|  |  |  | Patients with stage 1-5 CKD or on dialysis + Hyperkalaemia | 43,397 | Hospitalisation with ventricular arrhythmia | 6 months before hyperkalaemia event | NR | 18.6 per 1000 person years; HR (95% CI): 1.5 (1.3-1.8)* | NR | NR |
|  |  |  | Patients with stage 1-5 CKD or on dialysis + Normokalaemia | 43,397 |  | 6 months before index date | NR | 12.4 per 1000 person years | NR | NR |
|  |  |  | Patients with stage 1-5 CKD or on dialysis + Hyperkalaemia | 43,397 | Hospitalisation with cardiac arrest | 6 months before hyperkalaemia event | NR | 1.4 per 1000 person years; HR (95% CI): 1.7 (0.9-3.0)* | NR | NR |
|  |  |  | Patients with stage 1-5 CKD or on dialysis + Normokalaemia | 43,397 |  | 6 months before index date | NR | 0.8 per 1000 person years | NR | NR |
|  |  |  | Patients with stage 1-5 CKD or on dialysis + Hyperkalaemia | 43,397 | Hospitalisation with dialysis procedure | 6 months before hyperkalaemia event | NR | 9.3 per 1000 person years; HR (95% CI): 2.3 (1.8-3.0)* | NR | NR |
|  |  |  | Patients with stage 1-5 CKD or on dialysis + Normokalaemia | 43,397 |  | 6 months before index date | NR | 4 per 1000 person years | NR | NR |
|  |  |  | Patients with stage 1-5 CKD or on dialysis + Hyperkalaemia | 43,397 | Hospitalisation with ventilator treatment | 6 months before hyperkalaemia event | NR | 14.4 per 1000 person years; HR (95% CI): 1.8 (1.5–2.1)* | NR | NR |
|  |  |  | Patients with stage 1-5 CKD or on dialysis + Normokalaemia | 43,397 |  | 6 months before index date | NR | 8.3 per 1000 person years | NR | NR |
|  |  |  | Patients with stage 1-5 CKD or on dialysis + Hyperkalaemia | 43,397 | Hospitalisation with ICU admission | 6 months before hyperkalaemia event | NR | 42.6 per 1000 person years; HR (95% CI): 1.6 (1.5-1.8)* | NR | NR |
|  |  |  | Patients with stage 1-5 CKD or on dialysis + Normokalaemia | 43,397 |  | 6 months before index date | NR | 26.2 per 1000 person years | NR | NR |
|  |  |  | Patients with stage 1-5 CKD or on dialysis + Hyperkalaemia | 43,397 | Any acute hospitalisation | 6 months before hyperkalaemia event | NR | 2332 per 1000 person years; HR (95% CI): 3.8 (3.7-3.9)* | NR | NR |
|  |  |  | Patients with stage 1-5 CKD or on dialysis + Normokalaemia | 43,397 |  | 6 months before index date | NR | 515 per 1000 person years | NR | NR |
|  |  |  | Patients with stage 1-5 CKD or on dialysis + Hyperkalaemia | 43,397 | Any non-acute hospitalisation | 6 months before hyperkalaemia event | NR | 642 per 1000 person years; HR (95% CI): 2.6 (2.5-2.7)* | NR | NR |
|  |  |  | Patients with stage 1-5 CKD or on dialysis + Normokalaemia | 43,397 |  | 6 months before index date | NR | 232 per 1000 person years | NR | NR |
|  |  |  | Patients with stage 1-5 CKD or on dialysis + Hyperkalaemia | 43,397 | Hospitalisation with any cardiac diagnosis | 6 months before hyperkalaemia event | NR | 940 per 1000 person years; HR (95% CI): 3.4 (3.3–3.5)* | NR | NR |
|  |  |  | Patients with stage 1-5 CKD or on dialysis + Normokalaemia | 43,397 |  | 6 months before index date | NR | 247 per 1000 person years | NR | NR |
|  |  |  | Patients with stage 1-5 CKD or on dialysis + Hyperkalaemia | 43,397 | Hospitalisation with ventricular arrhythmia | 6 months before hyperkalaemia event | NR | 41.9 per 1000 person years; HR (95% CI): 3.4 (3.0-4.0)* | NR | NR |
|  |  |  | Patients with stage 1-5 CKD or on dialysis + Normokalaemia | 43,397 |  | 6 months before index date | NR | 11.5 per 1000 person years | NR | NR |
|  |  |  | Patients with stage 1-5 CKD or on dialysis + Hyperkalaemia | 43,397 | Hospitalisation with cardiac arrest | 6 months before hyperkalaemia event | NR | 16.4 per 1000 person years; HR (95% CI): 5.4 (4.1-7.2)* | NR | NR |
|  |  |  | Patients with stage 1-5 CKD or on dialysis + Normokalaemia | 43,397 |  | 6 months before index date | NR | 2.8 per 1000 person years | NR | NR |
|  |  |  | Patients with stage 1-5 CKD or on dialysis + Hyperkalaemia | 43,397 | Hospitalisation with dialysis procedure | 6 months before hyperkalaemia event | NR | 38.3 per 1000 person years; HR (95% CI): 4.5 (3.8-5.4)* | NR | NR |
|  |  |  | Patients with stage 1-5 CKD or on dialysis + Normokalaemia | 43,397 |  | 6 months before index date | NR | 8.1 per 1000 person years | NR | NR |
|  |  |  | Patients with stage 1-5 CKD or on dialysis + Hyperkalaemia | 43,397 | Hospitalisation with ventilator treatment | 6 months before hyperkalaemia event | NR | 163 per 1000 person years; HR (95% CI): 9.6 (8.6-10.8)* | NR | NR |
|  |  |  | Patients with stage 1-5 CKD or on dialysis + Normokalaemia | 43,397 |  | 6 months before index date | NR | 15.7 per 1000 person years | NR | NR |
|  |  |  | Patients with stage 1-5 CKD or on dialysis + Hyperkalaemia | 43,397 | Hospitalisation with ICU admission | 6 months before hyperkalaemia event | NR | 330 per 1000 person years; HR (95% CI): 7.8 (7.2-8.4)* | NR | NR |
|  |  |  | Patients with stage 1-5 CKD or on dialysis + Normokalaemia | 43,397 |  | 6 months before index date | NR | 39 per 1000 person years | NR | NR |
| Luo 2016 ([109](#_ENREF_109)) | Electronic health records | Overall | Patients with CKD with eGFR <30 mL/min/1.73 m2 + Hyperkalaemia (sK+ 5.0-5.4 mEq/L) | NR | Crude hospitalisation rate | NR | NR | Rate (95% CI): 3.0 (2.9-3.1) events per patient-year | NR | NR |
|  |  |  |  | NR | Adjusted hospitalisation rate | NR | NR | IRR (95% CI): 1.0 (0.8-1.2) | NR | NR |
|  |  |  | Patients with CKD with eGFR <30 mL/min/1.73 m2 + Hyperkalaemia (sK+ 5.5-5.9 mEq/L) | NR | Crude hospitalisation rate | NR | NR | Rate (95% CI): 1.0 (0.8-1.2) events per patient-year | NR | NR |
|  |  |  |  | NR | Adjusted hospitalisation rate | NR | NR | IRR (95% CI): 1.0 (0.8-1.2) | NR | NR |
|  |  |  | Patients with CKD with eGFR <30 mL/min/1.73 m2 + Hyperkalaemia (sK+ ≥6.0 mEq/L) | NR | Crude hospitalisation rate | NR | NR | Rate (95% CI): 9.0 (8.6-9.5) events per patient-year | NR | NR |
|  |  |  |  | NR | Adjusted hospitalisation rate | NR | NR | IRR (95% CI): 3.7 (2.7-5.0) | NR | NR |
|  |  |  | Patients with CKD with eGFR <30 mL/min/1.73 m2 + Normokalaemia (sK+ 3.5-3.9 mEq/L) | NR | Crude hospitalisation rate | NR | NR | Rate (95% CI): 4.1 (4.0-4.3) events per patient-year | NR | NR |
|  |  |  |  | NR | Adjusted hospitalisation rate | NR | NR | IRR (95% CI): 1.7 (1.3-2.1) | NR | NR |
|  |  |  | Patients with CKD with eGFR <30 mL/min/1.73 m2 + Normokalaemia (sK+ 4.0-4.4 mEq/L) | NR | Crude hospitalisation rate | NR | NR | Rate (95% CI): 3.0 (2.9-3.1) events per patient-year | NR | NR |
|  |  |  |  | NR | Adjusted hospitalisation rate | NR | NR | IRR (95% CI): 0.9 (0.8-1.1) | NR | NR |
|  |  |  | Patients with CKD with eGFR <30 mL/min/1.73 m2 + Normokalaemia (sK+ 4.5-4.9 mEq/L) | NR | Crude hospitalisation rate | NR | NR | Rate (95% CI): 3.0 (3.0-3.1) events per patient-year | NR | NR |
|  |  |  |  | NR | Adjusted hospitalisation rate | NR | NR | Reference | NR | NR |
|  |  |  | Patients with CKD with eGFR <30 mL/min/1.73 m2 + Hypokalaemia (sK+ <3.5 mEq/L) | NR | Crude hospitalisation rate | NR | NR | Rate (95% CI): 7.4 (7.0-7.9) events per patient-year | NR | NR |
|  |  |  |  | NR | Adjusted hospitalisation rate | NR | NR | IRR (95% CI): 1.9 (1.2-3.0) | NR | NR |
|  |  |  | Patients with CKD with eGFR 30-39 mL/min/1.73 m2 + Hyperkalaemia (sK+ 5.0-5.4 mEq/L) | NR | Crude hospitalisation rate | NR | NR | Rate (95% CI): 1.8 (1.8-1.9) events per patient-year | NR | NR |
|  |  |  |  | NR | Adjusted hospitalisation rate | NR | NR | IRR (95% CI): 1.0 (0.8-1.1) | NR | NR |
|  |  |  | Patients with CKD with eGFR 30-39 mL/min/1.73 m2 + Hyperkalaemia (sK+ 5.5-5.9 mEq/L) | NR | Crude hospitalisation rate | NR | NR | Rate (95% CI): 2.4 (2.3-2.5) events per patient-year | NR | NR |
|  |  |  |  | NR | Adjusted hospitalisation rate | NR | NR | IRR (95% CI): 1.1 (0.8-1.4) | NR | NR |
|  |  |  | Patients with CKD with eGFR 30-39 mL/min/1.73 m2 + Hyperkalaemia (sK+ ≥6.0 mEq/L) | NR | Crude hospitalisation rate | NR | NR | Rate (95% CI): 4.2 (3.9-4.6) events per patient-year | NR | NR |
|  |  |  |  | NR | Adjusted hospitalisation rate | NR | NR | IRR (95% CI): 1.8 (1.2-2.9) | NR | NR |
|  |  |  | Patients with CKD with eGFR 30-39 mL/min/1.73 m2 + Normokalaemia (sK+ 3.5-3.9 mEq/L) | NR | Crude hospitalisation rate | NR | NR | Rate (95% CI): 2.3 (2.2-2.4) events per patient-year | NR | NR |
|  |  |  |  | NR | Adjusted hospitalisation rate | NR | NR | IRR (95% CI): 1.4 (1.1-1.7) | NR | NR |
|  |  |  | Patients with CKD with eGFR 30-39 mL/min/1.73 m2 + Normokalaemia (sK+ 4.0-4.4 mEq/L) | NR | Crude hospitalisation rate | NR | NR | Rate (95% CI): 1.8 (1.7-1.8) events per patient-year | NR | NR |
|  |  |  |  | NR | Adjusted hospitalisation rate | NR | NR | IRR (95% CI): 1.0 (0.9-1.2) | NR | NR |
|  |  |  | Patients with CKD with eGFR 30-39 mL/min/1.73 m2 + Normokalaemia (sK+ 4.5-4.9 mEq/L) | NR | Crude hospitalisation rate | NR | NR | Rate (95% CI): 1.7 (1.7-1.7) events per patient-year | NR | NR |
|  |  |  |  | NR | Adjusted hospitalisation rate | NR | NR | Reference | NR | NR |
|  |  |  | Patients with CKD with eGFR 30-39 mL/min/1.73 m2 + Hypokalaemia (sK+ <3.5 mEq/L) | NR | Crude hospitalisation rate | NR | NR | Rate (95% CI): 4.4 (4.1-4.6) events per patient-year | NR | NR |
|  |  |  |  | NR | Adjusted hospitalisation rate | NR | NR | IRR (95% CI): 1.8 (1.2-2.6) | NR | NR |
|  |  |  | Patients with CKD with eGFR 40-49 mL/min/1.73 m2 + Hyperkalaemia (sK+ 5.0-5.4 mEq/L) | NR | Crude hospitalisation rate | NR | NR | Rate (95% CI): 1.4 (1.3-1.4) events per patient-year | NR | NR |
|  |  |  |  | NR | Adjusted hospitalisation rate | NR | NR | IRR (95% CI): 1.1 (0.9-1.3) | NR | NR |
|  |  |  | Patients with CKD with eGFR 40-49 mL/min/1.73 m2 + Hyperkalaemia (sK+ 5.5-5.9 mEq/L) | NR | Crude hospitalisation rate | NR | NR | Rate (95% CI): 1.9 (1.9-2.0) events per patient-year | NR | NR |
|  |  |  |  | NR | Adjusted hospitalisation rate | NR | NR | IRR (95% CI): 1.2 (0.9-1.6) | NR | NR |
|  |  |  | Patients with CKD with eGFR 40-49 mL/min/1.73 m2 + Hyperkalaemia (sK+ ≥6.0 mEq/L) | NR | Crude hospitalisation rate | NR | NR | Rate (95% CI): 2.5 (2.2-2.8) events per patient-year | NR | NR |
|  |  |  |  | NR | Adjusted hospitalisation rate | NR | NR | IRR (95% CI): 1.9 (1.1-3.3) | NR | NR |
|  |  |  | Patients with CKD with eGFR 40-49 mL/min/1.73 m2 + Normokalaemia (sK+ 3.5-3.9 mEq/L) | NR | Crude hospitalisation rate | NR | NR | Rate (95% CI): 1.6 (1.6-1.7) events per patient-year | NR | NR |
|  |  |  |  | NR | Adjusted hospitalisation rate | NR | NR | IRR (95% CI): 1.2 (1.0-1.5) | NR | NR |
|  |  |  | Patients with CKD with eGFR 40-49 mL/min/1.73 m2 + Normokalaemia (sK+ 4.0-4.4 mEq/L) | NR | Crude hospitalisation rate | NR | NR | Rate (95% CI): 1.3 (1.3-1.3) events per patient-year | NR | NR |
|  |  |  |  | NR | Adjusted hospitalisation rate | NR | NR | IRR (95% CI): 1.1 (1.0-1.2) | NR | NR |
|  |  |  | Patients with CKD with eGFR 40-49 mL/min/1.73 m2 + Normokalaemia (sK+ 4.5-4.9 mEq/L) | NR | Crude hospitalisation rate | NR | NR | Rate (95% CI): 1.2 (1.2-1.2) events per patient-year | NR | NR |
|  |  |  |  | NR | Adjusted hospitalisation rate | NR | NR | Reference | NR | NR |
|  |  |  | Patients with CKD with eGFR 40-49 mL/min/1.73 m2 + Hypokalaemia (sK+ <3.5 mEq/L) | NR | Crude hospitalisation rate | NR | NR | Rate (95% CI): 2.5 (2.4-2.7) events per patient-year | NR | NR |
|  |  |  |  | NR | Adjusted hospitalisation rate | NR | NR | IRR (95% CI): 2.2 (1.6-3.1) | NR | NR |
|  |  |  | Patients with CKD with eGFR 50-59 mL/min/1.73 m2 + Hyperkalaemia (sK+ 5.0-5.4 mEq/L) | NR | Crude hospitalisation rate | NR | NR | Rate (95% CI): 1.1 (1.1-1.1) events per patient-year | NR | NR |
|  |  |  |  | NR | Adjusted hospitalisation rate | NR | NR | IRR (95% CI): 1.0 (0.9-1.1) | NR | NR |
|  |  |  | Patients with CKD with eGFR 50-59 mL/min/1.73 m2 + Hyperkalaemia (sK+ 5.5-5.9 mEq/L) | NR | Crude hospitalisation rate | NR | NR | Rate (95% CI): 1.2 (1.2-1.3) events per patient-year | NR | NR |
|  |  |  |  | NR | Adjusted hospitalisation rate | NR | NR | IRR (95% CI): 0.8 (0.6-1.1) | NR | NR |
|  |  |  | Patients with CKD with eGFR 50-59 mL/min/1.73 m2 + Hyperkalaemia (sK+ ≥6.0 mEq/L) | NR | Crude hospitalisation rate | NR | NR | Rate (95% CI): 2.6 (2.3-2.9) events per patient-year | NR | NR |
|  |  |  |  | NR | Adjusted hospitalisation rate | NR | NR | IRR (95% CI): 1.2 (0.6-1.8) | NR | NR |
|  |  |  | Patients with CKD with eGFR 50-59 mL/min/1.73 m2 + Normokalaemia (sK+ 3.5-3.9 mEq/L) | NR | Crude hospitalisation rate | NR | NR | Rate (95% CI): 1.2 (1.2-1.3) events per patient-year | NR | NR |
|  |  |  |  | NR | Adjusted hospitalisation rate | NR | NR | IRR (95% CI): 1.1 (1.0-1.3) | NR | NR |
|  |  |  | Patients with CKD with eGFR 50-59 mL/min/1.73 m2 + Normokalaemia (sK+ 4.0-4.4 mEq/L) | NR | Crude hospitalisation rate | NR | NR | Rate (95% CI): 1.0 (1.0-1.0) events per patient-year | NR | NR |
|  |  |  |  | NR | Adjusted hospitalisation rate | NR | NR | IRR (95% CI): 1.0 (0.9-1.1) | NR | NR |
|  |  |  | Patients with CKD with eGFR 50-59 mL/min/1.73 m2 + Normokalaemia (sK+ 4.5-4.9 mEq/L) | NR | Crude hospitalisation rate | NR | NR | Rate (95% CI): 1.0 (1.0-1.1) events per patient-year | NR | NR |
|  |  |  |  | NR | Adjusted hospitalisation rate | NR | NR | Reference | NR | NR |
|  |  |  | Patients with CKD with eGFR 50-59 mL/min/1.73 m2 + Hypokalaemia (sK+ <3.5 mEq/L) | NR | Crude hospitalisation rate | NR | NR | Rate (95% CI): 2.1 (2.0-2.2) events per patient-year | NR | NR |
|  |  |  |  | NR | Adjusted hospitalisation rate | NR | NR | IRR (95% CI): 2.1 (1.6-2.7) | NR | NR |
| REVOLUTIONIZE III Bakris 2023 ([84](#_ENREF_84)) | Optum’s de-identified Market Clarity data | Overall | Patients with stage 3-4 CKD + Recurrent hyperkalaemia | 4549 | Inpatient admission or visits per patient | 12-month follow-up | Mean (SD): 0.8 (2.1) | NR | NR | NR |
|  |  |  | Patients with stage 3-4 CKD + Normokalaemia | 4549 |  |  | Mean (SD): 0.3 (1.2) | NR | NR | NR |

*6 months after hyperkalaemia event vs 6 months after index date.

**Abbreviations:** CI: confidence interval; CKD: chronic kidney disease; CPRD: Clinical Practice Research Datalink; DM: diabetes mellitus; DOPPS: Dialysis Outcomes and Practice Patterns Study; eGFR: estimated glomerular filtration rate; EMR: electronic medical record; ESRD: end stage renal disease; FFS: Free-For Service; HF: heart failure; HR: hazard ratio; HT: hypertension; ICU: intensive care unit; IQR: interquartile range; IRR: incidence rate ratio; JMDV: Japan Medical Data Vision; KDIGO: Kidney disease improving global outcomes; LCED: limited claims and electronic health record; m2: square metre; mEq/L: milliequivalents per litre; mL/min: millilitre per minutes; mmol/L: millimoles per litre; NR: not reported; RAASi: renin-angiotensin-aldosterone system inhibitors; SD: standard deviation; sK+: serum potassium; UEBMI: Urban Employee Basic Medical Insurance; UK: United Kingdom; USA: United States of America; vs: versus.

Table S21. RAASi sub-optimal dosing due to hyperkalaemia

| Study | Population type | Population description | Sample size | Treatment* | Proportion of patients with RAASi sub-optimal dosing (%)* |
| --- | --- | --- | --- | --- | --- |
| Kanda 2023 ([117](#_ENREF_117)) | Overall (Japan) | Patients with stage 3 or 4 CKD with or without HF | 1,427 | ACEi/ARB/ARNi/MRA | 4.4 |
|  | Subgroup (Japan) | Patients with stage 3 CKD with or without HF | 534 |  | 3.7 |
|  |  | Patients with stage 4 CKD with or without HF | 893 |  | 4.8 |
|  | Overall (USA) | Patients with stage 3 or 4 CKD with or without HF | 11,873 |  | 3.9 |
|  | Subgroup (USA) | Patients with stage 3 CKD with or without HF | 8,931 |  | 4 |
|  |  | Patients with stage 4 CKD with or without HF | 2,942 |  | 3.5 |
| FIDELIO-DKD  Agarwal 2022 ([128](#_ENREF_128)) | Overall | Patients with advanced CKD + DM | 2,802 | MRA (finerenone) | 0.3 |
|  |  |  | 2,796 | Placebo | 0.2 |
| Leon 2022 ([105](#_ENREF_105))  ([105](#_ENREF_105)) | Overall | Patients with stage 3 to 5 CKD survived 90-days after the hyperkalaemia episode (Manitoba cohort) | 7,200 | RAASi | 12.3 |
|  |  | Patients with stage 3 to 5 CKD survived 90-days after the hyperkalaemia episode (Ontario cohort) | 71,290 |  | 14.5 |
| Ren 2022 ([96](#_ENREF_96)) | Overall | Stage 3-5 CKD patients who survived 90 days after their first hyperkalaemia episode | 8,145 | RAASi | 64.2 |
| Riccio 2022 ([70](#_ENREF_70)) | Overall | Patients with stage 1 to 5 CKD | 556 | ACEi/ARB/ACEi + ARB combination | 0.2 |
|  | Subgroup | Patients with stage 1 CKD | 57 |  | 0 |
|  |  | Patients with stage 2 CKD | 106 |  | 0 |
|  |  | Patients with stage 3 CKD | 194 |  | 0 |
|  |  | Patients with stage 4 CKD | 114 |  | 0.9 |
|  |  | Patients with stage 5 CKD | 85 |  | 0 |
| Edwards 2021 ([1](#_ENREF_1)) | Overall | Patients with stage 2 or 3 non-diabetic CKD | 77 | MRA (spironolactone) | 4.6 |
|  |  |  | 77 | Diuretic (chlorthalidone) | 0 |
| Jun 2019 ([78](#_ENREF_78)) | Overall | Patients with stage 3-5 CKD | 20,184 | RAASi | 10 |
|  | Subgroup | Patients with stage 3a CKD | 9,444 |  | 10.7 |
|  |  | Patients with stage 3b CKD | 7,739 |  | 10.6 |
|  |  | Patients with stage 4 CKD | 2,632 |  | 8.7 |
|  |  | Patients with stage 5 CKD | 369 |  | 5.4 |
| Linde 2019 ([47](#_ENREF_47)) | Overall | Patients with CKD (sK+ 5 mmol/L) | 132,840 | RAASi <50% ESC recommended dose | 1.8** |
|  |  |  | 39,181 | RAASi ≥50% ESC recommended dose | 3.5** |
|  |  | Patients with CKD (sK+ 5.5 mmol/L) | 161,795 | RAASi <50% ESC recommended dose | 2** |
|  |  |  | 10,226 | RAASi ≥50% ESC recommended dose | 6** |
|  |  | Patients with CKD (sK+ 6 mmol/L) | 170,046 | RAASi <50% ESC recommended dose | 2.1** |
|  |  |  | 1,975 | RAASi ≥50% ESC recommended dose | 8.9** |
| Fröhlich 2016 ([69](#_ENREF_69)) | Overall | Patients with stable stage 3 or 4 CKD + HF | 722 | ACEi/ARB | 19 |
| Lee 2014 ([103](#_ENREF_103)) | Overall | Patients with stage 3 or 4 CKD with hyperkalaemia | 150 | Maintenance group (ACEi or ARB without discontinuation) | 27.3 |
|  |  |  | 108 | Withdrawal group (ACEi or ARB discontinuation for >3 months after hyperkalaemia) | NR |
| Pisoni 2012 ([48](#_ENREF_48)) | Overall | Patients with stage 3 CKD + Resistant HT | 36 | MRA (spironolactone/eplerenone) | NR |
|  |  |  | 32 | MRA (spironolactone) | 3.1 |
| Frimodt-Moller 2010 ([13](#_ENREF_13)) | Overall | Pre-dialysis stage 3-5 CKD patients | 47 | ACEi (enalapril) + ARB (candesartan) after 16 weeks of monotherapy with either enalapril or candesartan | 2.1 |
| Gulcicek 2023 ([82](#_ENREF_82)) | Overall | Patients with stages 1 to 5 CKD | 137 | ACEi/ARBs | 3.7 |
|  |  | Patients with stages 1-5 CKD and mild hyperkalaemia | 102 |  | 0.0 |
|  |  | Patients with stages 1-5 CKD and moderate/severe hyperkalaemia | 35 |  | 14.3 |
| Rastogi 2023 ([140](#_ENREF_140)) | Overall | Patients with stage 3-4 CKD | 7,506 | RAASi | 6.1 |
|  | Overall | Patients with stage 3-4 CKD | 1,179 | RAASi | 5.3 |
| Jimenez-Marrero 2024 ([56](#_ENREF_56)) | Overall | Patients with CKD | 3,868 | RAASi | 20.5 |
| OPTIMIZE I  Agiro 2023 ([142](#_ENREF_142)) | Overall | Patients with stages 3-5 CKD initiating SZC | 398 | RAASi | 6 |
|  | Overall | Patients with stages 3-5 CKD initiating SZC + DM | 311 | RAASi | 7 |

*To comprehensively understand the specific impact of RAASi treatment in patients with CKD, non-RAASi treatment comparisons were also included in the review;**Percentages of prescriptions down titrated.

**Abbreviations:** ACEi: angiotensin-converting enzyme inhibitors; ARBs: angiotensin-receptor blockers; ARNi: angiotensin receptor-neprilysin inhibitors; CKD: chronic kidney disease; DM: diabetes mellitus; eGFR: estimated glomerular filtration rate; ESC: European Society of Cardiology; HF: heart failure; HT: hypertension; mmol/L: millimoles per litre; MRAs: mineralocorticoid-receptor antagonists; NA: not applicable; NR: not reported; RAASi: renin-angiotensin-aldosterone system inhibitors; sK+: serum potassium; USA: United states of America.

Table S22. RAASi discontinuation due to hyperkalaemia

| Study | Population type | Population description | Sample size | Treatment | Discontinuation rate (%) | Duration of discontinuation |
| --- | --- | --- | --- | --- | --- | --- |
| Kanda 2023 ([117](#_ENREF_117)) | Overall (Japan) | Patients with stage 3 or 4 CKD with or without HF | 1,427 | ACEi/ARB/ARNi/MRA | 22.6 | NR |
|  | Subgroup (Japan) | Patients with stage 3 CKD with or without HF | 534 |  | 21.5 | NR |
|  |  | Patients with stage 4 CKD with or without HF | 893 |  | 23.3 | NR |
|  | Overall (USA) | Patients with stage 3 or 4 CKD with or without HF | 11,873 |  | 20.7 | NR |
|  | Subgroup (USA) | Patients with stage 3 CKD with or without HF | 8,931 |  | 20.5 | NR |
|  |  | Patients with stage 4 CKD with or without HF | 2,942 |  | 21.4 | NR |
| Li 2023b ([67](#_ENREF_67)) | Overall | Patients with stage 3-5 CKD + HFpEF | 22 | MRA (spironolactone - >40 mg) | NR | NR |
|  |  |  | 41 | MRA (spironolactone - ≤40 mg) | 2.4 | NR |
| Leon 2022 ([105](#_ENREF_105)) | Overall | Patients with stage 3-5 CKD survived 90-days after the hyperkalaemia episode (Manitoba cohort) | 7,200 | RAASi | 32.1 | NR |
|  |  | Patients with stage 3-5 CKD survived 90-days after the hyperkalaemia episode (Ontario cohort) | 71,290 |  | 11.4 | NR |
| Ren 2022 ([96](#_ENREF_96)) | Overall | Stage 3-5 CKD patients who survived 90 days after their first hyperkalaemia episode | 8,145 | RAASi | 17.8 | NR |
| Riccio 2022 ([70](#_ENREF_70)) | Overall | Patients with stage 1-5 CKD | 556 | ACEi/ARB/ACEi + ARB combination | 4.1 | NR |
|  | Subgroup | Patients with stage 1 CKD | 57 |  | 0.0 | NR |
|  |  | Patients with stage 2 CKD | 106 |  | 3.5 | NR |
|  |  | Patients with stage 3 CKD | 194 |  | 1.5 | NR |
|  |  | Patients with stage 4 CKD | 114 |  | 8.8 | NR |
|  |  | Patients with stage 5 CKD | 85 |  | 9.4 | NR |
| Santoro 2022 ([98](#_ENREF_98)) | Overall | Patients with stage ≤3 and >3 CKD and unspecified stage + Hyperkalaemia | 4,451 | RAASi drugs taken into consideration:  1. ACEi - plain  2. ACEi - combinations  3. ARB - plain  4. ARB combinations  5. Other agents acting on renin-angiotensin system | 21.8 | NR |
| MRA-ACE Trial  Tumlin 2022 ([21](#_ENREF_21)) | Overall | Patients with diabetic kidney disease | 18 | Maximum tolerated ACE/ARB | 0.0 | NR |
|  |  |  | 14 | Maximum tolerated ACE/ARB + MRA (spironolactone -25 mg) | 0.0 | NR |
| EX-DKD  Uchida 2022 ([97](#_ENREF_97)) | Overall | Patients with diabetic kidney disease + HT | 112 | MRA (esaxerenone) | 1.8 | NR |
|  | Subgroup | Patients with diabetic kidney disease + HT (UACR <30 mg/gCr) | 46 |  | 4.3 | NR |
|  |  | Patients with diabetic kidney disease + DM (UACR 30 to <300 mg/gCr) | 42 |  | 0 | NR |
|  |  | Patients with diabetic kidney disease + DM (UACR 300 to <1000 mg/gCr) | 24 |  | 0 | NR |
| BLOCK-CKD  Bakris 2021 ([12](#_ENREF_12)) | Overall | Patients with stage 3b or 4 CKD + Uncontrolled grade 1 and 2 systolic HT | 51 | MRA (KBP-5074 - 0.25 mg) | 0 | NR |
|  |  |  | 54 | MRA (KBP-5074 - 0.5 mg) | 3.7 | NR |
|  |  |  | 57 | Placebo | 3.5 | NR |
| Edwards 2021 ([1](#_ENREF_1)) | Overall | Patients with stage 2 or 3 non-diabetic CKD | 77 | MRA (spironolactone) | 0.8 | NR |
|  |  |  | 77 | Diuretic (chlorthalidone) | 0 | NR |
| FIGARO-DKD  Pitt 2021 ([9](#_ENREF_9)) | Overall | Patients with stage 2-4 CKD with moderately elevated albuminuria or stage 1 or 2 CKD with severely elevated albuminuria + DM | 3,683 | MRA (finerenone) | 1.2 | NR |
|  |  |  | 3,658 | Placebo | 0.4 | NR |
| Qu 2021 ([71](#_ENREF_71)) | Overall | Patients with stage 3-5 CKD | 44 | MRA (spironolactone - >40 mg per day) | 13.6 | NR |
|  |  |  | 156 | MRA (spironolactone - ≤40 mg per day) | 3.2 | NR |
| DRINK  Tang 2021 ([11](#_ENREF_11)) | Overall | Patients with stage 3 or 4 non-diabetic CKD | 37 | ARB (losartan) + DRI (aliskiren) | 0 | NR |
|  |  |  | 39 | ARB (losartan) | 0 | NR |
| FIDELIO-DKD  Bakris 2020 ([10](#_ENREF_10)) | Overall | Patients with advanced CKD + DM | 2,827 | MRA (finerenone) | 2.3 | NR |
|  |  |  | 2,831 | Placebo | 0.9 | NR |
| FIDELIO-DKD  Filippatos 2021 ([130](#_ENREF_130)) | Subgroup | Patients with advanced CKD and DM with history of CVD at baseline | 1,301 | MRA (finerenone) | 2.3 | NR |
|  |  |  | 1,299 | Placebo | 0.8 | NR |
|  |  | Patients with advanced CKD and DM without history of CVD at baseline | 1,526 | MRA (finerenone) | 2.2 | NR |
|  |  |  | 1,532 | Placebo | 1 | NR |
| FIDELIO-DKD  Agarwal 2022 ([128](#_ENREF_128)) | Subgroup | Patients with advanced CKD + DM | 2,802 | MRA (finerenone) | 2.3 | NR |
|  |  |  | 2,785 |  |  | NR |
|  |  |  | 2,796 | Placebo | 0.9 | NR |
|  |  |  | 2,775 |  |  | NR |
| FIDELIO-DKD  Rossing 2022a ([131](#_ENREF_131)) | Subgroup | Patients with advanced CKD and DM with concomitant SGLT2i therapy at baseline | 124 | MRA (finerenone) | Hyperkalaemia-related events leading to discontinuation: 0.8  Hyperkalaemia related serious AEs leading to discontinuation: 0.8 | NR |
|  |  |  | 135 | Placebo | Hyperkalaemia-related events leading to discontinuation: 0.7  Hyperkalaemia related serious AEs leading to discontinuation: 0 | NR |
|  |  | Patients with advanced CKD and DM without concomitant SGLT2i therapy at baseline | 2,703 | MRA (finerenone) | Hyperkalaemia-related events leading to discontinuation: 2.3  Hyperkalaemia serious AE leading to discontinuation: 0.1 | NR |
|  |  |  | 2,696 | Placebo | Hyperkalaemia-related events leading to discontinuation: 0.9  Hyperkalaemia related serious AEs leading to discontinuation: <0.1 | NR |
| FIDELIO-DKD  Zhang 2022b ([129](#_ENREF_129)) | Subgroup | Patients with advanced CKD + DM (Chinese sub-population) | 188 | MRA (finerenone) | 4.3 | NR |
|  |  |  | 184 | Placebo | 1.1 | NR |
| FIDELIO-DKD  Rossing 2022b ([132](#_ENREF_132)) | Subgroup | Patients with advanced CKD and DM with baseline HbA1C <7.5% | 1,382 | MRA (finerenone) | 2 | NR |
|  |  |  | 1,407 | Placebo | 0 | NR |
|  |  | Patients with advanced CKD and DM with baseline HbA1C >7.5% | 1,439 | MRA (finerenone) | 25 | NR |
|  |  |  | 1,421 | Placebo | 8 | NR |
|  |  | Patients with advanced CKD and DM without insulin use at baseline | 989 | MRA (finerenone) | 1.9 | NR |
|  |  |  | 1,041 | Placebo | 0.6 | NR |
|  |  | Patients with advanced CKD and DM with insulin use at baseline | 1,838 | MRA (finerenone) | 2.4 | NR |
|  |  |  | 1,790 | Placebo | 1.1 | NR |
| TOPCAT  Beldhuis 2019 ([7](#_ENREF_7)) | Overall | Patients with stage 1 and 2 CKD + HFpEF | 413 | MRA (spironolactone) | Incidence rate (95% CI): 4.3 (3.1-5.8) | p=0.003 |
|  |  |  | 410 | Placebo | Incidence rate (95% CI): 1.7 (1.0-2.9) |  |
|  |  | Patients with stage 3a CKD + HFpEF | 264 | MRA (spironolactone) | Incidence rate (95% CI): 10.7 (8.3-13.9) | p<0.001 |
|  |  |  | 259 | Placebo | Incidence rate (95% CI): 2.7 (1.7-4.3) |  |
|  |  | Patients with stage 3b CKD + HFpEF | 209 | MRA (spironolactone) | Incidence rate (95% CI): 21.0 (16.5-26.6) | p<0.001 |
|  |  |  | 202 | Placebo | Incidence rate (95% CI): 9.4 (6.8-13.1) |  |
| Spin-D  Charyatan 2019 ([5](#_ENREF_5)) | Overall | Patients with ESRD on haemodialysis | 27 | MRA (spironolactone - 12.5 mg per day) | 3.7 | NR |
|  |  |  | 26 | MRA (spironolactone - 25 mg per day) | 0 | NR |
|  |  |  | 25 | MRA (spironolactone - 50 mg per day) | 12 | NR |
|  |  |  | 51 | Placebo | 15.6 | NR |
| MiREnDa  Hammer 2019 ([4](#_ENREF_4)) | Overall | Patients with ESRD on haemodialysis | 50 | MRA (spironolactone) | 2 | NR |
|  |  |  | 47 | Placebo | NR | NR |
| Jun 2019 ([78](#_ENREF_78)) | Overall | Patients with stage 3-5 CKD | 20,184 | RAASi | 36.6 | NR |
| Linde 2019 ([47](#_ENREF_47)) | Overall | Patients with CKD (sK+ 5.0 mmol/L) | 132,840 | RAASi <50% ESC recommended dose | 2.6** | NR |
|  |  |  | 39,181 | RAASi ≥50% ESC recommended dose | 3.7** | NR |
|  |  | Patients with CKD (sK+ 5.5 mmol/L) | 161,795 | RAASi <50% ESC recommended dose | 2.7** | NR |
|  |  |  | 10,226 | RAASi ≥50% ESC recommended dose | 5.6** | NR |
|  |  | Patients with CKD (sK+ 6.0 mmol/L) | 170,046 | RAASi <50% ESC recommended dose | 2.8** | NR |
|  |  |  | 1,975 | RAASi ≥50% ESC recommended dose | 10** | NR |
| UK HARP-III  Haynes 2018 ([6](#_ENREF_6)) | Overall | Patients with stage A1-A3 CKD with eGFR 20 to 60 mL/min/1.73 m2 | 207 | ARNi (sacubitril/valsartan) | 2 | NR |
|  |  |  | 207 | ARB (irbesartan) | 0 | NR |
| Agrawal 2016 ([134](#_ENREF_134)) | Overall | Patients with CKD | 55 | ARB (telmisartan) | 1.8 | NR |
| PHASE  Walsh 2015 ([20](#_ENREF_20)) | Overall | Patients with ESRD on haemodialysis | 77 | MRA (eplerenone) | 3.9 | NR |
|  |  |  | 77 | Placebo | 2.6 | NR |
| EMPHASIS-HF  Eschalier 2013 ([25](#_ENREF_25)) | Overall | Patients with CKD with eGFR <60 ml/min/1.73 m2 | 422 | MRA (eplerenone) | 1.2 | NR |
|  |  |  | 461 | Placebo | NR | NR |
| ARTS-HF  Pitt 2013 ([26](#_ENREF_26)) | Overall | Patients with moderate (stage 3) CKD (eGFR 30 to 60 mL/min/1.73 m2) + HFpEF (Part B) | 66 | MRA (finerenone - 2.5 mg qd) | 3 | NR |
|  |  |  | 67 | MRA (finerenone - 5 mg qd) | 0 | NR |
|  |  |  | 67 | MRA (finerenone - 10 mg qd) | 0 | NR |
|  |  |  | 64 | MRA (finerenone - 5 mg bid) | 3.1 | NR |
|  |  |  | 63 | MRA (spironolactone - 25 or 50 mg qd) | 3.2 | NR |
|  |  |  | 65 | Placebo | 0 | NR |
| CRIB-II study  Edwards 2012 ([17](#_ENREF_17)) | Overall | Patients with stage 2 or 3 CKD | 56 | MRA (spironolactone) | 0.9 | NR |
|  |  |  | 56 | Placebo | NR | NR |
| Espinel 2012 ([15](#_ENREF_15)) | Overall | Patients with stage 3 CKD | 7 | ACEi (enalapril) | 42 | NR |
|  |  |  | 17 | ARB (olmesartan) | 0 | NR |
| Pisoni 2012 ([48](#_ENREF_48)) | Overall | Patients with stage 3 CKD + Resistant HT | 36 | MRA (spironolactone/eplerenone) | NR | NR |
|  |  |  | 32 | MRA (spironolactone) | 3.1 | NR |
| Taheri 2012 ([14](#_ENREF_14)) | Overall | Patients on chronic ambulatory peritoneal dialysis + Advanced HF | 9 | MRA (spironolactone) | 11.1 | NR |
|  |  |  | 9 | Placebo | NR | NR |
| Yildirim 2012 ([135](#_ENREF_135)) | Overall | Patients with stage 3-5 CKD | 279 | RAASi | 12.2 | NR |
| Boesby 2011 ([16](#_ENREF_16)) | Overall | Non-diabetic stage 1-4 CKD patients | 40 | MRA (eplerenone) vs control | 0 | NR |
| Frimodt-Moller 2010 ([13](#_ENREF_13)) | Overall | Pre-dialysis stage 3-5 CKD patients | 47 | ACEi (enalapril) + ARB (candesartan) after 16 weeks of monotherapy with either enalapril or candesartan | 0 | NR |
| Heshka 2010 ([104](#_ENREF_104)) | Overall | Patients with stage 3 or 4 CKD + Difficult-to-control HT | 34 | MRA (spironolactone) | 5.9 | NR |
| Rysava 2005 ([127](#_ENREF_127)) | Overall | Patients with CKD + HT | 92 | ARB (telmisartan) | 2.17 | NR |
| De Rosa 2002 ([76](#_ENREF_76)) | Subgroup | Patients with mild (CrCl: 30-60 mL/min/1.73 m2) chronic renal insufficiency + HT | 30 | ARB (irbesartan with or without concomitant antihypertensive therapy) | NR | NR |
|  |  | Patients with moderate to severe (CrCl: 10-29 mL/min/1.73 m2) chronic renal insufficiency + HT | 2 |  | 50 | NR |
| De Rosa 2001 ([77](#_ENREF_77)) | Subgroup | Patients with mild renal impairment (CrCl: 30-60 mL/min/1.73 m2) | 36 | ARB (irbesartan) | NR | NR |
|  |  | Patients with moderate to severe renal impairment (CrCl: 10-29 mL/min/1.73 m2) | 16 |  | 6.3 | NR |
| Ruilope 2000 ([3](#_ENREF_3)) | Overall | Patients with progressive CKD with or without proteinuria and HT | 22 | ARB (valsartan - 160 mg) | 2.4 | NR |
|  |  |  | 42 | ARB (valsartan - 80 mg) + ACEi (benazepril - 5 or 10 mg) | 2.3 | NR |
|  |  |  | 44 | ARB (valsartan - 160 mg) + ACEi (benazepril - 5 or 10 mg) | 1.7 | NR |
| Gulcicek 2023 ([82](#_ENREF_82)) | Overall | Patients with stages 1 to 5 CKD | 137 | ACEi/ARBs | 6.6 | NR |
|  |  | Patients with stages 1-5 CKD and mild hyperkalaemia | 102 |  | 0.0 | NR |
|  |  | Patients with stages 1-5 CKD and moderate/severe hyperkalaemia | 35 |  | 25.7 | NR |
| Rastogi 2023 ([140](#_ENREF_140)) | Overall | Patients with stage 3-4 CKD | 7,506 | RAASi | 32.8 | NR |
|  | Overall | Patients with stage 3-4 CKD | 1,179 | RAASi | 27.4 | NR |
| PROERCAN Garcia-Prieto 2024 ([31](#_ENREF_31)) | Overall | Patients with stages 3-4 CKD without proteinuria + HT | 40 | ACEi | 2.5 | NR |
|  |  |  | 48 | Non ACEi | 0.0 | NR |
| OPTIMIZE I Agiro 2023 ([142](#_ENREF_142)) | Overall | Patients with stages 3-5 CKD initiating SZC | 398 | RAASi | 15.0 | NR |
|  | Overall | Patients with stages 3-5 CKD initiating SZC + DM | 311 | RAASi | 15.0 | NR |
| Chinnadurai 2023 ([64](#_ENREF_64)) | Overall | Patients with non-dialysis dependent CKD | 321 | RAASi | 3.1 | NR |
| Tuttle 2024 ([32](#_ENREF_32)) | Overall | Patients with CKD | 147 | Pooled# BI 690517 3 mg | 4 | NR |
|  |  |  | 146 | Pooled# BI 690517 10 mg |  | NR |
|  |  |  | 146 | Pooled# BI 690517 20 mg |  | NR |
|  |  |  | 147 | Pooled# BI 690517 placebo | 0 | NR |
| Guney 2009 ([33](#_ENREF_33)) | Overall | Patients with stage 1-3 non-diabetic CKD | 15 | ACEIs and/or ARBs + spironolactone | **6.7** | NR |
|  |  |  | 15 | ACEIs and/or ARBs | NR | NR |
| Edwards 2009 ([34](#_ENREF_34)) | Overall | Patients with stage 2-3 non-diabetic CKD | 56 | Spironolactone | **0** | NR |
|  |  |  | 56 | Placebo | **0** | NR |

*To comprehensively understand the specific impact of RAASi treatment in patients with CKD, non-RAASi treatment comparisons were also included in the review ; **Percentage of prescriptions discontinuing RAAS therapy; #Pooled groups include participants who received BI 690517 either as monotherapy or in combination with empagliflozin.

**Abbreviations:** ACEi: angiotensin-converting enzyme inhibitors; AEs: adverse events; ARBs: angiotensin-receptor blockers; ARNi: angiotensin receptor-neprilysin inhibitors; bid: twice a day; CI: confidence interval; CKD: chronic kidney disease; CrCl: creatinine clearance; CVD: cardiovascular disease; DM: diabetes mellitus; DRI: direct renin inhibitors; eGFR: estimated glomerular filtration rate; ESC: European Society of Cardiology; ESRD: end stage renal disease; HBA1C: haemoglobin A1C; HF: heart failure; HFpEF: heart failure with preserved ejection fraction; HT: hypertension; m2: square metre; mg/gCr: milligram per gram creatinine; mg: milligram; MI: myocardial infarction; mL/min: millilitres per minute; mmol/L: millimoles per litre; MRA: mineralocorticoid-receptor antagonist; NA: not applicable; NR: not reported; qd; once daily; RAASi: renin-angiotensin-aldosterone system inhibitors; SGLT2i: sodium/glucose cotransporter-2 inhibitors; sK+: serum potassium; UACR: urinary albumin-to-creatinine ratio; USA: United states of America; vs: versus; SZC: sodium zirconium cyclosilicate.

Table S23. Impact of hyperkalaemia on the health-related quality of life

| Study | Population description | Treatment | Sample size | HRQoL instrument | Sub-scale | HRQoL score |
| --- | --- | --- | --- | --- | --- | --- |
| Grandy 2021 ([37](#_ENREF_37)) | Patients with stage 3-4 non-dialysis dependent CKD + Hyperkalaemia | NA | 216 | KDQOL | Burden of kidney disease | Mean: 54.9 |
|  |  |  |  |  | Effects of kidney disease | Mean: 69.6 |
|  |  |  |  |  | Physical health | Mean: 39.1 |
|  |  |  |  |  | Mental health | Mean: 45.3 |
|  |  |  |  |  | Symptoms/problems | Mean: 80.1 |
|  | Patients with stage 3-4 non-dialysis dependent CKD + Normokalaemia | NA | 933 | KDQOL | Burden of kidney disease | Mean: 60.7 |
|  |  |  |  |  | Effects of kidney disease | Mean: 76.1 |
|  |  |  |  |  | Physical health | Mean: 41.6 |
|  |  |  |  |  | Mental health | Mean: 46.8 |
|  |  |  |  |  | Symptoms/problems | Mean: 82 |
|  | Patients with stage 3-4 non-dialysis dependent CKD + Hyperkalaemia | NA | 216 | EQ-5D-3L VAS score | NA | Mean: 67.5 |
|  | Patients with stage 3-4 non-dialysis dependent CKD + Normokalaemia | NA | 933 | EQ-5D-3L VAS score | NA | Mean: 69.5 |

**Abbreviations:** CKD: chronic kidney disease; EQ-5D-3L VAS: European Quality of Life 5 Dimensions 3 Level Version and visual analogue scale; KDQOL: Kidney disease quality of life instrument; NA: not applicable.

Table S24. Healthcare costs (medical and pharmacy) associated with hyperkalaemia

| **Study** | **Country, Currency, Cost year** | **Population type** | **Population description** | **Sample size** | **Time point** | **Cost outcomes** |
| --- | --- | --- | --- | --- | --- | --- |
| Zhang 2022a ([36](#_ENREF_36)) | China, Chinese yuan (¥), NR | Overall | Patients with CKD having ≥1 inpatient or outpatient visit + Hyperkalaemia | 1,015 (Before PSM) | Baseline | All-cause direct medical cost per patient, mean (SD): ¥33,955 (¥44,475) |
|  |  |  |  | 1,003 (After PSM) |  | All-cause direct medical cost per patient, mean (SD): ¥33,015 (¥43,676) |
|  |  |  |  | 1,003 (After PSM) | Month-1 | **Total cost per patient, mean:** **23,630**  - Inpatient cost per patient, Mean: ¥22,204  - Outpatient cost per patient, Mean: ¥1,426 |
|  |  |  |  |  | Month-2 | **Total cost per patient, mean: 3,798**  - Inpatient cost per patient, mean: ¥2,510  - Outpatient cost per patient, mean: ¥1,288 |
|  |  |  |  |  | Month-3 | **Total cost per patient, mean:** ¥**3,357**  - Inpatient cost per patient, mean: ¥2,052  - Outpatient cost per patient, mean: ¥1,305 |
|  |  |  |  |  | Month-4 | **Total cost per patient, mean:** ¥**3,264**  - Inpatient cost per patient, mean: ¥1,875  - Outpatient cost per patient, mean: ¥1,389 |
|  |  |  |  |  | Month-5 | **Total cost per patient, mean:** ¥**2,967**  - Inpatient cost per patient, mean: ¥1,543  - Outpatient cost per patient, mean: ¥1,423 |
|  |  |  |  |  | Month-6 | **Total cost per patient, mean:** ¥**2,937**  - Inpatient cost per patient, mean: ¥1,534  - Outpatient cost per patient, mean: ¥1,402 |
|  |  |  |  |  | Month-7 | **Total cost per patient, mean:** ¥**3,041**  - Inpatient cost per patient, mean: ¥1,666  - Outpatient cost per patient, mean: ¥1,375 |
|  |  |  |  |  | Month-8 | **Total cost per patient, mean:** ¥**3,569**  - Inpatient cost per patient, mean: ¥2,064  - Outpatient cost per patient, mean: ¥1,506 |
|  |  |  |  |  | Month-9 | **Total cost per patient, mean:** ¥**3,218**  - Inpatient cost per patient, mean: ¥1,727  - Outpatient cost per patient, mean: ¥1,491 |
|  |  |  |  |  | Month-10 | **Total cost per patient, mean:** ¥**3,206**  - Inpatient cost per patient, mean: ¥1,654  - Outpatient cost per patient, mean: ¥1,552 |
|  |  |  |  |  | Month-11 | **Total cost per patient, mean:** ¥**3,070**  - Inpatient cost per patient, mean: ¥1,459  - Outpatient cost per patient, mean: ¥1,612 |
|  |  |  |  |  | Month-12 | **Total cost per patient, mean:** ¥**3,153**  - Inpatient cost per patient, mean: ¥1,567  - Outpatient cost per patient, mean: ¥1,586 |
|  |  |  |  |  | Overall, 12-Month follow-up | **1. Total all-cause direct medical costs, mean (95% CI):** ¥**59,120 (55,279-63,639)**  - Medication, mean (%): ¥26,696 (45%)  - Examination, mean (%): ¥8,955 (15%)  - Treatment, mean (%): ¥13,410 (23%)  - Surgery, mean (%): ¥592 (1%)  - Medical consumable, mean (%): ¥5,357 (9%)  - Other, mean (%): ¥4,111 (7%)  **2. Inpatient all-cause direct medical costs, mean (95% CI): 41,855 (38,297-45,830)**  - Medication, mean (%): ¥17,179 (41%)  - Examination, mean (%): ¥7,757 (18%)  - Treatment, mean (%): ¥7,431 (18%)  - Surgery, mean (%): ¥552 (1%)  - Medical consumable, mean (%): ¥4,926 (12%)  - Other, mean (%): ¥4,010 (10%)  **3. Outpatient all-cause direct medical costs, mean (95% CI): 17,355 (15,755-19,031)**  - Medication, mean (%): ¥9,607 (55%)  - Examination, mean (%): ¥1,198 (7%)  - Treatment, mean (%): ¥5,979 (34%)  - Surgery, mean (%): ¥40 (0%)  - Medical consumable, mean (%): ¥430 (3%)  - Other, mean (%): ¥102 (1%) |
|  |  |  | Patients with CKD having ≥1 inpatient or outpatient visit + Normokalaemia | 25,981 (Before PSM) | Baseline | All-cause direct medical cost per patient, mean (SD): ¥14,428 (¥22,326) |
|  |  |  |  | 1,003 (After PSM) |  | All-cause direct medical cost per patient, mean (SD): ¥31,353 (¥45,046) |
|  |  |  |  | 1,003 (After PSM) | Month-1 | **Total cost per patient, mean:** ¥**1,945**  - Inpatient cost per patient, mean: ¥1,032  - Outpatient cost per patient, mean: ¥913 |
|  |  |  |  |  | Month-2 | **Total cost per patient, mean:** ¥**1,650**  - Inpatient cost per patient, mean: ¥793  - Outpatient cost per patient, mean: ¥857 |
|  |  |  |  |  | Month-3 | **Total cost per patient, mean:** ¥**1,705**  - Inpatient cost per patient, mean: ¥860  - Outpatient cost per patient, mean: ¥844 |
|  |  |  |  |  | Month-4 | **Total cost per patient, mean:** ¥**1,469**  - Inpatient cost per patient, mean: ¥649  - Outpatient cost per patient, mean: ¥820 |
|  |  |  |  |  | Month-5 | **Total cost per patient, mean:** **1,550**  - Inpatient cost per patient, mean: ¥739  - Outpatient cost per patient, mean: ¥812 |
|  |  |  |  |  | Month-6 | **Total cost per patient, mean:** ¥**1,555**  - Inpatient cost per patient, mean: ¥774  - Outpatient cost per patient, mean: ¥781 |
|  |  |  |  |  | Month-7 | **Total cost per patient, mean:** ¥**1,683**  - Inpatient cost per patient, mean: ¥901  - Outpatient cost per patient, mean: ¥781 |
|  |  |  |  |  | Month-8 | **Total cost per patient, mean:** ¥**1,967**  - Inpatient cost per patient, mean: ¥1,158  - Outpatient cost per patient, mean: ¥809 |
|  |  |  |  |  | Month-9 | **Total cost per patient, mean:** ¥**2,077**  - Inpatient cost per patient, mean: ¥1,297  - Outpatient cost per patient, mean: ¥781 |
|  |  |  |  |  | Month-10 | **Total cost per patient, mean:** ¥**1,756**  - Inpatient cost per patient, mean: ¥943  - Outpatient cost per patient, mean: ¥813 |
|  |  |  |  |  | Month-11 | **Total cost per patient, mean:** ¥**1,798**  - Inpatient cost per patient, mean: ¥1,031  - Outpatient cost per patient, mean: ¥767 |
|  |  |  |  |  | Month-12 | **Total cost per patient, mean:** ¥**1,576**  - Inpatient cost per patient, mean: ¥803  - Outpatient cost per patient, mean: ¥773 |
|  |  |  |  |  | Overall, 12-Month follow-up | **1. Total all-cause direct medical costs, mean (95% CI):** ¥**20,732 (**¥**18,606–**¥**23,029)**  - Medication, mean (%): ¥12,378 (60%)  - Examination, mean (%): ¥2,621 (13%)  - Treatment, mean (%): ¥2,335 (11%)  - Surgery, mean (%): ¥243 (1%)  - Medical consumable, mean (%): ¥1,870 (9%)  - Other, mean (%): ¥1,284 (6%)  **2. Inpatient all-cause direct medical costs, mean (95% CI):** ¥**10,980 (**¥**9,034-**¥**13,061)**  - Medication, mean (%): ¥4,406 (40%)  - Examination, mean (%): ¥1,935 (18%)  - Treatment, mean (%): ¥1,551 (14%)  - Surgery, mean (%): ¥204 (2%)  - Medical consumable, mean (%): ¥1,660 (15%)  - Other, mean (%): ¥1,225 (11%)  **3. Outpatient all-cause direct medical costs, mean (95% CI):** ¥**9,752 (**¥**9,017-**¥**10,498)**  - Medication, mean (%): ¥7,972 (82%)  - Examination, mean (%): ¥686 (7%)  - Treatment, mean (%): ¥785 (8%)  - Surgery, mean (%): ¥40 (0%)  - Medical consumable, mean (%): ¥210 (2%)  - Other, mean (%): ¥59 (1%) |
| Sharma 2021 ([120](#_ENREF_120)) | USA, USD ($), NR | Subgroup | Patients with stage 1 CKD + Hyperkalaemia | NR | Post-index 1 year | Total healthcare costs PMPY: $11,960 |
|  |  |  | Patients with stage 1 CKD + Normokalaemia |  |  | Total healthcare costs PMPY: $6,417 |
|  |  |  | Patients with stage 2 CKD + Hyperkalaemia |  |  | Total healthcare costs PMPY: $10,687 |
|  |  |  | Patients with stage 2 CKD + Normokalaemia |  |  | Total healthcare costs PMPY: $7,341 |
|  |  |  | Patients with stage 3 CKD + Hyperkalaemia |  |  | Total healthcare costs PMPY: $17,986 |
|  |  |  | Patients with stage 3 CKD + Normokalaemia |  |  | Total healthcare costs PMPY: $11,500 |
|  |  |  | Patients with stage 4 CKD + Hyperkalaemia |  |  | Total healthcare costs PMPY: $23,711 |
|  |  |  | Patients with stage 4 CKD + Normokalaemia |  |  | Total healthcare costs PMPY: $17,996 |
|  |  |  | Patients with stage 5 CKD with dialysis + Hyperkalaemia |  |  | Total healthcare costs PMPY: $67,758 |
|  |  |  | Patients with stage 5 CKD with dialysis + Normokalaemia |  |  | Total healthcare costs PMPY: $58,969 |
|  |  |  | Patients with stage 5 CKD without dialysis + Hyperkalaemia |  |  | Total healthcare costs PMPY: $37,094 |
|  |  |  | Patients with stage 5 CKD without dialysis + Normokalaemia |  |  | Total healthcare costs PMPY: $26,945 |
| Kanda 2020 ([87](#_ENREF_87)) | Japan, USD ($), 2017 | Overall | Patients with stage 1-5 CKD + Hyperkalaemia | 16,133 | Within 12 months of follow-up | 1. Annual total costs per patient, mean (95% CI): $16,969 ($16,621-17,316)  2. Inpatient cost per visit, median: $6,594  3. Outpatient cost per visit, median: $246 |
|  |  |  |  |  | Month-1 | Cumulative total healthcare cost, mean: $4,836 |
|  |  |  |  |  | Month-2 | Cumulative total healthcare cost, mean: $6,557 |
|  |  |  |  |  | Month-3 | Cumulative total healthcare cost, mean: $7,951 |
|  |  |  |  |  | Month-4 | Cumulative total healthcare cost, mean: $9,016 |
|  |  |  |  |  | Month-5 | Cumulative total healthcare cost, mean: $9,754 |
|  |  |  |  |  | Month-6 | Cumulative total healthcare cost, mean: $10,574 |
|  |  |  |  |  | Month-7 | Cumulative total healthcare cost, mean: $11,516 |
|  |  |  |  |  | Month-8 | Cumulative total healthcare cost, mean: $12,336 |
|  |  |  |  |  | Month-9 | Cumulative total healthcare cost, mean: $13,115 |
|  |  |  |  |  | Month-10 | Cumulative total healthcare cost, mean: $14,098 |
|  |  |  |  |  | Month-11 | Cumulative total healthcare cost, mean: $14,836 |
|  |  |  |  |  | Month-12 | Cumulative total healthcare cost, mean: $15,656 |
|  |  |  |  | 14,193 | After 12 months of follow-up | 1. Annual total costs per patient, mean (95% CI): $11,980 ($11,702-$12,257)  2. Inpatient cost per visit, median: $6,210  3. Outpatient cost per visit, median: $259 |
|  |  |  | Patients with stage 1-5 CKD + Normokalaemia | 15,287 | Within 12 months of follow-up | 1. Annual total costs per patient, mean (95% CI): $4,439 ($4,298-$4,580)  2. Inpatient cost per visit, median: $4,397  3. Outpatient cost per visit, median: $180 |
|  |  |  |  |  | Month-1 | Cumulative total healthcare cost, mean: $984 |
|  |  |  |  |  | Month-2 | Cumulative total healthcare cost, mean: $1,312 |
|  |  |  |  |  | Month-3 | Cumulative total healthcare cost, mean: $1,680 |
|  |  |  |  |  | Month-4 | Cumulative total healthcare cost, mean: $2,049 |
|  |  |  |  |  | Month-5 | Cumulative total healthcare cost, mean: $2,459 |
|  |  |  |  |  | Month-6 | Cumulative total healthcare cost, mean: $2,664 |
|  |  |  |  |  | Month-7 | Cumulative total healthcare cost, mean: $2,951 |
|  |  |  |  |  | Month-8 | Cumulative total healthcare cost, mean: $3,238 |
|  |  |  |  |  | Month-9 | Cumulative total healthcare cost, mean: $3,443 |
|  |  |  |  |  | Month-10 | Cumulative total healthcare cost, mean: $3,771 |
|  |  |  |  |  | Month-11 | Cumulative total healthcare cost, mean: $4,016 |
|  |  |  |  |  | Month-12 | Cumulative total healthcare cost, mean: $4,303 |
|  |  |  |  | 15,159 | After 12 months of follow-up | 1. Annual total costs per patient, mean (95% CI): $3,228 (3,115-3,340)  2. Inpatient cost per visit, median: $4,156  3. Outpatient cost per visit, median: $194 |
|  |  | Subgroup | Patients with stage 4-5 CKD + Hyperkalaemia | 8,010 | Within 12 months of follow-up | **Cumulative total healthcare cost per patient, mean:** $**18,593**  - RRT cost, mean: $5,053  - Other cost: $13,540 |
|  |  |  | Patients with stage 4-5 CKD + Normokalaemia | 669 |  | **Cumulative total healthcare cost per patient, mean:** $**4,911**  **-** RRT cost, mean: $706  - Other cost: $4,205 |
| Betts 2020 ([121](#_ENREF_121)) | USA, USD ($), NR | Overall | CKD patients with hyperkalaemia-related hospitalisations | 1,809 | 1-year post-discharge period | Total all-cause healthcare costs, mean (SD): $66,961 ($135,515) |
|  |  |  | CKD patients with a hospitalisation without evidence of hyperkalaemia (normokalaemia) | 1,809 |  | Total all-cause healthcare costs, mean (SD): $38,166 ($74,066) |
|  |  | Subgroup | Stage 5 CKD patients with hyperkalaemia-related hospitalisations | 301 |  | Total all-cause healthcare costs, mean (SD): $94,336 ($135,750) |
|  |  |  | Stage 5 CKD patients with a hospitalisation without evidence of hyperkalaemia (normokalaemia) | 301 |  | Total all-cause healthcare costs, mean (SD): $65,227 ($122,738) |
|  |  |  | Stage 4 CKD patients with hyperkalaemia-related hospitalisations | 355 |  | Total all-cause healthcare costs, mean (SD): $55,446 ($76,012) |
|  |  |  | Stage 4 CKD patients with a hospitalisation without evidence of hyperkalaemia (normokalaemia) | 355 |  | Total all-cause healthcare costs, mean (SD): $34,929 ($55,386) |
|  |  |  | Stage 3 CKD patients with hyperkalaemia-related hospitalisations | 736 |  | Total all-cause healthcare costs, mean (SD): $65,064 ($148,342 |
|  |  |  | Stage 3 CKD patients with a hospitalisation without evidence of hyperkalaemia (normokalaemia) | 736 |  | Total all-cause healthcare costs, mean (SD): $34,137 ($66,168) |
|  |  |  | Unspecified CKD stage patients with hyperkalaemia-related hospitalisations | 417 |  | Total all-cause healthcare costs, mean (SD): $60,351 ($148,398) |
|  |  |  | Unspecified CKD stage patients with a hospitalisation without evidence of hyperkalaemia (normokalaemia) | 417 |  | Total all-cause healthcare costs, mean (SD): $28,500 ($44,392) |
| Mu 2020 ([122](#_ENREF_122)) | USA, USD ($), 2016 | Overall | Patients with CKD + Hyperkalaemia | 26,809 | Within 30 days | All-cause total medical cost, mean (SD): $8,831 ($14,595) |
|  |  |  | Patients CKD + Normokalaemia | 26,809 |  | All-cause total medical cost, mean (SD): $1,928 ($5,306) |
|  |  | Subgroup | Patients with stage 3 CKD + Hyperkalaemia | 9,733 |  | All-cause total medical cost, mean (SD): $7,330 ($13,932) |
|  |  |  | Patients with stage 3 CKD + Normokalaemia | 9,733 |  | All-cause total medical cost, mean (SD): $1,653 ($4,610) |
|  |  |  | Patients with stage 4 CKD + Hyperkalaemia | 4,535 |  | All-cause total medical cost, mean (SD): $7,786 ($13,007) |
|  |  |  | Patients with stage 4 CKD + Normokalaemia | 4535 |  | All-cause total medical cost, mean (SD): $1,747 ($4,516) |
|  |  |  | Patients with stage 5 CKD + Hyperkalaemia | 2,803 |  | All-cause total medical cost, mean (SD): $11,439 ($16,354) |
|  |  |  | Patients with stage 5 CKD + Normokalaemia | 2,803 |  | All-cause total medical cost, mean (SD): $2,331 ($6,090) |
|  |  |  | Patients with unspecified CKD stage + Hyperkalaemia | 9,738 |  | All-cause total medical cost, mean (SD): $10,067 ($15,183) |
|  |  |  | Patients with unspecified CKD stage + Normokalaemia | 9,738 |  | All-cause total medical cost, mean (SD): $2,173 ($5,992) |
|  |  | Overall | Patients with CKD + Hyperkalaemia | 26,809 | Within 1 year | All-cause total medical cost, mean (SD): $37,202 ($48,809) |
|  |  |  | Patients with CKD + Normokalaemia | 26,809 |  | All-cause total medical cost, mean (SD): $17,445 ($28,272) |
|  |  | Subgroup | Patients with stage 3 CKD + Hyperkalaemia | 9,733 |  | All-cause total medical cost, mean (SD): $30,498 ($42,910) |
|  |  |  | Patients with stage 3 CKD + Normokalaemia | 9,733 |  | All-cause total medical cost, mean (SD): $15,283 ($25,374) |
|  |  |  | Patients with stage 4 CKD + Hyperkalaemia | 4,535 |  | All-cause total medical cost, mean (SD): $37,621 ($48,462) |
|  |  |  | Patients with stage 4 CKD + Normokalaemia | 4,535 |  | All-cause total medical cost, mean (SD): $18,376 ($28,724) |
|  |  |  | Patients with stage 5 CKD + Hyperkalaemia | 2,803 |  | All-cause total medical cost, mean (SD): $56,175 (59,612) |
|  |  |  | Patients with stage 5 CKD + Normokalaemia | 2,803 |  | All-cause total medical cost, mean (SD): $22,625 ($34,591) |
|  |  |  | Patients with unspecified CKD stage + Hyperkalaemia | 9,738 |  | All-cause total medical cost, mean (SD): $38,245 ($49,527) |
|  |  |  | Patients with unspecified CKD stage + Normokalaemia | 9,738 |  | All-cause total medical cost, mean (SD): $17,682 ($28,561) |
| Neuenschwander 2023 ([116](#_ENREF_116)) | USA, USD ($), 2019 | Overall | CKD patients with ESRD + Hyperkalaemia | 134,241 | During index long-term care stay | **Direct healthcare costs per patient, mean (SD)**  **Total direct healthcare costs (Medical + Pharmacy):** $**27,354 (**$**33,285)**  Inpatient hospitalisations: $7,190 ($17,763)  Long-term care stay: $12,874 (16,344)  Outpatient: $2,687 ($8,444)  - Dialysis-related: $1,258 ($5,732)  Physician services & tests: $2,765 ($5,032)  Durable medical equipment: $158 ($1,344)  Hospice: $26 ($814)  Prescription drugs: $1,653 ($7,588)  - Oral binders: $16 ($229) |
|  |  |  | CKD patients with ESRD + Normokalaemia | 126,205 |  | **Direct healthcare costs per patient, mean (SD)**  **Total direct healthcare costs (Medical + Pharmacy):** $**19,059 (**$**24,376)**  Inpatient hospitalisations: $3,477 ($11,601)  Long-term care stay: $10,117 ($13,014)  Outpatient: $2,170 ($6,419)  - Dialysis-related: $1,052 ($4,553)  Physician services & tests: $1,815 ($4,032)  Durable medical equipment: $120 ($1,172)  Hospice: $22 ($733)  Prescription drugs: $1,338 ($6,790)  - Oral binders: $0 ($0) |
|  |  |  | CKD patients with ESRD + Hyperkalaemia | 59,445 | Up to 365 days post-discharge | **Direct healthcare costs per patient, mean (SD)**  **Total direct healthcare costs (Medical + Pharmacy):** $**70,896 (**$**69,037)**  Inpatient hospitalisations: $20,788 ($34,184)  Long-term care stay: $14,715 ($24,236)  Outpatient: $15,043 ($21,137)  - Dialysis-related: 1,258 (5,732)  Physician services & tests: $9,927 ($12,011)  Durable medical equipment: $908 ($3,345)  Hospice: $775 ($5,241)  Prescription drugs: $8,740 ($16,364)  - Oral binders: $80 ($648) |
|  |  |  | CKD patients with ESRD + Normokalaemia | 58,359 |  | **Direct healthcare costs per patient, mean (SD)**  **Total direct healthcare costs (Medical + Pharmacy):** **49,700 (50,836)**  Inpatient hospitalisations: $11,752 ($22,153)  Long-term care stay: $9,658 ($18,012)  Outpatient: $11,594 ($18,682)  - Dialysis-related: $1,052 ($4,553)  Physician services & tests: $7,260 ($10,081)  Durable medical equipment: $696 ($2,796)  Hospice: $835 ($5,536)  Prescription drugs: $7,905 ($16,179)  - Oral binders: 0 (0) |
| Betts 2018 ([125](#_ENREF_125)) | USA, USD ($), NR | Overall | Patients with CKD + Hyperkalaemia | 9620 | Within 30 days | All-cause total healthcare cost, mean: $7,324 |
|  |  |  | Patients with CKD + Normokalaemia | 9620 |  | All-cause total healthcare cost, mean: $2,541 |
|  |  |  | Patients with CKD + Hyperkalaemia | 9620 | Within 1 year | All-cause total healthcare cost, mean: $46,483 |
|  |  |  | Patients with CKD + Normokalaemia | 9620 |  | All-cause total healthcare cost, mean: $24,625 |
|  |  | Subgroup | Patients with stage 5 CKD + Hyperkalaemia | 1191 | Within 30 days | All-cause total healthcare cost, mean: $13,683 |
|  |  |  | Patients with stage 5 CKD + Normokalaemia | 1191 |  | All-cause total healthcare cost, mean: $3,998 |
|  |  |  | Patients with stage 4 CKD + Hyperkalaemia | 1903 |  | All-cause total healthcare cost, mean: $6,748 |
|  |  |  | Patients with stage 4 CKD + Normokalaemia | 1903 |  | All-cause total healthcare cost, mean: $3,019 |
|  |  |  | Patients with stage 3 CKD + Hyperkalaemia | 5795 |  | All-cause total healthcare cost, mean: $5,996 |
|  |  |  | Patients with stage 3 CKD + Normokalaemia | 5795 |  | All-cause total healthcare cost, mean: $2,116 |
|  |  |  | Patients with unspecified CKD stage + Hyperkalaemia | 731 |  | All-cause total healthcare cost, mean: $8,994 |
|  |  |  | Patients with unspecified CKD stage + Normokalaemia | 731 |  | All-cause total healthcare cost, mean: $2,289 |
|  |  |  | Patients with stage 5 CKD + Hyperkalaemia | 1191 | Within 1 year | All-cause total healthcare cost, mean: $99,616 |
|  |  |  | Patients with stage 5 CKD + Normokalaemia | 1191 |  | All-cause total healthcare cost, mean: $46,821 |
|  |  |  | Patients with stage 4 CKD + Hyperkalaemia | 1903 |  | All-cause total healthcare cost, mean: $45,911 |
|  |  |  | Patients with stage 4 CKD + Normokalaemia | 1903 |  | All-cause total healthcare cost, mean: $29,175 |
|  |  |  | Patients with stage 3 CKD + Hyperkalaemia | 5795 |  | All-cause total healthcare cost, mean: $35,592 |
|  |  |  | Patients with stage 3 CKD + Normokalaemia | 5795 |  | All-cause total healthcare cost, mean: $19,118 |
|  |  |  | Patients with unspecified CKD stage + Hyperkalaemia | 731 |  | All-cause total healthcare cost, mean: $47,736 |
|  |  |  | Patients with unspecified CKD stage + Normokalaemia | 731 |  | All-cause total healthcare cost, mean: $20,277 |
| Polson 2017 ([118](#_ENREF_118)) | USA, USD ($), NR | Overall | Patients with CKD + Hyperkalaemia | 321 | NR | 1. Overall medical cost, mean (SD): $28,891 ($55,076)  2. Fills per patient, mean (SD): $31.6 ($24.0)  3. Cost per fill, mean (SD): $221 ($935) |
|  |  |  | Patients with CKD + Normokalaemia | 3,102 |  | 1. Overall medical cost, mean (SD): $15,895 ($47,638)  2. Fills per patient, mean (SD): $32.2 ($25.2)  3. Cost per fill, mean (SD): 178 (867) |
|  |  |  | Patients with CKD + HF + Hyperkalaemia | 178 |  | 1. Overall medical cost, mean (SD): $50,029 ($72,305.1)  2. Fills per patient, mean (SD): $37.6 ($27.6)  3. Cost per fill, mean (SD): $157 ($692.20) |
|  |  |  | Patients with CKD + HF + Normokalaemia | 808 |  | 1. Overall medical cost, mean (SD): $33,614 ($66,157.2)  2. Fills per patient, mean (SD): $34.2 ($22.1)  3. Cost per fill, mean (SD): $149 ($516.4) |
| REVOLUTIONIZE III Bakris 2023 ([84](#_ENREF_84)) | USA, USD ($), 2022 | Overall | Patients with stage 3-4 CKD + Recurrent hyperkalaemia | 4549 | NR | **All-cause medical cost per patient:** Any setting, mean (SD): $34,163 ($63,556) Inpatient, mean (SD): $21,250 ($54,737) Emergency department, mean (SD), per patient: $2,191 ($8,491) Outpatient, mean (SD): $7,412 ($11,301) |
|  |  |  | Patients with stage 3-4 CKD + Normokalaemia | 4549 |  | **All-cause medical cost per patient:** Any setting, mean (SD): $15,175 ($33,634) Inpatient, mean (SD): $7,329 ($27,690) Emergency department, mean (SD): $961 ($3,431) Outpatient, mean (SD): $4,591 ($7,708) |

**Abbreviations:** CI: confidence interval; CKD: chronic kidney disease; ESRD: end-stage renal disease; NR: not reported; PMPY: per member per year; PSM: propensity score matching; RRT: renal replacement therapy; SD: standard; USA: United States of America; USD: United States dollar.

Table S25. Impact of sub-optimal RAASi dosing/ discontinuation

| Study | Population type | Population description | Sample size | Treatment arm | Outcomes |
| --- | --- | --- | --- | --- | --- |
| Polson 2017 ([118](#_ENREF_118)) | Overall | Patients with CKD | 850 | RAASi - optimal dose | 1. Inpatient visits, Mean (SD): 1.0 (0.0)  2. Length of stay, Mean (SD): 7.2 (6.8)  3. Overall cost, Mean (SD): $9,747 ($37,108)  4. Cost per fill, Mean (SD): $142.7 ($767.8) |
|  |  |  | 1,924 | RAASi - suboptimal dose | 1. Inpatient visits, Mean (SD): 1.2 (0.4)  2. Length of stay, Mean (SD): 6.2 (5.4)  3. Overall cost, Mean (SD): $10,259 ($26,453)  4. Cost per fill, Mean (SD): $142.1 ($639.6) |
|  |  | Patients with CKD + HF | 184 | RAASi - optimal dose | 1. Inpatient visits, Mean (SD): 1.0 (0.0)  2. Length of stay, Mean (SD): 15.0 (0)  3. Overall cost, Mean (SD): $22,672 ($37,180)  4. Cost per fill, Mean (SD): $117.1 ($701.6) |
|  |  |  | 507 | RAASi - suboptimal dose | 1. Inpatient visits, Mean (SD): 1.1 (0.3)  2. Length of stay, Mean (SD): 7.4 (3.9)  3. Overall cost, Mean (SD): $30,956 ($69,275)  4. Cost per fill, Mean (SD): $105.6 ($345.9) |
|  | Subgroup | Patients with CKD + Hyperkalaemia | 45 | RAASi - optimal dose | 1. Inpatient visits, Mean (SD): 1.0 (0.0)  2. Length of stay, Mean (SD): 10.3 (7.5)  3. Overall cost, Mean (SD): $58,097 ($139,595)  4. Cost per fill, Mean (SD): $115 ($314) |
|  |  |  | 158 | RAASi - suboptimal dose | 1. Inpatient visits, Mean (SD): 1.0 (0.0)  2. Length of stay, Mean (SD): 8.5 (9.2)  3. Overall cost, Mean (SD): $20,645 ($31,501)  4. Cost per fill, Mean (SD): $126 ($356) |
|  |  | Patients with CKD + Normokalaemia | 805 | RAASi - optimal dose | 1. Inpatient visits, Mean (SD): 1.0 (0.0)  2. Length of stay, Mean (SD): 2.5 (0.7)  3. Overall cost, Mean (SD): $7,044 ($15,795)  4. Cost per fill, Mean (SD): $144 ($784) |
|  |  |  | 1,766 | RAASi - suboptimal dose | 1. Inpatient visits, Mean (SD): 1.3 (0.5)  2. Length of stay, Mean (SD): 5.7 (4.9)  3. Overall cost, Mean (SD): $9,329 ($25,761)  4. Cost per fill, Mean (SD): $143 ($655) |
|  |  | Patients with CKD + HF + Hyperkalaemia | 26 | RAASi - optimal dose | 1. Inpatient visits: NR  2. Length of stay: NR  3. Overall cost, Mean (SD): $49,845 ($55,267)  4. Cost per fill, Mean (SD): $201 ($1,536.5) |
|  |  |  | 94 | RAASi - suboptimal dose | 1. Inpatient visits, Mean (SD): 1.0 (0.0)  2. Length of stay, Mean (SD): 11.0 (5.7)  3. Overall cost, Mean (SD): $58,682 ($93,346.3)  4. Cost per fill, Mean (SD): 1$11 ($274.00) |
|  |  | Patients with CKD + HF + Normokalaemia | 158 | RAASi - optimal dose | 1. Inpatient visits, Mean (SD): 1.0 (0)  2. Length of stay, Mean (SD): 15.0 (0)  3. Overall cost, Mean (SD): $18,200.5 ($31,344.5)  4. Cost per fill, Mean (SD): $108 ($541.80) |
|  |  |  | 413 | RAASi - suboptimal dose | 1. Inpatient visits, Mean (SD): 1.1 (0.3)  2. Length of stay, Mean (SD): 6.8 (3.5)  3. Overall cost, Mean (SD): $24,645 ($60,924.7)  4. Cost per fill, Mean (SD): $105 ($359.1) |
| Bhandari 2021 ([28](#_ENREF_28)) | Overall | Patients with stage 4 or 5 CKD | 206 | RAASi discontinuation group | **Outcomes at 3 years:**  1. eGFR (mL/min/1.732), Mean (SE): 12.6 (0.7); HR (95% CI): −0.7 (−2.5 to 1.0) vs RAASi continuation group  2. Proportion of patients with ESRD or RRT: 62%; HR (95% CI): 1.28 (0.99 to 1.65) vs RAASi continuation group  3. Proportion of patients with RRT (including patients with ESRD) or >50% decrease in eGFR: 68%; RR (95% CI): 1.07 (0.94 to 1.22) vs RAASi continuation group  4. Mortality rate: 10%; HR (95% CI): 0.85 (0.46 to 1.57) vs RAASi continuation group  5. Hospitalisation rate: 66%  6. SAE rate: 52%  7. No. of CV events: 108  8. Blood pressure (mm Hg), Mean (SE)  - Systolic: 140 (2)  - Diastolic: 76 (1)  9. Haemoglobin (g/dL), Mean (SE): 11.9 (0.1); HR (95% CI): 0 (−0.3 to 0.4) vs RAASi continuation group  10. Protein/creatinine ratio (mg/mmol), Mean (SE): 192 (31.0); HR (95% CI): -1.0 (-76.0 to 74.0 vs RAASi continuation group |
|  |  |  | 205 | RAASi continuation group | **Outcomes at 3 years:**  1. eGFR (mL/min/1.732), Mean (SE): 13.3 (0.6)  2. Proportion of patients with ESRD or RRT: 56%  3. Proportion of patients with RRT (including patients with ESRD) or >50% decrease in eGFR (Sample size: 202): 63%  4. Mortality rate: 11%  5. Hospitalisation rate: 72%  6. SAE rate: 49%  7. No. of CV events: 88  8. Blood pressure (mm Hg), Mean (SE)  - Systolic: 140 (2)  - Diastolic: 76 (1)  9. Haemoglobin (g/dL), Mean (SE): 11.9 (0.1); HR (95% CI): 0 (−0.3 to 0.4)  10. Protein/creatinine ratio (mg/mmol), Mean (SE): 193 (22.0) |
| Fu 2021 ([137](#_ENREF_137)) | Overall | Patients with advanced CKD (eGFR <30 mL/min per 1.73m2) | 10,254* | RAASi discontinuation group | **5-year absolute risk, % (95% CI); 5-yr risk difference, % (95% CI) vs RAASi continuation group:**  1. All-cause mortality: 54.5 (48.5 to 61.2); 13.6 (7.0 to 20.3)  2. MACE (composite of mortality, MI, and cerebrovascular events): 59.5 (53.8 to 66.1); 11.9 (5.7 to 18.6)  3. RRT (undergoing kidney transplantation or initiating maintenance dialysis): 27.9 (23.5 to 32.5); -8.3 (-12.8 to -3.6) |
|  |  |  | 10,254* | RAASi continuation group | **5-year absolute risk, % (95% CI):**  1. All-cause mortality: 40.9 (38.9 to 42.8)  2. MACE (composite of mortality, MI, and cerebrovascular events): 47.6 (45.9 to 49.4)  3. RRT (undergoing kidney transplantation or initiating maintenance dialysis): 36.1 (34.7 to 37.7) |
|  | Subgroup | Patients with advanced CKD (eGFR 20-30 mL/min per 1.73m2) | 7,277 | RAASi discontinuation group | **5-year absolute risk, % (95% CI); 5-yr risk difference, % (95% CI) vs RAASi continuation group:**  1. All-cause mortality: 50.9 (42.4 to 60.1); 12.3 (3.3 to 21.4)  2. MACE (composite of mortality, MI, and cerebrovascular events): 58.9 (49.2 to 67.8); 14.1 (4.6 to 23.5)  3. RRT (undergoing kidney transplantation or initiating maintenance dialysis): 12.8 (7.6 to 18.6); -2.5 (-17.8 to -6.6) |
|  |  |  |  | RAASi continuation group | **5-year absolute risk, % (95% CI):**  1. All-cause mortality: 38.6 (36.3 to 40.9)  2. MACE (composite of mortality, MI, and cerebrovascular events): 44.8 (42.7 to 46.9)  3. RRT (undergoing kidney transplantation or initiating maintenance dialysis): 25.3 (23.4 to 27.3) |
|  | Subgroup | Patients with advanced CKD (eGFR <20 mL/min per 1.73m2) | 6,907 | RAASi discontinuation group | **5-year absolute risk, % (95% CI); 5-yr risk difference, % (95% CI) vs RAASi continuation group:**  1. All-cause mortality: 61.0 (54.0 to 67.3); 17.1 (9.9 to 23.8)  2. MACE (composite of mortality, MI, and cerebrovascular events): 63.9 (57.0 to 70.0); 12.6 (5.8 to 19.3)  3. RRT (undergoing kidney transplantation or initiating maintenance dialysis): 43.4 (38.3 to 48.8); -9.6 (-15.0 to -3.8) |
|  |  |  |  | RAASi continuation group | **5-year absolute risk, % (95% CI):**  1. All-cause mortality: 43.9 (41.3 to 46.6)  2. MACE (composite of mortality, MI, and cerebrovascular events): 51.3 (48.9 to 53.9)  3. RRT (undergoing kidney transplantation or initiating maintenance dialysis): 52.9 (50.8 to 54.8) |
| Lee 2014 ([103](#_ENREF_103)) | Overall | Patients with stage 3 or 4 CKD who developed hyperkalaemia during treatment | 108 | Withdrawal group (ACEi or ARB discontinuation for >3 months after hyperkalaemia) | **1. Renal even-free survival rate, %:**  1-year follow-up: 89.0  2-year follow-up: 77.0  3-year follow-up: 67.0  4-year follow-up: 48.0  HR (95% CI): 1.35 (95% CI: 1.08–1.92, p=0.04) vs Maintenance group  **2. sCr (mg/dL):**  6-month follow-up: 2.9  12-month follow-up: 3.0  18-month follow-up: 3.4 |
|  |  |  | 150 | Maintenance group (ACEi or ARB continuation after hyperkalaemia)** | **1. Renal even-free survival rate, %:**  1-year follow-up: 93.0  2-year follow-up: 79.0  3-year follow-up: 74.0  4-year follow-up: 62.0  **2. sCr (mg/dL):**  6-month follow-up: 2.8  12-month follow-up: 2.6  18-month follow-up: 3.0; p<0.05 vs withdrawal group |
| Kanda 2023 ([117](#_ENREF_117)) | Overall (USA) | Patients with stage 3 or 4 CKD with or without HF | 11,873 | RAASi discontinuation following an episode of hyperkalaemia | **1. % risk of cardiorenal composite outcome ((HF emergency visit, HF hospitalisation, or progression to ESRD:**  - After 90 days of follow-up: 11.8  - After 180 days of follow-up: 17.1  **2. % risk of ESRD progression:**  - After 90 days of follow-up: 3.5  - After 180 days of follow-up: 5.8 |
|  |  |  |  | RAASi down-titration following an episode of hyperkalaemia (down-titration of at least one of the RAASi types by at least 25%) | **1. % risk of cardiorenal composite outcome ((HF emergency visit, HF hospitalisation, or progression to ESRD:**  - After 90 days of follow-up: 9.8  - After 180 days of follow-up: 17.1  **2. % risk of ESRD progression:**  - After 90 days of follow-up: 2.6  - After 180 days of follow-up: 5.4 |
|  |  |  |  | RAASi maintenance following an episode of hyperkalaemia (maintained the same dose for all RAASi types or up-titrated their dose) | **1. % risk of cardiorenal composite outcome ((HF emergency visit, HF hospitalisation, or progression to ESRD:**  - After 90 days of follow-up: 6.2  - After 180 days of follow-up: 10.0  **2. % risk of ESRD progression:**  - After 90 days of follow-up: 1.7  - After 180 days of follow-up: 3.2 |
|  | Overall (Japan) | Patients with stage 3 or 4 CKD with or without HF | 1,427 | RAASi discontinuation following an episode of hyperkalaemia | **1. % risk of cardiorenal composite outcome ((HF emergency visit, HF hospitalisation,**  **or progression to ESRD:**  - After 90 days of follow-up: 12.7  - After 180 days of follow-up: 17.1  **2. % risk of ESRD progression:**  - After 90 days of follow-up: 6.5  - After 180 days of follow-up: 8.1 |
|  |  |  |  | RAASi down-titration following an episode of hyperkalaemia (down-titration of at least one of the RAASi types by at least 25%) | **1. % risk of cardiorenal composite outcome ((HF emergency visit, HF hospitalisation,**  **or progression to ESRD:**  - After 90 days of follow-up: 9.5  - After 180 days of follow-up: 15.8  **2. % risk of ESRD progression:**  - After 90 days of follow-up: 3.2  - After 180 days of follow-up: 4.8 |
|  |  |  |  | RAASi maintenance following an episode of hyperkalaemia (maintained the same dose for all RAASi types or up-titrated their dose) | **% risk of cardiorenal composite outcome ((HF emergency visit, HF hospitalisation,**  **or progression to ESRD:**  - After 90 days of follow-up: 8.5  - After 180 days of follow-up: 12.6  **2. % risk of ESRD progression:**  **-** After 90 days of follow-up: 2.8  - After 180 days of follow-up: 5.2 |
| Linde 2019 ([47](#_ENREF_47)) | Overall | Patients with non-dialysis dependent stage ≥3 CKD | 27,935 | RAASi <50% ESC recommended dose (receiving <50% of the recommended dose over the majority [≥75% of quarters] of follow-up) | **Incidence rate per 1000 patient-years (95% CI):**  1. Nonfatal MACE (composite of arrhythmia, HF, MI, and stroke2): 130.38 (128.72–132.05); Adjusted IRR (95% CI): 1.61 (1.56–1.66) vs ≥50% RAASi ≥50% ESC recommended dose  2. All-cause mortality: 57.73 (56.63–58.85); Adjusted IRR (95% CI): 5.59 (95% CI, 5.28–5.92) vs RAASi ≥50% ESC recommended dose |
|  |  |  | 26,596 | RAASi ≥50% ESC recommended dose | **Incidence rate per 1000 patient-years (95% CI):**  1. Nonfatal MACE (composite of arrhythmia, HF, MI, and stroke): 72.95 (71.75–74.17)  2. All-cause mortality: 7.17 (6.80–7.56) |
| Santoro 2022 ([98](#_ENREF_98)) | Overall | Patients with CKD + hyperkalaemia | 881 (After PSM) | RAASi non-adherent group | **Incidence rate per 100 person-years**  1. CV events: 21.4; HR (95% CI): 1.45 (1.02 to 2.08), p<0.05 vs RAASi adherent group  2. All-cause mortality: 29.2; HR (95% CI): 2.26 (1.62–3.15), p<0.001 vs RAASi adherent group |
|  |  |  |  | RAASi adherent group*** | **Incidence rate per 100 person-years**  1. CV events: 13.4  2. All-cause mortality: 10.8 |
|  |  | Patients with CKD + no hyperkalaemia | 881 (After PSM) | RAASi non-adherent group | 1. CV events: HR (95% CI): 1.11 (0.93-1.32), p=0.252 compared to RAASi adherent group  2. Mortality: HR (95% CI): 1.29 (1.07-1.56), p=0.009 compared to RAASi adherent group |
| Yang 2023 ([94](#_ENREF_94)) | Overall | Patients with advanced CKD + DM | 1,766 | Discontinued-ACEi/ARBs group (stop within 6 months of eGFR  declined to <30 ml/min/1.73 m2) | **Crude incidence rate (95% CI) per 1000 person-years:**  1. MACE: 29.1 (25.5–33.1); HR (95% CI): 1.27 (1.08–1.49) compared to continued-ACEi/ARBs group  2. HF: 22.1 (18.9–25.6); HR (95% CI): 1.85 (1.53–2.25) compared to continued-ACEi/ARBs group  3. ESKD: 61.7 (56.1–67.7); HR (95% CI): 1.30 (1.17–1.43) compared to continued-ACEi/ARBs group  4. All-cause mortality: 84.4 (78.3–90.8); HR (95% CI): 0.93 (0.86–1.01) compared to continued-ACEi/ARBs group  5. CV specific mortality: HR (95% CI): 0.89 (0.73–1.08) compared to continued-ACEi/ARBs group  6. Cancer specific mortality: HR (95% CI): 0.0.97 (0.76–1.22) compared to continued-ACEi/ARBs group  7. Other cause-specific mortality: HR (95% CI): 0.94 (0.85–1.04) compared to continued-ACEi/ARBs group |
|  |  |  | 8,634 | Continued-ACEi/ARBs group (continuing within 6 months of eGFR  declined to <30 mL/min/1.73 m2) | **Crude incidence rate (95% CI) per 1000 person-years**  1. MACE: 34.9 (33.0–37.0)  2. HF: 34.8 (32.8–36.8)  3. ESRD: 82.2 (78.9–85.5)  4. All-cause mortality: 83.4 (80.5–86.5) |
| Ahmed 2010 ([138](#_ENREF_138)) | Overall | Patients with stage 4 or 5 CKD preparing for RRT | 52 | 12 months before ACEi/ARB were stopped | **1. Renal functional characteristics, Mean (SEM)**  - eGFR (ml/min/1.73 m2): 22.9 (1.4)  - Total change in eGFR slope: -0.39 (0.07)  - eGFR rate of change for patients who improved >25% (ml/min/month): -0.49 (0.1)  - eGFR slope for patients who improved >25%: -0.5 (0.1)  - eGFR rate of decline for patients who deteriorated >25% (mL/min/month):-0.06 (0.1)  - eGFR slope for patients who deteriorated >25%: -0.05 (0.1)  - eGFR rate of decline for patients who remained unchanged (mL/min/month): -0.14 (0.1)  - eGFR slope for patients who remained unchanged: -0.13 (0.06)  **2. Urine protein/creatinine ratio (mg/mmol), Mean (SEM):** 79.5 (24.1) |
|  |  |  |  | When ACEi/ARB were stopped | **1. eGFR (ml/min/1.73 m2), Mean (SEM): 16.38 (1.0)**  **2. Blood pressure (mmHg), Mean (SEM)**  - Systolic blood pressure: 134 (3.0)  - Diastolic blood pressure: 69 (1.7)  -Arterial blood pressure: 90 (1.8)  **3. Urine protein/creatinine ratio (mg/mmol), Mean (SEM): 77 (20)** |
|  |  |  |  | 12 months after ACEi/ARB were stopped | **1. Renal functional characteristics, Mean (SEM)**  - eGFR (ml/min/1.73 m2): 26.6 (2.2)  - Total change in eGFR slope: 0.48 (0.1)  - eGFR rate of change for patients who improved >25% (ml/min/month): 0.95 (0.1)  - eGFR slope for patients who improved >25%: 0.78 (0.1)  - eGFR rate of decline for patients who deteriorated >25% (mL/min/month): -0.2 (0.1)  - eGFR slope for patients who deteriorated >25%: -0.6 (0.1)  - eGFR rate of decline for patients who remained unchanged (mL/min/month): -0.11 (0.1)  - eGFR slope for patients who remained unchanged: -0.12 (0.06)  **2. Blood pressure (mmHg), Mean (SEM):**  - Systolic blood pressure: 139 (2.2)  - Diastolic blood pressure: 72 (1.4)  - Arterial pressure: 94 (1.3)  **3. Urine protein/creatinine ratio (mg/mmol), Mean (SEM): 121.6 (33.6)** |
|  | Subgroup | Patients with stage 4 or 5 CKD preparing for RRT + DM | NR | 12 months before ACEi/ARB were stopped | 1. eGFR (ml/min/1.73 m2), Mean (SEM): 22.7 (1.7)  2. Urine protein/creatinine ratio, Mean (SEM): 97.5 ± 36.2 |
|  |  |  |  | When ACEi/ARB were stopped | 1. eGFR (ml/min/1.73 m2), Mean (SEM): 15.5 (1.3)  2. Urine protein/creatinine ratio, Mean (SEM): 110.4 (38.3) |
|  |  |  |  | 12 months after ACEi/ARB were stopped | 1. eGFR (ml/min/1.73 m2), Mean (SEM): 23.9 (3.3)  2. Urine protein/creatinine ratio, Mean (SEM): 135.7 (48.2) |
|  | Subgroup | Patients with stage 4 or 5 CKD without DM preparing for RRT | NR | 12 months before ACEi/ARB were stopped | 1. eGFR (ml/min/1.73 m2), Mean (SEM): 23.2 (2.2)  2. Urine protein/creatinine ratio, Mean (SEM): 62.2 (32.5) |
|  |  |  |  | When ACEi/ARB were stopped | 1. eGFR (ml/min/1.73 m2), Mean (SEM): 17.5 (1.7)  2. Urine protein/creatinine ratio, Mean (SEM): 51.3 (16) |
|  |  |  |  | 12 months after ACEi/ARB were stopped | 1. eGFR (ml/min/1.73 m2), Mean (SEM): 28.6 (2.9)  2. Urine protein/creatinine ratio, Mean (SEM): 108 (47.6) |
| Rastogi 2023 ([140](#_ENREF_140)) | USA subgroup | Patients with stage 3-4 CKD | 2,460 | Maintained RAASi group | Reference |
|  |  |  | 4,586 | Discontinued RAASi group | Risk of progression to ESRD, adjusted HR (95% CI): 1.74 (1.37-2.21); p<0.001 |
|  |  |  | 460 | Down-titrated RAASi group | Risk of progression to ESRD, adjusted HR (95% CI): 1.60 (1.02-2.49); p=0.039 |
|  | Japan Subgroup |  | 793 | Maintained RAASi group | Reference |
|  |  |  | 323 | Discontinued RAASi group | Risk of progression to ESRD, adjusted HR (95% CI): 1.70 (1.01-2.86); p=0.045 |
|  |  |  | 63 | Down-titrated RAASi group | NR |
| An 2023 ([85](#_ENREF_85)) | Overall | Patients with CKD | 776 | RAASi discontinuation group | **1. Composite of kidney outcome or all-cause mortality:** - n (%): 292 (37.6) - Event per 1,000 person-years (95% CI): 199.9 (178.2 to 224.2); multivariable adjusted HR (95% CI): 1.21 (1.06 to 1.37) vs RAASi continuation group  **2. All-cause mortality:** - n (%): 207 (26.7) - Event per 1,000 person-years (95% CI): 131.5 (114.8 to 150.7); multivariable adjusted HR (95% CI): 1.34 (1.14 to 1.56) vs RAASi continuation group  3. Worsening of kidney function (40% reduction in eGFR or ESRD): - n (%): 116 (14.9) - Event per 1,000 person-years (95% CI): 79.4 (66.2 to 95.3); multivariable adjusted HR (95% CI): 1.01 (0.83, 1.23) vs RAASi continuation group  **4. 40% reduction in eGFR, n (%):** 112 (16.2); p=0.61 vs RAASi continuation group  **5. Cardiovascular outcome:** - n (%): 100 (12.9) - Event per 1,000 person-years (95% CI): 67.7 (55.7 to 82.4); multivariable adjusted HR (95% CI): 1.07 (0.86 to 1.34) vs RAASi continuation group  **6. ESRD (dialysis/kidney transplant), n (%):** 29 (3.7); p=0.11 vs RAASi continuation group  **7. New-onset proteinuria, n (%):** 9 (1.2); p=0.10 **8. 50% increase in serum creatinine, n (%):** 110 (15.6); p=0.71 vs RAASi continuation group |
|  |  |  | 4952 | RAASi continuation group | **1. Composite of kidney outcome or all-cause mortality:** - n (%): 1,411 (28.5) - Event per 1,000 person-years (95% CI): 138.2 (131.2, 145.6) **2. All-cause mortality:** - n (%): 847 (17.1) - Event per 1,000 person-years (95% CI): 76.9 (71.9, 82.2) **3. Worsening of kidney function (40% reduction in eGFR or ESRD):** - n (%): 733 (14.8) - Event per 1,000 person-years (95% CI): 71.8 (66.8, 77.2) **4. 40% reduction in eGFR, n (%):** 714 (15.4) **5. Cardiovascular outcome:** - n (%): 562 (11.3) - Event per 1,000 person-years (95% CI): 53.5 (49.3, 58.2) **6. ESRD (dialysis/kidney transplant), n (%):** 134 (2.7) **7. New-onset proteinuria, n (%):** 31 (0.6) **8. 50% increase in serum creatinine, n (%):** 705 (15.1) |
| OPTIMIZE I Agiro 2023 ([142](#_ENREF_142)) | Subgroup | Patients with stages 3-5 CKD initiating SZC | 314 | RAASi optimisation (same dose or with an up-titration) | **All-Cause healthcare resource use:** - Any inpatient stays, n (%): 69 (22.0) - Number of inpatient stays, mean (SD): 0.36 (0.82) - Any ED visits, n (%): 83 (26.4) - Number of ED visits, mean (SD): 0.40 (0.89) - Any outpatient visits, n (%): 310 (98.7)  - Number of outpatient visits, mean (SD):13.89 (13.62) |
|  |  |  | 84 | Non-optimised RAASi (discontinued or with a down-titration) | **All-Cause healthcare resource use:** - Any inpatient stays, n (%): 25 (29.8); p=0.18 vs RAASi optimisation group - Number of inpatient stays, mean (SD): 0.56 (1.20); p=0.13 vs RAASi optimisation group - Any ED visits, n (%): 32 (38.1); p=0.05 vs RAASi optimisation group - Number of ED visits, mean (SD): 0.61 (0.98); p<0.05 vs RAASi optimisation group - Any outpatient visits, n (%): 82 (97.6); p=0.61 vs RAASi optimisation group  - Number of outpatient visits, mean (SD): 17.04 (19.23); p=0.13 vs RAASi optimisation group |
|  | Subgroup | Patients with stages 3-5 CKD initiating SZC + DM | 243 | RAASi optimisation (same dose or with an up-titration) | 1. Any inpatient stays, n (%): 61 (25.1) 2. Number of inpatient stays, mean (SD): 0.41 (0.87) |
|  |  |  | 68 | Non-optimised RAASi (discontinued or with a down-titration) | 1. Any inpatient stays, n (%): 20 (29.4); p=0.58 vs RAASi optimisation group 2. Number of inpatient stays, mean (SD): 0.56 (1.19); p=0.42 vs RAASi optimisation group |
| Svensson 2023 ([59](#_ENREF_59)) | Overall | Patients with CKD | NR | Reduced RAASi | **CKD inpatient days per-person year:** - 6-month pre-hyperkalaemia: 3.4 - 6-month post-hyperkalaemia: 8.5 (increase by 149%) |
|  |  |  | NR | Maintained RAASi | **CKD inpatient days per-person year:** - 6-month pre-hyperkalaemia: 3.3 - 6-month post-hyperkalaemia: 6.6 (increase by 93%) |

*Of 10,254 prevalent RAASi users, 15,53 stopped therapy within 6 months. Of these, 887 patients restarted RAASi during follow-up. After cloning, 10,254 individuals were assigned to each treatment strategy.

**Most of the patients in the maintenance group were maintained on the previous dose of RAASi, and the dose was reduced in 41/150 patients.

***Adherence to RAASi therapy was evaluated in the 12-months after the index date by calculating the proportion of days covered, i.e., the ratio between the number of days of medication and days of observation (365 days), multiplied by 100. Patients were considered as being adherent to therapy if they had a PDC > 80%.

**Abbreviations:** ACEi: angiotensin converting enzyme inhibitors; ARBs: angiotensin-receptor blockers; CI: confidence interval; CKD: chronic kidney disease; CV: cardiovascular; DM: diabetes mellitus; ED: emergency department; eGFR; estimated glomerular filtration rate; ESC: European Society of Cardiology; ESRD; end stage renal disease; g/dL; grams per decilitre; HF: heart failure; HF: heart failure; HR: hazard ratio; HRQoL: health related quality of life; IRR, incident rate ratio; m2: square metre; MACE: major adverse cardiovascular events; mg/dL; milligrams per decilitre; mg/mmol: milligrams per millimole; MI: myocardial infarction; mL/min: millilitre per minute; NR: not reported; PSM: propensity score matching; RAASi: renin-angiotensin-aldosterone system inhibitors; RR: risk ratio; RRT: renal replacement therapy; RRT: renal replacement therapy; SAE: serious adverse event; sCr: serum creatinine; SD: standard deviation; SE: standard error; SEM: standard error of mean; vs: versus; SZC: sodium zirconium cyclosilicate.

**References**

1. Edwards NC, Price AM, Mehta S, et al. Effects of spironolactone and chlorthalidone on cardiovascular structure and function in chronic kidney disease: A randomized, open-label trial. Clinical Journal of the American Society of Nephrology 2021; 16:1491-1501.

2. Straburzynska-Migaj E, Senni M, Wachter R, et al. Initiation of sacubitril/valsartan in patients with renal impairment early after acute decompensated heart failure in the TRANSITION study. European Journal of Heart Failure 2021; 23:46.

3. Ruilope LM, Aldigier JC, Ponticelli C, Oddou-Stock P, Botteri F, Mann JF. Safety of the combination of valsartan and benazepril in patients with chronic renal disease. Journal of Hypertension 2000; 18:89-95.

4. Hammer F, Malzahn U, Donhauser J, et al. A randomized controlled trial of the effect of spironolactone on left ventricular mass in hemodialysis patients. Kidney international 2019; 95:983-991.

5. Charytan DM, Himmelfarb J, Ikizler TA, et al. Safety and cardiovascular efficacy of spironolactone in dialysis-dependent ESRD (SPin-D): a randomized, placebo-controlled, multiple dosage trial. Kidney international 2019; 95:973-982.

6. Haynes R, Judge PK, Staplin N, et al. Effects of sacubitril/valsartan versus irbesartan in patients with chronic kidney disease: A randomized double-blind trial. Circulation 2018; 138:1505-1514.

7. Beldhuis IE, Myhre PL, Claggett B, et al. Efficacy and Safety of Spironolactone in Patients With HFpEF and Chronic Kidney Disease. JACC: Heart Failure 2019; 7:25-32.

8. Provenzano M, Puchades MJ, Garofalo C, et al. Albuminuria-Lowering Effect of Dapagliflozin, Eplerenone, and their Combination in Patients with Chronic Kidney Disease: A Randomized Cross-over Clinical Trial. Journal of the American Society of Nephrology 2022; 33:1569-1580.

9. Pitt B, Filippatos G, Agarwal R, et al. Cardiovascular Events with Finerenone in Kidney Disease and Type 2 Diabetes. The New England journal of medicine 2021; 385:2252-2263.

10. Bakris GL, Agarwal R, Anker SD, et al. Effect of finerenone on chronic kidney disease outcomes in type 2 diabetes. New England Journal of Medicine 2020; 383:2219-2229.

11. Tang SCW, Chan KW, Ip DKM, et al. Direct Renin Inhibition in Non-diabetic chronic Kidney disease (DRINK): A prospective randomized trial. Nephrology Dialysis Transplantation 2021; 36:1648-1656.

12. Bakris G, Pergola PE, Delgado B, et al. Effect of KBP-5074 on Blood Pressure in Advanced Chronic Kidney Disease: Results of the BLOCK-CKD Study. Hypertension 2021; 78:74-81.

13. Frimodt-Moller M, Hoj Nielsen A, Strandgaard S, Kamper AL. Feasibility of combined treatment with enalapril and candesartan in advanced chronic kidney disease. Nephrology Dialysis Transplantation 2010; 25:842-847.

14. Taheri S, Mortazavi M, Pourmoghadas A, Seyrafian S, Alipour Z, Karimi S. A prospective double-blind randomized placebo-controlled clinical trial to evaluate the safety and efficacy of spironolactone in patients with advanced congestive heart failure on continuous ambulatory peritoneal dialysis. Saudi journal of kidney diseases and transplantation : an official publication of the Saudi Center for Organ Transplantation, Saudi Arabia 2012; 23:507-512.

15. Espinel E, Joven J, Gil I, et al. Risk of hyperkalemia in patients with moderate chronic kidney disease initiating angiotensin converting enzyme inhibitors or angiotensin receptor blockers: a randomized study. BMC research notes 2013; 6:306.

16. Boesby L, Elung-Jensen T, Klausen TW, Strandgaard S, Kamper AL. Moderate antiproteinuric effect of add-on aldosterone blockade with eplerenone in non-diabetic chronic kidney disease. a randomized cross-over study. PLoS ONE 2011; 6:e26904.

17. Edwards NC, Steeds RP, Chue CD, Stewart PM, Ferro CJ, Townend JN. The safety and tolerability of spironolactone in patients with mild to moderate chronic kidney disease. British Journal of Clinical Pharmacology 2012; 73:447-454.

18. Ando K, Ohtsu H, Uchida S, Kaname S, Arakawa Y, Fujita T. Anti-albuminuric effect of the aldosterone blocker eplerenone in non-diabetic hypertensive patients with albuminuria: A double-blind, randomised, placebo-controlled trial. The Lancet Diabetes and Endocrinology 2014; 2:944-953.

19. Vukusich A, Kunstmann S, Varela C, et al. A randomized, double-blind, placebo-controlled trial of spironolactone on carotid intima-media thickness in nondiabetic hemodialysis patients. Clin J Am Soc Nephrol 2010; 5:1380-7.

20. Walsh M, Manns B, Garg AX, et al. The Safety of Eplerenone in Hemodialysis Patients: A Noninferiority Randomized Controlled Trial. Clin J Am Soc Nephrol 2015; 10:1602-8.

21. Tumlin JA, Kopyt NP, Wilson DJ. Safety and Efficacy of Maximally Tolerated RAS Therapy Alone or in Combination With Spironolactone in Diabetic Kidney Disease: Effect on Proteinuria and eGFR in the MRA-ACE Trial. Journal of the American Society of Nephrology 2022; 33:677.

22. Weinberg JM, Appel LJ, Bakris G, et al. Risk of hyperkalemia in nondiabetic patients with chronic kidney disease receiving antihypertensive therapy. Archives of internal medicine 2009; 169:1587-94.

23. Hayashi K, Kumagai H, Saruta T. Effect of efonidipine and ACE inhibitors on proteinuria in human hypertension with renal impairment. American journal of hypertension 2003; 16:116-22.

24. Abolghasmi R, Taziki O. Efficacy of low dose spironolactone in chronic kidney disease with resistant hypertension. Saudi journal of kidney diseases and transplantation : an official publication of the Saudi Center for Organ Transplantation, Saudi Arabia 2011; 22:75-78.

25. Eschalier R, McMurray JJV, Swedberg K, et al. Safety and efficacy of eplerenone in patients at high risk for hyperkalemia and/or worsening renal function: Analyses of the EMPHASIS-HF study subgroups (eplerenone in mild patients hospitalization and survival study in heart failure). Journal of the American College of Cardiology 2013; 62:1585-1593.

26. Pitt B, Kober L, Ponikowski P, et al. Safety and tolerability of the novel non-steroidal mineralocorticoid receptor antagonist BAY 94-8862 in patients with chronic heart failure and mild or moderate chronic kidney disease: A randomized, double-blind trial. European Heart Journal 2013; 34:2453-2463.

27. Persson F, Lewis JB, Lewis EJ, Rossing P, Hollenberg NK, Parving HH. Impact of baseline renal function on the efficacy and safety of Aliskiren added to losartan in patients with type 2 diabetes and nephropathy. Diabetes Care 2010; 33:2304-2309.

28. Bhandari S, Mehta S, Khwaja A, et al. Renin–angiotensin system inhibition in advanced chronic kidney disease. New England Journal of Medicine 2022; 387:2021-2032.

29. Zhou Q, Yu W, Shao X, et al. EFFICACY AND SAFETY OF SACUBITRIL/VALSARTAN IN PATIENTS WITH STAGE 3B-5 CKD AND HYPERTENSION. Nephrology Dialysis Transplantation 2023b; 38:i665-i667.

30. Bornstein SR, de Zeeuw D, Heerspink HJ, et al. Aldosterone synthase inhibitor (BI 690517) therapy for people with diabetes and albuminuric chronic kidney disease: A multicentre, randomized, double‐blind, placebo‐controlled, Phase I trial. Diabetes, Obesity and Metabolism 2024; 26:2128-2138.

31. García-Prieto A, Verdalles Ú, de José AP, et al. Renin–angiotensin–aldosterone system blockers effect in chronic kidney disease progression in hypertensive elderly patients without proteinuria: PROERCAN trial. Hipertensión y Riesgo Vascular 2024; 41:95-103.

32. Tuttle KR, Hauske SJ, Canziani ME, et al. Efficacy and safety of aldosterone synthase inhibition with and without empagliflozin for chronic kidney disease: a randomised, controlled, phase 2 trial. The Lancet 2024; 403:379-390.

33. Guney I, Selcuk NY, Altintepe L, Atalay H, Başarali MK, Büyükbaş S. Antifibrotic effects of aldosterone receptor blocker (spironolactone) in patients with chronic kidney disease. Renal failure 2009; 31:779-784.

34. Edwards NC, Steeds RP, Stewart PM, Ferro CJ, Townend JN. Effect of spironolactone on left ventricular mass and aortic stiffness in early-stage chronic kidney disease: a randomized controlled trial. Journal of the American College of Cardiology 2009; 54:505-512.

35. Shang G, Gao Y, Liu K, Wang X. Serum potassium in elderly heart failure patients as a predictor of readmission within 1 year. Heart and vessels 2022.

36. Zhang J, He X, Wu J. The Impact of Hyperkalemia on Mortality and Healthcare Resource Utilization Among Patients With Chronic Kidney Disease: A Matched Cohort Study in China. Frontiers in public health 2022; 10:855395.

37. Grandy S, Jackson J, Moon R, Bluff D, Palaka E. Health-related quality of life and lifestyle changes in patients with chronic kidney disease and hyperkalaemia: Real-world data from the US, five European countries and China. International Journal of Clinical Practice 2021; 75:e14326.

38. Sharma A, Alvarez PJ, Woods SD, Dai D. A model to predict risk of hyperkalemia in patients with chronic kidney disease using a large administrative claims database. ClinicoEconomics and Outcomes Research 2020; 12:657-667.

39. Jimenez-Marrero S, Cainzos-Achirica M, Monterde D, et al. Real-world epidemiology of potassium derangements among chronic cardiovascular, metabolic and renal conditions: A population-based analysis. Clinical Epidemiology 2020; 12:941-952.

40. James G, Carrero JJ, Kumar S, et al. Pos-328 the Burden of Hyperkalemia in Patients with Chronic Kidney Disease: A Report from the Discover Ckd Retrospective Cohort. Kidney International Reports 2021; 6:S141-S142.

41. Wang HH, Hung CC, Hwang DY, et al. Hypokalemia, Its Contributing Factors and Renal Outcomes in Patients with Chronic Kidney Disease. PLoS ONE 2013; 8:e67140.

42. Jain N, Kotla S, Little BB, et al. Predictors of hyperkalemia and death in patients with cardiac and renal disease. American Journal of Cardiology 2012; 109:1510-1513.

43. Wagner S, Metzger M, Flamant M, et al. Association of plasma potassium with mortality and end-stage kidney disease in patients with chronic kidney disease under nephrologist care - The NephroTest study. BMC Nephrology 2017; 18:295.

44. Jimenez-Marrero S, Cainzos-Achirica M, Monterde D, et al. Impact on clinical outcomes and health costs of deranged potassium levels in patients with chronic cardiovascular, metabolic, and renal conditions. Revista Espanola de Cardiologia 2021; 74:312-320.

45. Sadjadi SA, McMillan JI, Jaipaul N, Blakely P, Hline SS. A comparative study of the prevalence of hyperkalemia with the use of angiotensinconverting enzyme inhibitors versus angiotensin receptor blockers. Therapeutics and Clinical Risk Management 2009; 5:547-552.

46. Adelborg K, Nicolaisen SK, Hasvold P, Palaka E, Pedersen L, Thomsen RW. Predictors for repeated hyperkalemia and potassium trajectories in high-risk patients - A population-based cohort study. PLoS ONE 2019; 14:e0218739.

47. Linde C, Bakhai A, Furuland H, et al. Real-World Associations of Renin-Angiotensin-Aldosterone System Inhibitor Dose, Hyperkalemia, and Adverse Clinical Outcomes in a Cohort of Patients With New-Onset Chronic Kidney Disease or Heart Failure in the United Kingdom. Journal of the American Heart Association 2019; 8:e012655.

48. Pisoni R, Acelajado MC, Cartmill FR, et al. Long-term effects of aldosterone blockade in resistant hypertension associated with chronic kidney disease. Journal of human hypertension 2012; 26:502-6.

49. Johnson M, Morrison FJ, McMahon G, Su M, Turchin A. Outcomes in patients with cardiometabolic disease who develop hyperkalemia while treated with a renin-angiotensin-aldosterone system inhibitor. American Heart Journal 2023; 258:49-59.

50. Woo K-T, Choong H-L, Wong K-S, et al. A retrospective Aliskiren and Losartan study in non-diabetic chronic kidney disease. World journal of nephrology 2013; 2:129-35.

51. Salik JR, Golas SB, McCoy TH. A comparative study assessing the incidence and degree of hyperkalemia in patients on angiotensin-converting enzyme inhibitors versus angiotensin-receptor blockers. Journal of Human Hypertension 2022; 36:485-487.

52. Valdivielso JM, Carriazo S, Martin M, Fernandez-Fernandez B, Bermudez-López M, Ortiz A. Gender-specific risk factors and outcomes of hyperkalemia in CKD patients: smoking as a driver of hyperkalemia in men. Clinical Kidney Journal 2024; 17:sfad212.

53. Zhou J, Jin X, Zhou J, et al. Clinical outcomes by serum potassium levels for patients hospitalized for heart failure: Secondary analysis of data from the China National Heart Failure Registry. Clinical Cardiology 2023a; 46:1345-1352.

54. Qadir A, Ullah Z, Khalil MD, Saqib MN, Khan A, Khan A; Frequency of Hyperkalaemia in Non-Dialysis Dependent Chronic Kidney Disease (CKD) Patients.Medical Forum Monthly. 2023.

55. Perez-Navarro LM, Valdez-Ortiz R, Reyna-Blanco J. Prevalence and Factors Associated with Hyperkalemia in Outpatients with CKD. Journal of the American Society of Nephrology 2023; 34:397.

56. Jiménez-Marrero S, Cainzos-Achirica M, Monterde D, et al. Serum potassium abnormalities, renin-angiotensin-aldosterone system inhibitor discontinuation, and clinical outcomes in patients with chronic cardiovascular, metabolic, and renal conditions: A population-based analysis. European Journal of Internal Medicine 2024.

57. McFarland KL, Sheridan EA. A Retrospective Analysis of Sacubitril/Valsartan in Heart Failure and Chronic Kidney Disease. Journal of Pharmacy Technology 2023; 39:117-122.

58. Ding Y, Wan L, Zhang Z-c, et al. Effects of sacubitril-valsartan in patients undergoing maintenance dialysis. Renal Failure 2023; 45:2222841.

59. Svensson M, Kim K, Cars T, Rao N, Lesén E, Jarbrink K. EE268 Healthcare Costs and All-Cause Mortality Following a Hyperkalemia Event and Reduction of RAASi Therapy in Sweden. Value in Health 2023; 26:S103.

60. Calabrese V, Cernaro V, Battaglia V, et al. Correlation between Hyperkalemia and the duration of several hospitalizations in patients with chronic kidney disease. Journal of Clinical Medicine 2022; 11:244.

61. Hayes J, Kalantar-Zadeh K, Lu JL, Turban S, Anderson JE, Kovesdy CP. Association of Hypo- and hyperkalemia with disease progression and mortality in males with chronic kidney disease: The role of race. Nephron - Clinical Practice 2012; 120:C8-C16.

62. Iskandar H, Hidayat WU, Hersunaryati Y, Syamsudin. A retrospective study on the potential drug interaction between angiotensin converting enzyme inhibitor or angiotensin receptor antagonist and other drugs in end-stage chronic renal failure patients. International Research Journal of Pharmacy 2012; 3:86-89.

63. Parmar S, Ali M, Lopez T, Shah R, Anderson L, Banerjee D. WCN24-2118 HYPERKALAEMIA IN PATIENTS WITH CHRONIC KIDNEY DISEASE AND HEART FAILURE. Kidney International Reports 2024; 9:S327-S328.

64. Chinnadurai R, Rengarajan S, Budden JJ, Quinn CM, Kalra PA. Maintaining Renin-Angiotensin-Aldosterone System Inhibitor Treatment with Patiromer in Hyperkalaemic Chronic Kidney Disease Patients: Comparison of a Propensity-Matched Real-World Population with AMETHYST-DN. American Journal of Nephrology 2023; 54:408-415.

65. Li X, Li B, Guo Y. Impact of hyperkalemia on hospitalization days in advanced chronic kidney disease patients with Type-2 diabetes mellitus: A prospective study. Pakistan Journal of Medical Sciences 2023; 39:885-890.

66. Hwang J-C, Wang C-T, Chen C-A, Chen H-C. Hypokalemia is associated with increased mortality rate in chronic hemodialysis patients. Blood purification 2011; 32:254-61.

67. Li JF, Qu X, Gao Z, et al. Association between dosing of spironolactone and outcomes in heart failure with preserved ejection fraction patients combined with chronic kidney disease------Balance of efficacy and risk. Frontiers in Pharmacology 2023; 14:1084442.

68. Khosla N, Kalaitzidis R, Bakris GL. Predictors of hyperkalemia risk following hypertension control with aldosterone blockade. American journal of nephrology 2009; 30:418-24.

69. Frohlich H, Nelges C, Tager T, et al. Long-term changes of renal function in relation to ace inhibitor/angiotensin receptor blocker dosing in patients with heart failure and chronic kidney disease. American Heart Journal 2016; 178:28-36.

70. Riccio E, Capuano I, Buonanno P, et al. RAAS Inhibitor Prescription and Hyperkalemia Event in Patients With Chronic Kidney Disease: A Single-Center Retrospective Study. Frontiers in Cardiovascular Medicine 2022; 9:824095.

71. Qu X, Yao H, Chen C, et al. Spironolactone Improves the All-Cause Mortality and Re-Hospitalization Rates in Acute Myocardial Infarction with Chronic Kidney Disease Patients. Frontiers in Pharmacology 2021; 12:632978.

72. Hirai T, Yamaga R, Fujita A, Itoh T. Low body mass index is a risk factor for hyperkalaemia associated with angiotensin converting enzyme inhibitors and angiotensin II receptor blockers treatments. Journal of clinical pharmacy and therapeutics 2018; 43:829-835.

73. Marup FH, Peters C, Nielsen S, et al. POTASSIUM LEVELS AND EGFR DO NOT PREDICT SEVERE HYPERKALEMIA FOLLOWING SPIRONOLACTONE INTRODUCTION IN PATIENTS WITH CKD AT HIGH RISK OF HYPERKALEMIA. Nephrology Dialysis Transplantation 2023; 38:i668-i669.

74. Lin DSH, Lin FJ, Lin YS, Lee JK, Lin YH. The effects of mineralocorticoid receptor antagonists on cardiovascular outcomes in patients with end-stage renal disease and heart failure. European Journal of Heart Failure 2023; 25:98-107.

75. Wetmore JB, Yan H, Horne L, Peng Y, Gilbertson DT. Risk of hyperkalemia from renin-angiotensin-aldosterone system inhibitors and factors associated with treatment discontinuities in a real-world population. Nephrology Dialysis Transplantation 2021; 36:826-839.

76. De Rosa ML, Cardace P, Rossi M, Baiano A, De Cristofaro A. Evaluation of long-term efficacy and tolerability of irbesartan in elderly hypertensive patients with renal impairment in an open-label study. Current Therapeutic Research - Clinical and Experimental 2002; 63:201-215.

77. De Rosa ML, De Cristofaro A, Rossi M, et al. Irbesartan effects on renal function in patients with renal impairment and hypertension: A drug-withdrawal study. Journal of Cardiovascular Pharmacology 2001; 38:482-489.

78. Jun M, Jardine MJ, Perkovic V, et al. Hyperkalemia and renin-angiotensin aldosterone system inhibitor therapy in chronic kidney disease: A general practice-based, observational study. PLoS ONE 2019; 14:e0213192.

79. de Rooij EN, de Fijter JW, Le Cessie S, et al. Serum potassium and risk of death or kidney replacement therapy in older people with CKD stages 4-5: eight-year follow-up. American Journal of Kidney Diseases 2023; 82:257-266. e1.

80. Tanaka K, Saito H, Iwasaki T, et al. Association between serum potassium levels and adverse outcomes in chronic kidney disease: the Fukushima CKD cohort study. Clinical and Experimental Nephrology 2021; 25:410-417.

81. Gwoo S, Kim YN, Shin HS, Jung YS, Rim H. Predictors of hyperkalemia risk after hypertension control with aldosterone blockade according to the presence or absence of chronic kidney disease. Nephron - Clinical Practice 2014; 128:381-386.

82. Gülçiçek S, Seyahi N. Hyperkalemia: A Cause of Non-adherence to Renin-Angiotensin-Aldosterone System Inhibitors in Chronic Kidney Disease: A Retrospective Study. Istanbul Medical Journal 2023; 24.

83. Wang J, Wang F. WCN23-1101 Instantaneous and persistent elevation of serum potassium and progression of chronic kidney disease, a single center-based cohort study. Kidney International Reports 2023; 8:S159.

84. Bakris GL, Agiro A, Greatsinger A, et al. REVOLUTIONIZE III: Consequences of Recurrent Hyperkalemia on Healthcare Resource Utilization and Cost. Journal of the American Society of Nephrology 2023; 34:190-191.

85. An J, Zhou H, Ni L, et al. Discontinuation of renin-angiotensin-aldosterone system inhibitors secondary to hyperkalemia translates into higher cardiorenal outcomes. American Journal of Nephrology 2023; 54:258-267.

86. Kohsaka S, Okami S, Kanda E, Kashihara N, Yajima T. Cardiovascular and Renal Outcomes Associated With Hyperkalemia in Chronic Kidney Disease: A Hospital-Based Cohort Study. Mayo Clinic Proceedings: Innovations, Quality and Outcomes 2021; 5:274-285.

87. Kanda E, Kashihara N, Kohsaka S, Okami S, Yajima T. Clinical and Economic Burden of Hyperkalemia: A Nationwide Hospital-Based Cohort Study in Japan. Kidney Medicine 2020; 2:742-752.e1.

88. Kashihara N, Kohsaka S, Kanda E, Okami S, Yajima T. Hyperkalemia in Real-World Patients Under Continuous Medical Care in Japan. Kidney International Reports 2019; 4:1248-1260.

89. Wang J, Wang F. WCN23-1101 Instantaneous and persistent elevation of serum potassium and progression of chronic kidney disease, a single center-based cohort study. Kidney International Reports 2023; 8:S159.

90. Nakhoul GN, Huang H, Arrigain S, et al. Serum Potassium, End-Stage Renal Disease and Mortality in Chronic Kidney Disease. American Journal of Nephrology 2015; 41:456-463.

91. Korgaonkar S, Tilea A, Gillespie BW, et al. Serum potassium and outcomes in CKD: Insights from the RRI-CKD cohort study. Clinical Journal of the American Society of Nephrology 2010; 5:762-769.

92. Knoll GA, Sahgal A, Nair RC, Graham J, van Walraven C, Burns KD. Renin-angiotensin system blockade and the risk of hyperkalemia in chronic hemodialysis patients. The American journal of medicine 2002; 112:110-4.

93. Sengul E, Sahin T, Sevin E, Yilmaz A. Effect of spironolactone on urinary protein excretion in patients with chronic kidney disease. Renal Failure 2009; 31:928-932.

94. Yang A, Shi M, Lau ESH, et al. Clinical outcomes following discontinuation of renin-angiotensin-system inhibitors in patients with type 2 diabetes and advanced chronic kidney disease: A prospective cohort study. eClinicalMedicine 2023; 55:101751.

95. Buckallew AR, Tellor KB, Watson R, et al. Evaluation of the safety and tolerability of spironolactone in patients with heart failure and chronic kidney disease. European Journal of Clinical Pharmacology 2021; 77:955-960.

96. Ren H, Leon SJ, Whitlock R, et al. Prescription patterns of sodium and calcium polystyrene sulfonate in patients with hyperkalemia and chronic kidney disease receiving RAAS inhibitors. Clinical Kidney Journal 2022; 15:1713-1719.

97. Uchida HA, Nakajima H, Hashimoto M, et al. Efficacy and Safety of Esaxerenone in Hypertensive Patients with Diabetic Kidney Disease: A Multicenter, Open-Label, Prospective Study. Advances in Therapy 2022; 39:5158-5175.

98. Santoro A, Perrone V, Giacomini E, Sangiorgi D, Alessandrini D, Degli Esposti L. Association between hyperkalemia, RAASi non-adherence and outcomes in chronic kidney disease. Journal of Nephrology 2022; 35:463-472.

99. Johnson ES, Weinstein JR, Thorp ML, et al. Predicting the risk of hyperkalemia in patients with chronic kidney disease starting lisinopril. Pharmacoepidemiology and Drug Safety 2010; 19:266-272.

100. Maddirala S, Khan A, Vincent A, Lau K. Effect of angiotensin converting enzyme inhibitors and angiotensin receptor blockers on serum potassium levels and renal function in ambulatory outpatients: Risk factors analysis. American Journal of the Medical Sciences 2008; 336:330-335.

101. Saito Y, Yamamoto H, Nakajima H, Takahashi O, Komatsu Y. Incidence of and risk factors for newly diagnosed hyperkalemia after hospital discharge in non-dialysis-dependent CKD patients treated with RAS inhibitors. PLoS ONE 2017; 12:e0184402.

102. An J, Niu F, Sim JJ. Cardiovascular and kidney outcomes of spironolactone or eplerenone in combination with ACEI/ARBs in patients with diabetic kidney disease. Pharmacotherapy 2021; 41:998-1008.

103. Lee J-H, Kwon YE, Park JT, et al. The effect of renin-angiotensin system blockade on renal protection in chronic kidney disease patients with hyperkalemia. Journal of the renin-angiotensin-aldosterone system : JRAAS 2014; 15:491-7.

104. Heshka J, Ruzicka M, Hiremath S, McCormick BB. Spironolactone for difficult to control hypertension in chronic kidney disease: An analysis of safety and efficacy. Journal of the American Society of Hypertension 2010; 4:295-301.

105. Leon SJ, Whitlock R, Rigatto C, et al. Hyperkalemia-Related Discontinuation of Renin-Angiotensin-Aldosterone System Inhibitors and Clinical Outcomes in CKD: A Population-Based Cohort Study. American Journal of Kidney Diseases 2022; 80:164-173.e1.

106. Silvarino R, Rios P, Baldovinos G, et al. Is Chronic Kidney Disease Progression Influenced by the Type of Renin-Angiotensin-System Blocker Used? Nephron 2019; 143:100-107.

107. Sevamontree C, Jintajirapan S, Phakdeekitcharoen P, Phakdeekitcharoen B. The Prevalence and Risk Factors of Hyperkalemia in the Outpatient Setting. International Journal of Nephrology 2024; 2024.

108. Furuland H, McEwan P, Evans M, et al. Serum potassium as a predictor of adverse clinical outcomes in patients with chronic kidney disease: New risk equations using the UK clinical practice research datalink. BMC Nephrology 2018; 19:211.

109. Luo J, Brunelli SM, Jensen DE, Yang A. Association between serum potassium and outcomes in patients with reduced kidney function. Clinical Journal of the American Society of Nephrology 2016; 11:90-100.

110. Brookes EM, Snider J, Hart GK, Robbins R, Power DA. Serum potassium abnormalities in chronic kidney disease: prevalence, patient characteristics and clinical outcomes. Internal Medicine Journal 2021; 51:1906-1918.

111. Einhorn LM, Zhan M, Hsu VD, et al. The frequency of hyperkalemia and its significance in chronic kidney disease. Archives of Internal Medicine 2009; 169:1156-1162.

112. Obertynska O. The risks and benefits of spironolactone use in heart failure with a reduced left ventricular ejection fraction and chronic kidney disease. European Heart Journal 2021; 42:895.

113. Raebel MA, Ross C, Xu S, et al. Diabetes and drug-associated hyperkalemia: Effect of potassium monitoring. Journal of General Internal Medicine 2010; 25:326-333.

114. Belmar Vega L, Galabia ER, Bada da Silva J, et al. Epidemiology of hyperkalemia in chronic kidney disease. Nefrologia 2019; 39:277-286.

115. Collins AJ, Pitt B, Reaven N, et al. Association of Serum Potassium with All-Cause Mortality in Patients with and without Heart Failure, Chronic Kidney Disease, and/or Diabetes. American journal of nephrology 2017; 46:213-221.

116. Neuenschwander JF, Silverstein AR, Teigland CL, et al. The Increased Clinical and Economic Burden of Hyperkalemia in Medicare Patients Admitted to Long-Term Care Settings. Advances in therapy 2023; 40:1204-1223.

117. Kanda E, Rastogi A, Murohara T, et al. Clinical impact of suboptimal RAASi therapy following an episode of hyperkalemia. BMC Nephrology 2023; 24:18.

118. Polson M, Lord TC, Kangethe A, et al. Clinical and Economic Impact of Hyperkalemia in Patients with Chronic Kidney Disease and Heart Failure. Journal of managed care & specialty pharmacy 2017; 23:S2-S9.

119. Fang G, Annis IE, Farley JF, et al. Incidence of and Risk Factors for Severe Adverse Events in Elderly Patients Taking Angiotensin-Converting Enzyme Inhibitors or Angiotensin II Receptor Blockers after an Acute Myocardial Infarction. Pharmacotherapy 2018; 38:29-41.

120. Sharma A, Alvarez PJ, Woods SD, Fogli J, Dai D. Healthcare resource utilization and costs associated with hyperkalemia in a large managed care population. Journal of Pharmaceutical Health Services Research 2021; 12:35-41.

121. Betts KA, Woolley JM, Mu F, Wang Y, Dua A, Wu EQ. Postdischarge Health Care Costs and Readmission in Patients With Hyperkalemia-Related Hospitalizations. Kidney International Reports 2020; 5:1280-1290.

122. Mu F, Betts KA, Woolley JM, et al. Prevalence and economic burden of hyperkalemia in the United States Medicare population. Current Medical Research and Opinion 2020; 36:1333-1341.

123. James G, Carrero JJ, Khezrian M, et al. Pos-305 Hospitalizations and Length of Stay in Patients with Ckd with and without Hyperkalemia: A Report from the Discover Ckd Retrospective Cohort. Kidney International Reports 2022; 7:S136-S137.

124. Thomsen RW, Nicolaisen SK, Hasvold P, et al. Elevated potassium levels in patients with chronic kidney disease: Occurrence, risk factors and clinical outcomes-a Danish population-based cohort study. Nephrology Dialysis Transplantation 2018; 33:1610-1620.

125. Betts KA, Woolley JM, Mu F, Xiang C, Tang W, Wu EQ. The Cost of Hyperkalemia in the United States. Kidney international reports 2018; 3:385-393.

126. Tseng WC, Liu JS, Hung SC, et al. Effect of spironolactone on the risks of mortality and hospitalization for heart failure in pre-dialysis advanced chronic kidney disease: A nationwide population-based study. International Journal of Cardiology 2017; 238:72-78.

127. Rysava R, Tesar V, Merta M. Effect of telmisartan on blood pressure control and kidney function in hypertensive, proteinuric patients with chronic kidney disease. Blood Pressure Monitoring 2005; 10:207-213.

128. Agarwal R, Joseph A, Anker SD, et al. Hyperkalemia Risk with Finerenone: Results from the FIDELIO-DKD Trial. Journal of the American Society of Nephrology 2022; 33:225-237.

129. Zhang H; Effect of Finerenone on CKD outcomes in type 2 diabetes: A chinese subgroup analysis of the FIDELIO-DKD study.Kidney week. Journal of American Society of Nephrology 2022b.

130. Filippatos G, Anker SD, Agarwal R, et al. Finerenone and Cardiovascular Outcomes in Patients with Chronic Kidney Disease and Type 2 Diabetes. Circulation 2021; 143:540-552.

131. Rossing P, Filippatos G, Agarwal R, et al. Finerenone in Predominantly Advanced CKD and Type 2 Diabetes With or Without Sodium-Glucose Cotransporter-2 Inhibitor Therapy. Kidney International Reports 2022; 7:36-45.

132. Rossing P, Burgess E, Agarwal R, et al. Finerenone in Patients With Chronic Kidney Disease and Type 2 Diabetes According to Baseline HbA1c and Insulin Use: An Analysis From the FIDELIO-DKD Study. Diabetes Care 2022; 45:888-897.

133. Zhang H, Xie J, Hao C, et al. Finerenone in Patients with Chronic Kidney Disease and Type 2 Diabetes: The FIDELIO-DKD Subgroup from China. Kidney Diseases 2023; 9:498-506.

134. Agrawal A, Kamila S, Reddy S, Lilly J, Mariyala MS. Effect of telmisartan on kidney function in patients with chronic kidney disease: an observational study. Journal of drug assessment 2016; 5:24-28.

135. Yildirim T, Arici M, Piskinpasa S, et al. Major barriers against renin-angiotensin-aldosterone system blocker use in chronic kidney disease stages 3-5 in clinical practice: a safety concern? Renal failure 2012; 34:1095-9.

136. Tokunaga M, Kabashima N, Serino R, et al. Renoprotective effects of telmisartan in patients with advanced chronic kidney disease. Clinical nephrology 2010; 73:139-46.

137. Fu EL, Evans M, Clase CM, et al. Stopping renin-angiotensin system inhibitors in patients with advanced CKD and risk of adverse outcomes: a nationwide study. Journal of the American Society of Nephrology: JASN 2021; 32:424.

138. Ahmed AK, Kamath NS, El Kossi M, El Nahas AM. The impact of stopping inhibitors of the renin–angiotensin system in patients with advanced chronic kidney disease. Nephrology Dialysis Transplantation 2010; 25:3977-3982.

139. Gaol DL, Nilasari D, Halim DS, Cahyantari RA. WCN24-2258 SERUM POTASSIUM PROFILE AND ASSOCIATED FACTORS IN HEMODIALYSIS PATIENTS: SINGLE CENTER STUDY. Kidney International Reports 2024; 9:S8.

140. Rastogi A, Pollack C, Lesen E, et al. Association Between Reduced RAASi Therapy and Progression to ESKD in Hyperkalemic CKD Patients. Journal of the American Society of Nephrology 2023; 34:379.

141. Rajak K, Halder A, Khanal R, Atrash A. RENAL DYSFUNCTION ASSOCIATED WITH FINERENONE - A PHARMACOVIGILANCE ANALYSIS. Journal of the American College of Cardiology 2023; 81:583.

142. Agiro A, AN A, Cook EE, et al. Real-world modifications of renin-angiotensin-aldosterone system inhibitors in patients with hyperkalemia initiating sodium zirconium cyclosilicate therapy: the OPTIMIZE I study. Advances in Therapy 2023; 40:2886-2901.

143. Gregg LP, Richardson P, Herrera MA, et al. Documented adverse drug reactions and discontinuation of angiotensin converting enzyme inhibitors and angiotensin receptor blockers in chronic kidney disease. Am J Nephrol 2023; 54:126-35.

144. Nicholas SB, Correa-Rotter R, Desai N, et al. Interim Results from FINE-REAL: A Prospective Study Providing Insights into the Use of Finerenone in Routine Clinical Settings. Journal of the American Society of Nephrology 2023; 34:857.

145. Jariwala P, Pramod G. Effect of Fineronone on Heart Failure Outcomes in Type 2 Diabetes with Chronic Kidney Disease: An Early Indian Experience. Indian Heart Journal 2023; 75:S49.
